# Potassium Aluminyl Promoted Carbonylation of Ethene

**DOI:** 10.1002/anie.202117396

**Published:** 2022-02-23

**Authors:** Matthew J. Evans, Samuel E. Neale, Mathew D. Anker, Claire L. McMullin, Martyn P. Coles

**Affiliations:** ^1^ School of Chemical and Physical Sciences Victoria University of Wellington PO Box 600 Wellington 6012 New Zealand; ^2^ Department of Chemistry University of Bath Bath BA2 7AY UK

**Keywords:** Aluminyl Anions, Bimetallic Complexes, Carbonylation, Hydrogen-Shift, Metallacycles

## Abstract

The potassium aluminyl [K{Al(NON^Dipp^)}]_2_ ([NON^Dipp^]^2−^=[O{SiMe_2_NDipp}_2_]^2−^, Dipp=2,6‐*i*Pr_2_C_6_H_3_) activates ethene towards carbonylation with CO under mild conditions. An isolated bis‐aluminacyclopropane compound reacted with CO via carbonylation of an Al−C bond, followed by an intramolecular hydrogen shift to form K_2_[Al(NON^Dipp^)(*μ*‐CH_2_CH=CO‐1κ^2^ *C* ^1,3^‐2κ*O*)Al(NON^Dipp^)Et]. Restricting the chemistry to a mono‐aluminium system allowed isolation of [Al(NON^Dipp^)(CH_2_CH_2_CO‐κ^2^ *C* ^1,3^)]^−^, which undergoes thermal isomerisation to form the [Al(NON^Dipp^)(CH_2_CH=CHO‐κ^2^ *C*,*O*)]^−^ anion. DFT calculations highlight the stabilising influence of incorporated benzene at multiple steps in the reaction pathways.

## Graphical abstract

Metal‐promoted carbonylation of alkenes is established for *d*‐block metals, whilst prior work with aluminium has been limited by reversible C=C coordination and decomposition of CO insertion products. This work shows how the aluminyl anion allows the non‐reversible carbonylation of ethene using carbon monoxide under mild conditions. A bimetallic pathway involving an intramolecular 1,2‐hydrogen shift was identified and supported by DFT calculations.

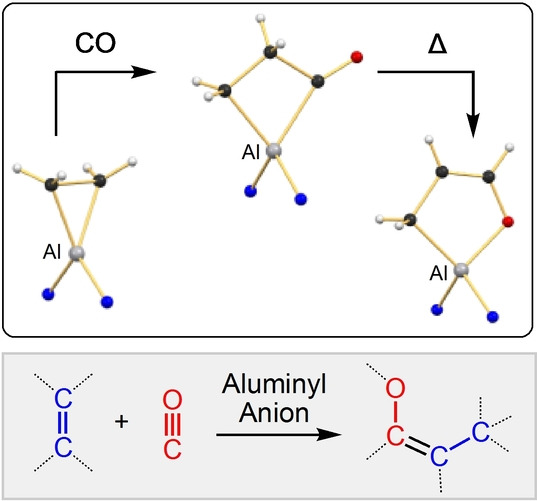

Carbonylation defines the introduction of CO into a substrate, either by insertion into a C−X bond or by addition to an unsaturated compound.^[1]^ The ′traditional′ mechanism involves insertion of an alkene into a metal–hydride or ‐alkyl bond, followed by insertion of CO into the M−C bond (Scheme 1, Mechanism 1). Alternatively, an initial oxidative cyclisation of a low‐valent metal with an alkene to form a metallacyclopropane, followed by CO insertion, affords a cyclic carbonylation product (Mechanism 2). Overall, the carbonylation reaction underpins several quintessential chemical processes including hydroformylation^[2]^ and alkoxy‐/hydro‐carboxylation,^[3]^ and is utilised on an industrial scale for the production of bulk and fine chemicals.^[4]^ The majority of carbonylation reactions are promoted by transition metals,^[5]^ with palladium, cobalt and rhodium taking the lead in this field.^[6]^ During the past two decades, research in main group chemistry has demonstrated that many characteristics believed to be exclusive to *d*‐block elements are now accessible by *p*‐block metals.^[7]^ This offers opportunities for the development of more sustainable main group metal promoted small molecule activation reactions with synthetic utility.

**Scheme 1:**
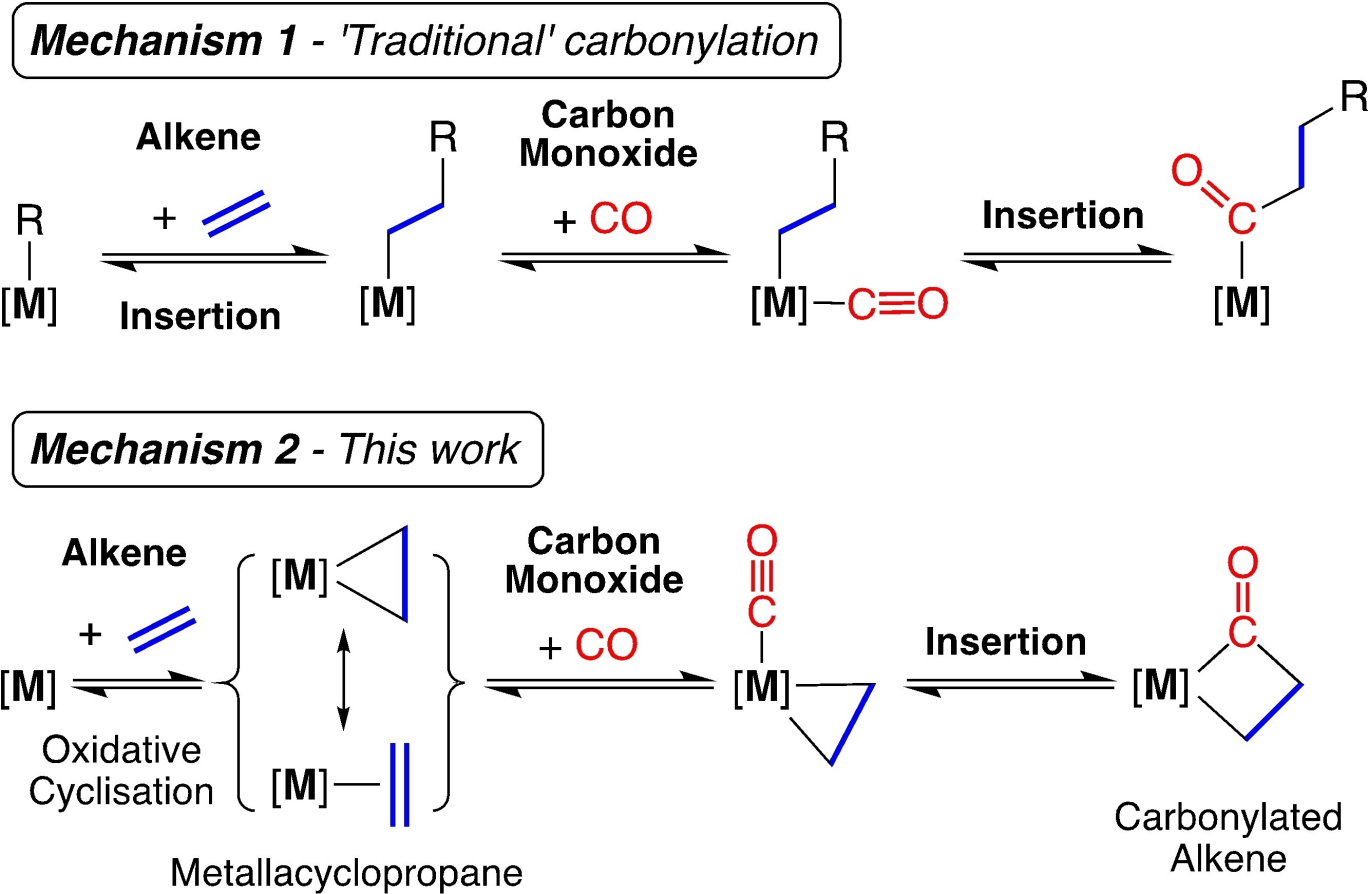
Metal‐promoted carbonylation reactions involving ethene and CO.

Low oxidation state aluminium compounds play a prominent role in advancing main group metal research.^[8]^ Within this context, a significant body of Al^I^ research has focused on the ability of the neutral, two‐coordinate species, (BDI^Dipp^)Al (BDI^Dipp^=[HC{CMeNDipp}_2_]^−^) to activate small molecules.^[9]^ Recently this field has expanded to include anionic Al^I^ complexes (aluminyl anions),^[10]^ fuelling significant developments in the field.^[11]^

The reactivity of both neutral and anionic Al^I^ systems with unsaturated organic fragments has been explored. The addition of alkenes and alkynes to (BDI^Dipp^)Al formed the corresponding aluminacyclopropane^[12]^ and aluminacyclopropene^[13]^ compounds, with analogous chemistry recently observed with a dialkyl aluminyl salt.^[14]^ Despite this interest, structurally characterised examples of the parent ethene complexes are limited to two systems; the neutral aluminacyclopropane **I**,^[12b]^ and the aluminyl derived dimer, [**II**]_2_ (Figure 1).^[15]^ In both products the coordinated ethene is described as containing an ′activated′ carbon–carbon bond, although this was not exploited in any reactivity studies. In this contribution we describe two pathways leading to the non‐reversible carbonylation of ethene by CO, affording stable products under mild conditions.

**Figure 1:**
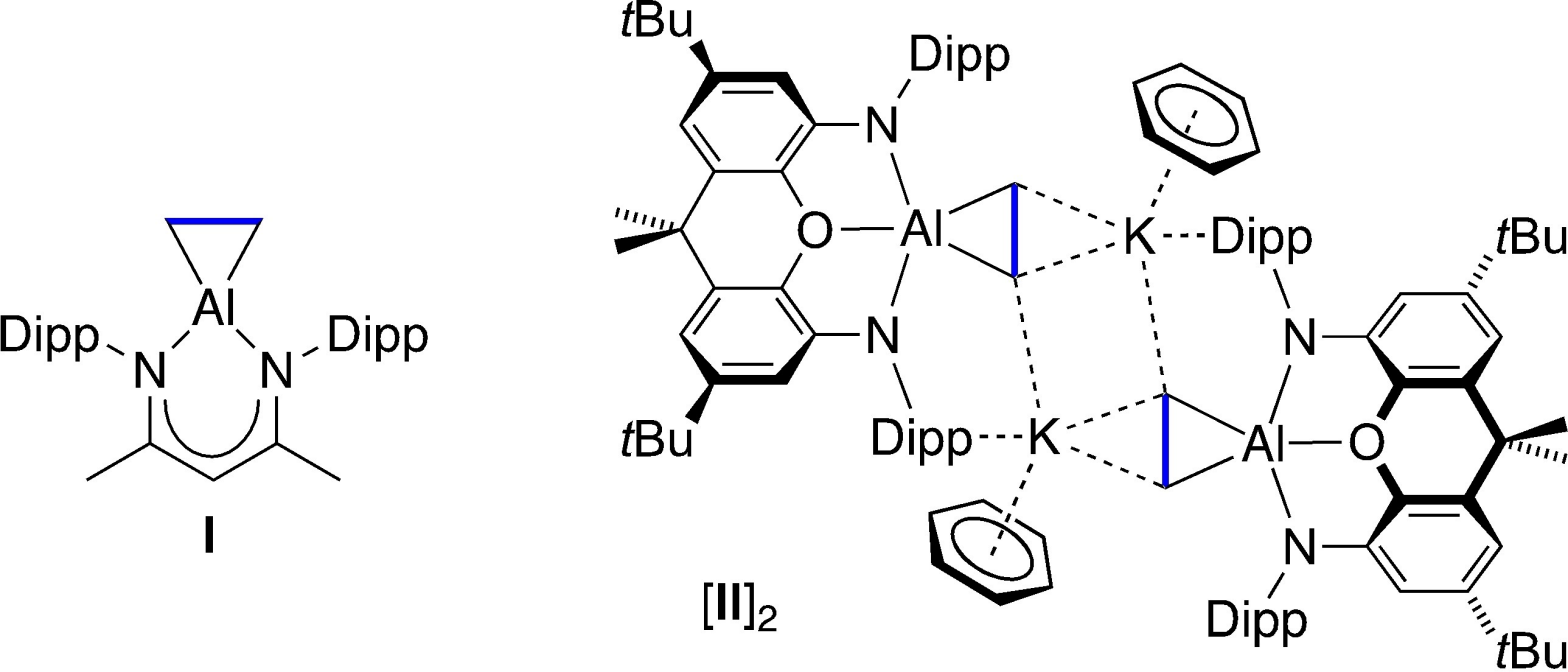
Structurally characterised aluminacyclopropanes.

The potassium aluminyl [K{Al(NON^Dipp^)}]_2_ ^[10b]^ was reacted with 1 bar of ethene to afford **1** (Scheme 2). The ^1^H NMR spectrum of **1** in C_6_D_6_ showed a high field singlet at *δ*_H_=−1.40 (4H) assigned to a symmetrical aluminacyclopropane. This resonance is at significantly higher field than the corresponding signal in **I** (*δ*_H_=0.67) and [**II**]_2_ (*δ*_H_=−0.70), which we attribute to an aromatic solvent induced shift. This postulate is supported by a low field shift of the corresponding resonance in D_8_‐THF (*δ*_H_=−0.81. Figure S1b). In contrast to **I**,^[12b]^ there is no evidence for release of the alkene on heating (343 K, Figure S5), offering the opportunity to study the reactivity of this group.

**Scheme 2:**
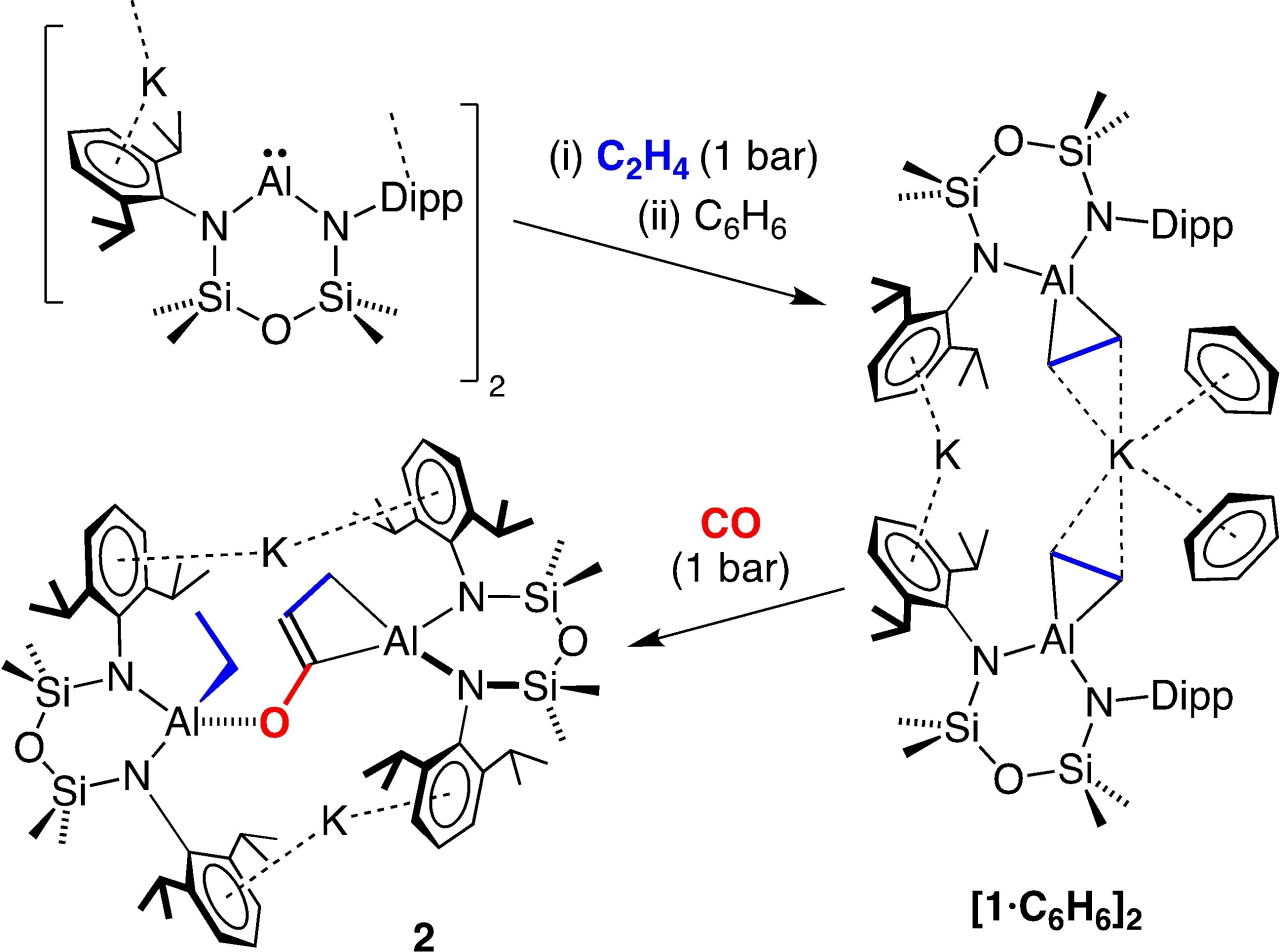
Synthesis of **[1**⋅**C_6_H_6_]_2_** and **2**.

Compound **1** crystallised from benzene as the non‐symmetric bis(aluminacyclopropane) **[1**⋅**C_6_H_6_]_2_** ⋅**C_6_H_6_** (Figure 2).^[16]^ Density functional theory (DFT) calculations identified that formation of the benzene‐deficient dimer **[1]_2_** from **[1**⋅**C_6_H_6_]_2_** is endergonic by +7.9 kcal mol^−1^ (Figure S20). The greater stability of the benzene included structure likely results from a decreased steric congestion compared with **[1]_2_**, in addition to the formation of stabilising K⋅⋅⋅π(arene) interactions on solvation of the potassium. The crystal structure shows that K1 contacts both C_2_H_4_ units (K⋅⋅⋅C range: 3.030(2) Å–3.119(2) Å) and two *η* ^6^‐benzene ligands, whilst K2 bridges two [Al(NON^Dipp^)(C_2_H_4_)]^−^ anions via K⋅⋅⋅Dipp interactions. The C−C bond lengths in the aluminacyclopropane groups (1.587(2) Å and 1.596(2) Å) correspond to an increase of 18.8 % and 19.5 % compared to the experimentally determined value for ethene.^[17]^ Similar increases were noted in **I** (1.577(2) Å, 18.0 % increase) and [**II**]_2_ (1.598(2) Å, 19.6 %).^[18]^

**Figure 2:**
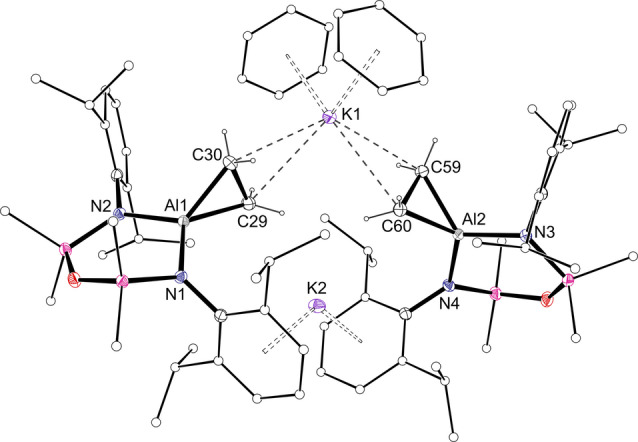
Displacement ellipsoid plot of **[1**⋅**C_6_H_6_]_2_** ⋅**C_6_H_6_** (ellipsoids 30 %; benzene solvate and H‐atoms except C_2_ *H_4_* omitted; peripheral C‐atoms represented as spheres). Selected bond lengths [Å]: C29−C30 1.587(2), C59−C60 1.596(2), Al1−C29 1.9669(18), Al1−C30 1.9510(17), Al2−C59 1.9473(16), Al2−C60 1.9632(18).

Inspired by the non‐reversible coordination of ethene in **[1**⋅**C_6_H_6_]_2_**, we examined the reactivity of the coordinated alkene towards carbonylation with CO gas. The reaction proceeded under ambient conditions (1 bar CO, room temperature) to afford dark orange crystals (**2**) on work‐up (Scheme 2). The ^1^H NMR spectrum contained unanticipated signals for an aluminium ethyl group, evident as a quartet at *δ*_H_=−0.17 (2H) and a triplet at *δ*_H_=1.58 (3H). Resonances at *δ*_H_=6.22 (t, 1H) and *δ*_H_=0.48 (d, 2H) indicated a second organometallic ligand, with corresponding signals at *δ*_C_=184.5 (*C*) 132.9 (*C*H) and 11.0 (*C*H_2_) in the ^13^C{^1^H} NMR spectrum. These data indicate a new, three‐carbon moiety formed from the incorporation of one equivalent of CO.

X‐ray crystallography showed that **2** was a dialuminium compound, containing the hitherto unknown *μ*‐(prop‐1‐ene‐1,3‐diyl‐1κ^2^ *C*‐1‐olato‐2κ*O*) group, and confirming the presence of a terminal ethyl ligand (Figure 3).[^16^, ^19^] The [C_3_H_3_O]^3−^ ligand chelates to one aluminium through the 1,3‐carbon atoms (bite angle 75.56(13)°) with C−C distances showing a single and double bond. The exocyclic C−O distance (1.384(4) Å) indicates a single bond,^[20]^ with the Al−O2′ bond length of 1.802(2) Å consistent with an aluminium alkoxide group.

**Figure 3:**
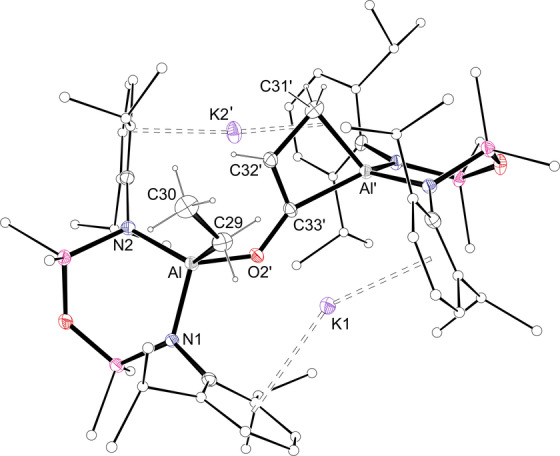
Displacement ellipsoid plot of **2** (ellipsoids 30 %; H‐atoms except Al*Et* and AlC_3_ *H_3_* O omitted; peripheral C‐atoms represented as spheres. ′=−*x*, *y*, 1/2 −*z*). Selected bond lengths [Å]: Al−O2′ 1.802(2), Al−C29 2.0144(19), C29−C30 1.578(5), Al′−C31′ 2.0144(19), Al′−C33′ 2.089(4), C31′−C32′ 1.636(4), C32′−C33′ 1.353(6), C33′−O2′ 1.384(4).

Previous reports of the reaction of Al−C bonds with CO describe formation of the expected insertion products.^[21]^ Most relevant, an aluminacyclopropane derived from norbornene was shown to form a propan‐1,3‐diyl‐1‐one group at the metal, containing C−C single bonds and a terminal C=O group.^[21b]^ However, this product was unstable at room temperature and decomposed to an unknown mixture of products. We propose that the formation of **2** involves an intramolecular β‐hydrogen shift from an initially formed propan‐1,3‐diyl‐1‐one ligand at one aluminium centre to an unreacted aluminacyclopropane. The energetics of this reaction have been determined using DFT calculations, with the results highlighting the role of the incorporated benzene in facilitating this process (Figure 4).

**Figure 4:**
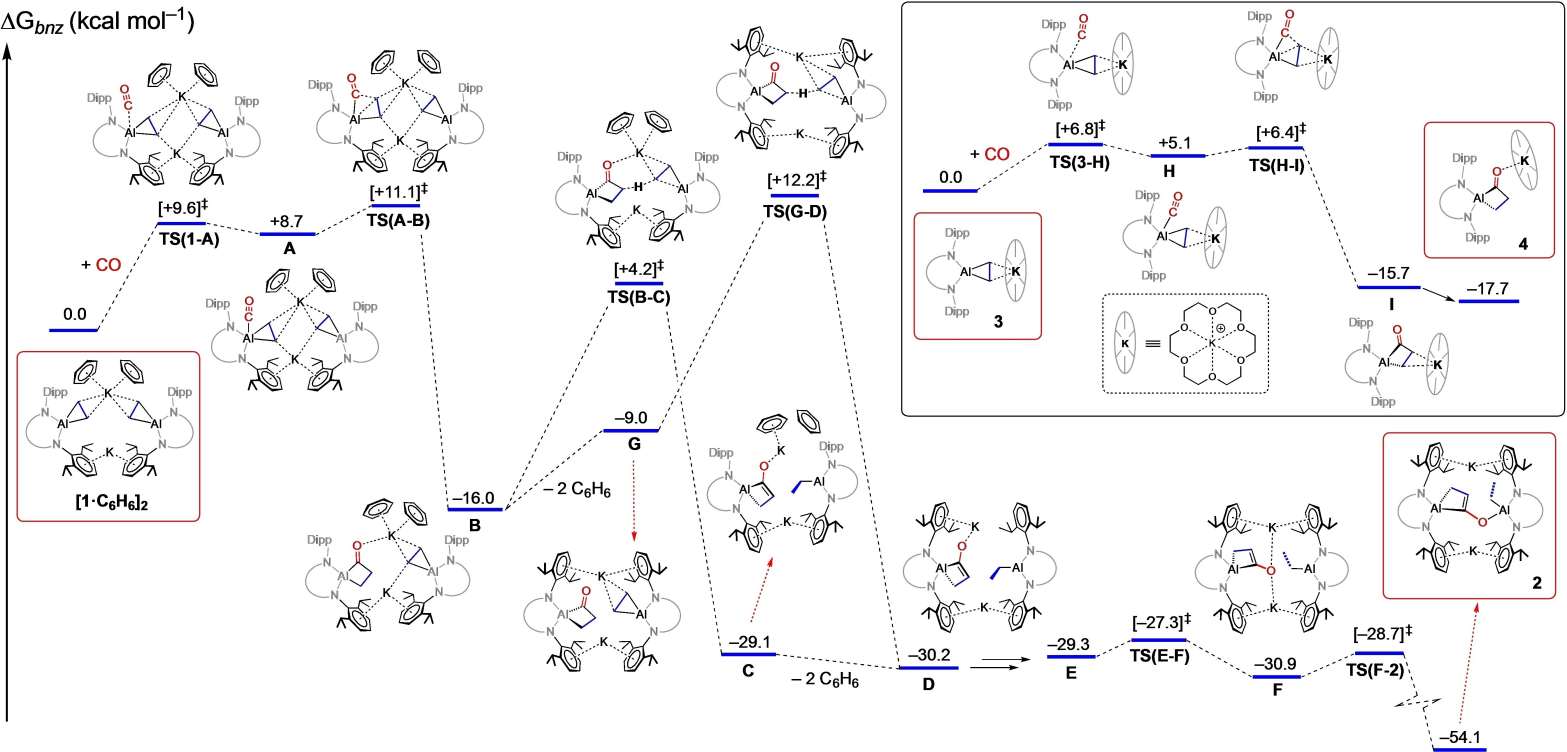
DFT‐calculated free energy profile at the BP86‐D3(BJ)−C_6_H_6_/6‐311++G**//BP86/BS1 level (in kcal mol^−1^) for the conversion of **[1**⋅**C_6_H_6_]_2_** to **2**. Inset: Conversion of **3** to **4**.

The computed results show that coordination of CO to **[1**⋅**C_6_H_6_]_2_** occurs at one of the aluminium centres via **TS(1‐A)** to afford **A**, with an activation barrier of +9.6 kcal mol^−1^. Subsequent C−C coupling takes place via **TS(A**‐**B)** (+11.1 kcal mol^−1^) to afford the asymmetric dimer **B** containing a propan‐1,3‐diyl‐1‐one ligand. The aforementioned stabilising influence of the benzene molecules is confirmed in this pathway, as the corresponding energies for CO addition (+15.2 kcal mol^−1^) and C−C bond formation (+15.8 kcal mol^−1^) are higher when explicit solvent molecules are absent (Figure S20). From **B**, rate‐limiting hydrogen transfer from the propan‐1,3‐diyl‐1‐one moiety to the intact aluminacyclopropane occurs via **TS(B**‐**C)** with an overall energetic span of +20.2 kcal mol^−1^, forming the prop‐1‐ene‐1,3‐diyl‐1‐olate ligand in **C** (−29.1 kcal mol^−1^) with concomitant formation of the aluminium ethyl ligand. The two non‐covalently bound benzene rings once again have a positive stabilising effect on this step, reducing the energy of the H‐transfer transition state and thus kinetically accelerating the process. This is evidenced by the characterisation of a disfavoured benzene deficient H‐transfer process via **TS(G**‐**D)** (+12.2 kcal mol^−1^). Dissociation of benzene from **C** forms **D** (−30.2 kcal mol^−1^), allowing for rearrangement of the AlC_3_H_3_O ring and facile Al−O bond formation to form **2**.

To promote formation of a mono‐aluminium species thereby blocking the pathway that leads to **2**, 18‐crown‐6 was added to **1** (Scheme 3). The ^1^H NMR spectrum of the isolated product **3** indicated retention of a symmetrical aluminacyclopropane unit *δ*_H_=−0.69 (s, 4H), confirmed by X‐ray diffraction (Scheme 3).^[16]^ K⋅⋅⋅C contacts to the C_2_H_4_ group are present (3.0242(14) Å and 3.0526(14) Å), and the aluminacyclopropane C−C distance (1.596(2) Å, 19.5 % increase) does not change significantly to that in **[1**⋅**C_6_H_6_]_2_**.

**Scheme 3:**
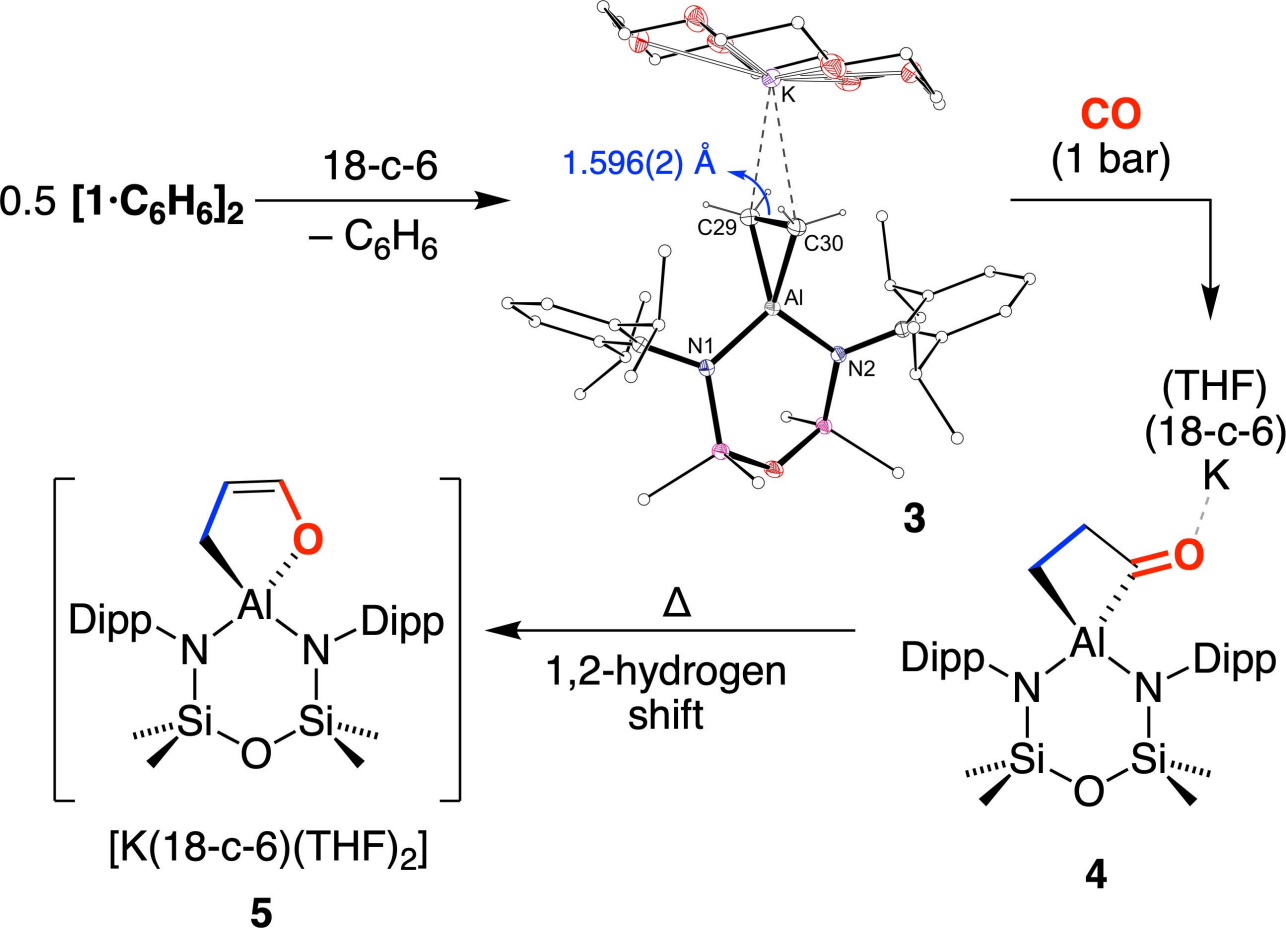
Synthesis of **3**, **4** and **5** (18‐c‐6=18‐crown‐6).

The reaction of **3** with CO afforded a new compound **4** on work‐up. ^1^H NMR analysis shows two high field methylene resonances at *δ*_H_=−0.24 and *δ*_H_=1.06 indicating a de‐symmetrisation of the aluminacyclopropane. The corresponding ^13^C{^1^H} NMR peaks appear at *δ*_C_=3.5 and 53.0, although an anticipated low field *C*O resonance could not be observed, likely due to its quaternary nature and proximity to quadrupolar ^27^Al (*I*=5/2). X‐ray diffraction data confirmed the solid‐state structure of **4**⋅**THF** as [K(18‐c‐6)(THF)][Al(NON^Dipp^)(C_3_H_4_O)] (Figure 5a).^[16]^ The anion contains a propan‐1,3‐diyl‐κ^2^ *C*‐1‐one ligand at aluminium (bite angle 73.36(15)°), with a contact between the exocyclic oxygen and the potassium cation (O3⋅⋅⋅K=2.720(3) Å). The bonding within this unit indicates C−C single bonds and a terminal C=O double bond (C31−O2=1.183(5) Å), consistent with the only other structurally characterised example of CO insertion into a cyclic AlC_2_ unit.^[21b]^

**Figure 5:**
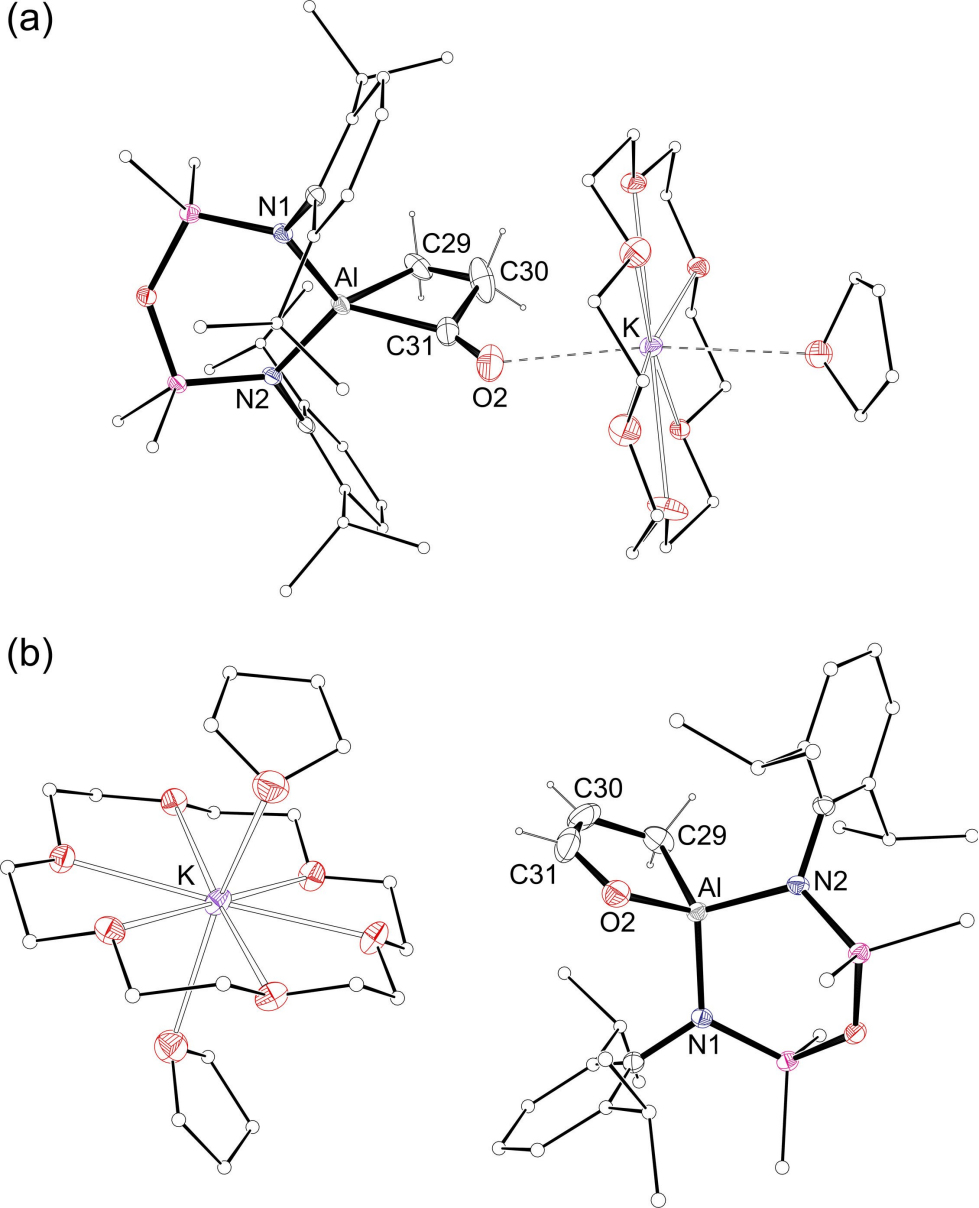
Displacement ellipsoid plots of **4**⋅**THF** (ellipsoids 20 %) and **5**⋅**THF** (ellipsoids 30 %). Disordered atoms, THF solvate and H‐atoms except AlC_3_ *H_4_* O omitted; peripheral C‐atoms represented as spheres). Selected bond lengths [Å]: a) Al−C29 2.020(3), Al−C31 2.048(4), C29−C30 1.481(6), C30−C31 1.576(6), C31−O2 1.183(5). b) Al−C29 1.975(2), Al−O2 1.8095(16), C29−C30 1.523(4), C30−C31 1.317(5), C31−O2 1.432(3).

The carbonylation of **3** was also examined by DFT (Figure 4: Inset). Rate‐limiting CO addition gives the carbonyl adduct **H** via **TS(3‐H)**, with an activation barrier of +6.8 kcal mol^−1^, lower than that for CO addition to **[1**⋅**C_6_H_6_]_2_**. This suggests that CO uptake is favoured in the monomeric species, presumably due to the greater accessibility of the aluminium centre compared to **[1**⋅**C_6_H_6_]_2_**. Formation of **H** is followed by facile C−C coupling via **TS(H**‐**I)** (+6.4 kcal mol^−1^), with subsequent rearrangement to yield **4**. An alternative pathway in which dissociation of [K(18‐c‐6)]^+^ occurs prior to CO coordination and C−C coupling was characterised (Figure S21), showing that initial separation to produce the solvent separated ion pair is highly endergonic (+29.3 kcal mol^−1^). The barrier to C−C bond formation in this process is kinetically inaccessible by comparison (**TS(K**‐**L)** +38.5 kcal mol^−1^).

In contrast to the facile intramolecular β‐hydrogen transfer that occurs during the formation of **2**, the propan‐1,3‐diyl‐κ^2^ *C‐*1‐one ligand in **4** appears to be relatively stable. DFT modelling of a corresponding *inter*molecular hydrogen transfer process comparable to that leading to **2**, involving addition of a second equivalent of **3** to **4** (albeit via loss of two [K(18‐c‐6)]^+^ cations), was found to be strongly disfavoured, with the corresponding transition‐state, **TS(L**‐**M)**, at +79.2 kcal mol^−1^.

The stability of the C_3_H_4_O‐metallacycle in **4** was also noted experimentally. There was no reaction of **4** with H_2_ at room temperature, although upon heating to 80 °C under a H_2_ atmosphere, a new species **5** was formed. The conversion of **4** to **5** proceeds over 5 days with consumption of the propan‐1,3‐diyl‐1‐one resonances in the ^1^H NMR spectrum, and formation of new multiplets at *δ*_H_=6.00 (dt, 1H), *δ*_H_=4.01 (q, 1H) and *δ*_H_=−0.44 (dd, 2H). Further investigation showed that **5** also formed under N_2_, indicating that hydrogen was not incorporated in the product and suggesting that **5** is a product of thermal isomerisation (Scheme 3). This was confirmed by X‐ray diffraction, showing a 1‐propen‐1‐olate ligand containing endocyclic C−C single (C29−C30=1.523(4) Å) and double (C30−C31=1.317(5) Å) bonds (Figure 5b).^[16]^ We propose that **5** forms via an intra‐annular 1,2‐hydrogen shift from the β‐position, akin to the hydrogen transfer that generates **2**. Although there are no examples of this isomerisation within the coordination sphere of a metal, it is reminiscent of the 1,2‐proton shift proposed by Bergman and co‐workers during the CO insertion in a N−H bond of a parent amido.^[22]^

In summary, we have reported the non‐reversible carbonylation of ethene using carbon monoxide under mild conditions, promoted by a potassium aluminyl. A bimetallic pathway involving an intramolecular hydrogen shift was identified, with DFT calculations highlighting the stabilising role of the coordinated benzene molecules. Restriction to a mono‐aluminium system enabled isolation of the C_3_H_4_O‐carbonylation product. This metallacycle undergoes a thermally induced 1,2‐hydrogen shift and ring‐expansion to generate an unsaturated aluminacycle, in which the integrity of the C_3_O‐framework is retained, thereby demonstrating the stability of the C−C bond formed between ethylene and CO. These results show that the conversion of inert substrates into more elaborate molecules containing synthetically useful functional groups for further reactivity can be achieved under ambient conditions using earth abundant and non‐toxic main group metals.

## Conflict of interest

The authors declare no conflict of interest.

## Supporting information

As a service to our authors and readers, this journal provides supporting information supplied by the authors. Such materials are peer reviewed and may be re‐organized for online delivery, but are not copy‐edited or typeset. Technical support issues arising from supporting information (other than missing files) should be addressed to the authors.

Supporting Information

Supporting Information

## Data Availability

The data that support the findings of this study are available from the corresponding author upon reasonable request.

## References

[1] J.-B. Peng , H.-Q. Geng , X.-F. Wu , Chem 2019, 5, 526–552.

[2a] R. Franke , D. Selent , A. Börner , Chem. Rev. 2012, 112, 5675–5732; 22937803 10.1021/cr3001803

[2b] K.-D. Wiese , D. Obst , in: Topics in Organometallic Chemistry (Ed.: M. Beller ), Springer, Berlin, Heidelberg, 2006, pp. 1–33.

[3] B. El Ali , H. Alper , in: Transition Metals for Organic Synthesis, Vol. 1 (Eds.: M. Beller , C. Bolm ), Wiley-VCH, Weinheim, 2004.

[4] W. Bertleff , M. Roeper , X. Sava , in: Ullmann's Encyclopedia of Industrial Chemistry, Vol. 7, Wiley-VCH, Weinheim, 2012, pp. 73–98.

[5] M. Beller , X.-F. Wu , Transition Metal Catalyzed Carbonylation Reactions, Springer, Berlin, Heidelberg, 2013.

[6a] X.-F. Wu , X. Fang , L. Wu , R. Jackstell , H. Neumann , M. Beller , Acc. Chem. Res. 2014, 47, 1041–1053; 24564478 10.1021/ar400222k

[6b] X.-F. Wu , H. Neumann , M. Beller , ChemSusChem 2013, 6, 229–241. 23307763 10.1002/cssc.201200683

[7a] C. Weetman , S. Inoue , ChemCatChem 2018, 10, 4213–4228;

[7b] P. P. Power , Nature 2010, 463, 171–177. 20075912 10.1038/nature08634

[8a] K. Hobson , C. J. Carmalt , C. Bakewell , Chem. Sci. 2020, 11, 6942–6956; 34122993 10.1039/d0sc02686g PMC8159300

[8b] Y. Liu , J. Li , X. Ma , Z. Yang , H. W. Roesky , Coord. Chem. Rev. 2018, 374, 387–415;

[8c] M. Asay , C. Jones , M. Driess , Chem. Rev. 2011, 111, 354–396. 21133370 10.1021/cr100216y

[9] M. Zhong , S. Sinhababu , H. W. Roesky , Dalton Trans. 2020, 49, 1351–1364. 31942579 10.1039/c9dt04763h

[10a] J. Hicks , P. Vasko , J. M. Goicoechea , S. Aldridge , Nature 2018, 557, 92–95; 29662211 10.1038/s41586-018-0037-y

[10b] R. J. Schwamm , M. D. Anker , M. Lein , M. P. Coles , Angew. Chem. Int. Ed. 2019, 58, 1489–1493; 10.1002/anie.201811675 30548141

[10c] R. J. Schwamm , M. P. Coles , M. S. Hill , M. F. Mahon , C. L. McMullin , N. A. Rajabi , A. S. S. Wilson , Angew. Chem. Int. Ed. 2020, 59, 3928–3932; 10.1002/anie.201914986 PMC7159655 31830364

[10d] S. Kurumada , S. Takamori , M. Yamashita , Nat. Chem. 2020, 12, 36–39; 31767993 10.1038/s41557-019-0365-z

[10e] K. Koshino , R. Kinjo , J. Am. Chem. Soc. 2020, 142, 9057–9062; 32321239 10.1021/jacs.0c03179

[10f] S. Grams , J. Eyselein , J. Langer , C. Färber , S. Harder , Angew. Chem. Int. Ed. 2020, 59, 15982–15986; 10.1002/anie.202006693 PMC7540686 32449816

[10g] M. J. Evans , M. D. Anker , C. L. McMullin , S. E. Neale , M. P. Coles , Angew. Chem. Int. Ed. 2021, 60, 22289–22292; 10.1002/anie.202108934 34402149

[10h] R. J. Schwamm , M. S. Hill , H.-Y. Liu , M. F. Mahon , C. L. McMullin , N. A. Rajabi , Chem. Eur. J. 2021, 27, 14971–14980; 34403562 10.1002/chem.202102682 PMC8596455

[10i] M. M. D. Roy , J. Hicks , P. Vasko , A. Heilmann , A.-M. Baston , J. M. Goicoechea , S. Aldridge , Angew. Chem. Int. Ed. 2021, 60, 22301–22306; 10.1002/anie.202109416 34396660

[10j] T. X. Gentner , M. J. Evans , A. R. Kennedy , S. E. Neale , C. L. McMullin , M. P. Coles , R. E. Mulvey , Chem. Commun. 2022, 58, 1390–1393. 10.1039/d1cc05379e 34994367

[11] J. Hicks , P. Vasko , J. M. Goicoechea , S. Aldridge , Angew. Chem. Int. Ed. 2021, 60, 1702–1713; 10.1002/anie.202007530 32567755

[12a] C. Bakewell , A. J. P. White , M. R. Crimmin , Angew. Chem. Int. Ed. 2018, 57, 6638–6642; 10.1002/anie.201802321 29645324

[12b] C. Bakewell , A. J. P. White , M. R. Crimmin , Chem. Sci. 2019, 10, 2452–2458; 30881673 10.1039/c8sc04865g PMC6388093

[12c] C. Bakewell , M. Garçon , R. Y. Kong , L. O'Hare , A. J. P. White , M. R. Crimmin , Inorg. Chem. 2020, 59, 4608–4616. 32207927 10.1021/acs.inorgchem.9b03701

[13a] C. Cui , S. Köpke , R. Herbst-Irmer , H. W. Roesky , M. Noltemeyer , H.-G. Schmidt , B. Wrackmeyer , J. Am. Chem. Soc. 2001, 123, 9091–9098; 11552816 10.1021/ja003185i

[13b] H. Zhu , J. Chai , H. Fan , H. W. Roesky , C. He , V. Jancik , H.-G. Schmidt , M. Noltemeyer , W. A. Merrill , P. P. Power , Angew. Chem. Int. Ed. 2005, 44, 5090–5093; 10.1002/anie.200500899 16015667

[13c] H. Zhu , R. B. Oswald , H. Fan , H. W. Roesky , Q. Ma , Z. Yang , H.-G. Schmidt , M. Noltemeyer , K. Starke , N. S. Hosmane , J. Am. Chem. Soc. 2006, 128, 5100–5108. 16608344 10.1021/ja057731p

[14] K. Sugita , R. Nakano , M. Yamashita , Chem. Eur. J. 2020, 26, 2174–2177. 31880356 10.1002/chem.201905830

[15] J. Hicks , P. Vasko , J. M. Goicoechea , S. Aldridge , J. Am. Chem. Soc. 2019, 141, 11000–11003. 31251586 10.1021/jacs.9b05925

[16] Deposition numbers 2126652 (for **[1**⋅**C** _ **6** _ **H** _ **6** _ **]** _ **2** _⋅**C** _ **6** _ **H** _ **6** _), 2126653 (for **2**), 2126654 (for **3**), 2126655 (for **4**⋅**THF**), and 2126656 (for **5**⋅**THF**) contain the supplementary crystallographic data for this paper. These data are provided free of charge by the joint Cambridge Crystallographic Data Centre and Fachinformationszentrum Karlsruhe Access Structures service.

[17] L. S. Bartell , E. A. Roth , C. D. Hollowell , K. Kuchitsu , J. E. Young , J. Chem. Phys. 1965, 42, 2683–2686.

[18] The hydrogen atom positions of the aluminacyclopropane groups were located and refined and, mindful of the limitations of accurately locating hydrogen atom positions from X-ray diffraction data, a distinctly pyramidal geometry is implied at the carbon atoms (Σ_HCH/CCH_ angles 323(3)°–338(3)°).

[19] The molecule lies on a 2-fold rotation access, with a single Al center and both the Al*Et* and Al(*CH_2_CHO*) ligands in the asymmetric unit. These were modelled at 50 % occupancy with the CH_2_ groups of each component (C29/C31) coincident. The exceptionally long C31′−C32′ bond length (1.636(4) Å) is an artifact of this model, and DFT calculations performed on **3** indicate a bond length of 1.515 Å, within the normal range for C−C single bonds.

[20] P. Pyykkö , M. Atsumi , Chem. Eur. J. 2009, 15, 186–197. 19058281 10.1002/chem.200800987

[21a] S. Fujimori , S. Inoue , J. Am. Chem. Soc. 2022, 144, 2034–2050; 35068141 10.1021/jacs.1c13152

[21b] R. Y. Kong , M. R. Crimmin , Chem. Commun. 2019, 55, 6181–6184; 10.1039/c9cc02818h 31086911

[21c] R. Y. Kong , M. R. Crimmin , J. Am. Chem. Soc. 2018, 140, 13614–13617; 30351139 10.1021/jacs.8b09761

[21d] X. Li , C. Ni , H. Song , C. Cui , Chem. Commun. 2006, 1763–1765; 10.1039/b601056c 16609797

[21e] M. R. Mason , B. Song , K. Kirschbaum , J. Am. Chem. Soc. 2004, 126, 11812–11813. 15382914 10.1021/ja046411n

[22] D. J. Fox , R. G. Bergman , J. Am. Chem. Soc. 2003, 125, 8984–8985. 15369333 10.1021/ja035707a

