# Supplementary material for: Potassium Aluminyl Promoted Carbonylation of Ethene
Source: Angew Chem Int Ed Engl. 2022 Feb 23;61(16):e202117396. doi: 10.1002/anie.202117396 (PMC9307019; doi:10.1002/anie.202117396)
Supplement: Supplementary file 2 — Supporting Information [file ANIE-61-0-s001.pdf]

Angewandte Chemie
Eine Zeitschrift der Gesellschaft Deutscher Chemiker

Supporting Information

Potassium Aluminyl Promoted Carbonylation of Ethene

M. J. Evans, S. E. Neale, M. D. Anker, C. L. McMullin*, M. P. Coles**

S3 General experimental procedures
S3 Synthesis of $K[Al(NON^{Dipp})(C_2H_4)]_2$ (**1**) and $[K_2(C_6H_6)_2][Al(NON^{Dipp})(C_2H_4)]_2$ (**[1·C$_6$H$_6$]$_2$**)
S5 **Figure S1** ^{1}H NMR spectrum $K[Al(NON^{Dipp})(C_2H_4)]_2$ (**1**) in C_6D_6
S6 **Figure S1b** ^{1}H NMR spectrum $K[Al(NON^{Dipp})(C_2H_4)]_2$ (**1**) in D_8-THF
S7 **Figure S2** $^{13}C\{^1H\}$ NMR spectrum $K[Al(NON^{Dipp})(C_2H_4)]_2$ (**1**) in C_6D_6
S8 **Figure S2b** $^{13}C\{^1H\}$ NMR spectrum $K[Al(NON^{Dipp})(C_2H_4)]_2$ (**1**) in D_8-THF
S9 **Figure S3** ^{1}H NMR spectrum $[K_2(C_6H_6)_2][Al(NON^{Dipp})(C_2H_4)]_2$ (**[1·C$_6$H$_6$]$_2$**)
S10 **Figure S4** $^{13}C\{^1H\}$ NMR spectrum $[K_2(C_6H_6)_2][Al(NON^{Dipp})(C_2H_4)]_2$ (**[1·C$_6$H$_6$]$_2$**)
S11 **Figure S5** Variable temperature ^{1}H NMR spectra $K[Al(NON^{Dipp})(C_2H_4)]_2$ (**1**)
S12 **Figure S6** Displacement ellipsoid plot of $[K_2(C_6H_6)_2][Al(NON^{Dipp})(C_2H_4)]_2 \cdot C_6H_6$ (**[1·C$_6$H$_6$]$_2$·C$_6$H$_6$**)
S13 Synthesis of $K_2[Al(NON^{Dipp})(\mu\text{-}CH_2CH{=}CO)Al(NON^{Dipp})Et]$ (**2**)
S14 **Figure S7** ^{1}H NMR spectrum $K_2[Al(NON^{Dipp})(\mu\text{-}CH_2CH{=}CO)Al(NON^{Dipp})Et]$ (**2**)
S15 **Figure S8** $^{13}C\{^1H\}$ NMR spectrum $K_2[Al(NON^{Dipp})(\mu\text{-}CH_2CH{=}CO)Al(NON^{Dipp})Et]$ (**2**)
S16 **Figure S8b** HSQC NMR spectrum $K_2[Al(NON^{Dipp})(\mu\text{-}CH_2CH{=}CO)Al(NON^{Dipp})Et]$ (**2**)
S17 **Figure S8c** HMBC NMR spectrum $K_2[Al(NON^{Dipp})(\mu\text{-}CH_2CH{=}CO)Al(NON^{Dipp})Et]$ (**2**)
S18 **Figure S9** Displacement ellipsoid plot $K_2[Al(NON^{Dipp})(\mu\text{-}CH_2CH{=}CO)Al(NON^{Dipp})Et]$ (**2**)
S19 Synthesis of $[K(18\text{-crown-}6)][Al(NON^{Dipp})(C_2H_4)]$ (**3**)
S20 **Figure S10** ^{1}H NMR spectrum $[K(18\text{-crown-}6)][Al(NON^{Dipp})(C_2H_4)]$ (**3**)
S21 **Figure S11** $^{13}C\{^1H\}$ NMR spectrum $[K(18\text{-crown-}6)][Al(NON^{Dipp})(C_2H_4)]$ (**3**)
S22 **Figure S12** Displacement ellipsoid plot $[K(18\text{-crown-}6)][Al(NON^{Dipp})(C_2H_4)]$ (**3**)
S23 Synthesis of $[K(18\text{-crown-}6)(THF)][Al(NON^{Dipp})(CH_2CH_2C{=}O)]$ (**4**)
S24 **Figure S13** ^{1}H NMR spectrum $[K(18\text{-crown-}6)(THF)][Al(NON^{Dipp})(CH_2CH_2C{=}O)]$ (**4**)
S25 **Figure S14** $^{13}C\{^1H\}$ NMR spectrum $[K(18\text{-crown-}6)(THF)][Al(NON^{Dipp})(CH_2CH_2C{=}O)]$ (**4**)
S26 **Figure S15** Displacement ellipsoid plot $[K(18\text{-crown-}6)(THF)][Al(NON^{Dipp})(CH_2CH_2C{=}O)] \cdot THF$ (**4·THF**)
S27 Synthesis of $[K(18\text{-c-}6)(THF)_2][Al(NON^{Dipp})(CH_2CH{=}CHO)]$ (**5**)
S28 **Figure S16** ^{1}H NMR spectrum $[K(18\text{-c-}6)(THF)_2][Al(NON^{Dipp})(CH_2CH{=}CHO)]$ (**5**)
S29 **Figure S17** $^{13}C\{^1H\}$ NMR spectrum $[K(18\text{-c-}6)(THF)_2][Al(NON^{Dipp})(CH_2CH{=}CHO)]$ (**5**)
S30 **Figure S18** Displacement ellipsoid plot $[K(18\text{-c-}6)(THF)_2][Al(NON^{Dipp})(CH_2CH{=}CHO)] \cdot THF$ (**5·THF**)
S31 Crystallographic Details
S32 **Table S1** Crystallographic details for **[1·C$_6$H$_6$]$_2$·C$_6$H$_6$, 2** and **3**
S33 **Table S2** Crystallographic details for **4·THF** and **5·THF**
S34 Computational Details
S35 **Figure S19** DFT-Calculated Free Energy Profile for the conversion of **[1·C$_6$H$_6$]$_2$** to **2**
S36 **Figure S20** DFT-Calculated Free Energy Profile for the conversion of **[1·C$_6$H$_6$]$_2$** to **III** via a benzene dissociation pathway

S37 **Figure S21** DFT-Calculated Free Energy Profile for the conversion of **3** to **4**
S38 Breakdown of Energy Contributions
S39 **Table S3** Relative energies for computed structures.
S40 References
S42 Cartesian Coordinates and Energies of Computed Structures

General Synthetic Procedures

All manipulations were performed under dry nitrogen or argon using standard Schlenk-line techniques, or in a conventional nitrogen-filled glovebox. Hexane, toluene, diethyl ether (Et_2O), and tetrahydrofuran (THF) were obtained from a PureSolv MD 5 system and stored over activated 5Å molecular sieves for 24 hours prior to use. NMR spectra were recorded using a Jeol JNM-ECZ500S 500 MHz spectrometer equipped with a ROYAL digital auto tune probe S, operating at 500.1 (^{1}H) and 125.8 (^{13}C). Spectra were recorded at 294 K (unless stated otherwise) and proton and carbon chemical shifts were referenced internally to residual solvent resonances. Coupling constants are quoted in Hz.

$[K\{Al(NON^{Dipp})\}]_2$ was prepared according to the literature procedures.[1] All other chemicals were purchased from Sigma-Aldrich and used without further purification.

Synthesis of $K[Al(NON^{Dipp})(C_2H_4)]$ (1) and $[K_2(C_6H_6)_2][Al(NON^{Dipp})(C_2H_4)]_2$ ($[1{\cdot}C_6H_6]_2$)

A benzene solution of $[K\{Al(NON^{Dipp})\}]_2$ (520 mg, 0.95 mmol) was prepared in an ampule and sealed under nitrogen using a Teflon tap. The solution was degassed, and ethene (~1.5 bar) introduced to the reaction vessel. The solution was allowed to stir for 18 hours at room temperature to afford a pale-yellow solution. Crystallisation was achieved at room temperature *via* slow evaporation from a benzene solution, affording **$[1{\cdot}C_6H_6]_2$** as colorless crystals. Yield 433 mg, 66 %.

The non-solvated compound $K[Al(NON^{Dipp})(C_2H_4)]$ (**1**) was obtained as a pure white powder by removing the benzene *in vacuo*, washing the residue washed with hexane (3 × 10 mL).

Compound 1 (*NMR spectrum recorded under C_2H_4 atmosphere*). ^{1}H NMR (500 MHz, C_6D_6): δ 7.04 (d, *J* = 7.6, 4H, C_6H_3), 6.92 (t, *J* = 7.6, 2H, C_6H_3), 4.21 (sept, *J* = 6.8, 4H, C*H*Me$_2$), 1.38 (d, *J* = 6.8, 12H, CH*Me$_2$*), 1.28 (s, 12H, CH*Me$_2$*), 0.45 (s, 12H, Si*Me$_2$*), −1.40 (s, 4H, =C*H$_2$*).

^{13}C{^{1}H} NMR (126 MHz, C_6D_6): δ 148.0, 147.6, 123.6, 122.5 (*C$_6$*H$_3$), 27.5 (*C*HMe$_2$), 25.4 (CH*Me$_2$*), 2.6 (Si*Me$_2$*), 1.9 (=*C*H$_2$).

Compound 1 (*NMR spectrum recorded on dried/desolvated crystals*). ^{1}H NMR (500 MHz, D_8-THF): δ 6.85 (d, *J* = 7.6, 4H, C_6H_3), 6.72 (t, *J* = 7.6, 2H, C_6H_3), 4.10 (sept, *J* = 6.8, 4H, C*H*Me$_2$), 1.22 (d, *J* = 6.8, 12H, CH*Me*$_2$), 1.15 (s, 12H, CH*Me*$_2$), 0.07 (s, 12H, Si*Me*$_2$), –0.81 (s, 4H, =C*H*$_2$).

^{13}C{^{1}H} NMR (126 MHz, C_6D_6): δ 147.4, 123.5, 123.1, 121.5 (*C*$_6$H$_3$), 28.0 (*C*HMe$_2$), 26.1 (CH*Me*$_2$), 2.8 (Si*Me*$_2$), 1.8 (=*C*H$_2$).

Compound [1·C_6H_6]$_2$ (*isolated crystals from C_6H_6*). ^{1}H NMR (500 MHz, C_6D_6): δ 7.16 (s, 6H, C_6H_6), 7.05 (d, *J* = 7.6, 4H, C_6H_3), 6.92 (t, *J* = 7.6, 2H, C_6H_3), 4.23 (sept, *J* = 6.8, 4H, C*H*Me$_2$), 1.39 (d, *J* = 6.8, 12H, CH*Me*$_2$), 1.28 (d, *J* = 6.8, 12H, CH*Me*$_2$), 0.47 (s, 12H, Si*Me*$_2$), –1.39 (s, 4H, =C*H*$_2$).

^{13}C NMR (126 MHz, C_6D_6): δ 148.0, 147.6, 128.6, 123.6 (*C*$_6$H$_3$), 122.5 (*C*$_6$H$_6$), 27.5 (*C*HMe$_2$), 25.4 (CH*Me*$_2$), 2.6 (Si*Me*$_2$), 2.0 (=*C*H$_2$).

Figure S1 ^{1}H NMR spectrum (500 MHz, C_6D_6) of K[Al(NON^{Dipp})(C_2H_4)] (**1**) (*NMR spectrum recorded under C_2H_4 atmosphere*)

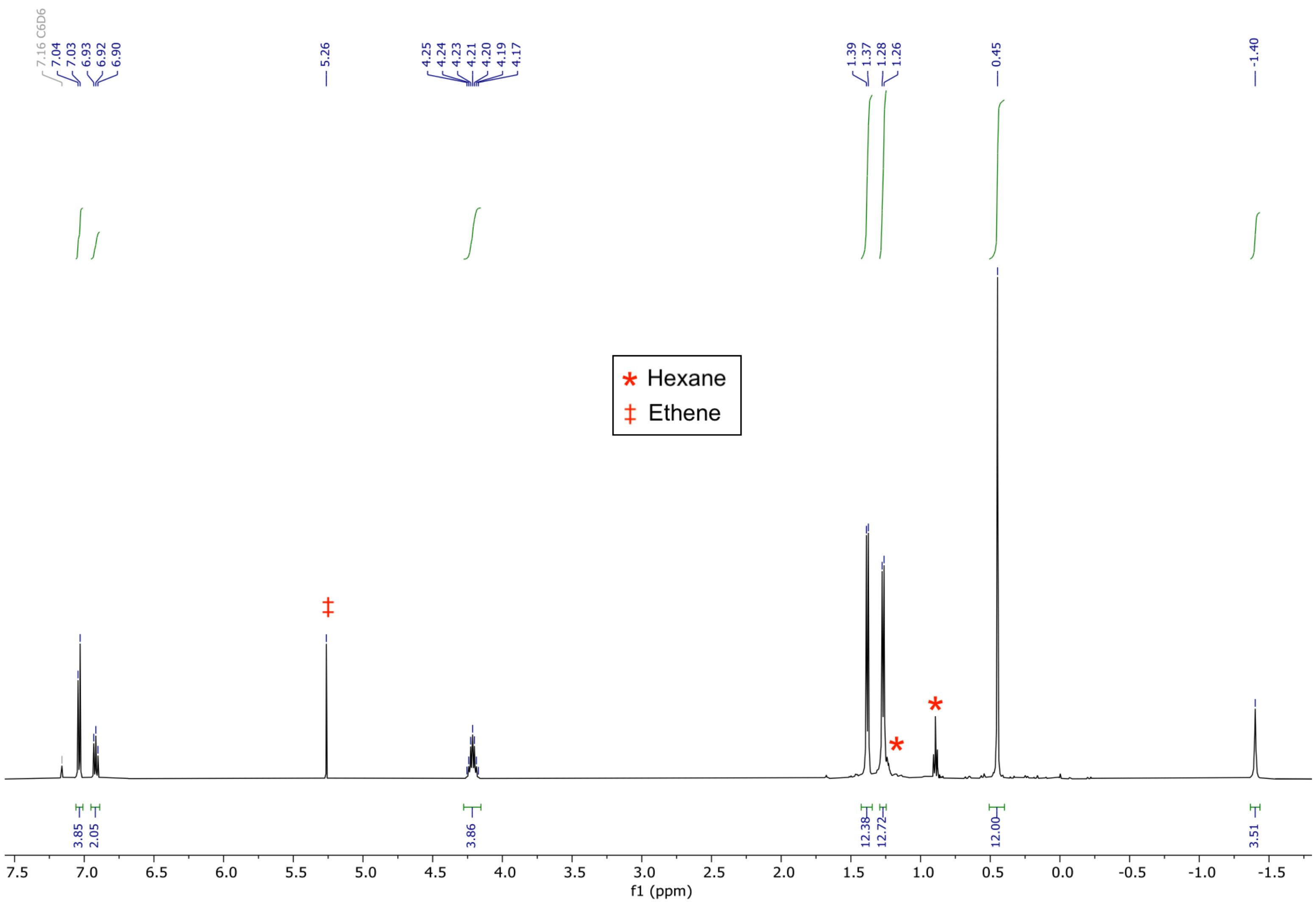

Figure S1b ^{1}H NMR spectrum (500 MHz, D_8-THF) of K[Al(NON^{Dipp})(C_2H_4)] (**1**) (*NMR spectrum recorded dried/desolvated crystals*)

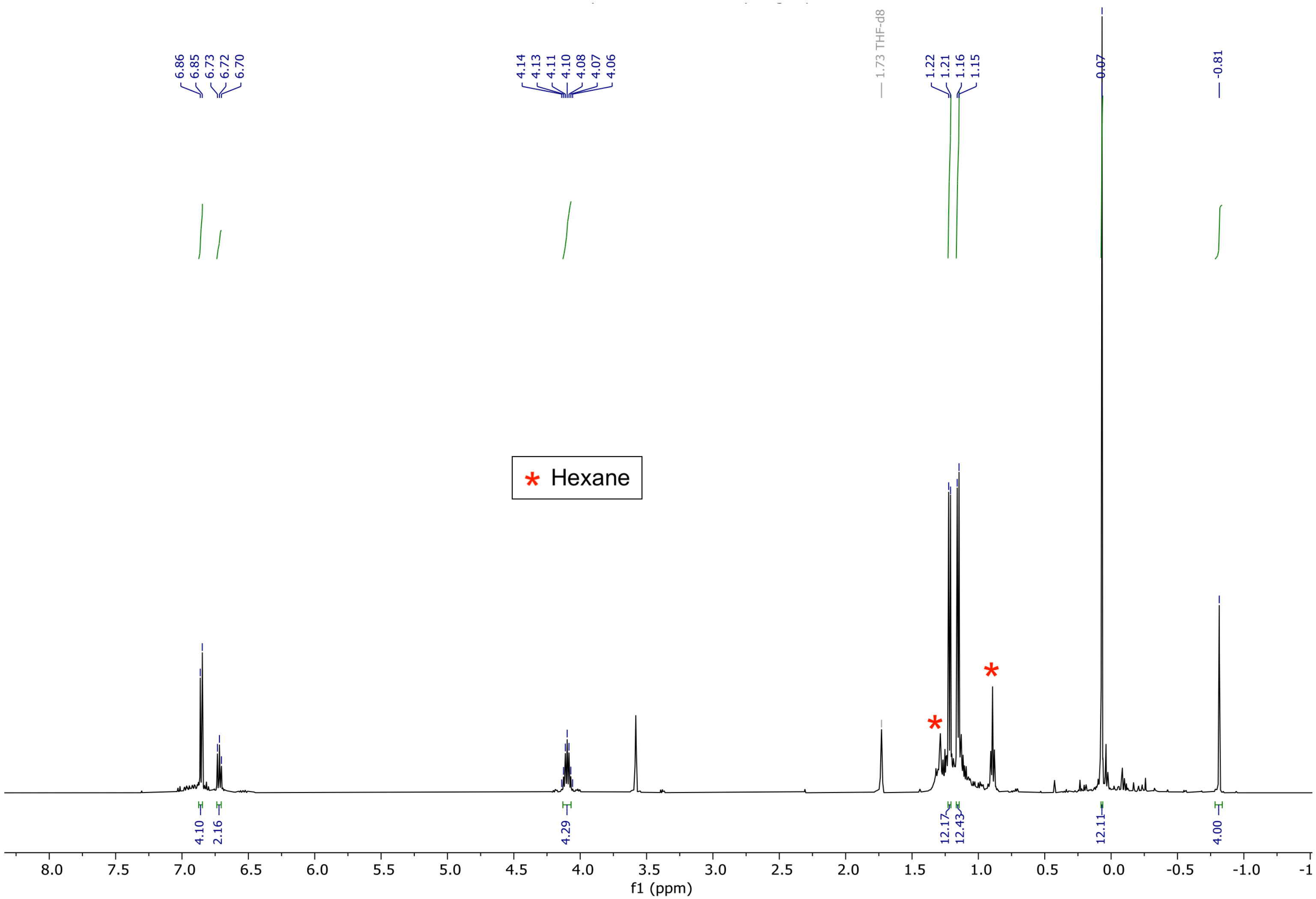

Figure S2 $^{13}C\{^1H\}$ NMR spectrum (126 MHz, C_6D_6) of $K[Al(NON^{Dipp})(C_2H_4)]$ (**1**) (*NMR spectrum recorded under C_2H_4 atmosphere*)

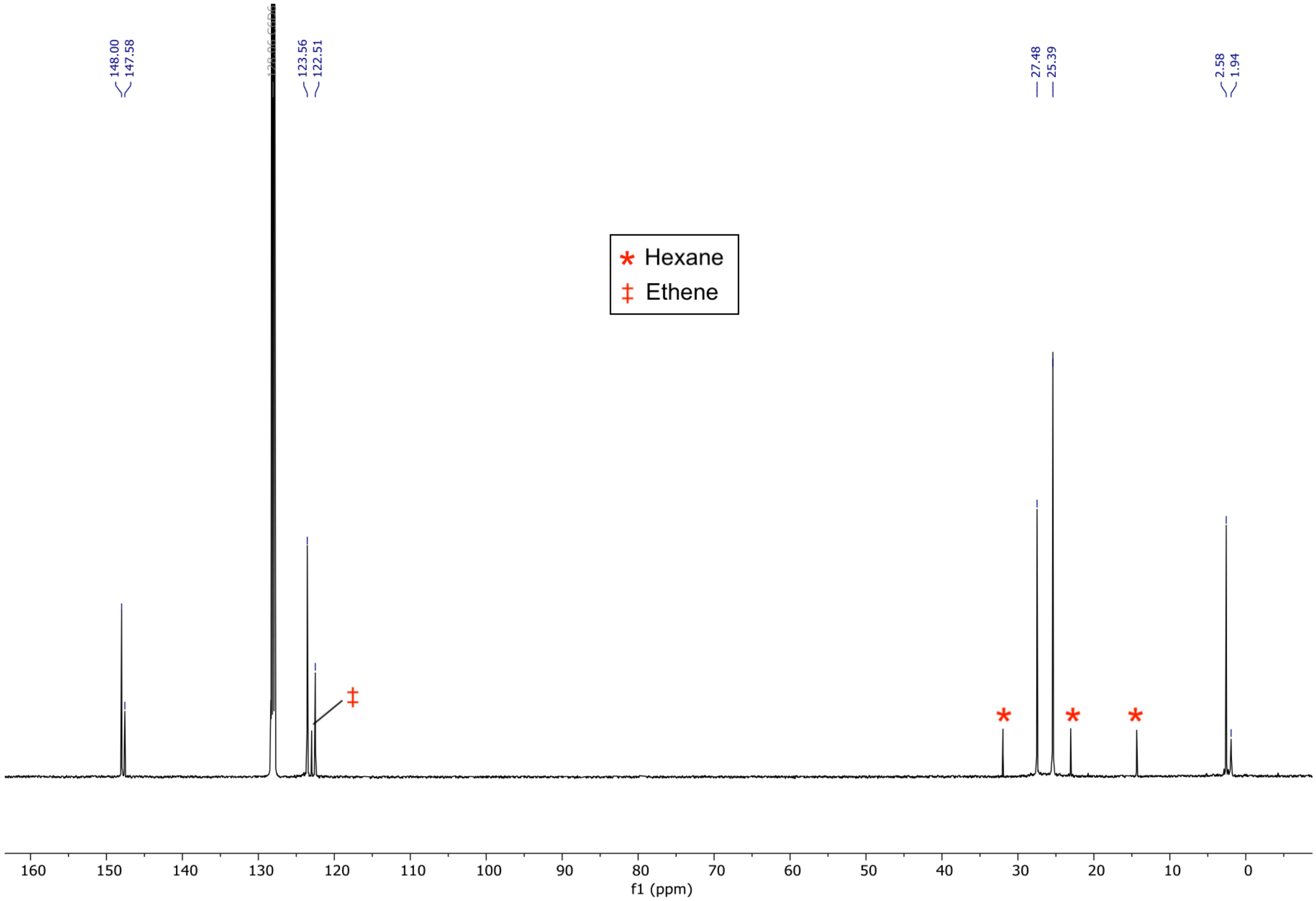

Figure S2b $^{13}C\{^{1}H\}$ NMR spectrum (500 MHz, D_8-THF) of K[Al(NON^{Dipp})(C_2H_4)] (**1**) (*NMR spectrum recorded dried/desolvated crystals*)

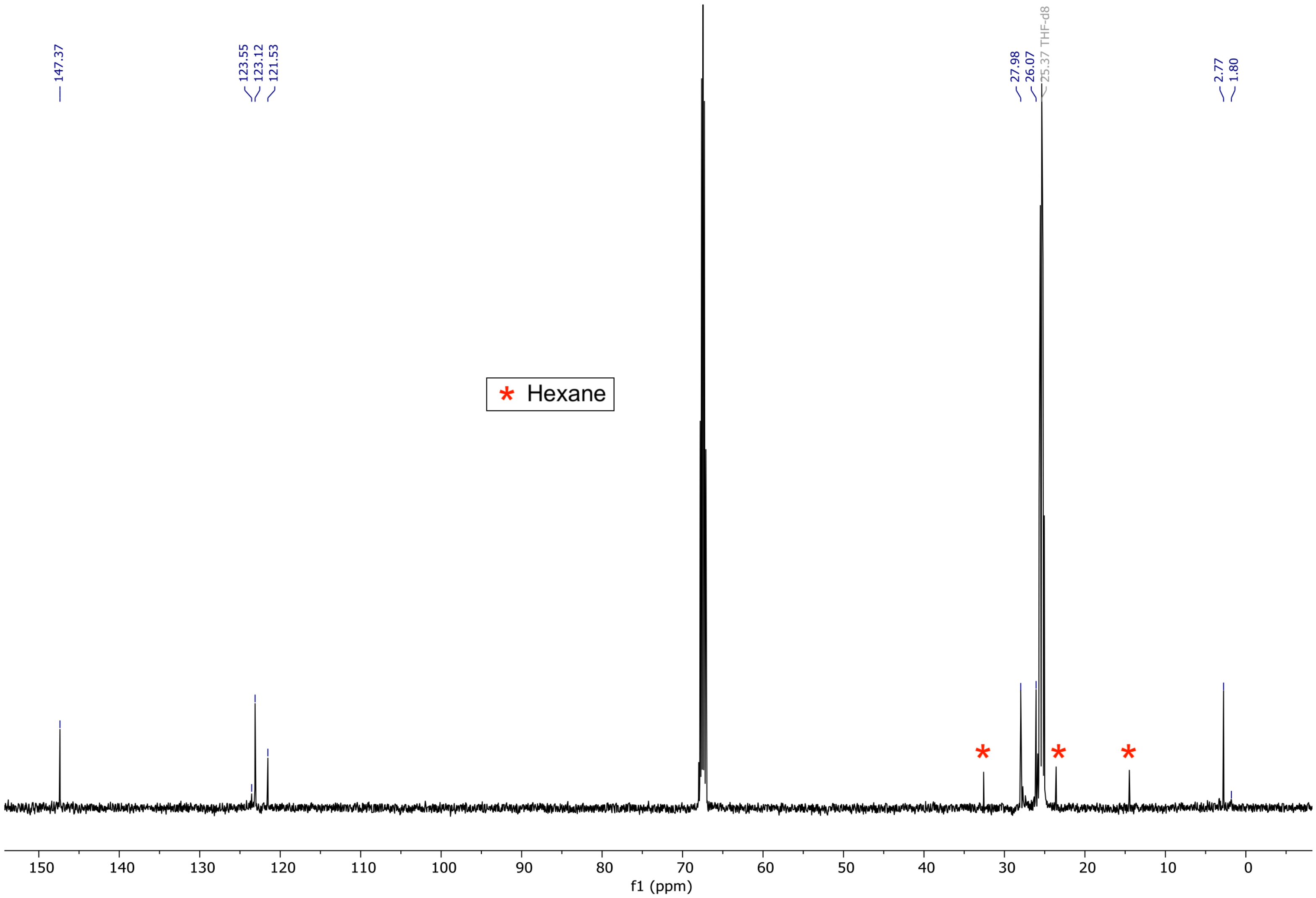

Figure S3 ^{1}H NMR spectrum (500 MHz, C_6D_6) of $[K_2(C_6H_6)_2][Al(NON^{Dipp})(C_2H_4)]_2{\cdot}C_6H_6$ (**$[1{\cdot}C_6H_6]_2{\cdot}C_6H_6$**) (*isolated crystals from C_6H_6*)

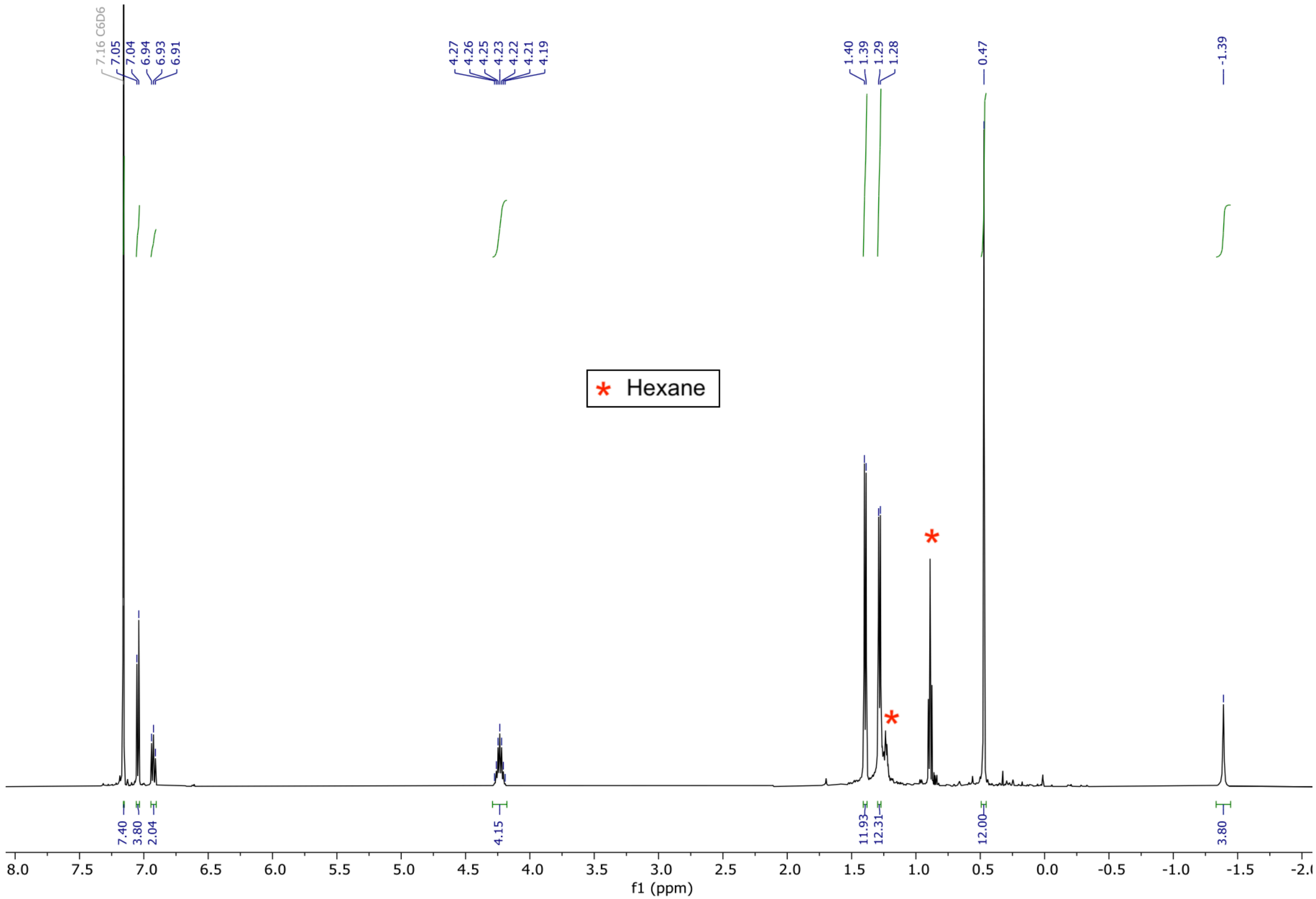

Figure S4 $^{13}C\{^1H\}$ NMR spectrum (126 MHz, C_6D_6) of $[K_2(C_6H_6)_2][Al(NON^{Dipp})(C_2H_4)]_2{\cdot}C_6H_6$ (**$[1{\cdot}C_6H_6]_2{\cdot}C_6H_6$**) (*isolated crystals from C_6H_6*)

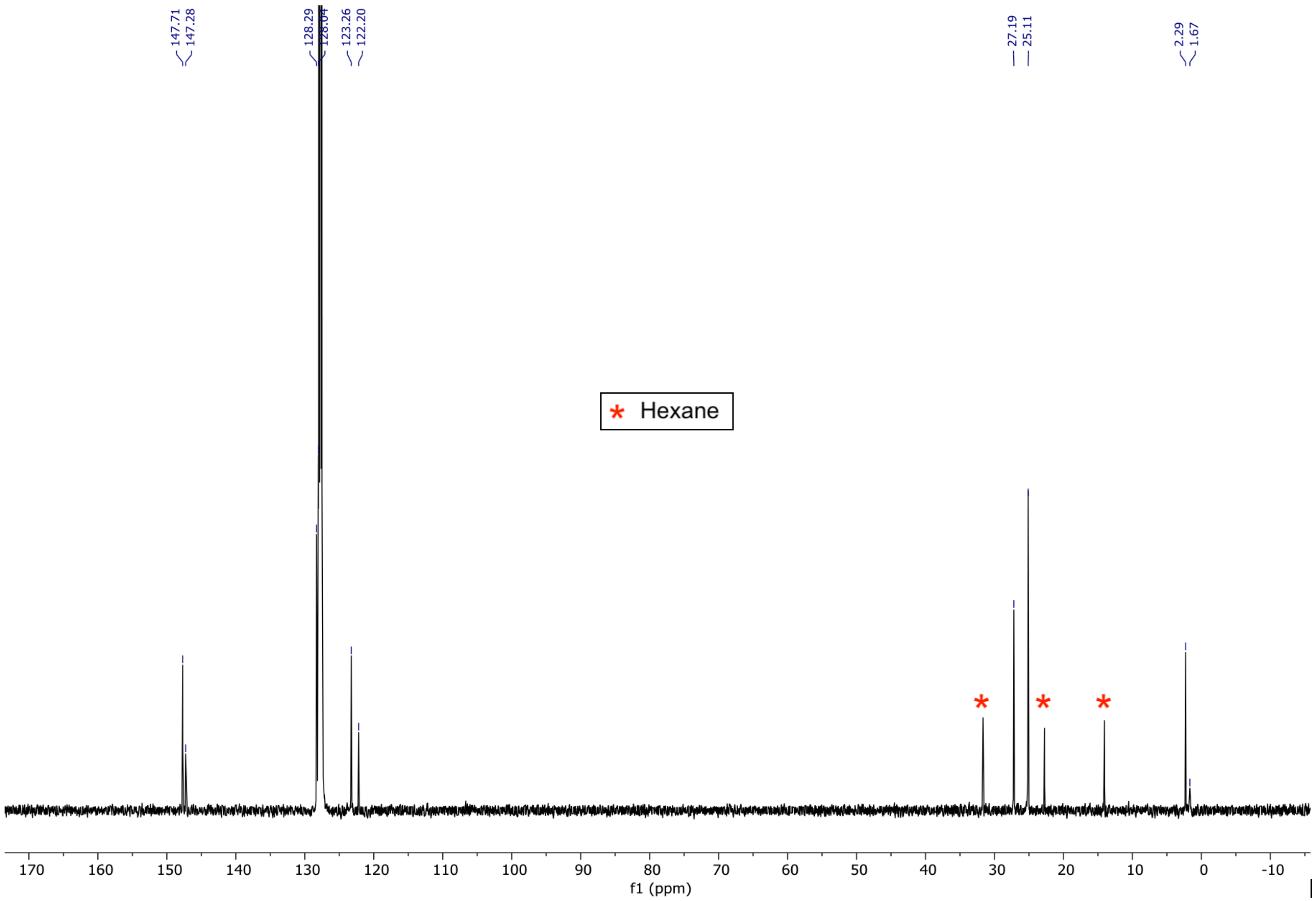

Figure S5 Variable temperature ^{1}H NMR spectrum (500 MHz, C_6D_6) of K[Al(NON^{Dipp})(C_2H_4)] (**1**)

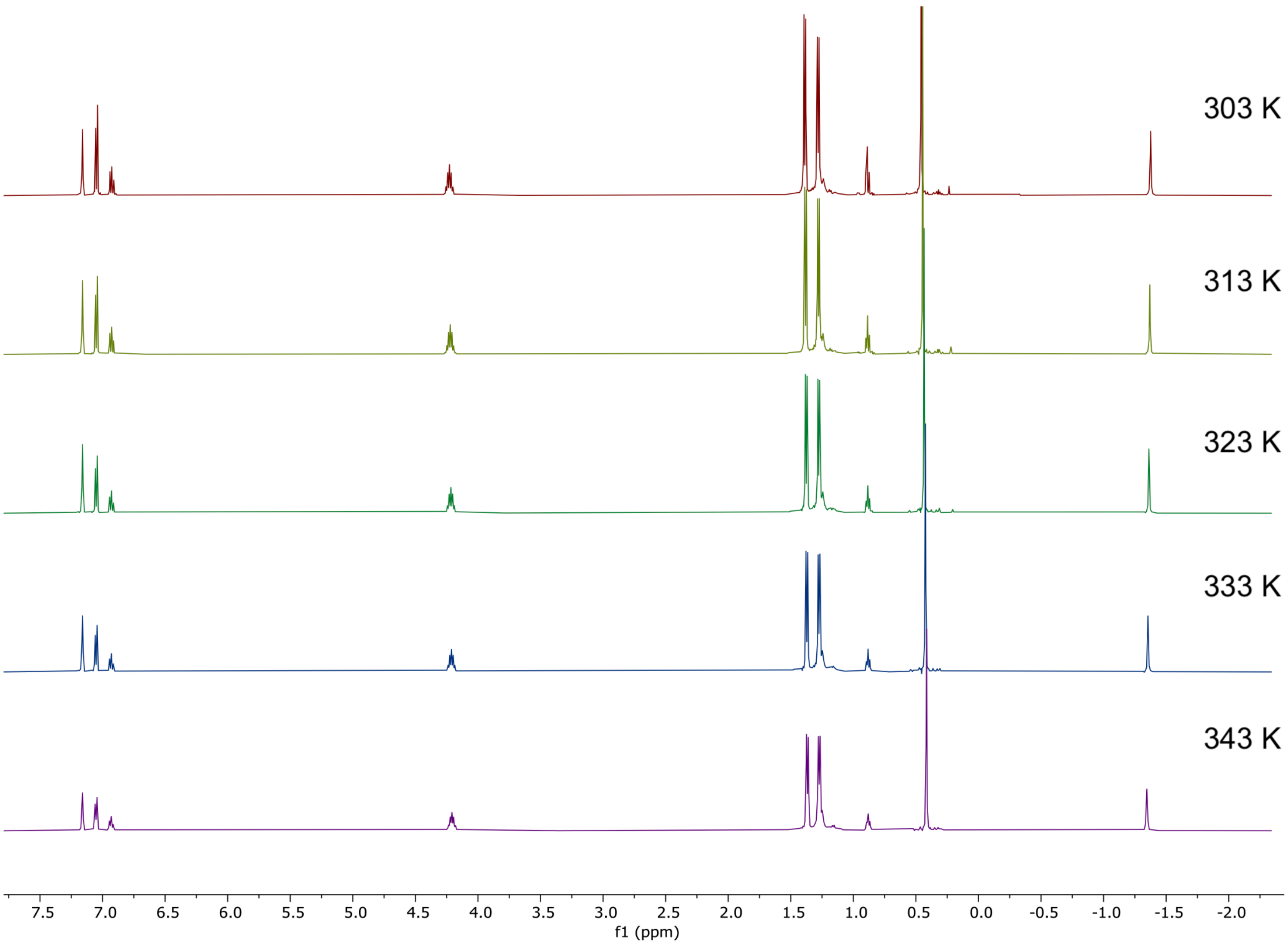

Figure S6 Displacement ellipsoid plot (30%, benzene solvate and H-atoms except C_2H_4 omitted) of the asymmetric unit of $[K_2(C_6H_6)_2][Al(NON^{Dipp})(C_2H_4)]_2{\cdot}C_6H_6$ (**$[1{\cdot}C_6H_6]_2{\cdot}C_6H_6$**)

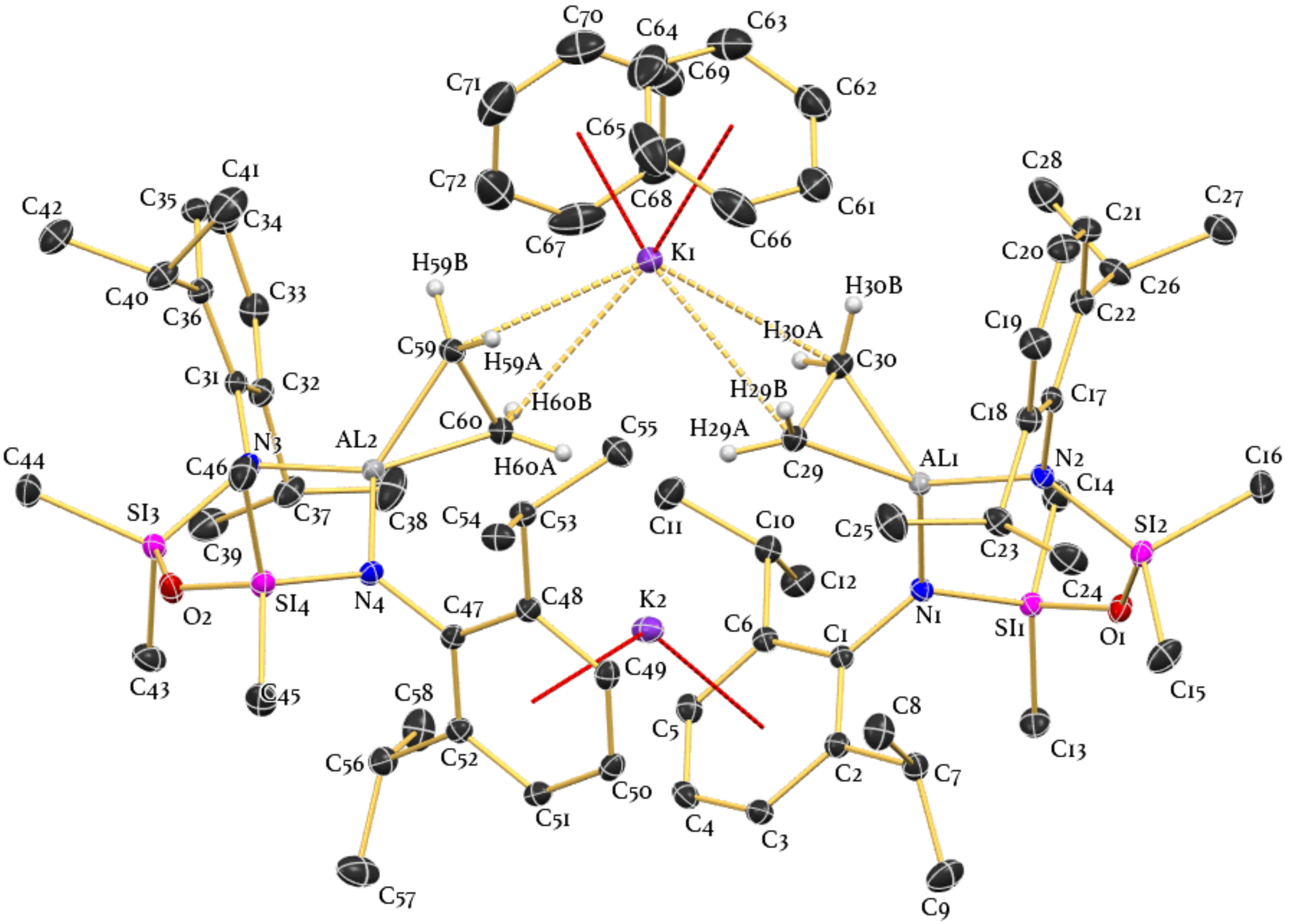

Synthesis of K_2[Al(NON^{Dipp})(μ-CH_2CH=CO)Al(NON^{Dipp})Et] (2)

A solution of [$K_2(C_6H_6)_2$][Al(NON^{Dipp})(C_2H_4)]$_2$ (142 mg, 0.10 mmol) in benzene was transferred to an ampule and sealed under nitrogen. The resulting solution was degassed, and carbon monoxide (~1 bar) added to the reaction vessel. The mixture was allowed to stir for 18 hours to give a dark orange solution. The solvent was removed *in vacuo* and residue dissolved in hexane (5 mL). Crystals were obtained at room temperature *via* slow evaporation from a hexane solution. Yield 28 mg, 23 %.

Anal. Calcd. for $C_{61}H_{100}Al_2K_2N_4O_3Si_4$ (*1181.97*): C, 61.99; H, 8.53; N, 4.74 %. Found: C, 61.42; H, 8.60; N, 4.52 %.

^{1}H NMR (500 MHz, C_6D_6): δ 6.90 (dd, *J* = 7.8, 1.8, 2H, C_6*H_3*), 6.83 (dd, *J* = 7.8, 1.8, 3H, C_6*H_3*), 6.80 (dd, *J* = 7.8, 1.8, 3H, C_6*H_3*), 6.68 (t, *J* = 7.8, 2H, C_6*H_3*), 6.64 (t, *J* = 7.8, 2H, C_6*H_3*), 6.22 (br t, *J* = 2.6, 1H, CH_2C*H*CO), 4.20 (sept, *J* = 6.8, 2H, C*H*Me_2), 4.15 (sept, *J* = 6.8, 4H, C*H*Me_2), 4.08 (sept, *J* = 6.8, 2H, C*H*Me_2), 1.58 (t, *J* = 7.8, 3H, CH_2C*H_3*), 1.31 (d, *J* = 6.8, 6H, CH*Me_2*), 1.27 (d, *J* = 5.0, 6H, CH*Me_2*), 1.25 (d, *J* = 5.0, 6H, CH*Me_2*), 1.15 (d, *J* = 6.8, 6H, CH*Me_2*), 1.14 (d, *J* = 6.8, 6H, CH*Me_2*), 1.03 (d, *J* = 6.8, 6H, CH*Me_2*), 0.56 (s, 6H, Si*Me_2*), 0.48 (d, *J* = 2.6, 2H, C*H_2*CHCO), 0.44 (s, 6H, Si*Me_2*), 0.34 (s, 6H, Si*Me_2*), 0.23 (s, 6H, Si*Me_2*), –0.17 (q, J = 7.8, 2H, C*H_2*CH_3).

^{13}C{^{1}H} NMR (126 MHz, C_6D_6): δ 184.5 (CH_2CH*C*O), 150.5, 149.91, 149.9, 149.5, 149.4, 148.0 (*C_6*H_3), 132.9 (CH_2*C*HCO), 124.9, 124.7, 124.0, 123.1, 122.3, 121.8 (*C_6*H_3), 27.9, 27.8 (CH*Me_2*), 27.6, 27.5, 27.3, 27.2 (*C*HMe_2), 26.4, 25.6, 25.1, 25.0, 23.7 (CH*Me_2*), 12.3 (CH_2*C*H_3) 11.0 (*C*H_2CHCO), 7.3 (*C*H_2CH_3), 5.2, 3.7, 3.3, 3.1 (Si*Me_2*).

Figure S7 ^{1}H NMR spectrum (500 MHz, C_6D_6) of $K_2[Al(NON^{Dipp})(\mu\text{-}CH_2CH{=}CO)Al(NON^{Dipp})Et]$ (**2**)

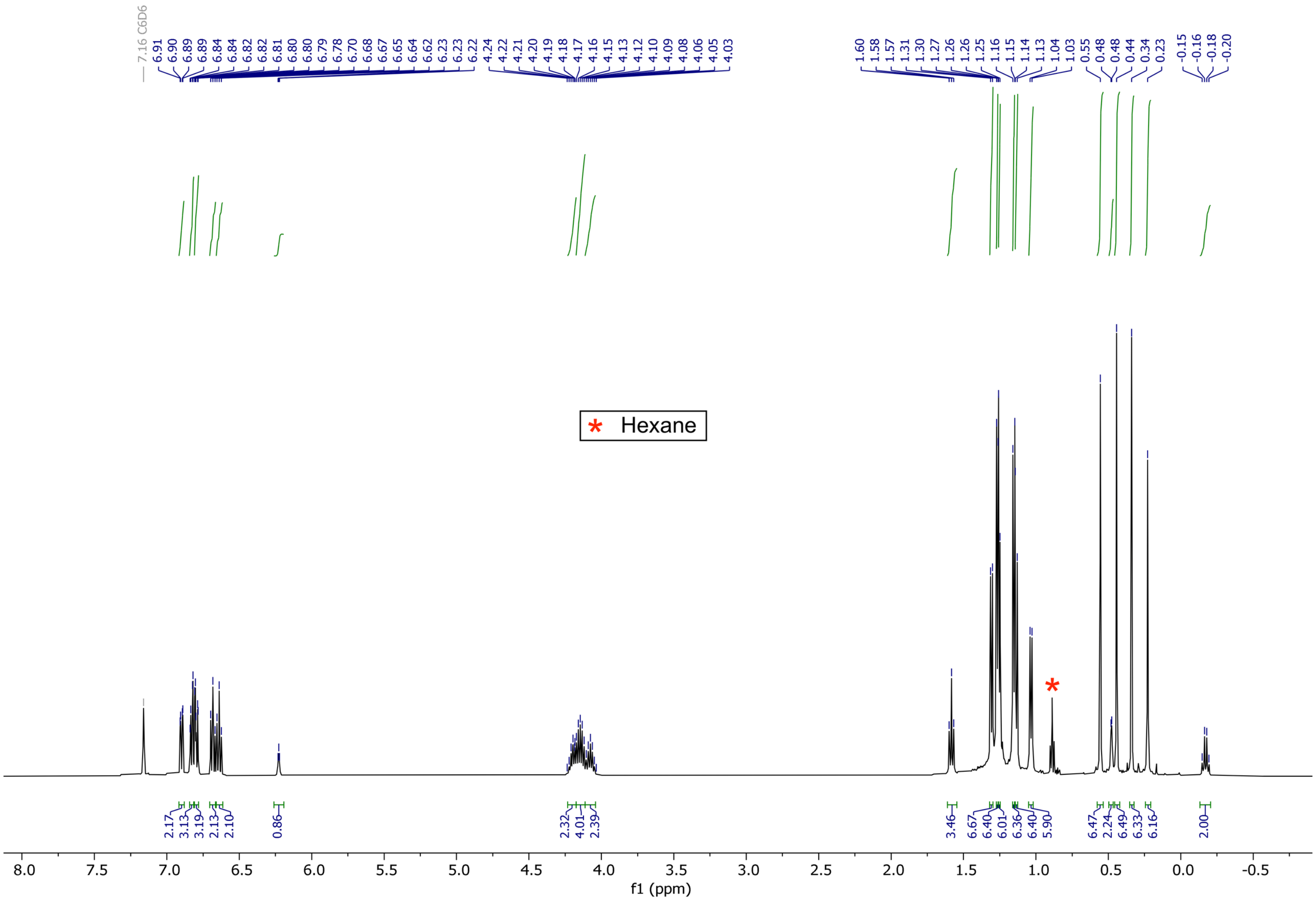

Figure S8 $^{13}C\{^{1}H\}$ NMR spectrum (126 MHz, C_6D_6) of $K_2[Al(NON^{Dipp})(\mu\text{-}CH_2CH{=}CO)Al(NON^{Dipp})Et]$ (**2**)

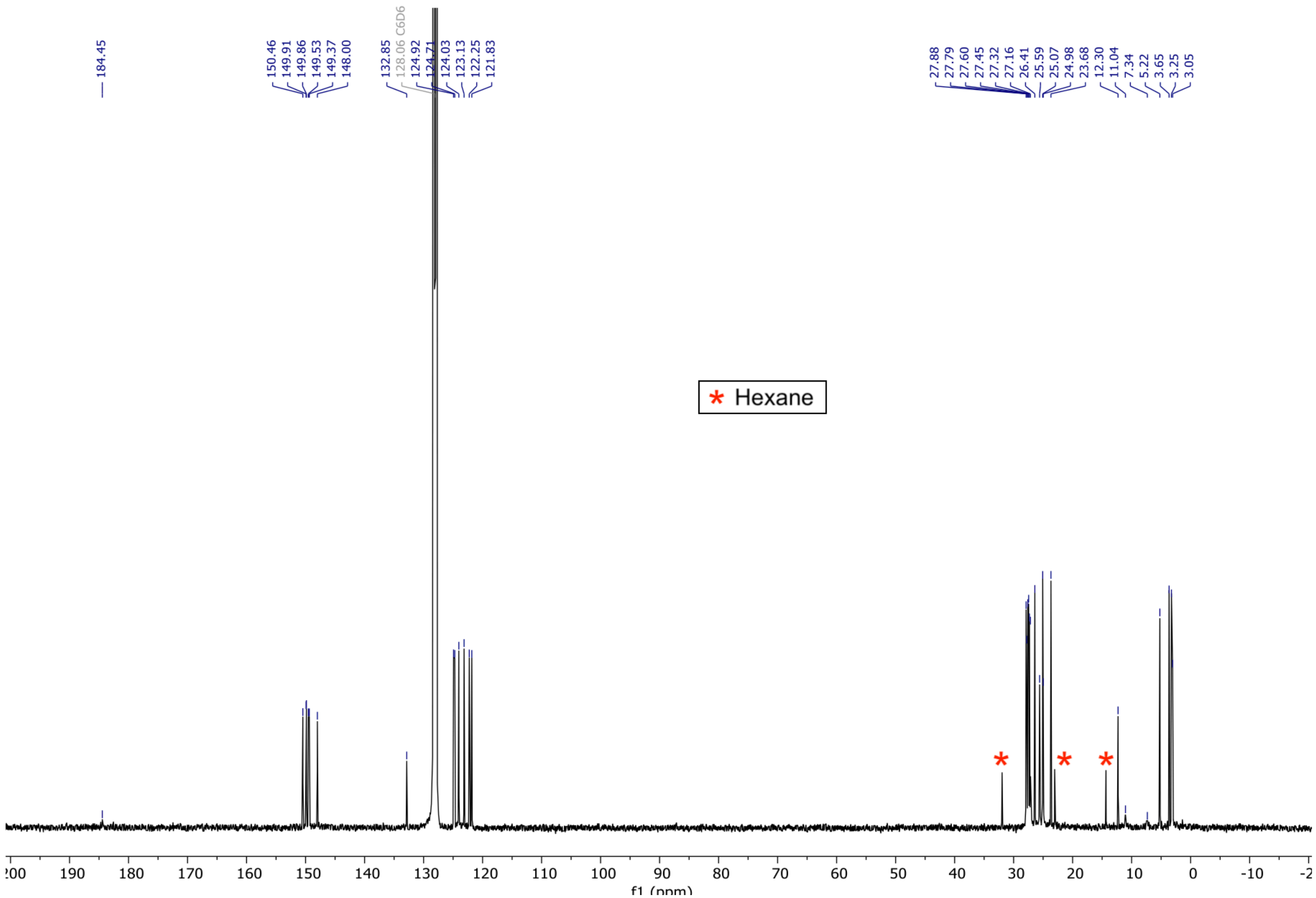

Figure S8b HSQC NMR spectrum $K_2[Al(NON^{Dipp})(\mu\text{-}CH_2CH{=}CO)Al(NON^{Dipp})Et]$ (**2**)

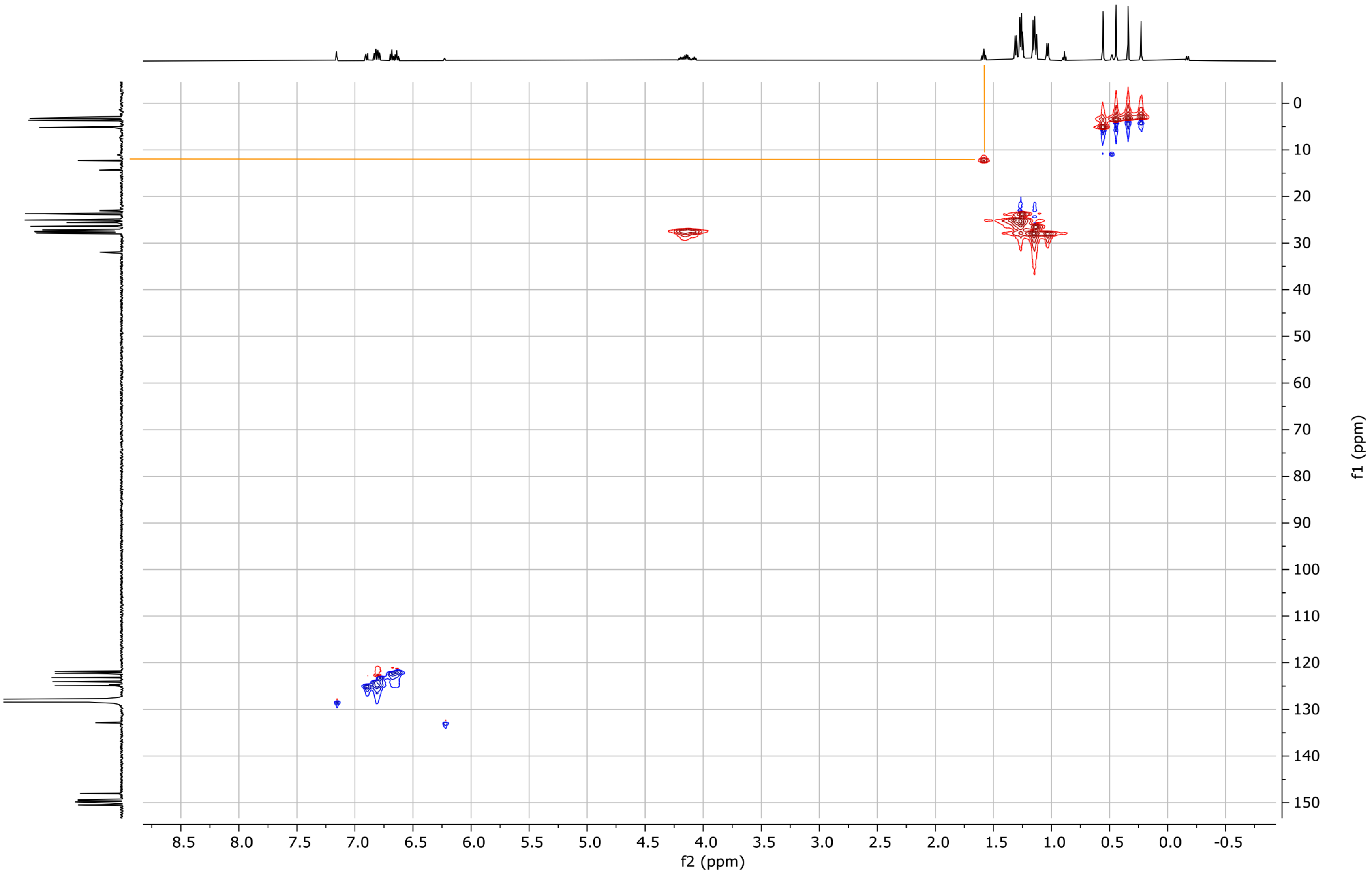

Figure S8c HMBC NMR spectrum $K_2[Al(NON^{Dipp})(\mu\text{-}CH_2CH{=}CO)Al(NON^{Dipp})Et]$ (**2**)

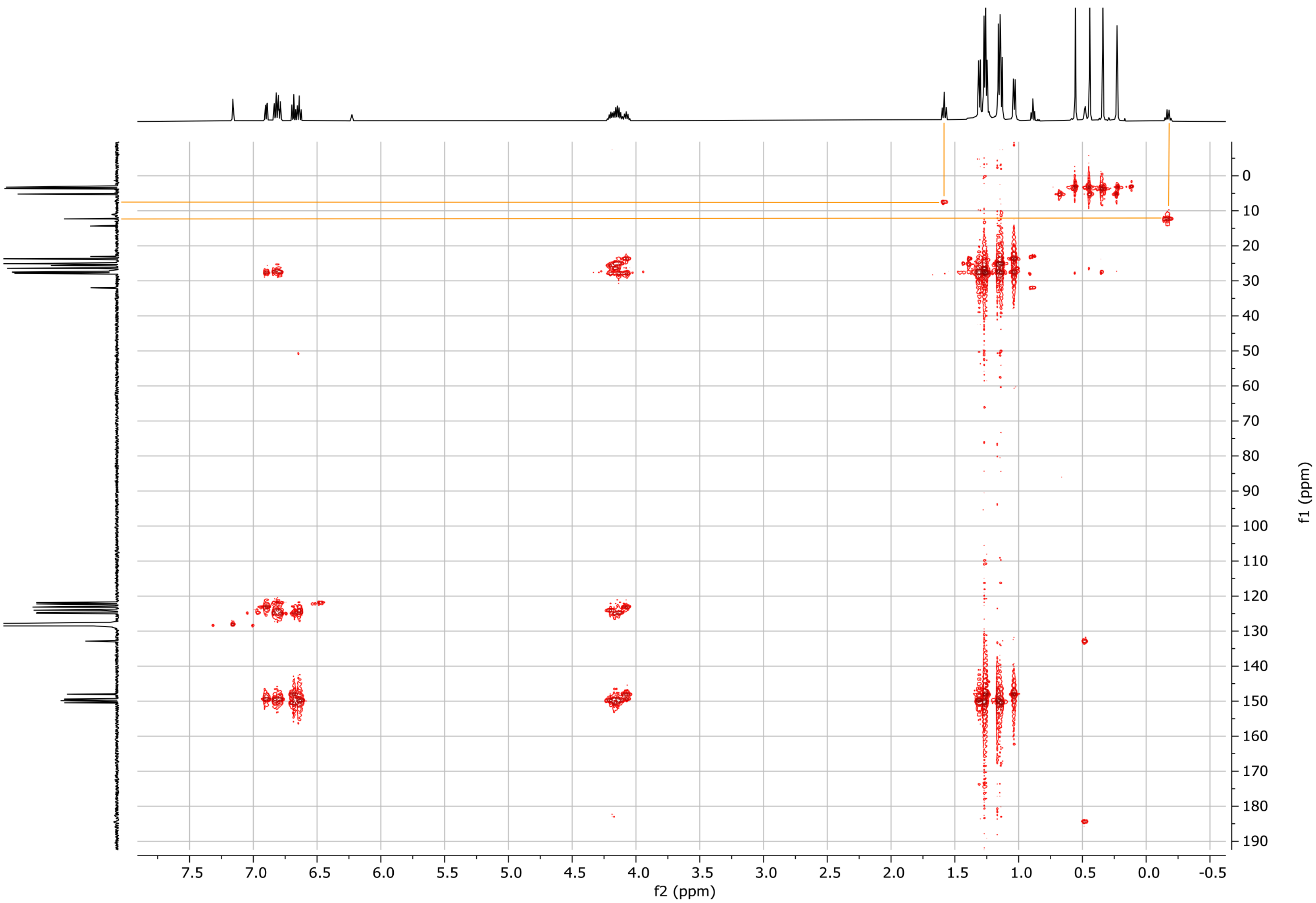

Figure S9 Displacement ellipsoid plot (30%, H-atoms omitted) of the asymmetric unit of $K_2[Al(NON^{Dipp})(\mu\text{-}CH_2CH{=}CO)Al(NON^{Dipp})Et]$ (**2**). Note: this molecule lies on a 2-fold rotation access, with both the Al*Et* and Al(*$CH_2CH{=}O$*) ligands co-existing in the asymmetric unit.

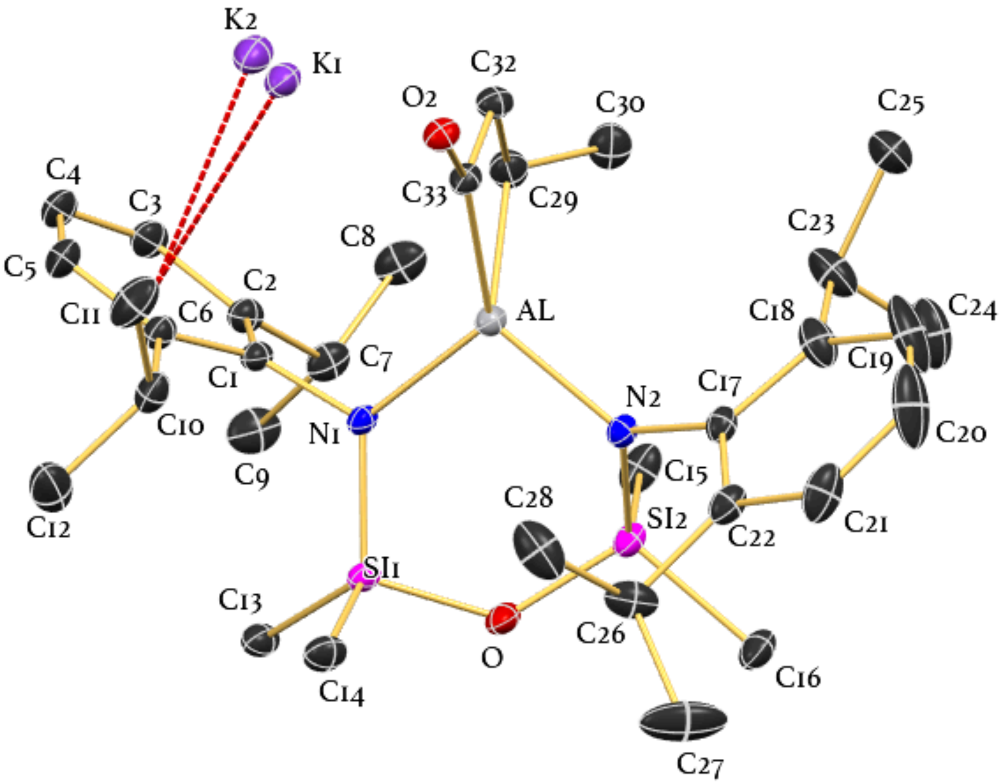

Synthesis of [K(18-crown-6)][Al(NON^{Dipp})(C_2H_4)] (3)

18-crown-6 (45 mg, 0.17 mmol) was added to a solution of [$K_2(C_6H_6)_2$][Al(NON^{Dipp})(C_2H_4)]$_2$ (113 mg, 0.17 mmol) in THF. The solvent was reduced *in vacuo* (*ca.* 2 mL) and allowed to crystallise at room temperature *via* slow evaporation. Yield 152 mg, 96 %.

^{1}H NMR (500 MHz, THF-D$_8$): δ 6.85 (d, J = 7.6, 4H, C_6H_3), 6.70 (t, J = 7.6, 2H, C_6H_3), 4.13 (sept, J = 6.8, 4H, C*H*Me$_2$), 3.48 (s, 24H, crown-C*H*$_2$), 1.25 (d, J = 6.8, 12H, CH*Me*$_2$), 1.15 (d, J = 6.8, 12H, CH*Me*$_2$), 0.06 (s, 12H, Si*Me*$_2$), –0.69 (s, 4H, =C*H*$_2$).

^{13}C{^{1}H} NMR (126 MHz, THF-D$_8$): δ 147.7, 147.4, 122.9, 121.2 (*C*$_6$H$_3$), 71.1 (crown-*C*H$_2$), 27.9 (*C*HMe$_2$), 26.1, 25.8 (CH*Me*$_2$), 3.1 (*C*H$_2$), 2.8 (Si*Me*$_2$).

Figure S10 ^1H NMR spectrum (500 MHz, THF-D_8) of [K(18-crown-6)][Al(NON^{Dipp})(C_2H_4)] (**3**)

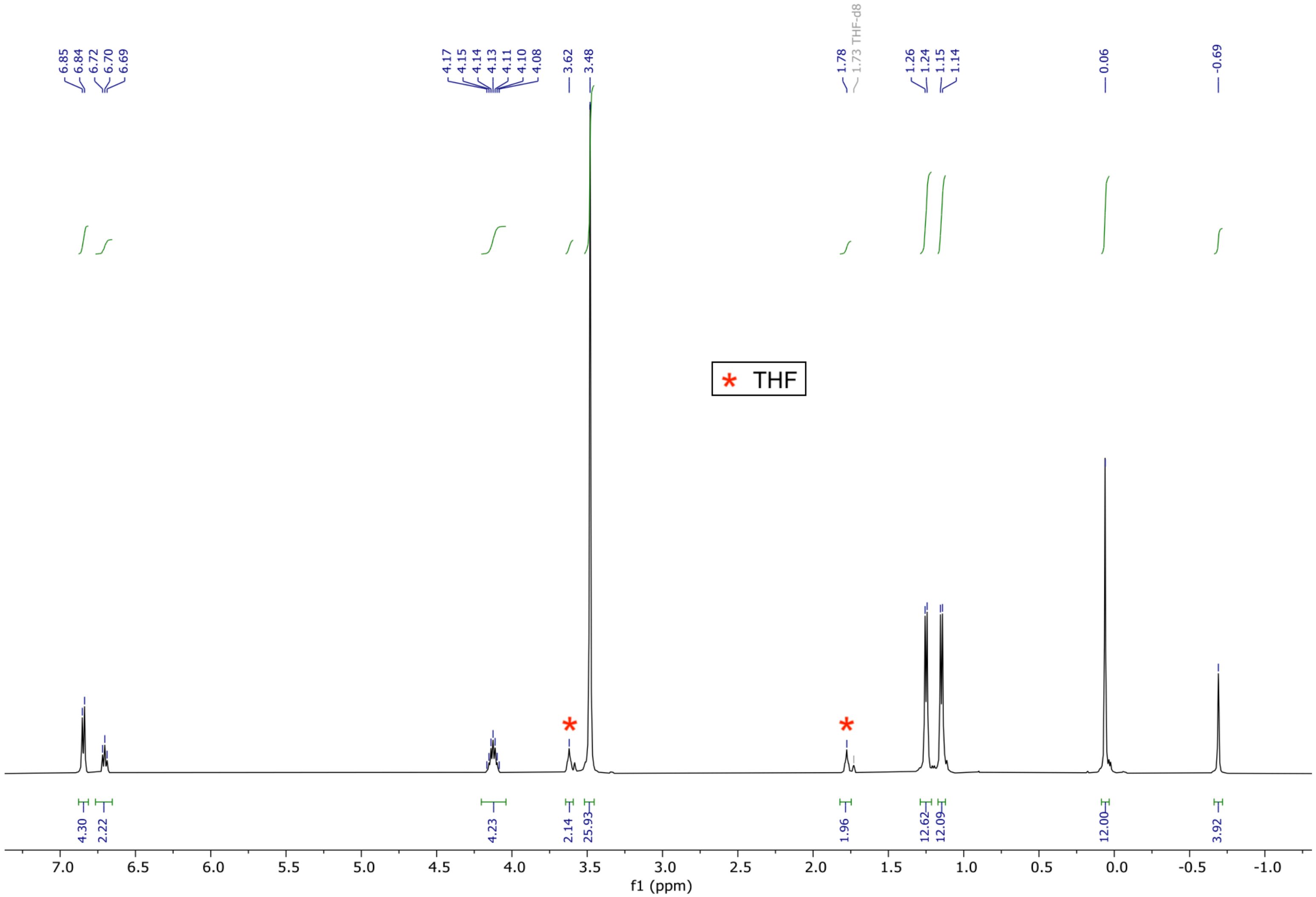

Figure S11 $^{13}C\{^{1}H\}$ NMR spectrum (126 MHz, THF-D_8) of [K(18-crown-6)][Al(NON^{Dipp})(C_2H_4)] (**3**)

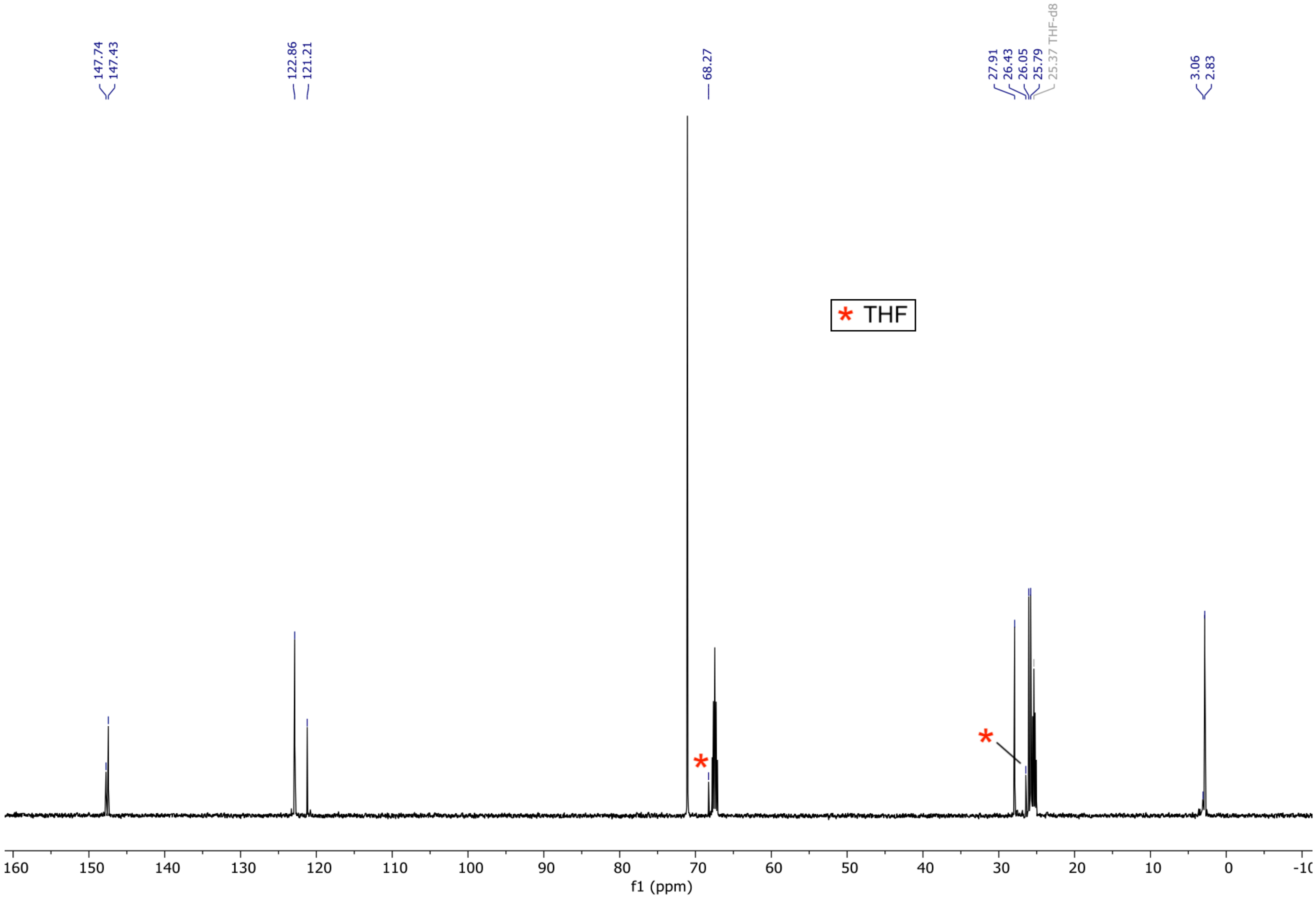

Figure S12 Displacement ellipsoid plot (30%, H-atoms except C_2H_4 omitted) of the asymmetric unit of [K(18-crown-6)][Al(NON^{Dipp})(C_2H_4)] (**3**)

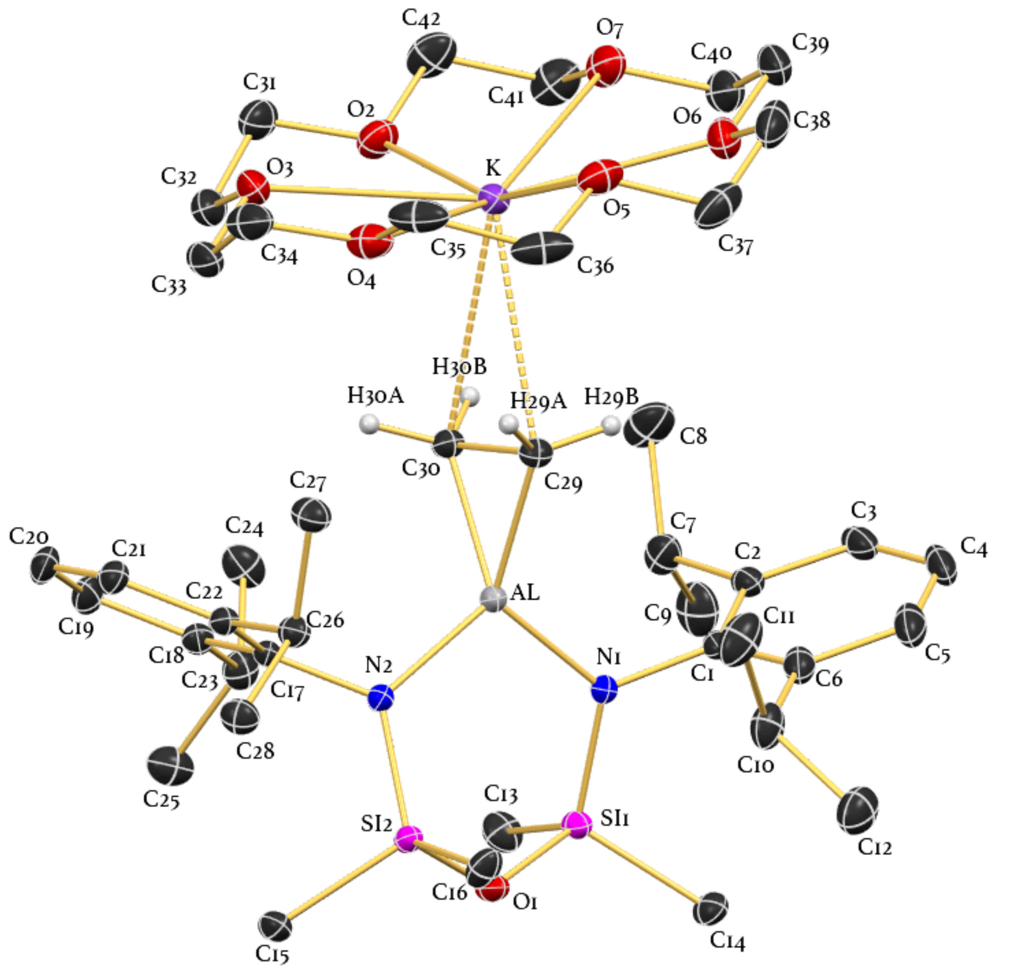

Synthesis of [K(18-crown-6)(THF)][Al(NON^{Dipp})($CH_2CH_2C{=}O$)] (4)

A solution of [K(18-crown-6)][Al(NON^{Dipp})(C_2H_4)] (152 mg, 0.17 mmol) in THF was transferred to an ampule and sealed under nitrogen. The resulting solution was degassed and carbon monoxide (~1 bar) added to the reaction vessel. The mixture was allowed to stir for 1 hour to give a bright yellow solution. The solvent was reduced *in vacuo* and a few drops of hexane added. Crystals of **4·THF** were obtained from a solution stored at –30 °C for *ca.* 4 hrs. Yield 41 mg, 26 %.

^{1}H NMR (500 MHz, THF-D_8): δ 6.87 (d, J = 7.6, 2H, C_6H_3), 6.82 (d, J = 7.6, 2H, C_6H_3), 6.70 (t, J = 7.6, 2H, C_6H_3), 4.16 (sept, J = 6.8, 2H, C*H*Me$_2$), 3.86 (sept, J = 6.8, 2H, C*H*Me$_2$), 3.62 (s, 2H‡, THF), 1.78 (s, 2H‡, THF), 1.24 (d, J = 6.8, 6H, CH*Me$_2$*), 1.15 (d, J = 6.8, 6H, CH*Me$_2$*), 1.12 (d, J = 6.8, 6H, CH*Me$_2$*), 1.09 (d, J = 6.8, 6H, CH*Me$_2$*), 1.06* (t, J = 9.6, 2H, CH_2CH_2CO), 0.09 (s, 6H, Si*Me$_2$*), 0.04 (s, 6H, Si*Me$_2$*), –0.24 (t, J = 9.6, 2H, CH_2CH_2O).

^{13}C NMR (126 MHz, THF-D_8) δ 147.5, 147.4, 147.2, 123.3, 122.8, 121.7 (C_6H_3), 71.1 (crown-CH_2), 68.3 (THF), 53.0 (CH_2CH_2CO), 27.9, 27.6 (*C*HMe$_2$), 26.4, 26.4 (CH*Me$_2$*), 26.1 (THF), 25.9, 25.1 (CH*Me$_2$*), 3.5 (CH_2CH_2CHO), 3.0, 2.9 (Si*Me$_2$*).

(^{13}C{^{1}H} NMR peaks for CH_2CH_2*C*O not observed).

‡ integral reflects partial loss of THF from sample during drying of the crystals prior to making the NMR sample

* Overlapping with CH*Me$_2$* signals.

Figure S13 ^{1}H NMR spectrum (500 MHz, THF-D$_8$) of [K(18-crown-6)(THF)][Al(NON^{Dipp})(CH$_2$CH$_2$C=O)]·THF (**4·THF**)

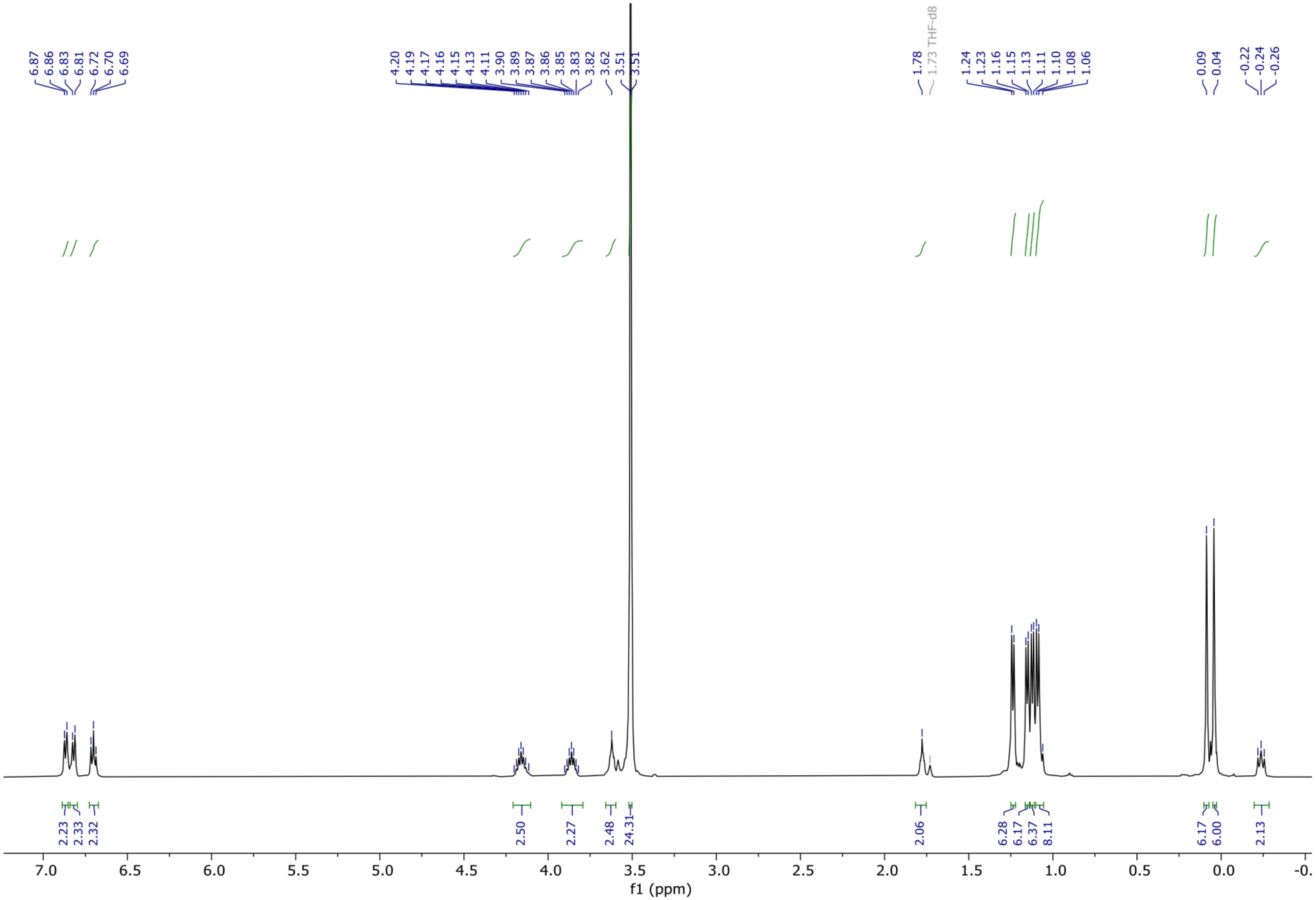

Figure S14 $^{13}C\{^{1}H\}$ NMR spectrum (126 MHz, THF-D_8) of [K(18-crown-6)(THF)][Al(NON^{Dipp})($CH_2CH_2C{=}O$)]·THF (**4·THF**)

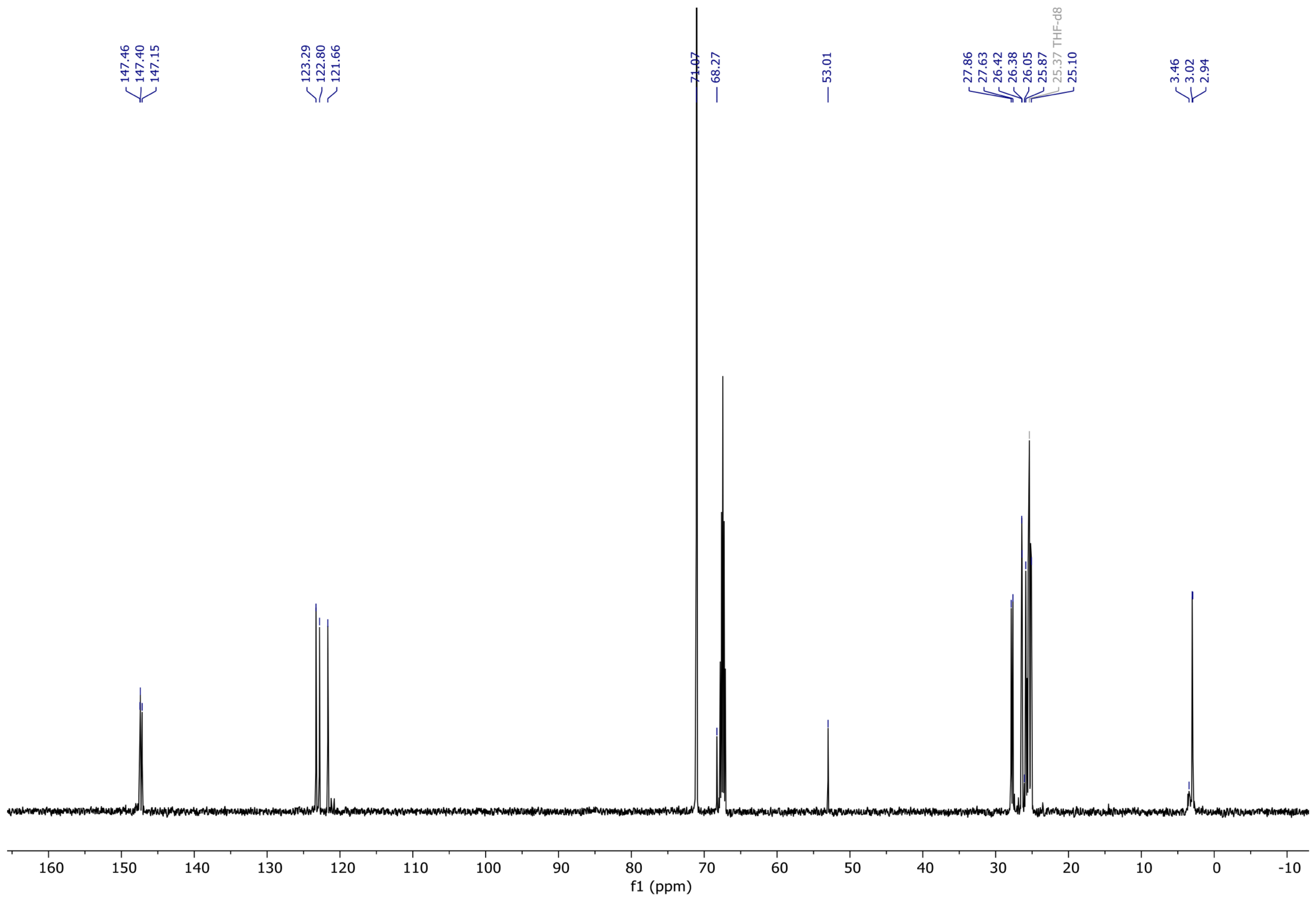

Figure S15 Displacement ellipsoid plot (30%, THF solvate, disordered atoms and H-atoms except C*H*$_2$C*H*$_2$C=O omitted) of the asymmetric unit of [K(18-crown-6)(THF)][Al(NON^{Dipp})(CH$_2$CH$_2$C=O)]·THF (**4·THF**)

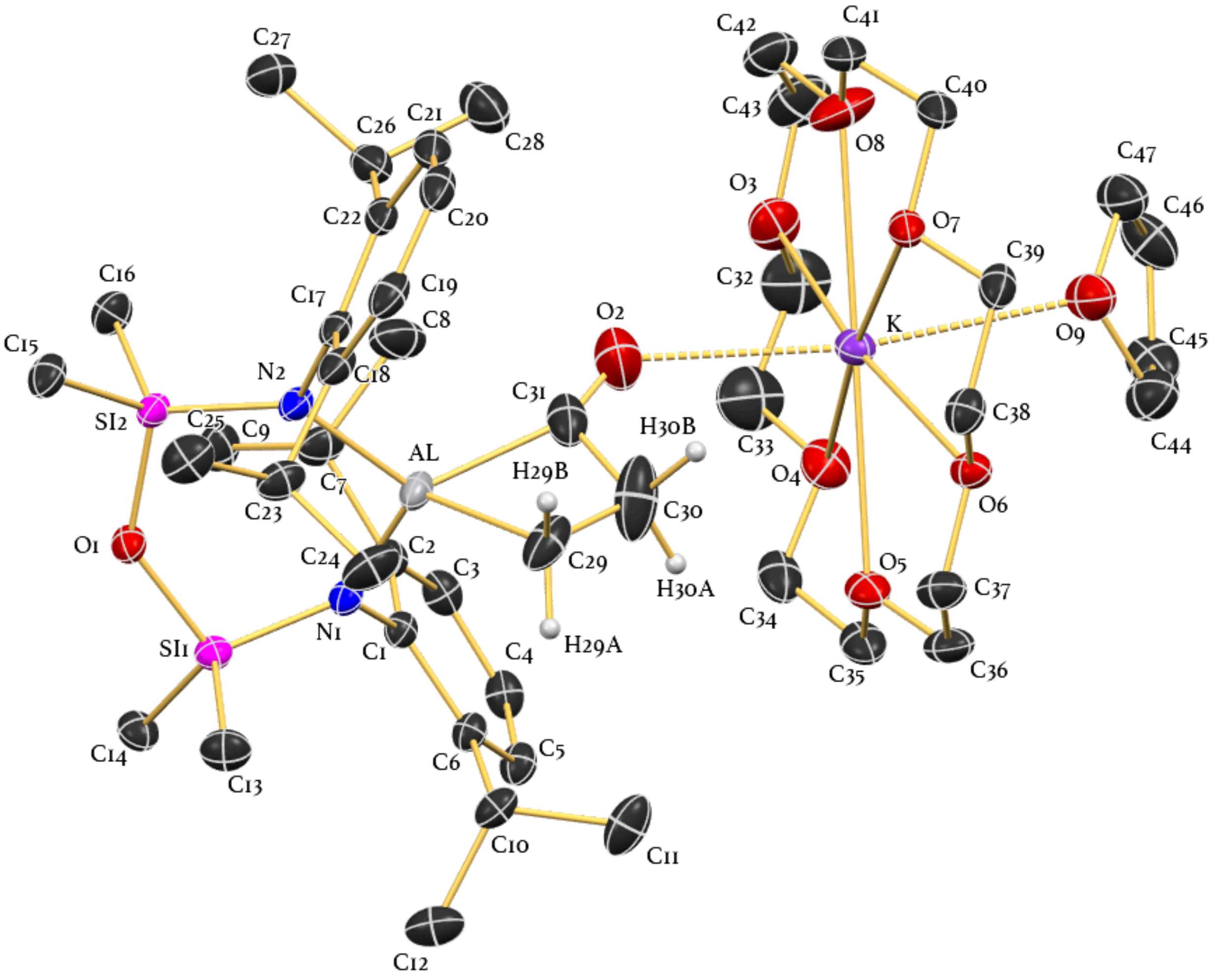

Synthesis of [K(18-crown-6)(THF)$_2$][Al(NON^{Dipp})(CH$_2$CH=CHO)] (5)

A solution of [K(18-crown-6)(THF)][Al(NON^{Dipp})(CH$_2$CH$_2$CO)] (120 mg, 0.14 mmol) in THF-D$_8$ was transferred to J Youngs NMR tube and sealed under nitrogen. The resulting solution was degassed and carbon monoxide (~1 bar) added to the reaction vessel. The mixture was allowed to stir for 1 hour to give a bright yellow solution. This solution was degassed and placed under an atmosphere of nitrogen. The solution was then heated for *ca.* 5 days at 80 °C. The solvent was removed *in vacuo* and the oily residue dissolved in a pentane/THF mixture (1:10). Slow evaporation of the solvent at room temperature yielded crystals of [K(18-crown-6)(THF)$_2$][Al(NON^{Dipp})(CH$_2$CH=CHO)]·THF (**5·THF**). Yield 114 mg, 82 %.

Anal. Calcd. for $C_{51}H_{90}AlKN_2O_{10}Si_2$ (*1013.52*): C, 60.44; H, 8.95; N, 2.76 %. Found: C, 59.82; H, 8.61; N, 2.96 %.

^{1}H NMR (500 MHz, THF-D$_8$): δ 6.81 – 6.75* (m, 4H, C$_6$*H$_3$*), 6.65 (t, *J* = 7.6, 2H, C$_6$*H$_3$*), 6.00 (dt, *J* = 4.0, 2.0, 1H, CH$_2$CH=C*H*O), 4.12 (sept, *J* = 6.8, 2H, C*H*Me$_2$), 4.06 (sept, *J* = 6.8, 2H, C*H*Me$_2$), 4.01 (q, *J* = 3.4 Hz, 1H, CH$_2$C*H*=CHO), 3.59 (s, 2H‡, THF), 1.74 (s, 2H‡, THF), 1.19 (d, *J* = 6.8, 6H, CH*Me$_2$*), 1.11* (d, *J* = 6.8, 6H, CH*Me$_2$*), 1.11* (d, *J* = 6.8, 6H, CH*Me$_2$*), 1.08 (d, *J* = 6.8, 6H, CH*Me$_2$*), –0.01 (s, 12H, Si*Me$_2$*), –0.44 (dd, J = 3.4, 2.0, 2H, C*H$_2$*CH=CHO).

^{13}C{^{1}H} NMR (126 MHz, THF-D$_8$): δ 152.3 (CH$_2$CH=*C*HO), 148.3, 148.2, 147.2, 123.3, 122.7, 121.3 (*C$_6$*H$_3$), 103.0 (CH$_2$*C*H=CHO), 71.3 (crown-*C*H$_2$), 68.4 (THF), 27.9, 27.9 (*C*HMe$_2$), 26.5 (THF), 26.4, 26.4, 26.1, 26.0 (CH*Me$_2$*), 3.2, 3.2 (Si*Me$_2$*).

(^{13}C{^{1}H} NMR peaks for Al*C*H$_2$CH=CHO not observed).

‡ integral reflects partial loss of THF from sample during drying of the crystals prior to making the NMR sample

* Overlapping signals.

Figure S16 ^{1}H NMR spectrum (500 MHz, THF-D_8) of [K(18-crown-6)(THF)$_2$][Al(NON^{Dipp})($CH_2CH=CHO$)]·THF (**5·THF**)

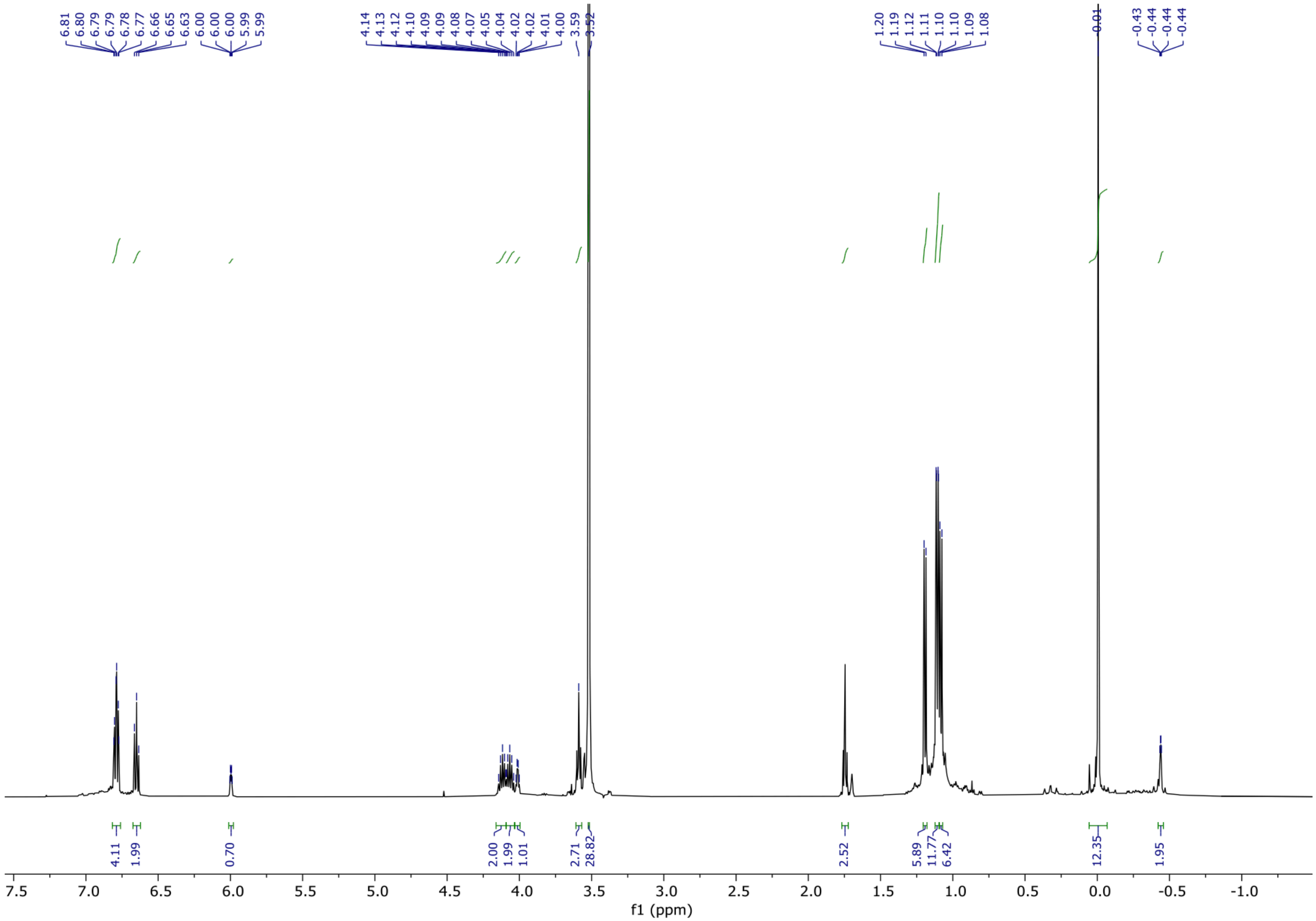

Figure S17 $^{13}C\{^{1}H\}$ NMR spectrum (126 MHz, THF-D_8) of [K(18-crown-6)(THF)$_2$][Al(NON^{Dipp})($CH_2CH{=}CHO$)]·THF (**5·THF**)

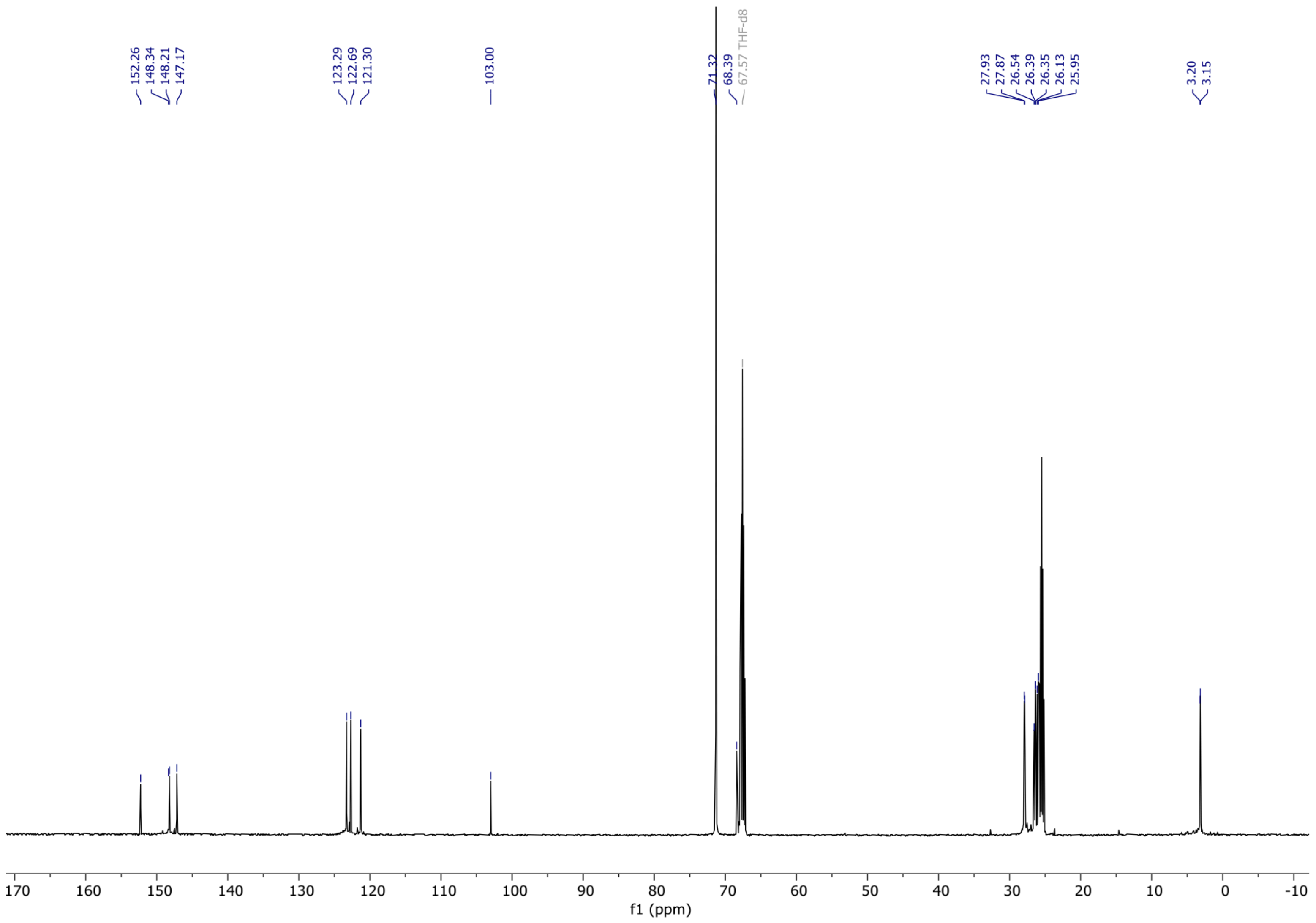

Figure S18 Displacement ellipsoid plot (30%, THF solvate, disordered atoms and H-atoms except CH2CH=CHO omitted) of the asymmetric unit of [K(18-crown-6)(THF)2][Al(NONDipp)(CH2CH=CHO)]·THF (**5·THF**) (*O10 and C48-C51 refined isotropically*)

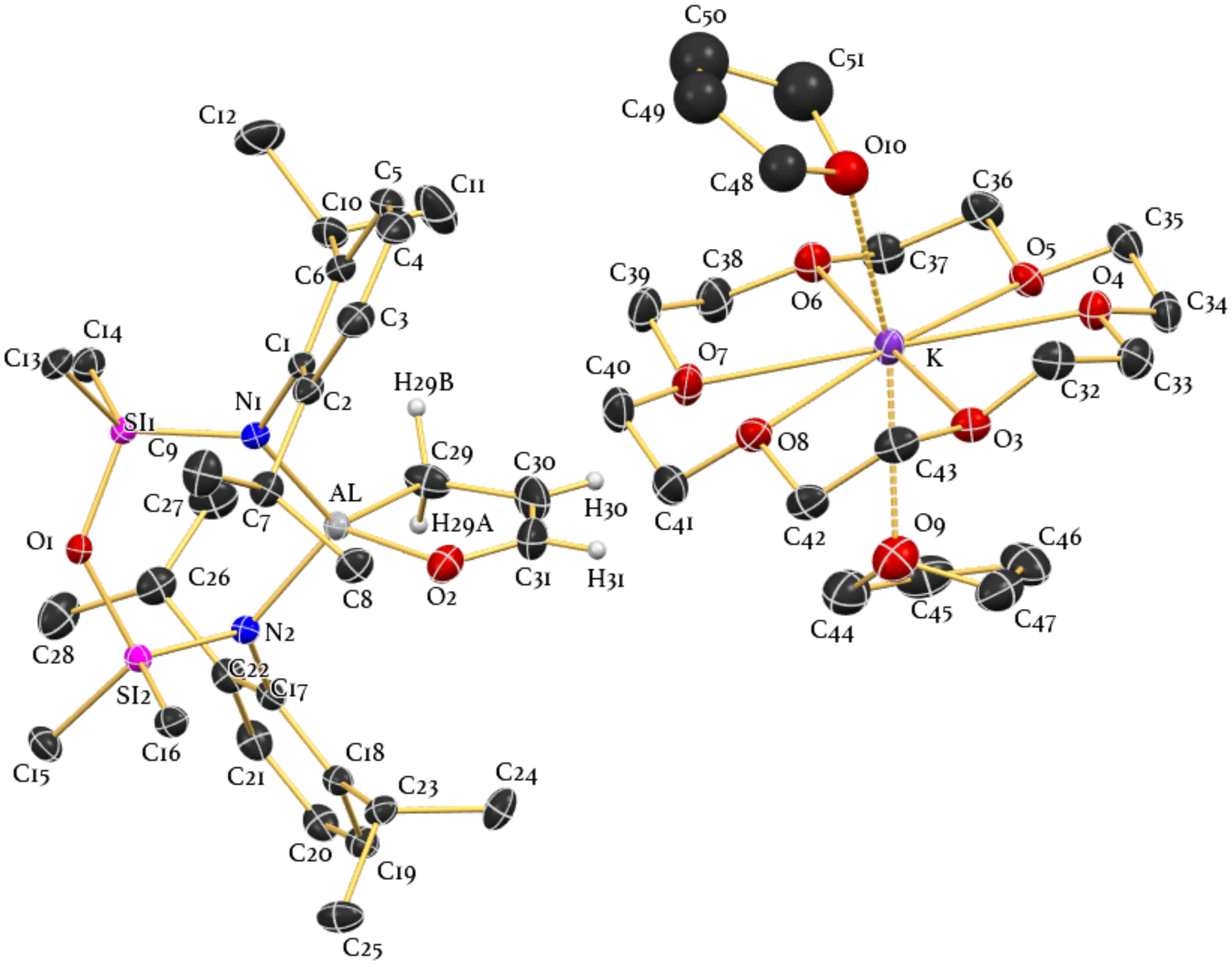

Crystallographic Details

Crystals were covered in inert oil and suitable single crystals were selected under a microscope and mounted on an Agilent SuperNova diffractometer fitted with an EOS S2 detector. Data were collected at the temperature indicated using focused micro-source Cu K_α radiation at 1.54184 Å. Intensities were corrected for Lorentz and polarisation effects and for absorption using multi-scan methods.[2] Space groups were determined from systematic absences and checked for higher symmetry. All structures were solved using direct methods with SHELXS,[3] refined on F^2 using all data by full matrix least-squares procedures with SHELXL-97,[4] within the WinGX[5] program. Non-hydrogen atoms were refined with anisotropic displacement parameters. Hydrogen atoms were placed in calculated positions or manually assigned from residual electron density where appropriate, unless otherwise stated. The functions minimized were $\Sigma w(F_o^2 - F_c^2)$, with $w = 1/[\sigma^2(F_o^2) + (aP)^2 + bP]-1$, where $P = [2F_c^2 + \mathrm{Max}(F_o^2, 0)]/3$. The isotropic displacement parameters are 1.2 or 1.5 times the isotropic equivalent of their carrier atoms.

Additional Details:

$[K_2(C_6H_6)_2][Al(NON^{Dipp})(C_2H_4)]_2 \cdot C_6H_6$ (**[1·C$_6$H$_6$]$_2$·C$_6$H$_6$**): The asymmetric unit contain a molecule of benzene solvent.

$K_2[Al(NON^{Dipp})(\mu\text{-}CH_2CH{=}CO)Al(NON^{Dipp})Et]$ (**2**): The molecule lies on a crystallographic 2-fold axis that passes between the two aluminium centres. The both the CH_2CH_3 and CH_2CHCO ligands are disordered by symmetry over two positions with 50% occupancy. The C29 and C31 (CH_2) atoms of each component are coincident. There is also disorder in one of the Me groups of an *i*Pr substituent, which was modelled over two positions.

$[K(18\text{-crown-}6)(THF)][Al(NON^{Dipp})(CH_2CH_2C{=}O)] \cdot THF$ (**4·THF**): The asymmetric unit contain a disordered molecule of THF solvent. One of the methylene groups was modelled over two positions and the atoms were refined isotropically.

$[K(18\text{-c-}6)(THF)_2][Al(NON^{Dipp})(CH_2CH{=}CHO)] \cdot THF$ (**5·THF**): The asymmetric unit contain a molecule of THF solvent. One of the THF molecules coordinated at potassium is highly disordered. It was modelled over two positions that were restrained to be equal using the SAME command. Attempts to refine the atoms aniostropically failed to give a satisfactory model, and they are included as isotropic atoms.

Table S1 Crystal structure and refinement data for $[K_2(C_6H_6)_2][Al(NON^{Dipp})(C_2H_4)]_2{\cdot}C_6H_6$ (**$[1{\cdot}C_6H_6]_2{\cdot}C_6H_6$**), $K_2[Al(NON^{Dipp})(\mu\text{-}CH_2CH{=}CO)Al(NON^{Dipp})Et]$ (**2**) and $[K(18\text{-crown-6})][Al(NON^{Dipp})(C_2H_4)]$ (**3**)

	**$[1{\cdot}C_6H_6]_2{\cdot}C_6H_6$**	**2**	**3**
Empirical formula	$C_{78}H_{118}Al_2K_2N_4O_2Si_4$	$C_{61}H_{100}Al_2K_2N_4O_3Si_4$	$C_{42}H_{74}AlKN_2O_7Si_2$
CCDC Number	2126652	2126653	2126654
M_r	1388.28	1181.97	841.29
T [K]	120.0(1)	120.0(1)	120.0(1)
Crystal size [mm]	0.53 × 0.34 × 0.16	0.29 × 0.22 × 0.17	0.26 × 0.21 × 0.06
Crystal system	Orthorhombic	Monoclinic	Monoclinic
Space group	$P2_12_12_1$ (No.19)	C2/c (No.15)	$P2_1/n$ (alternative No.14)
a [Å]	16.40787(5)	22.32757(17)	9.82290(18)
b [Å]	18.08249(6)	11.07152(10)	22.5801(4)
c [Å]	27.65476(9)	27.5620(2)	21.6469(4)
α [°]	90	90	90
β [°]	90	105.0764(8)	93.7197(17)
γ [°]	90	90	90
V [Å^3]	8205.03(5)	6578.81(9)	4791.21(15)
Z	4	4	4
$D_{calc.}$ [mg m^{-3}]	1.124	1.193	1.166
Absorption coefficient [mm^{-1}]	2.12	2.571	1.994
θ range for data collection [°]	3.638 to 73.484	3.321 to 73.460	3.915 to 75.699
Reflections collected	119256	36586	67956
Independent reflections	16465 [R_{int} 0.046]	6594 [R_{int} 0.023]	9637 [R_{int} 0.042]
Reflections with $I > 2\sigma(I)$	16204	6437	8924
Data/restraints/parameters	16465 / 0 / 886	6594 / 0 / 393	9637 / 0 / 524
Final R indices [$I > 2\sigma(I)$]	R_1 = 0.022, wR_2 = 0.057	R_1 = 0.041, wR_2 = 0.104	R_1 = 0.035, wR_2 = 0.094
Final R indices (all data)	R_1 = 0.022, wR_2 = 0.057	R_1 = 0.041, wR_2 = 0.104	R_1 = 0.038, wR_2 = 0.098
GOOF on F^2	1.05	1.117	1.063
Largest diff. peak/hole [e.Å^{-3}]	0.18 and –0.18	0.59 and –0.30	0.27 and –0.27
Flack parameter	0.001(5)	-	-

Table S2 Crystal structure and refinement data for [K(18-crown-6)(THF)][Al(NON^{Dipp})(CH_2CH_2C=O)]·THF (**4·THF**) and [K(18-c-6)(THF)$_2$][Al(NON^{Dipp})(CH_2CH=CHO)]·THF (**5·THF**)

	**4·THF**	**5·THF**
Empirical formula	$C_{51}H_{90}AlKN_2O_{10}Si_2$	$C_{55}H_{98}AlKN_2O_{11}Si_2$
CCDC Number	2126655	2126656
M_r	1013.50	1085.61
T [K]	120.0(1)	128(12)
Crystal size [mm]	0.27 × 0.22 × 0.12	0.43 × 0.27 × 0.06
Crystal system	Monoclinic	Monoclinic
Space group	P2$_1$/c (No.14)	P2$_1$/n (Alternative No.14)
a [Å]	12.59785(10)	20.6978(2)
b [Å]	19.01311(13)	14.67839(9)
c [Å]	24.67219(19)	22.4651(2)
α [°]	90	90
β [°]	101.2950(7)	115.1222(11)
γ [°]	90	90
V [Å^3]	5795.11(7)	6179.50(9)
Z	4	4
$D_{calc.}$ [mg m^{-3}]	1.162	1.167
Absorption coefficient [mm^{-1}]	1.766	1.700
θ range for data collection [°]	3.578 to 73.449	3.714 to 73.399
Reflections collected	76976	84371
Independent reflections	11648 [R_{int} 0.034]	12382 [R_{int} 0.035]
Reflections with $I > 2\sigma(I)$	10950	10877
Data/restraints/parameters	11648 / 0 / 625	12382 / 10 / 657
Final R indices [$I > 2\sigma(I)$]	R_1 = 0.066, wR_2 = 0.178	R_1 = 0.049, wR_2 = 0.134
Final R indices (all data)	R_1 = 0.068, wR_2 = 0.180	R_1 = 0.056, wR_2 = 0.141
GOOF on F^2	1.071	1.033
Largest diff. peak/hole [e.Å^{-3}]	1.04 and –0.77	0.69 and –0.67

Computational Details

DFT calculations were, unless stated otherwise, performed with Gaussian 16 (Revision A.03).[6] The Al, Si and K centres were described with the Stuttgart RECPs and associated basis sets,[7] and the 6-31G** basis set was used for all other atoms, with this combination referred to herein as "BS1".[8-9] Polarization functions were also added to Al (ζd = 0.190), Si (ζd = 0.284) and K (ζd = 1.000). Initial BP86[10-11] optimizations were performed using the 'grid = ultrafine' option, with all stationary points being fully characterized via analytical frequency calculations as either minima (all positive eigenvalues) or transition states (one negative eigenvalue). IRC calculations and subsequent geometry optimizations were used to confirm the minima linked by each transition state. All energies were recomputed with a larger basis set featuring 6-311++G** on all atoms. Corrections for the effect of benzene (ε = 2.2706) solvent were run using the polarizable continuum model and BS1.[12] Single-point dispersion corrections to the BP86 results employed Grimme's D3 parameter set with Becke-Johnson damping as implemented in Gaussian.[13]

Figure S19 DFT-Calculated Free Energy Profile at the BP86-D3(BJ)-C_6H_6/6-311++G**//BP86/BS1 level (kcal mol^{-1}) for the conversion of **[1·C_6H_6]$_2$** to **2**

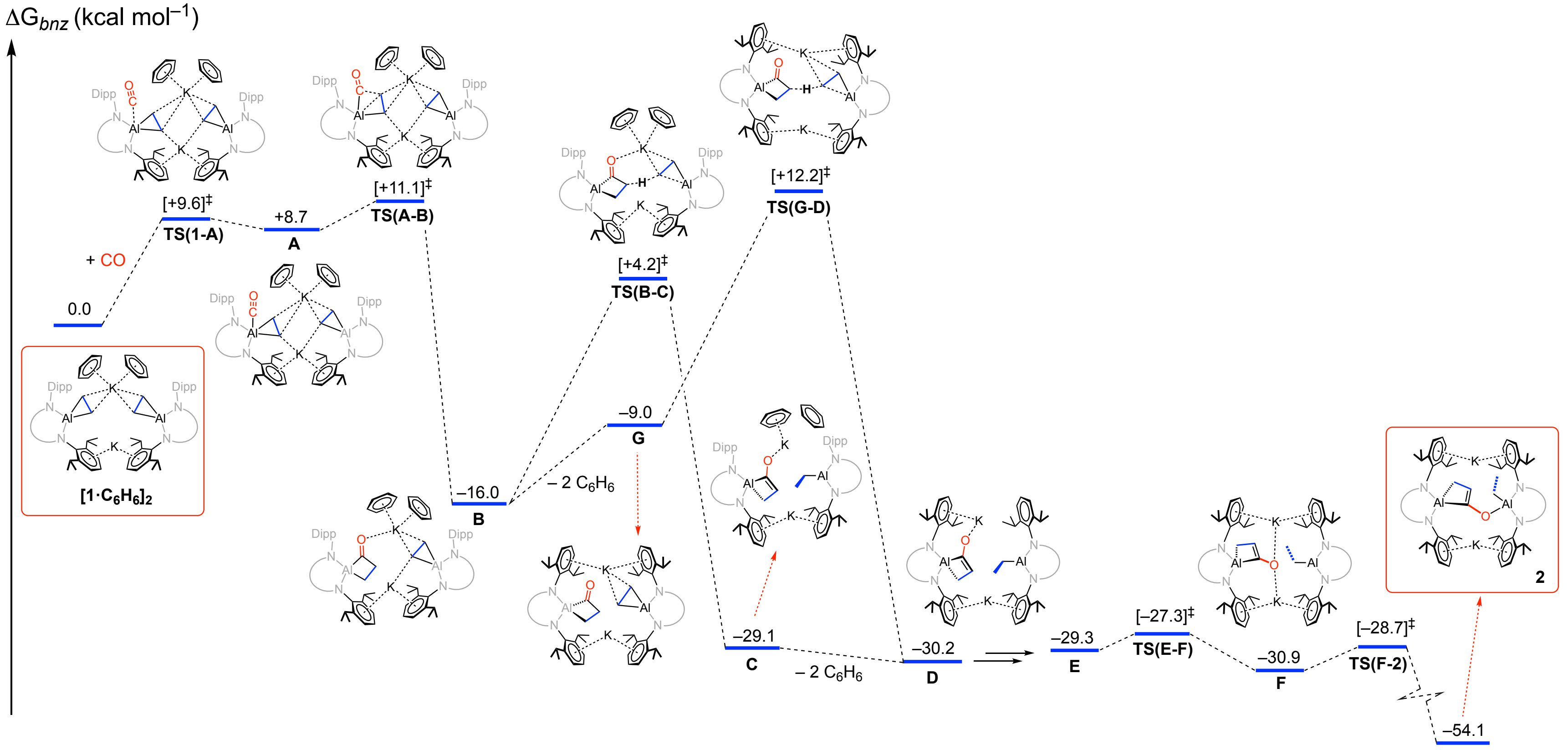

Notes

A connecting mechanistic pathway between intermediates **D** and **E** could not be explicitly characterized. However, the subtle conformational changes that appear to be required to ultimately yield **E** are small. Thus, it is expected that the transition-states would be sufficiently low in energy such that the current interpretation of the computed profile does not change.

Figure S20 DFT-Calculated Free Energy Profile at the BP86-D3(BJ)-C_6H_6/6-311++G**//BP86/BS1 level (kcal mol^{-1}) for the conversion of **[1·C_6H_6]$_2$** to **G** via a benzene dissociation pathway

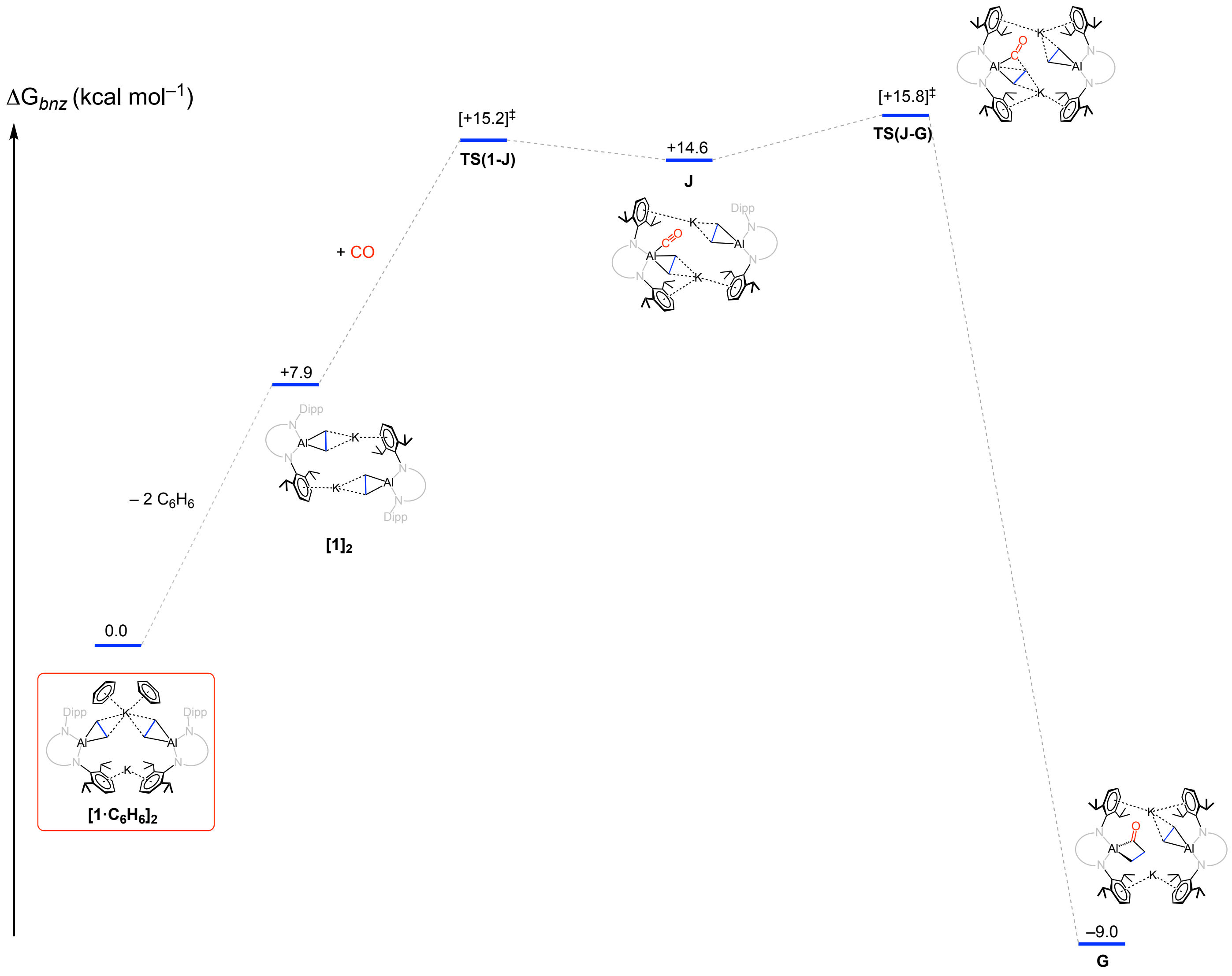

Figure S21 DFT-Calculated Free Energy Profile at the BP86-D3(BJ)-C_6H_6/6-311++G**//BP86/BS1 level (kcal mol^{-1}) for the Conversion of **3** to **4**

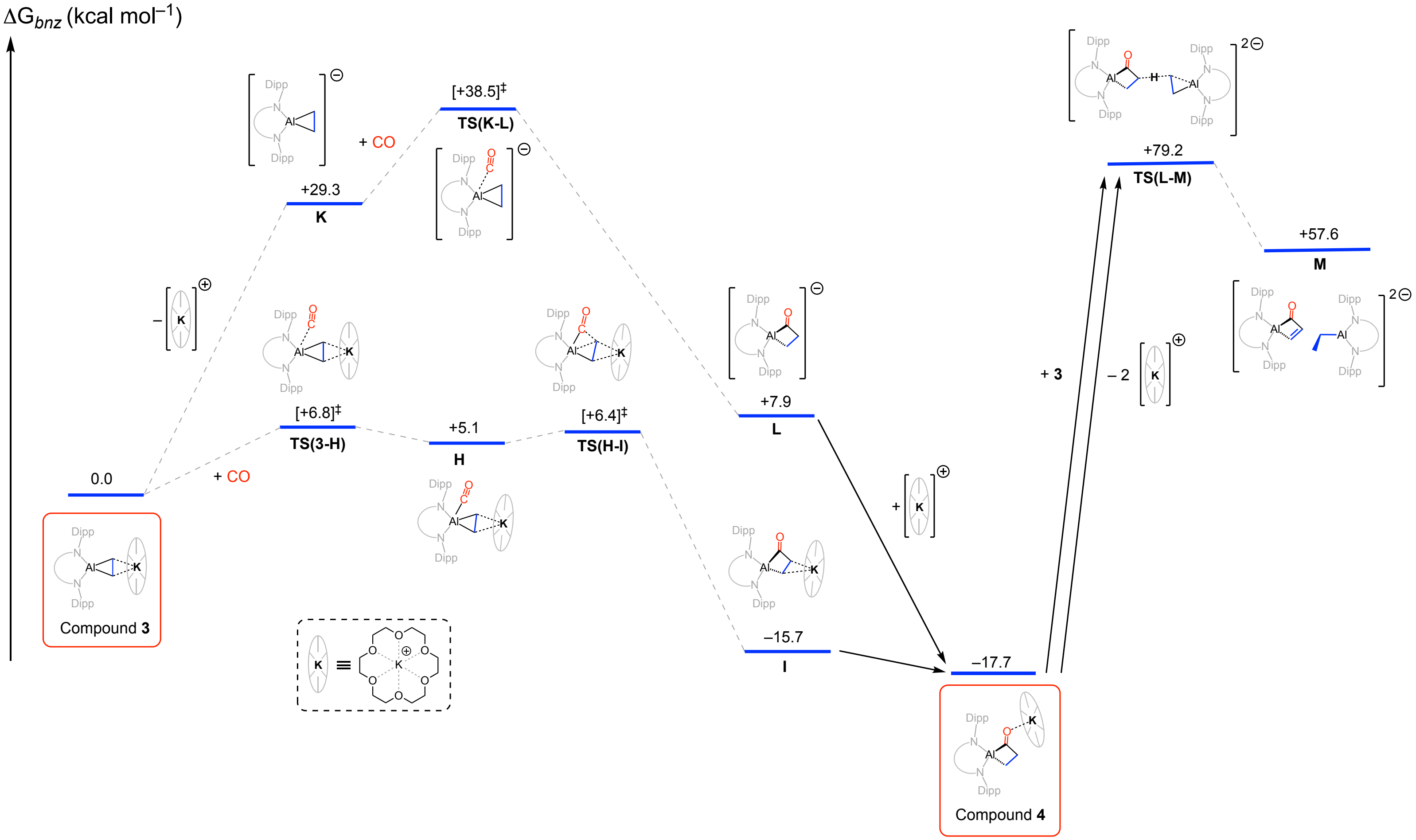

Notes

IRC calculations, and subsequent optimizations from **TS(H-I)** do not form **4** directly, but an intermediate conformer of **4** at –15.7 kcal mol^{-1} (**I**). While an explicit pathway from **I** to **4** was not fully characterized, it is expected that this pathway is low in energy as the conformational changes required appear to be subtle in nature.

Breakdown of Energy Contributions

The following table details the evolution of the relative energies as the successive corrections to the initial SCF energy are included.

Terms used are:

$\mathbf{\Delta E_{BSI}}$	SCF energy computed with the BP86 functional with BS1
$\mathbf{\Delta H_{BSI}}$	Enthalpy at 0 K with BS1
$\mathbf{\Delta G_{BSI}}$	Free energy at 298.15 K and 1 atm with BS1
$\mathbf{\Delta G_{BSI/C_6H_6}}$	Free energy corrected for C_6H_6 solvent with BS1
$\mathbf{\Delta G_{BSI/C_6H_6+D3BJ}}$	Free energy corrected for C_6H_6 and dispersion effects (D3BJ) with BS1
$\mathbf{\Delta E_{6-311++G^{**}}}$	SCF energy computed with the BP86 functional with 6-311++G**
$\mathbf{\Delta G_{C_6H_6}}$	Free energy corrected for 6-311++G**, D3BJ and C_6H_6 solvent

In each case the final data used in the main article are highlighted in bold.

Table S3 Relative energies (kcal/mol) for computed structures. Data in bold are those used in the main text. Energies are quoted relative to [**1**·C_6H_6]$_2$ (**1** → **2**) and **3** (**3** → **4**) at 0.0 kcal/mol.

**1 → 2**	**ΔE_{BSI}**	**ΔH_{BSI}**	**ΔG_{BSI}**	**$\Delta G_{BSI/C_6H_6}$**	**$\Delta G_{BSI/C_6H_6+D3BJ}$**	**$\Delta E_{6-311++G**}$**	**ΔG_{bnz}**
**[1·C_6H_6]$_2$**	0.0	0.0	0.0	0.0	0.0	0.0	**0.0**
**TS(1-A)**	0.9	1.6	14.6	15.0	6.2	4.2	**9.6**
**A**	-0.2	1.0	13.7	13.8	4.3	4.2	**8.7**
**TS(A-B)**	0.4	1.2	15.5	15.4	6.4	5.0	**11.1**
**B**	-34.4	-31.9	-19.6	-20.3	-22.6	-27.8	**-16.0**
**TS(B-C)**	-14.2	-14.4	-0.1	-1.3	-2.8	-7.2	**4.2**
**C**	-53.6	-49.6	-38.4	-39.1	-33.9	-48.9	**-29.1**
**D**	-46.4	-44.8	-51.5	-55.5	-30.7	-45.9	**-30.2**
**E**	-47.4	-45.6	-50.7	-52.9	-30.6	-46.2	**-29.3**
**TS(E-F)**	-46.6	-45.3	-47.6	-49.7	-28.7	-45.1	**-27.3**
**F**	-47.2	-45.2	-48.6	-50.2	-33.2	-44.9	**-30.9**
**TS(F-2)**	-46.2	-44.8	-44.4	-45.0	-31.6	-43.3	**-28.7**
**2**	-71.3	-68.7	-65.4	-64.9	-60.7	-64.7	**-54.1**
**G**	-21.9	-21.6	-27.5	-31.5	-10.5	-20.4	**-9.0**
**TS(G-D)**	1.6	-1.1	-4.0	-9.2	8.9	5.0	**12.2**
**[1]$_2$**	10.1	7.8	-10.1	-13.5	13.2	4.8	**7.9**
**TS(1-J)**	11.4	9.5	1.8	-0.8	18.0	8.5	**15.2**
**J**	9.5	8.1	2.1	-0.5	15.5	8.5	**14.6**
**TS(J-G)**	11.1	9.3	3.1	0.1	16.5	10.4	**15.8**
**3 → 4**							
**3**	0.0	0.0	0.0	0.0	0.0	0.0	**0.0**
**TS(3-H)**	0.3	0.7	9.6	9.7	4.8	2.3	**6.8**
**H**	-1.9	-0.8	10.8	11.3	0.9	2.4	**5.1**
**TS(H-I)**	-1.3	-0.7	11.8	12.0	2.0	3.2	**6.4**
**I**	-30.8	-27.9	-14.1	-15.0	-22.3	-24.1	**-15.7**
**4**	-35.4	-32.8	-20.8	-21.3	-23.9	-29.2	**-17.7**
**K**	62.2	61.1	49.8	14.4	33.5	58.0	**29.3**
**TS(K-L)**	63.0	62.6	63.1	28.1	40.1	61.4	**38.5**
**L**	26.8	28.2	28.8	-6.5	6.9	27.8	**7.9**
**TS(L-M)**	152.6	150.6	152.3	57.7	79.9	151.9	**79.2**
**M**	132.4	133.9	136.2	38.0	57.0	133.1	**57.6**

References

(1) Schwamm, R. J.; Anker, M. D.; Lein, M.; Coles, M. P. Reduction vs. Addition: The Reaction of an Aluminyl Anion with 1,3,5,7-Cyclooctatetraene. *Angew. Chem. Int. Ed.* **2019**, *58*, 1489-1493.

(2) Blessing, R. An empirical correction for absorption anisotropy. *Acta Cryst.* **1995**, *A51*, 33-38.

(3) Sheldrick, G. M. A short history of SHELX. *Acta Cryst.* **2008**, *A64*, 112-122.

(4) Sheldrick, G. M. *SHELXL-97*, University of Gottingen, Germany, 1997.

(5) Farrugia, L. J. WinGX suite for small-molecule single-crystal crystallography. *J. Appl. Cryst.* **1999**, *32*, 837-838.

(6) Frisch, M. J.; Trucks, G. W.; Schlegel, H. B.; Scuseria, G. E.; Robb, M. A.; Cheeseman, J. R.; Scalmani, G.; Barone, V.; Petersson, G. A.; Nakatsuji, H.; Li, X.; Caricato, M.; Marenich, A. V.; Bloino, J.; Janesko, B. G.; Gomperts, R.; Mennucci, B.; Hratchian, H. P.; Ortiz, J. V.; Izmaylov, A. F.; Sonnenberg, J. L.; Williams; Ding, F.; Lipparini, F.; Egidi, F.; Goings, J.; Peng, B.; Petrone, A.; Henderson, T.; Ranasinghe, D.; Zakrzewski, V. G.; Gao, J.; Rega, N.; Zheng, G.; Liang, W.; Hada, M.; Ehara, M.; Toyota, K.; Fukuda, R.; Hasegawa, J.; Ishida, M.; Nakajima, T.; Honda, Y.; Kitao, O.; Nakai, H.; Vreven, T.; Throssell, K.; Montgomery Jr., J. A.; Peralta, J. E.; Ogliaro, F.; Bearpark, M. J.; Heyd, J. J.; Brothers, E. N.; Kudin, K. N.; Staroverov, V. N.; Keith, T. A.; Kobayashi, R.; Normand, J.; Raghavachari, K.; Rendell, A. P.; Burant, J. C.; Iyengar, S. S.; Tomasi, J.; Cossi, M.; Millam, J. M.; Klene, M.; Adamo, C.; Cammi, R.; Ochterski, J. W.; Martin, R. L.; Morokuma, K.; Farkas, O.; Foresman, J. B.; Fox, D. J. *Gaussian 16 Rev. A.03*, Wallingford, CT, 2016.

(7) Andrae, D.; Häußermann, U.; Dolg, M.; Stoll, H.; Preuß, H. Energy-adjustedab initio pseudopotentials for the second and third row transition elements. *Theor. Chim. Acta* **1990**, *77*, 123-141.

(8) Hariharan, P. C.; Pople, J. A. The influence of polarization functions on molecular orbital hydrogenation energies. *Theor. Chim. Acta* **1973**, *28*, 213-222.

(9) Hehre, W. J.; Ditchfield, R.; Pople, J. A. Self—Consistent Molecular Orbital Methods. XII. Further Extensions of Gaussian—Type Basis Sets for Use in Molecular Orbital Studies of Organic Molecules. *J. Chem. Phys.* **1972**, *56*, 2257-2261.

(10) Becke, A. D. Density-functional exchange-energy approximation with correct asymptotic behavior. *Phys. Rev. A* **1988**, *38*, 3098-3100.

(11) Perdew, J. P. Density-functional approximation for the correlation energy of the inhomogeneous electron gas. *Phys. Rev. B* **1986**, *33*, 8822-8824.

(12) Tomasi, J.; Mennucci, B.; Cammi, R. Quantum Mechanical Continuum Solvation Models. *Chem. Rev.* **2005**, *105*, 2999-3094.

(13) Grimme, S.; Ehrlich, S.; Goerigk, L. Effect of the damping function in dispersion corrected density functional theory. *J. Comp. Chem.* **2011**, *32*, 1456-1465.

(14) Glendening, E. D.; Badenhoop, J., K.; Reed, A. E.; Carpenter, J. E.; Bohmann, J. A.; Morales, C. M.; Karafiloglou, P.; Landis, C. R.; Weinhold, F. *NBO 7.0.10*, Theoretical Chemistry Institute, University of Wisconsin, Madison., 2018.

(15) Frisch, M. J.; Trucks, G. W.; Schlegel, H. B.; Scuseria, G. E.; Robb, M. A.; Cheeseman, J. R.; Scalmani, G.; Barone, V.; Petersson, G. A.; Nakatsuji, H.; Li, X.; Caricato, M.; Marenich, A. V.; Bloino, J.; Janesko, B. G.; Gomperts, R.; Mennucci, B.; Hratchian, H. P.; Ortiz, J. V.; Izmaylov, A. F.; Sonnenberg, J. L.; Williams; Ding, F.; Lipparini, F.; Egidi, F.; Goings, J.; Peng, B.; Petrone, A.; Henderson, T.; Ranasinghe, D.; Zakrzewski, V. G.; Gao, J.; Rega, N.; Zheng, G.; Liang, W.; Hada, M.; Ehara, M.; Toyota, K.; Fukuda, R.; Hasegawa, J.; Ishida, M.; Nakajima, T.; Honda, Y.; Kitao, O.; Nakai, H.; Vreven, T.; Throssell, K.; Montgomery Jr., J.

A.; Peralta, J. E.; Ogliaro, F.; Bearpark, M. J.; Heyd, J. J.; Brothers, E. N.; Kudin, K. N.; Staroverov, V. N.; Keith, T. A.; Kobayashi, R.; Normand, J.; Raghavachari, K.; Rendell, A. P.; Burant, J. C.; Iyengar, S. S.; Tomasi, J.; Cossi, M.; Millam, J. M.; Klene, M.; Adamo, C.; Cammi, R.; Ochterski, J. W.; Martin, R. L.; Morokuma, K.; Farkas, O.; Foresman, J. B.; Fox, D. J. *Gaussian 16 Rev. C.01*, Wallingford, CT, 2016.

(16) Keith, T. A. *AIMAll* Version 19.10.12; TK Gristmill Software: Overland Park KS, USA, , 2019.

Cartesian Coordinates and Energies (Hartree) of Computed Structures

C_6H_6

```
SCF (BP86) Energy = -232.242068253
Enthalpy 0K = -232.139828
Enthalpy 298K = -232.138884
Free Energy 298K = -232.171901
Lowest Frequency = 397.3340 cm-1
Second Frequency = 397.5672 cm-1
SCF (BP86-D3BJ) Energy =  -232.258990187
SCF (C6H6) Energy =  -232.243166506
SCF (6-311++G**) Energy =  -232.299997471

C  -1.06464  0.91646  0.00000
C   0.26115  1.38021 -0.00001
C  -1.32602 -0.46399  0.00004
H   0.46532  2.45638  0.00000
H  -2.36007 -0.82525  0.00004
C   1.32610  0.46378 -0.00003
C  -0.26135 -1.38012 -0.00007
H   2.35977  0.82603  0.00001
H  -0.46453 -2.45651 -0.00006
C   1.06473 -0.91640  0.00007
H   1.89479 -1.63106  0.00004
H  -1.89505  1.63077 -0.00003
```

CO

```
SCF (BP86) Energy = -113.305193181
Enthalpy 0K = -113.298010
Enthalpy 298K = -113.297065
Free Energy 298K = -113.319529
Lowest Frequency = 2116.9106 cm-1
Second Frequency =  cm-1
SCF (BP86-D3BJ) Energy =  -113.305815827
SCF (C6H6) Energy =  -113.305476554
SCF (6-311++G**) Energy =  -113.346138993

C   0.00000  0.00000 -0.65707
O   0.00000  0.00000  0.49280
```

K_18_crown_6

```
SCF (BP86) Energy = -951.242917287
Enthalpy 0K = -950.861416
Enthalpy 298K = -950.860472
Free Energy 298K = -950.933933
Lowest Frequency = 36.9545 cm-1
Second Frequency = 37.0049 cm-1
SCF (BP86-D3BJ) Energy =  -951.315978073
SCF (C6H6) Energy =  -951.277819226
SCF (6-311++G**) Energy =  -1523.14555802

K   0.00009  0.00005 -0.00040
O   2.44209 -1.39762 -0.18286
O   2.43151  1.41590  0.18264
O  -0.01055  2.81315 -0.18289
O  -2.44226  1.39772  0.18306
O  -2.43146 -1.41587 -0.18243
O   0.01055 -2.81344  0.18336
C   3.58997 -0.68485  0.29690
H   4.52429 -1.20122 -0.00663
H   3.57544 -0.62811  1.40612
C   3.58472  0.71179 -0.29710
H   4.51513  1.23517  0.00645
H   3.57063  0.65491 -1.40631
C   2.38802  2.76628 -0.29737
H   2.33157  2.78183 -1.40659
H   3.30232  3.31738  0.00603
C   1.17583  3.46010  0.29650
H   1.21813  3.41994  1.40572
H   1.18762  4.52742 -0.00750
C  -1.20176  3.45119  0.29649
H  -1.22156  4.51837 -0.00756
H  -1.24375  3.41076  1.40572
C  -2.40871  2.74824 -0.29731
H  -3.32710  3.29265  0.00583
H  -2.35224  2.76392 -1.40653
C  -3.58997  0.68484 -0.29690
H  -3.57522  0.62804 -1.40612
H  -4.52440  1.20115  0.00640
C  -3.58475 -0.71178  0.29715
H  -3.57080 -0.65487  1.40637
H  -4.51510 -1.23520 -0.00650
C  -2.38802 -2.76625  0.29760
H  -3.30235 -3.31730 -0.00579
H  -2.33156 -2.78179  1.40682
C  -1.17588 -3.46012 -0.29628
H  -1.18784 -4.52752  0.00745
H  -1.21809 -3.41969 -1.40551
C   1.20171 -3.45113 -0.29659
H   1.24342 -3.41025 -1.40582
H   1.22172 -4.51845  0.00699
C   2.40874 -2.74829  0.29716
H   2.35251 -2.76428  1.40638
H   3.32711 -3.29254 -0.00634
```

[1·C_6H_6]$_2$

```
SCF (BP86) Energy = -3257.42606324
Enthalpy 0K = -3255.682733
Enthalpy 298K = -3255.681789
Free Energy 298K = -3255.960410
Lowest Frequency = 6.1788 cm-1
Second Frequency = 8.7272 cm-1
SCF (BP86-D3BJ) Energy =  -3257.99483873
SCF (C6H6) Energy =  -3257.43593046
SCF (6-311++G**) Energy =  -6024.87968917

K   0.00011  2.73053 -0.00026
K  -0.00054 -2.40336 -0.00011
Si -5.56638 -1.91351  2.31249
Si -6.84210 -0.95812 -0.36711
Si  6.84208 -0.95849  0.36726
Si  5.56632 -1.91403 -2.31226
Al -3.80184 -0.16111  0.39777
Al  3.80194 -0.16119 -0.39780
O  -6.76783 -1.77951  1.12286
O   6.76775 -1.78009 -1.12259
N  -3.98182 -1.66554  1.54189
N  -5.46449  0.15434 -0.46187
N   5.46462  0.15417  0.46183
N   3.98177 -1.66577 -1.54175
C  -2.92869 -2.59127  1.77514
C  -2.79935 -3.77773  0.96879
C  -1.77515 -4.70630  1.24815
H  -1.70778 -5.62186  0.64973
C  -0.85824 -4.49955  2.29210
H  -0.08756 -5.24628  2.51213
C  -0.96588 -3.33610  3.06735
H  -0.26402 -3.17559  3.89439
C  -1.98274 -2.38423  2.83633
C  -3.74552 -4.03381 -0.20308
H  -4.64980 -3.43276 -0.01125
C  -3.12166 -3.51134 -1.51989
H  -2.18617 -4.05543 -1.75413
H  -2.90018 -2.42952 -1.45379
H  -3.80804 -3.66124 -2.37216
C  -4.17372 -5.50725 -0.35194
H  -4.57244 -5.91344  0.59289
H  -3.33896 -6.15694 -0.67222
```

H	-4.96196	-5.59374	-1.11919
C	-2.08514	-1.17720	3.76793
H	-2.88254	-0.53127	3.36209
C	-0.77895	-0.35531	3.81272
H	-0.48237	-0.00619	2.80788
H	0.06244	-0.93718	4.22974
H	-0.91100	0.53330	4.45562
C	-2.49248	-1.61080	5.19538
H	-1.71966	-2.25266	5.65538
H	-3.43661	-2.18050	5.19057
H	-2.62818	-0.72981	5.84735
C	-5.69814	-3.64309	3.07075
H	-5.57683	-4.42959	2.30865
H	-6.69357	-3.76566	3.53023
H	-4.93823	-3.80583	3.85336
C	-5.93177	-0.59648	3.62878
H	-6.98244	-0.66416	3.95913
H	-5.76494	0.41834	3.23053
H	-5.28528	-0.72239	4.51377
C	-6.88793	-2.32724	-1.68740
H	-7.63962	-3.08185	-1.39705
H	-5.91793	-2.83773	-1.79669
H	-7.17407	-1.92785	-2.67448
C	-8.49930	-0.04349	-0.44695
H	-8.69606	0.50556	0.48691
H	-9.31582	-0.77116	-0.59282
H	-8.52742	0.67427	-1.28391
C	-5.58299	1.41290	-1.14853
C	-5.30099	1.50726	-2.55162
C	-5.34125	2.76362	-3.18965
H	-5.13332	2.82170	-4.26516
C	-5.65822	3.93275	-2.48731
H	-5.68925	4.89988	-3.00127
C	-5.97004	3.83871	-1.12549
H	-6.25296	4.74463	-0.57587
C	-5.95667	2.60589	-0.44301
C	-5.00670	0.27080	-3.40305
H	-4.99628	-0.59266	-2.71735
C	-6.12012	0.04687	-4.45322
H	-6.14126	0.86592	-5.19447
H	-7.11764	0.00388	-3.98457
H	-5.95717	-0.89687	-5.00386
C	-3.62960	0.33199	-4.09893
H	-2.81371	0.41424	-3.36156
H	-3.55880	1.19177	-4.78985
H	-3.45608	-0.58209	-4.69497
C	-6.38550	2.59071	1.02603
H	-6.42327	1.53119	1.33280
C	-7.79305	3.20225	1.21927
H	-8.53840	2.73873	0.55313
H	-7.79393	4.28798	1.01461
H	-8.13042	3.06620	2.26217
C	-5.37311	3.31048	1.94612
H	-5.26321	4.37248	1.65833
H	-4.38584	2.82541	1.89073
H	-5.71945	3.28568	2.99544
C	-2.01525	0.34743	-0.36378
H	-2.02115	0.75476	-1.39055
H	-0.99115	-0.01461	-0.14413
C	-2.59276	1.37068	0.71675
H	-2.81357	2.38900	0.35129
H	-2.03404	1.39760	1.66813
C	5.58320	1.41276	1.14839
C	5.30109	1.50727	2.55145
C	5.34139	2.76368	3.18937
H	5.13333	2.82187	4.26486
C	5.65852	3.93273	2.48696
H	5.68958	4.89990	3.00084
C	5.97045	3.83854	1.12518
H	6.25348	4.74439	0.57549
C	5.95703	2.60567	0.44280
C	5.00654	0.27091	3.40293
H	4.99606	-0.59259	2.71729
C	3.62937	0.33234	4.09865
H	2.81358	0.41462	3.36117
H	3.55860	1.19219	4.78948
H	3.45567	-0.58166	4.69476
C	6.11982	0.04687	4.45323
H	6.14102	0.86597	5.19442
H	7.11738	0.00367	3.98468
H	5.95666	-0.89681	5.00392
C	6.38596	2.59032	-1.02621
H	6.42368	1.53077	-1.33288
C	5.37367	3.31007	-1.94643
H	5.26382	4.37211	-1.65875
H	4.38637	2.82507	-1.89106
H	5.72007	3.28514	-2.99573
C	7.79356	3.20175	-1.21941
H	8.53884	2.73824	-0.55318
H	7.79450	4.28750	-1.01485
H	8.13099	3.06559	-2.26228
C	6.88771	-2.32745	1.68773
H	7.63934	-3.08216	1.39752
H	5.91765	-2.83783	1.79702
H	7.17382	-1.92797	2.67479
C	8.49939	-0.04405	0.44702
H	8.69617	0.50498	-0.48686
H	9.31584	-0.77180	0.59284
H	8.52763	0.67371	1.28397
C	5.69787	-3.64370	-3.07034
H	5.57634	-4.43011	-2.30818
H	6.69333	-3.76649	-3.52971
H	4.93802	-3.80639	-3.85302
C	5.93195	-0.59718	-3.62866
H	6.98261	-0.66507	-3.95900
H	5.76531	0.41771	-3.23048
H	5.28544	-0.72304	-4.51364
C	2.92852	-2.59137	-1.77498
C	1.98270	-2.38429	-2.83627
C	0.96577	-3.33608	-3.06735
H	0.26403	-3.17555	-3.89449
C	0.85793	-4.49946	-2.29204
H	0.08723	-5.24615	-2.51214
C	1.77468	-4.70622	-1.24795
H	1.70715	-5.62174	-0.64948
C	2.79894	-3.77773	-0.96854
C	2.08529	-1.17729	-3.76789
H	2.88271	-0.53143	-3.36199
C	2.49271	-1.61093	-5.19531
H	1.71989	-2.25274	-5.65537
H	3.43680	-2.18069	-5.19041
H	2.62852	-0.72995	-5.84727
C	0.77917	-0.35530	-3.81277
H	0.48252	-0.00621	-2.80795
H	-0.06222	-0.93709	-4.22991
H	0.91134	0.53334	-4.45560
C	3.74493	-4.03381	0.20347
H	4.64934	-3.43294	0.01164
C	4.17286	-5.50730	0.35260
H	4.57164	-5.91370	-0.59212
H	3.33796	-6.15679	0.67287
H	4.96099	-5.59380	1.11997
C	3.12101	-3.51102	1.52012
H	2.18544	-4.05497	1.75437
H	2.89964	-2.42920	1.45379
H	3.80729	-3.66085	2.37248
C	2.59309	1.37077	-0.71695
H	2.81418	2.38894	-0.35123
H	2.03443	1.39806	-1.66835
C	2.01529	0.34748	0.36342
H	2.02044	0.75479	1.39020
H	0.99147	-0.01491	0.14307

```
C  -1.76438  3.76693 -2.72782
H  -2.59999  3.08871 -2.92960
C  -1.98220  4.91367 -1.94126
H  -2.98437  5.10715 -1.54504
C  -0.92398  5.80686 -1.69319
H  -1.09778  6.70969 -1.09832
C   0.35398  5.55137 -2.22446
H   1.17485  6.25305 -2.04258
C   0.57429  4.40284 -3.00686
H   1.56595  4.20934 -3.42852
C  -0.48688  3.51298 -3.26072
H  -0.32082  2.62799 -3.88361
C   0.48737  3.51279  3.25969
H   0.32145  2.62725  3.88182
C  -0.57391  4.40279  3.00673
H  -1.56550  4.20883  3.42833
C  -0.35378  5.55202  2.22532
H  -1.17473  6.25380  2.04415
C   0.92410  5.80809  1.69414
H   1.09776  6.71147  1.10005
C   1.98242  4.91478  1.94131
H   2.98453  5.10870  1.54516
C   1.76480  3.76733  2.72688
H   2.60048  3.08901  2.92799
```

TS(1-A)

```
SCF (BP86) Energy = -3370.72981988
Enthalpy 0K = -3368.977269
Enthalpy 298K = -3368.976325
Free Energy 298K = -3369.256726
Lowest Frequency = -21.7087 cm-1
Second Frequency = 6.5977 cm-1
SCF (BP86-D3BJ) Energy =  -3371.31310879
SCF (C6H6) Energy =  -3370.73933819
SCF (6-311++G**) Energy =  -6138.21909596

K   0.00442  2.69328 -0.13470
K   0.31452 -2.38760  0.08240
Si  5.75667 -1.96724 -2.14701
Si  6.90934 -0.80419  0.50370
Si -6.84372 -0.86350 -0.27404
Si -5.59750 -2.04729  2.34325
Al  3.86406 -0.16988 -0.39576
Al -3.85044 -0.15126  0.45221
O   6.91605 -1.71425 -0.93644
O  -6.76899 -1.32032  1.36211
N   4.14064 -1.73316 -1.43674
N   5.48843  0.25837  0.48953
N  -5.41641  0.14037 -0.57744
N  -4.01863 -1.65768  1.60896
C   3.13331 -2.71611 -1.63771
C   3.02724 -3.85175 -0.75789
C   2.03297 -4.82531 -0.98848
H   1.97841 -5.69854 -0.32863
C   1.12856 -4.71556 -2.05721
H   0.37794 -5.49352 -2.23333
C   1.22296 -3.60948 -2.91365
H   0.53388 -3.52820 -3.76257
C   2.20867 -2.61510 -2.73287
C   3.96074 -4.00519  0.44220
H   4.84558 -3.38171  0.23239
C   3.29628 -3.43736  1.71982
H   2.37094 -3.99450  1.96338
H   3.04976 -2.36555  1.59928
H   3.96828 -3.52748  2.59154
C   4.43967 -5.45170  0.67772
H   4.86827 -5.89297 -0.23776
H   3.62374 -6.11370  1.02030
H   5.21766 -5.46719  1.45996
C   2.30230 -1.47899 -3.75080
H   3.06894 -0.77988 -3.37494
C   0.97566 -0.70315 -3.89572
H   0.64429 -0.27913 -2.93128
H   0.16106 -1.34149 -4.28164
H   1.09965  0.13236 -4.60768
C   2.76266 -2.00828 -5.12932
H   2.02173 -2.70699 -5.55782
H   3.72224 -2.54660 -5.05646
H   2.89105 -1.17598 -5.84366
C   5.97307 -3.73466 -2.78951
H   5.86924 -4.47569 -1.98081
H   6.98196 -3.84368 -3.22232
H   5.23701 -3.97899 -3.57358
C   6.11037 -0.72613 -3.53790
H   7.17260 -0.77354 -3.83291
H   5.89244  0.30606 -3.21675
H   5.49739 -0.93892 -4.43011
C   6.96458 -2.09094  1.90394
H   7.74884 -2.83418  1.67746
H   6.01016 -2.62801  2.02077
H   7.21121 -1.62422  2.87182
C   8.52842  0.17619  0.57763
H   8.73121  0.68258 -0.37871
H   9.36588 -0.51243  0.78250
H   8.50840  0.93711  1.37568
C   5.55343  1.56441  1.09107
C   5.24766  1.74420  2.48088
C   5.24403  3.04122  3.03327
H   5.02104  3.16500  4.10016
C   5.53505  4.16985  2.25738
H   5.53071  5.16962  2.70554
C   5.86785  3.99429  0.90884
H   6.13138  4.86949  0.30277
C   5.90284  2.71844  0.31172
C   4.97856  0.56051  3.41263
H   4.99039 -0.34769  2.78714
C   6.09424  0.43513  4.47697
H   6.09265  1.30321  5.16041
H   7.09404  0.38533  4.01399
H   5.95277 -0.47261  5.09049
C   3.59984  0.63576  4.10476
H   2.78029  0.65710  3.36688
H   3.51099  1.53426  4.74201
H   3.44562 -0.24345  4.75590
C   6.36272  2.61775 -1.14460
H   6.44375  1.54174 -1.37611
C   7.75258  3.26414 -1.35301
H   8.49783  2.87780 -0.63921
H   7.71119  4.36116 -1.22872
H   8.11854  3.06493 -2.37588
C   5.34783  3.23614 -2.13310
H   5.19309  4.30999 -1.91972
H   4.37743  2.71948 -2.06688
H   5.72057  3.15477 -3.17044
C   2.06464  0.35899  0.34632
H   2.20870  0.83325  1.33443
H   0.99455  0.07186  0.27925
C   2.60544  1.29062 -0.82459
H   2.76443  2.35839 -0.58813
H   2.05802  1.17134 -1.77551
C  -5.43844  1.18322 -1.56671
C  -5.07347  0.90454 -2.92702
C  -5.07403  1.94023 -3.88290
H  -4.80174  1.70719 -4.91955
C  -5.42942  3.25068 -3.54456
H  -5.43375  4.04164 -4.30244
C  -5.79658  3.52651 -2.22291
H  -6.09760  4.54610 -1.95310
C  -5.81449  2.52811 -1.22689
C  -4.69429 -0.50004 -3.39687
H  -4.77912 -1.16406 -2.52059
C  -3.23427 -0.56357 -3.89779
```

H	-2.53279	-0.26355	-3.10165
H	-3.07698	0.10087	-4.76678
H	-2.97472	-1.58980	-4.21522
C	-5.65569	-1.01265	-4.49367
H	-5.56309	-0.41895	-5.42080
H	-6.70876	-0.95444	-4.17147
H	-5.43245	-2.06365	-4.75016
C	-6.26239	2.94363	0.17715
H	-6.22764	2.04213	0.81313
C	-5.32186	4.00502	0.79355
H	-5.32554	4.93407	0.19438
H	-4.28548	3.63955	0.85631
H	-5.65424	4.27116	1.81257
C	-7.70661	3.50049	0.18016
H	-8.42051	2.80713	-0.28915
H	-7.76516	4.45864	-0.36724
H	-8.04245	3.69232	1.21483
C	-6.96847	-2.45373	-1.31502
H	-7.77676	-3.08704	-0.90894
H	-6.04040	-3.04717	-1.32042
H	-7.22263	-2.21983	-2.36264
C	-8.48025	0.05767	-0.50582
H	-8.67378	0.73012	0.34382
H	-9.30745	-0.67056	-0.56014
H	-8.48770	0.65162	-1.43481
C	-5.88581	-3.92203	2.43388
H	-6.02187	-4.36698	1.43555
H	-6.80084	-4.11747	3.01896
H	-5.04925	-4.44016	2.93278
C	-5.91031	-1.32978	4.06837
H	-6.96086	-1.52027	4.34938
H	-5.74439	-0.24107	4.07526
H	-5.26727	-1.79045	4.83618
C	-2.90937	-2.51903	1.86839
C	-2.02530	-2.28461	2.97634
C	-0.95746	-3.17344	3.22733
H	-0.30218	-2.98640	4.08631
C	-0.74202	-4.30903	2.43501
H	0.05962	-5.01547	2.67777
C	-1.59488	-4.54187	1.34356
H	-1.44440	-5.43505	0.72605
C	-2.65907	-3.66859	1.03647
C	-2.21367	-1.09772	3.91944
H	-3.13433	-0.58274	3.59663
C	-2.39196	-1.54592	5.38768
H	-1.46794	-1.99983	5.78889
H	-3.19821	-2.29042	5.49168
H	-2.63955	-0.68139	6.02782
C	-1.04235	-0.09559	3.81218
H	-0.91439	0.26264	2.77674
H	-0.09030	-0.55370	4.13669
H	-1.22768	0.78214	4.45622
C	-3.51067	-3.95072	-0.19890
H	-4.43544	-3.36516	-0.07228
C	-3.89191	-5.43496	-0.37090
H	-4.34820	-5.84801	0.54356
H	-3.01864	-6.06236	-0.62606
H	-4.61717	-5.54563	-1.19575
C	-2.81074	-3.41875	-1.47134
H	-1.86039	-3.95771	-1.65372
H	-2.60514	-2.33598	-1.38285
H	-3.44607	-3.56793	-2.36228
C	-2.61926	1.41313	0.64838
H	-2.96632	2.23815	0.00216
H	-2.09513	1.79675	1.53987
C	-1.97166	0.22366	-0.15985
H	-1.56681	0.43896	-1.16831
H	-1.20815	-0.28311	0.45968
C	1.68389	3.95442	2.51471
H	2.56318	3.34000	2.73501
C	1.82302	5.08679	1.69021
H	2.80900	5.33441	1.28356
C	0.70696	5.89879	1.41705
H	0.81922	6.79187	0.79312
C	-0.55025	5.57526	1.96069
H	-1.41680	6.21360	1.75821
C	-0.69294	4.44074	2.78117
H	-1.66827	4.18851	3.20819
C	0.42676	3.63346	3.05997
H	0.32001	2.76010	3.71184
C	-0.30445	3.15539	-3.47616
H	-0.04957	2.24346	-4.02567
C	0.69374	4.10872	-3.19760
H	1.72371	3.93698	-3.52642
C	0.36203	5.28995	-2.50879
H	1.13446	6.03983	-2.30809
C	-0.96471	5.51488	-2.09544
H	-1.22478	6.44161	-1.57320
C	-1.96035	4.55966	-2.37005
H	-2.99840	4.72916	-2.06667
C	-1.63092	3.37913	-3.06290
H	-2.41782	2.65187	-3.28901
C	-4.42629	1.75613	3.09537
O	-3.70530	2.50950	3.59862

A

SCF (BP86) Energy = -3370.73156962
Enthalpy 0K = -3368.978176
Enthalpy 298K = -3368.977231
Free Energy 298K = -3369.258056
Lowest Frequency = 5.1723 cm-1
Second Frequency = 8.3677 cm-1
SCF (BP86-D3BJ) Energy = -3371.31602344
SCF (C6H6) Energy = -3370.74164671
SCF (6-311++G**) Energy = -6138.21915390

K	-0.02783	2.71576	0.10364
K	-0.37127	-2.38128	0.00317
Si	-5.81429	-1.83812	2.17555
Si	-6.90888	-0.76738	-0.53924
Si	6.83653	-0.79102	0.24417
Si	5.56406	-2.20594	-2.25618
Al	-3.87557	-0.12587	0.39844
Al	3.89059	-0.12365	-0.57496
O	-6.94752	-1.62380	0.93294
O	6.71959	-1.35796	-1.35575
N	-4.18355	-1.64864	1.48693
N	-5.47513	0.27771	-0.54198
N	5.40531	0.21732	0.52097
N	3.97825	-1.76369	-1.56313
C	-3.18675	-2.63029	1.73978
C	-3.08235	-3.80330	0.91065
C	-2.09479	-4.77123	1.18889
H	-2.04068	-5.67200	0.56713
C	-1.19569	-4.61982	2.25684
H	-0.44917	-5.39245	2.46976
C	-1.28960	-3.47793	3.06492
H	-0.60507	-3.36413	3.91374
C	-2.26891	-2.48734	2.83588
C	-4.00802	-4.00216	-0.28908
H	-4.88877	-3.36187	-0.11568
C	-3.32767	-3.49913	-1.58536
H	-2.40355	-4.07233	-1.79342
H	-3.07625	-2.42413	-1.51345
H	-3.99156	-3.62588	-2.45868
C	-4.49795	-5.45370	-0.46335
H	-4.93826	-5.84928	0.46732
H	-3.68499	-6.13751	-0.76797
H	-5.26927	-5.49813	-1.25107
C	-2.36592	-1.30994	3.80538
H	-3.11702	-0.61735	3.38818
C	-1.03278	-0.54456	3.94553

H	-0.67690	-0.16412	2.97167
H	-0.23392	-1.17593	4.37348
H	-1.16028	0.32033	4.62083
C	-2.85892	-1.77943	5.19431
H	-2.13556	-2.47076	5.66284
H	-3.82422	-2.30771	5.12352
H	-2.98936	-0.91868	5.87376
C	-6.06074	-3.57476	2.88712
H	-5.95467	-4.34958	2.11106
H	-7.07648	-3.65478	3.31008
H	-5.33798	-3.79454	3.69067
C	-6.17832	-0.53485	3.50550
H	-7.24489	-0.56112	3.78723
H	-5.94745	0.48050	3.14229
H	-5.57933	-0.71259	4.41476
C	-6.96032	-2.10483	-1.89105
H	-7.76271	-2.82453	-1.65233
H	-6.01420	-2.66327	-1.96775
H	-7.17938	-1.67119	-2.88071
C	-8.51502	0.22765	-0.67580
H	-8.73188	0.76522	0.26018
H	-9.35591	-0.45771	-0.87752
H	-8.46959	0.96339	-1.49616
C	-5.50249	1.55644	-1.20215
C	-5.14916	1.66974	-2.58769
C	-5.09919	2.94131	-3.19415
H	-4.83996	3.01448	-4.25757
C	-5.39176	4.10787	-2.47700
H	-5.35228	5.08709	-2.96698
C	-5.77412	3.99717	-1.13470
H	-6.04000	4.90271	-0.57612
C	-5.85519	2.74924	-0.48517
C	-4.87972	0.44097	-3.45875
H	-4.92674	-0.43771	-2.79404
C	-5.97067	0.29025	-4.54531
H	-5.93328	1.12656	-5.26633
H	-6.98256	0.28171	-4.10670
H	-5.83332	-0.64691	-5.11389
C	-3.48282	0.45750	-4.11723
H	-2.68179	0.49623	-3.35998
H	-3.35918	1.32383	-4.79215
H	-3.33089	-0.45318	-4.72418
C	-6.36719	2.72022	0.95715
H	-6.47202	1.65690	1.23364
C	-7.75366	3.39433	1.08505
H	-8.47889	2.98541	0.36337
H	-7.69069	4.48367	0.91186
H	-8.15890	3.24817	2.10209
C	-5.38008	3.36926	1.95408
H	-5.20042	4.42987	1.69825
H	-4.41619	2.83657	1.94891
H	-5.79264	3.34101	2.97904
C	-2.05541	0.37340	-0.31738
H	-2.17252	0.81303	-1.32495
H	-0.98732	0.09003	-0.21032
C	-2.62572	1.34638	0.80579
H	-2.78167	2.40389	0.52578
H	-2.10084	1.26387	1.77301
C	5.41361	1.27902	1.48904
C	5.03917	1.03406	2.85379
C	5.02565	2.09366	3.78338
H	4.74657	1.88378	4.82319
C	5.37690	3.39762	3.41819
H	5.36991	4.20658	4.15681
C	5.75673	3.64193	2.09404
H	6.05589	4.65562	1.80093
C	5.78895	2.61904	1.12392
C	4.66626	-0.35823	3.36277
H	4.74675	-1.04540	2.50408
C	3.21131	-0.40979	3.87947
H	2.50040	-0.13132	3.08402
H	3.06258	0.27762	4.73194
H	2.95508	-1.42658	4.22769
C	5.63449	-0.83606	4.46980
H	5.53640	-0.22091	5.38233
H	6.68667	-0.77488	4.14600
H	5.42207	-1.88278	4.75178
C	6.24846	3.00699	-0.28382
H	6.25057	2.08361	-0.88999
C	5.29058	4.02377	-0.94792
H	5.27627	4.97528	-0.38537
H	4.25990	3.64020	-1.00006
H	5.62014	4.25060	-1.97696
C	7.67951	3.59615	-0.28558
H	8.40282	2.92955	0.20795
H	7.71145	4.56877	0.23777
H	8.02197	3.76953	-1.32126
C	7.00222	-2.31392	1.37569
H	7.81382	-2.95776	0.99341
H	6.08158	-2.91752	1.42334
H	7.26569	-2.02172	2.40587
C	8.47199	0.15296	0.36609
H	8.61277	0.81258	-0.50386
H	9.30970	-0.56485	0.39024
H	8.52091	0.76369	1.28306
C	5.88415	-4.07501	-2.14506
H	6.03414	-4.40677	-1.10573
H	6.79816	-4.31884	-2.71344
H	5.05259	-4.65679	-2.57799
C	5.86851	-1.70185	-4.05993
H	6.91802	-1.92684	-4.31898
H	5.70071	-0.62651	-4.22442
H	5.22457	-2.26614	-4.75528
C	2.87457	-2.64547	-1.75178
C	1.99640	-2.50333	-2.88129
C	0.92735	-3.40689	-3.06202
H	0.27611	-3.29051	-3.93634
C	0.70631	-4.47310	-2.17924
H	-0.09591	-5.19573	-2.36622
C	1.55496	-4.61714	-1.06963
H	1.39975	-5.45539	-0.38041
C	2.61989	-3.72321	-0.83088
C	2.19346	-1.39826	-3.91796
H	3.14254	-0.89687	-3.66810
C	2.30598	-1.95501	-5.35480
H	1.35439	-2.39928	-5.69807
H	3.08185	-2.73482	-5.42906
H	2.56492	-1.14671	-6.06036
C	1.06637	-0.34316	-3.84331
H	1.00318	0.10642	-2.83782
H	0.08360	-0.78884	-4.08284
H	1.25166	0.46875	-4.56894
C	3.46456	-3.90413	0.42777
H	4.38295	-3.31612	0.26796
C	3.86074	-5.36764	0.70966
H	4.33367	-5.83902	-0.16725
H	2.99117	-5.98586	0.99782
H	4.57627	-5.41076	1.54923
C	2.74457	-3.29011	1.65011
H	1.79419	-3.82026	1.85703
H	2.53906	-2.21732	1.48141
H	3.36822	-3.37358	2.55762
C	2.55345	1.42290	-0.67710
H	2.94995	2.23347	-0.04267
H	2.03584	1.81028	-1.57193
C	1.99832	0.20321	0.10264
H	1.60794	0.36918	1.12639
H	1.26304	-0.34815	-0.51268
C	-1.50555	3.83191	-2.72132
H	-2.37952	3.21156	-2.94632
C	-1.66886	4.99996	-1.95254
H	-2.66785	5.26916	-1.59382

```
C  -0.55883  5.81879 -1.67446
H  -0.68897  6.73916 -1.09522
C   0.71567  5.46675 -2.15680
H   1.57790  6.10942 -1.95020
C   0.88179  4.29625 -2.92006
H   1.87189  4.02159 -3.29744
C  -0.23106  3.48195 -3.20484
H  -0.10456  2.57953 -3.81189
C   0.19022  3.35000  3.42698
H  -0.11078  2.48072  4.02057
C  -0.76624  4.31859  3.06665
H  -1.80960  4.20111  3.37655
C  -0.37555  5.44578  2.32056
H  -1.11567  6.20849  2.05632
C   0.96840  5.60137  1.93150
H   1.27422  6.48678  1.36432
C   1.92263  4.63069  2.28735
H   2.97360  4.74643  2.00321
C   1.53373  3.50467  3.03779
H   2.28894  2.76553  3.32509
C   4.52655  1.02603 -2.40311
O   4.30851  1.81022 -3.24624
```

TS(A-B)

```
SCF (BP86) Energy = -3370.73060251
Enthalpy 0K = -3368.977944
Enthalpy 298K = -3368.977000
Free Energy 298K = -3369.255273
Lowest Frequency = -167.5295 cm-1
Second Frequency = 8.5509 cm-1
SCF (BP86-D3BJ) Energy =  -3371.31425741
SCF (C6H6) Energy =  -3370.74088455
SCF (6-311++G**) Energy =  -6138.21783406
```

```
K  -0.08693  2.79412  0.10809
K  -0.28954 -2.37458 -0.00810
Si -5.76459 -1.88373  2.17825
Si -6.87287 -0.83748 -0.54056
Si  6.81388 -0.72646  0.26001
Si  5.59021 -2.21903 -2.22469
Al -3.85353 -0.14109  0.40306
Al  3.87895 -0.15136 -0.60415
O  -6.89879 -1.69329  0.93233
O   6.71233 -1.31809 -1.33060
N  -4.13632 -1.66737  1.49242
N  -5.45752  0.23215 -0.54235
N   5.36469  0.26848  0.50621
N   4.00000 -1.84416 -1.51022
C  -3.12199 -2.62935  1.74944
C  -3.00051 -3.80735  0.92993
C  -1.99998 -4.75907  1.21705
H  -1.93354 -5.66476  0.60380
C  -1.10279 -4.58514  2.28322
H  -0.34687 -5.34634  2.50411
C  -1.21026 -3.43577  3.07888
H  -0.52619 -3.30377  3.92550
C  -2.20343 -2.46127  2.84108
C  -3.92183 -4.02729 -0.26954
H  -4.81547 -3.40453 -0.09816
C  -3.25025 -3.51195 -1.56562
H  -2.31546 -4.06791 -1.77281
H  -3.01906 -2.43239 -1.49347
H  -3.91093 -3.65129 -2.43946
C  -4.38249 -5.48837 -0.44289
H  -4.81524 -5.89203  0.48786
H  -3.55596 -6.15607 -0.74677
H  -5.15239 -5.54880 -1.23091
C  -2.31390 -1.27359  3.79643
H  -3.07948 -0.59919  3.37571
C  -0.99262 -0.48452  3.91536
H  -0.65122 -0.11228  2.93315
H  -0.18009 -1.09692  4.34517
H  -1.12794  0.38805  4.57913
C  -2.78934 -1.73262  5.19481
H  -2.05172 -2.40580  5.66756
H  -3.74631 -2.27748  5.13742
H  -2.92951 -0.86526  5.86383
C  -5.98156 -3.62121  2.89730
H  -5.86067 -4.39829  2.12572
H  -6.99618 -3.71710  3.31965
H  -5.25589 -3.82340  3.70290
C  -6.15316 -0.58237  3.50313
H  -7.21914 -0.62686  3.78480
H  -5.94052  0.43564  3.13626
H  -5.55117 -0.74592  4.41312
C  -6.89844 -2.17636 -1.89160
H  -7.69022 -2.90865 -1.65577
H  -5.94317 -2.71955 -1.96393
H  -7.12022 -1.74673 -2.88244
C  -8.49585  0.12934 -0.68063
H  -8.72344  0.66441  0.25424
H  -9.32441 -0.57080 -0.88261
H  -8.46193  0.86452 -1.50205
C  -5.50480  1.50893 -1.20519
C  -5.14850  1.62513 -2.58962
C  -5.11783  2.89602 -3.19891
H  -4.85621  2.97106 -4.26162
C  -5.43127  4.05919 -2.48511
H  -5.40580  5.03796 -2.97693
C  -5.81609  3.94505 -1.14389
H  -6.09774  4.84741 -0.58792
C  -5.87953  2.69736 -0.49203
C  -4.85276  0.39922 -3.45602
H  -4.89246 -0.47883 -2.79004
C  -5.93065  0.22922 -4.55261
H  -5.89990  1.06444 -5.27521
H  -6.94639  0.20524 -4.12350
H  -5.77292 -0.70674 -5.11783
C  -3.44961  0.43655 -4.10023
H  -2.65771  0.48751 -3.33415
H  -3.33193  1.30472 -4.77383
H  -3.27756 -0.47190 -4.70519
C  -6.39625  2.66331  0.94851
H  -6.48343  1.59915  1.22778
C  -7.79475  3.31352  1.06920
H  -8.51001  2.88982  0.34613
H  -7.75004  4.40324  0.89272
H  -8.20123  3.16348  2.08519
C  -5.42459  3.33228  1.94748
H  -5.26228  4.39510  1.68919
H  -4.45183  2.81598  1.94790
H  -5.84086  3.29985  2.97082
C  -2.03216  0.38227 -0.29157
H  -2.11246  0.81227 -1.30669
H  -0.96971  0.10144 -0.13799
C  -2.64032  1.36073  0.80976
H  -2.82791  2.40518  0.50237
H  -2.12088  1.31748  1.78217
C   5.34458  1.33725  1.46659
C   4.94991  1.09727  2.82665
C   4.90770  2.16264  3.74874
H   4.61406  1.95585  4.78520
C   5.25242  3.46765  3.38157
H   5.22531  4.28107  4.11483
C   5.65347  3.70694  2.06266
H   5.94789  4.72156  1.76818
C   5.71247  2.67906  1.09917
C   4.59063 -0.29638  3.34170
H   4.69441 -0.98960  2.49037
C   3.13059 -0.36766  3.84114
H   2.42271 -0.09772  3.03986
H   2.96265  0.31812  4.69127
```

```
H   2.88431 -1.38765  4.18715
C   5.55044 -0.75040  4.46618
H   5.42790 -0.13090  5.37275
H   6.60600 -0.67286  4.15767
H   5.35203 -1.79879  4.75214
C   6.19052  3.06576 -0.30295
H   6.19818  2.14408 -0.91074
C   5.24661  4.08835 -0.97874
H   5.23944  5.04343 -0.42201
H   4.21277  3.71575 -1.03717
H   5.58587  4.30435 -2.00673
C   7.62266  3.65292 -0.28399
H   8.33931  2.98618  0.21860
H   7.64705  4.62481  0.24118
H   7.97912  3.82768 -1.31465
C   6.98508 -2.22634  1.42095
H   7.80132 -2.87203  1.05184
H   6.06793 -2.83433  1.47852
H   7.24588 -1.91332  2.44561
C   8.43919  0.23397  0.37817
H   8.57872  0.88479 -0.49844
H   9.28190 -0.47755  0.41383
H   8.47964  0.85450  1.28890
C   5.98859 -4.07308 -2.12134
H   6.13337 -4.40568 -1.08123
H   6.91935 -4.27974 -2.67702
H   5.18548 -4.68262 -2.57002
C   5.85657 -1.66888 -4.01986
H   6.91493 -1.82963 -4.29016
H   5.63007 -0.59913 -4.15092
H   5.23867 -2.24709 -4.72689
C   2.90837 -2.73570 -1.70094
C   2.02138 -2.60318 -2.82600
C   0.95379 -3.51152 -2.99167
H   0.29583 -3.40366 -3.86217
C   0.74084 -4.57153 -2.09866
H  -0.06307 -5.29528 -2.27302
C   1.59891 -4.70720 -0.99522
H   1.45014 -5.54034 -0.29860
C   2.66412 -3.80988 -0.77304
C   2.21318 -1.51134 -3.87831
H   3.15641 -0.99650 -3.63245
C   2.33996 -2.09452 -5.30386
H   1.39349 -2.55167 -5.64447
H   3.12089 -2.87098 -5.35725
H   2.59894 -1.29828 -6.02294
C   1.07765 -0.46317 -3.83455
H   1.00799  0.01740 -2.84377
H   0.09841 -0.92210 -4.06339
H   1.25832  0.32939 -4.58217
C   3.52822 -3.97810  0.47363
H   4.44305 -3.39110  0.29062
C   3.93094 -5.43802  0.76247
H   4.39837 -5.91176 -0.11632
H   3.06645 -6.05842  1.06100
H   4.65423 -5.47368  1.59569
C   2.82859 -3.34993  1.70020
H   1.87673 -3.86969  1.92364
H   2.62324 -2.27803  1.52751
H   3.46366 -3.42660  2.60050
C   2.52240  1.53340 -0.68064
H   2.94701  2.29706 -0.00920
H   1.97103  1.97067 -1.52960
C   2.01222  0.25130  0.01464
H   1.64434  0.34299  1.05617
H   1.25487 -0.25427 -0.61327
C  -1.53887  3.84643 -2.75132
H  -2.44118  3.24890 -2.92023
C  -1.63727  5.06325 -2.04996
H  -2.61537  5.39485 -1.68589
C  -0.48976  5.85198 -1.84625
H  -0.56993  6.80965 -1.32075
C   0.75763  5.42115 -2.33491
H   1.64942  6.03853 -2.18455
C   0.85965  4.20226 -3.03080
H   1.82987  3.86481 -3.40913
C  -0.29093  3.41846 -3.24166
H  -0.21493  2.47781 -3.79712
C   0.04051  3.53751  3.43195
H  -0.29520  2.70777  4.06249
C  -0.88300  4.50965  3.00196
H  -1.93512  4.43393  3.29471
C  -0.44811  5.58690  2.20801
H  -1.16264  6.35321  1.88929
C   0.90707  5.68893  1.84039
H   1.24702  6.53543  1.23463
C   1.82854  4.71436  2.26564
H   2.88746  4.78819  1.99728
C   1.39506  3.63889  3.06434
H   2.12522  2.89673  3.40378
C   4.25270  1.05576 -2.26803
O   4.18142  1.85577 -3.13413
```

B

SCF (BP86) Energy = -3370.78613482
Enthalpy 0K = -3369.030624
Enthalpy 298K = -3369.029680
Free Energy 298K = -3369.311119
Lowest Frequency = 7.3393 cm-1
Second Frequency = 9.3626 cm-1
SCF (BP86-D3BJ) Energy = -3371.35915721
SCF (C6H6) Energy = -3370.79751501
SCF (6-311++G**) Energy = -6138.27020511

```
K  -0.31316  3.00036  0.16426
K  -0.29897 -2.48873  0.05958
Si -5.98099 -1.93145  2.12492
Si -6.95606 -1.04052 -0.69715
Si  7.17057 -0.64904  0.16235
Si  5.75485 -1.85829 -2.33825
Al -4.01935 -0.20353  0.38421
Al  4.00832 -0.49493 -0.03601
O  -7.03248 -1.85728  0.79445
O   7.05854 -1.47115 -1.32445
N  -4.32507 -1.68202  1.53488
N  -5.57256  0.07358 -0.66498
N   5.68268  0.28587  0.38280
N   4.27193 -1.85769 -1.35586
C  -3.27928 -2.58131  1.87804
C  -3.06551 -3.78775  1.12297
C  -2.02087 -4.66481  1.48566
H  -1.88608 -5.59666  0.92378
C  -1.16361 -4.38287  2.56220
H  -0.36879 -5.08368  2.83976
C  -1.36489 -3.20559  3.29751
H  -0.71397 -2.99119  4.15340
C  -2.40924 -2.30880  2.98800
C  -3.92974 -4.12017 -0.09354
H  -4.84224 -3.50676 -0.00969
C  -3.21394 -3.69191 -1.39777
H  -2.26950 -4.25565 -1.53194
H  -2.99577 -2.60722 -1.39383
H  -3.83906 -3.89994 -2.28396
C  -4.35171 -5.60132 -0.17093
H  -4.82228 -5.93915  0.76745
H  -3.49667 -6.27050 -0.37697
H  -5.07913 -5.74471 -0.98814
C  -2.63200 -1.09200  3.88603
H  -3.41273 -0.48126  3.40110
C  -1.36942 -0.21725  4.03755
H  -1.00380  0.13553  3.05778
H  -0.54440 -0.76199  4.53082
```

H	-1.59424	0.66862	4.65810
C	-3.15381	-1.52721	5.27567
H	-2.40670	-2.14232	5.80889
H	-4.07650	-2.12546	5.19172
H	-3.37406	-0.64601	5.90394
C	-6.18583	-3.63328	2.92742
H	-5.99966	-4.44655	2.20800
H	-7.21814	-3.74077	3.30129
H	-5.49852	-3.76647	3.77973
C	-6.50475	-0.57389	3.34256
H	-7.58411	-0.64452	3.56068
H	-6.30576	0.42928	2.92957
H	-5.95579	-0.66112	4.29574
C	-6.86880	-2.40938	-2.01463
H	-7.66998	-3.14467	-1.82438
H	-5.90590	-2.94415	-2.00029
H	-7.01671	-2.00367	-3.02939
C	-8.59796	-0.12925	-0.94698
H	-8.88221	0.43621	-0.04610
H	-9.39531	-0.86360	-1.15329
H	-8.55035	0.57146	-1.79712
C	-5.63006	1.29660	-1.42220
C	-5.16799	1.33200	-2.77882
C	-5.17619	2.54981	-3.48856
H	-4.82866	2.56257	-4.52888
C	-5.63349	3.73604	-2.90222
H	-5.64145	4.67169	-3.47216
C	-6.10560	3.70047	-1.58426
H	-6.48700	4.62094	-1.12622
C	-6.12607	2.50800	-0.83354
C	-4.67374	0.07603	-3.49816
H	-4.80412	-0.76441	-2.79651
C	-5.50298	-0.21967	-4.76846
H	-5.36483	0.56398	-5.53483
H	-6.58190	-0.27400	-4.54558
H	-5.19642	-1.18013	-5.21959
C	-3.17113	0.16155	-3.84614
H	-2.56279	0.29499	-2.93610
H	-2.96251	1.00402	-4.53080
H	-2.83462	-0.76242	-4.35089
C	-6.69589	2.55849	0.58583
H	-6.76550	1.51438	0.93644
C	-8.11105	3.17977	0.62682
H	-8.79635	2.69151	-0.08463
H	-8.08926	4.25699	0.38239
H	-8.54091	3.08520	1.63976
C	-5.76232	3.31654	1.55817
H	-5.62253	4.36404	1.23252
H	-4.77664	2.82718	1.61059
H	-6.19745	3.33926	2.57394
C	-2.07609	0.17911	0.02611
H	-1.47154	0.40880	-0.87432
H	-1.43085	-0.29174	0.79334
C	-2.86555	1.37213	0.71793
H	-3.12832	2.18853	0.02153
H	-2.46711	1.76661	1.67038
C	5.67636	1.60670	0.94763
C	5.55708	1.80516	2.36365
C	5.50152	3.11461	2.88370
H	5.42903	3.25314	3.96938
C	5.56057	4.23885	2.05095
H	5.52935	5.24848	2.47599
C	5.69418	4.04944	0.67030
H	5.76342	4.92338	0.01131
C	5.76331	2.76290	0.10133
C	5.54186	0.63433	3.34746
H	5.47196	-0.28848	2.74685
C	4.33031	0.67178	4.30435
H	3.37776	0.70446	3.74825
H	4.36226	1.55188	4.97170
H	4.31641	-0.22554	4.94840
C	6.85551	0.58412	4.16293
H	6.96165	1.48202	4.79809
H	7.73982	0.54101	3.50536
H	6.87583	-0.30009	4.82510
C	5.95187	2.64929	-1.41099
H	6.07087	1.57521	-1.63158
C	4.71983	3.16379	-2.19143
H	4.57297	4.24610	-2.01410
H	3.79417	2.64294	-1.89595
H	4.86996	3.02771	-3.27834
C	7.22439	3.38375	-1.89199
H	8.12044	3.05858	-1.33854
H	7.13352	4.47741	-1.76221
H	7.39527	3.19374	-2.96645
C	7.42749	-2.00383	1.47184
H	8.24097	-2.67991	1.15630
H	6.51711	-2.61053	1.61086
H	7.70084	-1.57686	2.45118
C	8.70797	0.45443	0.11082
H	8.75373	1.02152	-0.83209
H	9.61751	-0.16614	0.17989
H	8.71860	1.17350	0.94704
C	6.09516	-3.56581	-3.08616
H	6.42886	-4.27559	-2.31301
H	6.89563	-3.48703	-3.84137
H	5.20166	-3.98457	-3.57937
C	5.75395	-0.57183	-3.73657
H	6.77841	-0.45617	-4.13070
H	5.41136	0.41499	-3.38521
H	5.10314	-0.88028	-4.57180
C	3.24111	-2.82615	-1.52407
C	2.17696	-2.62315	-2.47184
C	1.13605	-3.57209	-2.57625
H	0.35320	-3.42184	-3.33050
C	1.10138	-4.71917	-1.76753
H	0.30121	-5.45982	-1.88198
C	2.14420	-4.92958	-0.85215
H	2.14570	-5.84138	-0.24286
C	3.21897	-4.02584	-0.72787
C	2.17518	-1.43645	-3.43624
H	2.99956	-0.77273	-3.12689
C	2.44722	-1.91254	-4.88326
H	1.62820	-2.55661	-5.25148
H	3.38088	-2.49534	-4.95065
H	2.52990	-1.05076	-5.56878
C	0.86686	-0.61829	-3.38755
H	0.68433	-0.19826	-2.38394
H	-0.01047	-1.22173	-3.68350
H	0.92487	0.23318	-4.08792
C	4.36439	-4.38678	0.21920
H	5.13350	-3.60670	0.08921
C	4.99218	-5.75255	-0.14539
H	5.27933	-5.80066	-1.20812
H	4.29279	-6.58576	0.04895
H	5.89506	-5.93262	0.46383
C	3.93615	-4.38901	1.70419
H	3.14066	-5.13444	1.88937
H	3.56509	-3.40193	2.02136
H	4.79176	-4.65483	2.34995
C	1.70366	0.22185	0.99489
H	1.74552	0.98645	1.80103
H	0.61699	0.05803	0.80534
C	2.58599	-1.00072	1.36109
H	2.82943	-1.05881	2.43264
H	2.12579	-1.97011	1.08933
C	-1.77320	3.98191	-2.69603
H	-2.48353	3.21932	-3.03225
C	-2.23501	5.05036	-1.90365
H	-3.29631	5.09916	-1.63848
C	-1.33905	6.04904	-1.48063
H	-1.70146	6.89166	-0.88179

```
C   0.01903  5.97698 -1.84467
H   0.71431  6.76184 -1.52817
C   0.48240  4.90630 -2.63064
H   1.53820  4.84749 -2.91268
C  -0.41539  3.91022 -3.05857
H  -0.05467  3.08211 -3.67685
C   0.53694  3.37657  3.51187
H   0.56815  2.43922  4.07691
C  -0.66906  4.09680  3.41472
H  -1.57422  3.71976  3.90187
C  -0.70601  5.31270  2.70665
H  -1.63951  5.88232  2.64718
C   0.46193  5.80474  2.09346
H   0.43636  6.75867  1.55666
C   1.66481  5.08046  2.18533
H   2.58039  5.45728  1.71823
C   1.70406  3.86709  2.89699
H   2.65057  3.32107  2.97580
C   2.39250  0.86320 -0.25321
O   1.84345  1.78467 -0.88498
```

TS(B-C)

SCF (BP86) Energy = -3370.75382961
Enthalpy 0K = -3369.002808
Enthalpy 298K = -3369.001864
Free Energy 298K = -3369.280062
Lowest Frequency = -870.9324 cm-1
Second Frequency = 7.7054 cm-1
SCF (BP86-D3BJ) Energy = -3371.32568912
SCF (C6H6) Energy = -3370.76590523
SCF (6-311++G**) Energy = -6138.23725822

```
K  -0.07318  3.40053 -0.10414
K   0.44723 -2.81037 -0.25423
Si -5.78707 -2.26956  1.97738
Si -6.82057 -0.96225 -0.67296
Si  6.67432 -0.47168  0.76619
Si  5.87513 -1.82049 -1.89481
Al -3.86004 -0.39927  0.38672
Al  3.57598 -0.47232 -0.02857
O  -6.88736 -1.90462  0.73876
O   6.95470 -1.29431 -0.70130
N  -4.14382 -1.98561  1.34060
N  -5.38357  0.09735 -0.56200
N   5.14900  0.41467  0.64278
N   4.24227 -1.88359 -1.18823
C  -3.09023 -2.92604  1.59659
C  -2.86416 -4.01882  0.69197
C  -1.80491 -4.91630  0.93947
H  -1.65208 -5.76075  0.25683
C  -0.96777 -4.77482  2.05765
H  -0.16599 -5.49688  2.24808
C  -1.19607 -3.71582  2.94573
H  -0.55597 -3.60861  3.82871
C  -2.24239 -2.79177  2.74353
C  -3.73524 -4.23204 -0.54674
H  -4.61489 -3.57706 -0.43025
C  -2.99655 -3.79149 -1.83313
H  -2.09449 -4.41010 -2.00088
H  -2.69102 -2.73124 -1.77794
H  -3.64346 -3.90997 -2.72053
C  -4.23560 -5.68555 -0.69083
H  -4.73094 -6.03993  0.22829
H  -3.41157 -6.38607 -0.91614
H  -4.95991 -5.75805 -1.52042
C  -2.45323 -1.69020  3.78185
H  -3.24186 -1.02577  3.38872
C  -1.18318 -0.84163  4.01117
H  -0.79167 -0.42708  3.06723
H  -0.37372 -1.43479  4.47211
H  -1.40420 -0.00451  4.69742
C  -2.95022 -2.28396  5.12120
H  -2.18973 -2.94997  5.56658
H  -3.87127 -2.87679  4.98959
H  -3.15951 -1.48145  5.85049
C  -6.02967 -4.07255  2.48660
H  -5.97204 -4.74708  1.61798
H  -7.02559 -4.19252  2.94557
H  -5.27282 -4.39201  3.22212
C  -6.21340 -1.12472  3.42939
H  -7.28541 -1.21306  3.67603
H  -6.01119 -0.06810  3.18465
H  -5.63256 -1.38013  4.33121
C  -6.78126 -2.19172 -2.11759
H  -7.58711 -2.93376 -1.98300
H  -5.82357 -2.73502 -2.16748
H  -6.93986 -1.69075 -3.08653
C  -8.40725  0.06089 -0.77818
H  -8.57318  0.63988  0.14401
H  -9.26948 -0.61319 -0.91740
H  -8.38069  0.76360 -1.62735
C  -5.44118  1.42437 -1.12540
C  -5.12538  1.64648 -2.50333
C  -5.18086  2.95580 -3.02324
H  -4.95350  3.11662 -4.08388
C  -5.53351  4.04885 -2.22293
H  -5.58733  5.05600 -2.65092
C  -5.84168  3.83181 -0.87430
H  -6.13052  4.68206 -0.24582
C  -5.81374  2.54201 -0.30919
C  -4.72828  0.50714 -3.44271
H  -4.77592 -0.42573 -2.85663
C  -5.70337  0.38065 -4.63550
H  -5.65509  1.26770 -5.29195
H  -6.74896  0.27858 -4.29912
H  -5.45249 -0.50040 -5.25208
C  -3.27788  0.66233 -3.95308
H  -2.56088  0.71394 -3.11622
H  -3.16078  1.57767 -4.56136
H  -2.99469 -0.19418 -4.59016
C  -6.17375  2.37924  1.16826
H  -6.32221  1.29934  1.34277
C  -7.48137  3.10320  1.55588
H  -8.31739  2.81271  0.89833
H  -7.37781  4.20130  1.49806
H  -7.76031  2.85738  2.59535
C  -5.01255  2.84425  2.07912
H  -4.80888  3.92032  1.92908
H  -4.08525  2.28711  1.85713
H  -5.26273  2.69239  3.14472
C  -1.43805 -0.48387 -0.30182
H  -1.50875 -0.42829 -1.40374
H  -1.74810 -1.49646  0.03658
C  -2.22106  0.64702  0.42587
H  -2.32130  1.54680 -0.20945
H  -1.75641  0.94103  1.38826
C   5.07797  1.77650  1.08644
C   4.87476  2.10013  2.47127
C   4.82211  3.44971  2.87672
H   4.69719  3.67980  3.94229
C   4.95081  4.49988  1.95891
H   4.93227  5.54234  2.29817
C   5.14778  4.19095  0.60763
H   5.27521  5.00367 -0.11824
C   5.22580  2.85989  0.15436
C   4.76128  1.02177  3.55103
H   4.71654  0.05132  3.02799
C   3.47899  1.16360  4.40120
H   2.57408  1.15221  3.76992
H   3.47779  2.10196  4.98532
H   3.39745  0.33043  5.12214
C   6.00017  1.02049  4.47735
```

H	6.08094	1.97326	5.03135
H	6.93581	0.89012	3.90905
H	5.93497	0.20585	5.22128
C	5.52606	2.61849	-1.32377
H	5.57704	1.52476	-1.45164
C	4.41404	3.15881	-2.25132
H	4.33678	4.26027	-2.16653
H	3.43251	2.71511	-2.00697
H	4.65585	2.93150	-3.30650
C	6.89525	3.21057	-1.73063
H	7.70934	2.84460	-1.08377
H	6.89096	4.31410	-1.66652
H	7.13797	2.93993	-2.77366
C	6.66949	-1.81905	2.11100
H	7.51755	-2.50890	1.95865
H	5.73829	-2.40856	2.08247
H	6.76085	-1.38475	3.12058
C	8.16111	0.67304	1.03840
H	8.42615	1.19100	0.10290
H	9.03577	0.08010	1.35628
H	7.96261	1.43681	1.80790
C	6.44992	-3.53377	-2.47496
H	6.62747	-4.20952	-1.62371
H	7.40037	-3.42879	-3.02597
H	5.71799	-4.00908	-3.14977
C	6.06063	-0.63380	-3.36947
H	7.13392	-0.46414	-3.56395
H	5.59049	0.34468	-3.18265
H	5.61455	-1.05676	-4.28517
C	3.36245	-2.92983	-1.57552
C	2.48193	-2.79001	-2.70900
C	1.58093	-3.82863	-3.03372
H	0.92913	-3.71034	-3.90796
C	1.51993	-5.01454	-2.28685
H	0.82903	-5.81716	-2.56826
C	2.39266	-5.17106	-1.19786
H	2.37723	-6.10877	-0.62962
C	3.31514	-4.16745	-0.83403
C	2.52878	-1.56320	-3.62109
H	3.27038	-0.87279	-3.18696
C	2.99470	-1.95772	-5.04258
H	2.25226	-2.60465	-5.54447
H	3.95027	-2.50786	-5.02096
H	3.13005	-1.05847	-5.66875
C	1.17921	-0.81536	-3.69102
H	0.88605	-0.41399	-2.70715
H	0.37072	-1.46743	-4.07137
H	1.25854	0.04412	-4.37953
C	4.25982	-4.43210	0.33903
H	5.02065	-3.63449	0.30788
C	4.97561	-5.79631	0.22864
H	5.46521	-5.92364	-0.75069
H	4.27723	-6.64180	0.36641
H	5.74780	-5.88279	1.01265
C	3.53116	-4.32309	1.69822
H	2.71997	-5.07242	1.77350
H	3.09963	-3.31808	1.83858
H	4.23104	-4.51077	2.53180
C	1.20258	0.23492	0.66069
H	0.68333	0.97584	1.31891
H	0.07588	-0.23538	0.11556
C	2.09567	-0.81063	1.37831
H	2.47554	-0.43479	2.34713
H	1.58228	-1.75212	1.66420
C	-1.62870	4.33736	-2.87649
H	-2.42432	3.61820	-3.09862
C	-1.93353	5.50240	-2.14722
H	-2.96300	5.67594	-1.81635
C	-0.92235	6.43832	-1.86272
H	-1.16224	7.35392	-1.31159
C	0.39484	6.20537	-2.30119
H	1.18145	6.93643	-2.08605
C	0.70095	5.03928	-3.02606
H	1.72641	4.85163	-3.35850
C	-0.31208	4.10664	-3.31689
H	-0.07048	3.19907	-3.87821
C	-0.08310	3.62925	3.25099
H	-0.15558	2.64856	3.73241
C	-1.24277	4.39979	3.04224
H	-2.21879	4.01991	3.36165
C	-1.14613	5.66507	2.43377
H	-2.04651	6.27075	2.28531
C	0.10983	6.15983	2.03421
H	0.18634	7.15115	1.57510
C	1.26804	5.38743	2.24027
H	2.25190	5.75964	1.93619
C	1.17282	4.12216	2.84943
H	2.08178	3.53116	3.00353
C	1.99697	0.81452	-0.44944
O	1.62440	1.81430	-1.14012

C

SCF (BP86) Energy = -3370.81670726
Enthalpy 0K = -3369.058911
Enthalpy 298K = -3369.057967
Free Energy 298K = -3369.341151
Lowest Frequency = 5.8564 cm-1
Second Frequency = 6.4569 cm-1
SCF (BP86-D3BJ) Energy = -3371.37783192
SCF (C6H6) Energy = -3370.82787835
SCF (6-311++G**) Energy = -6138.30369676

K	-1.99092	3.08149	-0.56130
K	-0.87145	-2.42155	0.10081
Si	6.20151	-2.69159	-1.64391
Si	7.67682	-0.71973	0.32138
Si	-7.09167	-0.03651	-0.91016
Si	-6.48666	-1.97143	1.44665
Al	4.52524	-0.53385	-0.12152
Al	-4.03001	-0.61322	-0.27405
O	7.40105	-2.12417	-0.58982
O	-7.45990	-1.09592	0.36844
N	4.63404	-2.18054	-0.94674
N	6.15089	0.22130	0.30049
N	-5.47344	0.62054	-0.64901
N	-4.84259	-2.03826	0.78770
C	3.49057	-3.05380	-0.94596
C	3.27602	-3.93298	0.16618
C	2.13200	-4.75363	0.18179
H	1.96940	-5.43036	1.02742
C	1.21358	-4.75018	-0.87944
H	0.34670	-5.42062	-0.86191
C	1.43742	-3.90487	-1.97521
H	0.73739	-3.91953	-2.81937
C	2.56184	-3.05371	-2.03301
C	4.26437	-3.98888	1.33371
H	5.22356	-3.59056	0.96034
C	3.81576	-3.07231	2.49486
H	2.83632	-3.38882	2.89584
H	3.70876	-2.01956	2.17364
H	4.54677	-3.09724	3.32182
C	4.52026	-5.42039	1.84978
H	4.80286	-6.10269	1.03062
H	3.63489	-5.84770	2.35299
H	5.33940	-5.41468	2.58912
C	2.76088	-2.18436	-3.27420
H	3.65018	-1.55795	-3.08841
C	1.56221	-1.24521	-3.53454
H	1.33847	-0.61122	-2.65961
H	0.64392	-1.80939	-3.77521
H	1.77144	-0.58197	-4.39206
C	3.04223	-3.05358	-4.52249

H	2.17275	-3.68749	-4.77132
H	3.90504	-3.72354	-4.36791
H	3.25268	-2.41784	-5.40041
C	6.32933	-4.57224	-1.74345
H	6.29987	-5.03521	-0.74455
H	7.28316	-4.85153	-2.22191
H	5.50886	-4.99809	-2.34477
C	6.52710	-1.92046	-3.34467
H	7.55980	-2.14304	-3.66390
H	6.41038	-0.82379	-3.31919
H	5.84129	-2.31713	-4.11139
C	8.18424	-1.35107	2.03245
H	9.04742	-2.02977	1.91978
H	7.36927	-1.91769	2.51237
H	8.48206	-0.53355	2.70865
C	9.10317	0.27065	-0.42719
H	8.95598	0.44751	-1.50438
H	10.04723	-0.28517	-0.29737
H	9.21154	1.24833	0.07159
C	6.17209	1.64345	0.58094
C	6.05603	2.12931	1.91962
C	6.03841	3.52123	2.14400
H	5.94801	3.89155	3.17156
C	6.13414	4.43583	1.09008
H	6.12246	5.51309	1.28715
C	6.25130	3.95714	-0.22007
H	6.32508	4.67093	-1.04783
C	6.27842	2.57752	-0.49910
C	5.91410	1.19613	3.12268
H	6.05943	0.16654	2.75625
C	6.98031	1.47164	4.20662
H	6.83936	2.46272	4.67269
H	8.00215	1.44560	3.79174
H	6.91635	0.71883	5.01153
C	4.49599	1.27974	3.73273
H	3.72078	1.03433	2.98746
H	4.28006	2.29498	4.10910
H	4.39107	0.57842	4.57928
C	6.37436	2.11797	-1.95531
H	6.64874	1.04905	-1.93790
C	7.45691	2.86631	-2.76165
H	8.43924	2.81480	-2.26390
H	7.20622	3.93246	-2.90096
H	7.55778	2.42353	-3.76799
C	5.00230	2.23338	-2.66025
H	4.66036	3.28318	-2.68395
H	4.22278	1.64965	-2.13729
H	5.05890	1.86927	-3.70153
C	1.72992	-0.27129	1.03250
H	2.02413	-0.24918	2.09660
H	1.61979	-1.34143	0.77201
C	2.79874	0.39919	0.13357
H	3.01778	1.42005	0.50013
H	2.37444	0.53978	-0.88147
C	-5.21279	2.00987	-0.76493
C	-4.96134	2.62407	-2.04219
C	-4.71856	4.01218	-2.12037
H	-4.55626	4.46693	-3.10546
C	-4.69768	4.82468	-0.97642
H	-4.53267	5.90469	-1.06046
C	-4.91905	4.22970	0.27556
H	-4.91802	4.85512	1.17695
C	-5.17804	2.84960	0.40469
C	-4.95349	1.80869	-3.33557
H	-5.09923	0.75557	-3.04226
C	-3.59572	1.90369	-4.06740
H	-2.77519	1.54823	-3.41937
H	-3.37539	2.94035	-4.38376
H	-3.60032	1.27608	-4.97623
C	-6.10600	2.21879	-4.27980
H	-6.00336	3.26882	-4.60950
H	-7.08849	2.12086	-3.78849
H	-6.11259	1.58507	-5.18455
C	-5.42863	2.27455	1.79545
H	-5.68655	1.21423	1.63955
C	-4.15911	2.33044	2.67524
H	-3.87859	3.38132	2.88482
H	-3.29906	1.82413	2.19706
H	-4.34509	1.84692	3.65143
C	-6.61810	2.95830	2.50468
H	-7.53521	2.91719	1.89345
H	-6.40775	4.02164	2.72185
H	-6.82861	2.46063	3.46764
C	-7.22520	-1.08302	-2.49138
H	-8.16810	-1.65642	-2.49350
H	-6.38819	-1.79938	-2.55085
H	-7.20122	-0.45938	-3.40107
C	-8.41358	1.32188	-0.90865
H	-8.45056	1.83339	0.06687
H	-9.40590	0.87886	-1.09888
H	-8.22287	2.08285	-1.68370
C	-7.26192	-3.69362	1.63980
H	-7.51208	-4.13426	0.66238
H	-8.19479	-3.61120	2.22380
H	-6.58939	-4.38595	2.17479
C	-6.68616	-1.11677	3.13773
H	-7.76250	-0.94809	3.31773
H	-6.17802	-0.14103	3.18992
H	-6.30297	-1.74547	3.95877
C	-3.95141	-3.10533	1.08929
C	-3.09501	-3.05876	2.25048
C	-2.13636	-4.07493	2.45978
H	-1.50642	-4.03117	3.35710
C	-1.98500	-5.14348	1.56351
H	-1.24336	-5.92858	1.75008
C	-2.83925	-5.21645	0.45271
H	-2.76199	-6.07346	-0.22774
C	-3.82932	-4.24181	0.20770
C	-3.22375	-1.96519	3.31323
H	-4.00798	-1.27523	2.96298
C	-3.67415	-2.57814	4.66123
H	-2.88724	-3.22424	5.09161
H	-4.58171	-3.19519	4.54898
H	-3.88745	-1.78171	5.39565
C	-1.93055	-1.13915	3.50570
H	-1.69470	-0.51486	2.62374
H	-1.06775	-1.79061	3.74622
H	-2.05212	-0.44848	4.35953
C	-4.79458	-4.47498	-0.95754
H	-5.50341	-3.62940	-0.94902
C	-5.58429	-5.79242	-0.76457
H	-6.07465	-5.84082	0.22002
H	-4.92108	-6.67226	-0.85237
H	-6.36285	-5.88923	-1.54195
C	-4.09784	-4.49698	-2.33579
H	-3.34803	-5.30830	-2.39453
H	-3.59692	-3.54002	-2.54657
H	-4.83806	-4.67894	-3.13505
C	-1.87145	-0.04886	-1.24148
H	-0.94402	0.33077	-1.73918
H	0.74086	0.22653	0.95042
C	-2.84902	-1.02048	-1.90091
H	-3.23262	-0.69575	-2.88816
H	-2.42649	-2.03706	-2.08428
C	-0.21719	3.50300	2.58644
H	-0.78828	2.58973	2.37180
C	1.16757	3.53289	2.33673
H	1.66161	2.63882	1.94269
C	1.91675	4.69343	2.59581
H	2.99490	4.70266	2.40044
C	1.27907	5.83805	3.10777
H	1.85985	6.74359	3.31537

```
C  -0.10328  5.81526  3.36226
H  -0.59895  6.70273  3.77118
C  -0.84730  4.65015  3.10325
H  -1.92181  4.63058  3.31670
C   0.59247  4.17849 -2.66688
H   1.10517  3.34171 -3.15254
C   0.92607  4.53944 -1.34833
H   1.69429  3.98397 -0.80130
C   0.27315  5.61988 -0.72536
H   0.53641  5.89372  0.30182
C  -0.71075  6.34066 -1.42674
H  -1.21154  7.18913 -0.94836
C  -1.04412  5.98085 -2.74538
H  -1.80489  6.54764 -3.29202
C  -0.39362  4.89924 -3.36543
H  -0.64896  4.62219 -4.39344
C  -2.20230  0.27438  0.08921
O  -1.48958  1.01629  0.91122
```

D

```
SCF (BP86) Energy = -2906.32104748
Enthalpy 0K = -2904.773494
Enthalpy 298K = -2904.772550
Free Energy 298K = -2905.018194
Lowest Frequency = 4.2180 cm-1
Second Frequency = 8.4324 cm-1
SCF (BP86-D3BJ) Energy =  -2906.81709957
SCF (C6H6) Energy =  -2906.33531388
SCF (6-311++G**) Energy =  -5673.69905755
```

```
K  -1.50717  3.00952  1.29759
K  -0.65416 -1.96129  0.94995
Si  6.16560 -2.20116 -1.14239
Si  7.52755  0.48470 -0.21930
Si -6.58474  1.53081 -1.22258
Si -6.73998 -1.32658  0.05091
Al  4.39076  0.04126  0.11949
Al -3.85603  0.13446 -0.40958
O   7.37630 -1.17244 -0.55085
O  -7.29295  0.06107 -0.75333
N   4.66656 -1.73909 -0.28158
N   5.86145  1.13042 -0.08503
N  -5.03554  1.68027 -0.38565
N  -4.98919 -1.42928 -0.17660
C   3.70850 -2.70735  0.18293
C   3.83155 -3.24723  1.50518
C   2.83878 -4.12370  1.98450
H   2.92818 -4.53023  2.99783
C   1.75461 -4.51004  1.18405
H   1.01025 -5.21906  1.56398
C   1.66323 -4.01975 -0.12663
H   0.84241 -4.35884 -0.77130
C   2.62050 -3.12228 -0.64791
C   5.01856 -2.89443  2.40567
H   5.79478 -2.45548  1.75563
C   4.64425 -1.82298  3.45474
H   3.83019 -2.17612  4.11252
H   4.29726 -0.88697  2.98149
H   5.51318 -1.57732  4.09007
C   5.62967 -4.13190  3.09824
H   5.87484 -4.92403  2.37123
H   4.94769 -4.56657  3.85018
H   6.55729 -3.85116  3.62626
C   2.48428 -2.66296 -2.09980
H   3.26833 -1.90607 -2.27267
C   1.11862 -2.00697 -2.39950
H   0.91704 -1.13782 -1.74866
H   0.28441 -2.72257 -2.28423
H   1.08407 -1.64580 -3.44158
C   2.73350 -3.84121 -3.07146
H   1.95754 -4.61921 -2.95913
H   3.71005 -4.32143 -2.89002
H   2.70858 -3.49402 -4.11922
C   6.65776 -3.98384 -0.76440
H   6.94452 -4.10797  0.29167
H   7.52013 -4.27125 -1.38915
H   5.83176 -4.68142 -0.98212
C   6.05051 -1.93729 -3.01595
H   7.04502 -2.08079 -3.47246
H   5.70876 -0.91793 -3.26266
H   5.35520 -2.65139 -3.48713
C   8.53093  0.55530  1.38411
H   9.48406  0.02029  1.22988
H   7.99925  0.06476  2.21597
H   8.76821  1.58846  1.68503
C   8.49235  1.35143 -1.59593
H   8.08103  1.12117 -2.59146
H   9.54515  1.02249 -1.57553
H   8.47447  2.44603 -1.46070
C   5.61608  2.55431 -0.20484
C   5.66620  3.40640  0.94144
C   5.37773  4.77763  0.78955
H   5.41193  5.42806  1.67110
C   5.04666  5.32437 -0.45420
H   4.82758  6.39315 -0.55016
C   5.00032  4.48861 -1.57573
H   4.73792  4.91276 -2.55114
C   5.28279  3.11244 -1.48048
C   5.98195  2.87998  2.34212
H   6.28273  1.82485  2.23442
C   7.14773  3.64430  3.00820
H   6.87784  4.69420  3.21883
H   8.04573  3.65823  2.36796
H   7.41855  3.17540  3.97028
C   4.72997  2.91908  3.24916
H   3.90056  2.33503  2.81567
H   4.37204  3.95509  3.38688
H   4.95555  2.50593  4.24822
C   5.17448  2.24739 -2.73782
H   5.67152  1.28808 -2.51224
C   5.88106  2.86341 -3.96410
H   6.93275  3.10989 -3.74372
H   5.38350  3.78749 -4.30671
H   5.86405  2.15455 -4.81034
C   3.69507  1.93826 -3.06743
H   3.13540  2.86692 -3.27677
H   3.18217  1.43552 -2.22739
H   3.61375  1.28524 -3.95426
C   1.90985  0.18600  1.86572
H   2.37782  0.57936  2.78564
H   1.99642 -0.91534  1.93339
C   2.60017  0.73615  0.59241
H   2.66954  1.83956  0.64341
H   1.94864  0.53165 -0.28345
C  -4.58598  2.90033  0.17322
C  -3.97545  3.92766 -0.63362
C  -3.54568  5.13395 -0.03871
H  -3.11065  5.91312 -0.67720
C  -3.67961  5.37008  1.33846
H  -3.37190  6.32718  1.77536
C  -4.24876  4.36533  2.13762
H  -4.37052  4.53777  3.21435
C  -4.69636  3.14426  1.59099
C  -3.76987  3.75037 -2.13797
H  -4.09808  2.72620 -2.38131
C  -2.27994  3.86651 -2.53320
H  -1.67219  3.10896 -2.00719
H  -1.87317  4.87021 -2.30795
H  -2.15281  3.69669 -3.61676
C  -4.62275  4.74938 -2.95217
H  -4.31860  5.79346 -2.75429
H  -5.69417  4.66475 -2.70494
```

```
H  -4.50687  4.56842 -4.03555
C  -5.29343  2.10218  2.53233
H  -5.58068  1.24888  1.89566
C  -4.25532  1.60144  3.56325
H  -3.94105  2.41946  4.24083
H  -3.35750  1.17874  3.07307
H  -4.69703  0.81252  4.19818
C  -6.56319  2.62550  3.23976
H  -7.31871  2.97121  2.51474
H  -6.33322  3.47168  3.91284
H  -7.01921  1.82884  3.85359
C  -6.43502  1.39989 -3.11309
H  -7.39252  1.05767 -3.54214
H  -5.65800  0.66488 -3.38410
H  -6.17331  2.36190 -3.58428
C  -7.76921  2.93540 -0.75444
H  -8.01474  2.90175  0.31952
H  -8.71014  2.84054 -1.32254
H  -7.33507  3.92558 -0.97360
C  -7.67460 -2.80396 -0.68659
H  -7.62772 -2.80269 -1.78642
H  -8.73636 -2.74990 -0.39012
H  -7.27153 -3.76370 -0.31992
C  -7.36569 -1.16150  1.84422
H  -8.43945 -0.90555  1.81100
H  -6.84936 -0.36913  2.40907
H  -7.26355 -2.10492  2.40589
C  -4.23543 -2.63827 -0.12187
C  -3.72359 -3.13994  1.12940
C  -2.85155 -4.25058  1.14028
H  -2.49863 -4.63830  2.10559
C  -2.46106 -4.89268 -0.04451
H  -1.79432 -5.76258 -0.01649
C  -3.00254 -4.44734 -1.25798
H  -2.74982 -4.97870 -2.18347
C  -3.89728 -3.36000 -1.32326
C  -4.16549 -2.55994  2.47567
H  -4.79112 -1.68169  2.24975
C  -5.02564 -3.59359  3.24188
H  -4.42272 -4.46988  3.54296
H  -5.86090 -3.96626  2.62537
H  -5.44687 -3.14679  4.15992
C  -2.99606 -2.07546  3.36307
H  -2.47608 -1.20206  2.92265
H  -2.27192 -2.89166  3.56176
H  -3.37734 -1.75494  4.34914
C  -4.55107 -3.05104 -2.67305
H  -5.17569 -2.15358 -2.52399
C  -5.45958 -4.22832 -3.10801
H  -6.18534 -4.50374 -2.32760
H  -4.85713 -5.12643 -3.33639
H  -6.02077 -3.96497 -4.02246
C  -3.54439 -2.75374 -3.80536
H  -2.88621 -3.62068 -3.99988
H  -2.91207 -1.88526 -3.56816
H  -4.08400 -2.53833 -4.74474
C  -1.47422  0.53290 -0.63776
H  -0.39073  0.80504 -0.74313
H   0.83067  0.45009  1.89387
C  -2.38373  0.20930 -1.81909
H  -2.42114  0.99002 -2.60332
H  -2.09073 -0.73143 -2.32493
C  -2.09434  0.38292  0.60996
O  -1.48966  0.48154  1.79591
```

E

```
SCF (BP86) Energy = -2906.32260418
Enthalpy 0K = -2904.774749
Enthalpy 298K = -2904.773805
Free Energy 298K = -2905.016859
Lowest Frequency = 5.5512 cm-1
Second Frequency = 8.9865 cm-1
SCF (BP86-D3BJ) Energy =  -2906.82251586
SCF (C6H6) Energy =  -2906.33418737
SCF (6-311++G**) Energy =  -5673.69937999

K  -0.73621  2.71216  0.42169
Si -6.42600  1.63279  0.49954
Si -6.54582 -1.01254 -1.18219
Al -3.75968  0.41930 -0.75875
O  -6.96174  0.56280 -0.70377
O  -0.78178  0.09404  0.50899
N  -4.69404  1.84212  0.22048
N  -4.84841 -1.21020 -0.73466
C  -3.97977  2.99336  0.64741
C  -3.71170  4.09428 -0.25168
C  -2.97743  5.21327  0.19767
H  -2.80766  6.04397 -0.49771
C  -2.48808  5.30965  1.50765
H  -1.95117  6.20464  1.84234
C  -2.73116  4.24529  2.38615
H  -2.36247  4.30528  3.41770
C  -3.44759  3.09591  1.98661
C  -4.20755  4.12459 -1.69904
H  -4.64769  3.13548 -1.91065
C  -3.05367  4.37170 -2.69982
H  -3.41711  4.27154 -3.73732
H  -2.23091  3.64919 -2.56655
H  -2.64000  5.39196 -2.59660
C  -5.29007  5.21036 -1.90662
H  -5.67917  5.17291 -2.93958
H  -4.87266  6.22114 -1.74450
H  -6.13746  5.08491 -1.21669
C  -3.59440  1.97591  3.01666
H  -4.17833  1.17398  2.53540
C  -2.21205  1.39624  3.40400
H  -2.32120  0.61725  4.17858
H  -1.56073  2.18290  3.83248
H  -1.70368  0.93151  2.53643
C  -4.34802  2.45117  4.27971
H  -4.54290  1.60209  4.95822
H  -5.31458  2.91869  4.02892
H  -3.75859  3.19603  4.84499
C  -7.43573  3.22479  0.28908
H  -6.97764  4.07485  0.82275
H  -8.45184  3.07863  0.69430
H  -7.52998  3.48719 -0.77627
C  -6.93553  0.90041  2.18366
H  -6.79013  1.62370  3.00340
H  -6.38262 -0.01742  2.43965
H  -8.01075  0.65069  2.14756
C  -6.95622 -0.99354 -3.03683
H  -6.88162 -1.98744 -3.50640
H  -6.28539 -0.30269 -3.57414
H  -7.99110 -0.63223 -3.16886
C  -7.65308 -2.29294 -0.32120
H  -7.29856 -3.32015 -0.51554
H  -8.68928 -2.21557 -0.69245
H  -7.66745 -2.13621  0.76949
C  -4.24007 -2.48272 -0.57494
C  -3.73824 -3.24684 -1.69082
C  -3.12076 -4.49804 -1.47253
H  -2.76676 -5.06890 -2.33987
C  -2.96780 -5.03880 -0.18836
H  -2.51184 -6.02504 -0.04563
C  -3.44274 -4.30129  0.90596
H  -3.34539 -4.71473  1.91752
C  -4.07009 -3.04957  0.74061
C  -4.69849 -3.71081 -3.99874
H  -4.85371 -3.28818 -5.00692
H  -5.68781 -3.89494 -3.54791
H  -4.20538 -4.69229 -4.12262
```

```
H  -4.33586 -1.76944 -3.10132
C  -2.45006 -2.56264 -3.77477
H  -2.54592 -2.17849 -4.80555
H  -1.89624 -3.51887 -3.82954
H  -1.84879 -1.83130 -3.20582
C  -3.84458 -2.75535 -3.13403
C  -4.54557 -2.31372  1.98853
H  -5.04786 -1.40017  1.62838
C  -5.55513 -3.14093  2.81393
H  -5.94357 -2.54431  3.65839
H  -5.08732 -4.04710  3.24031
H  -6.41161 -3.46456  2.20042
C  -3.34906 -1.88612  2.86543
H  -3.69417 -1.31028  3.74212
H  -2.63941 -1.25310  2.30460
H  -2.80346 -2.76942  3.24944
C  -2.85946  0.79563 -2.53632
H  -3.00933  0.07001 -3.35925
H  -2.94092  1.79442 -2.99882
C  -1.55851  0.58289 -1.75913
H  -0.54498  0.65489 -2.22340
C  -1.74653  0.27588 -0.40571
H   2.22439  0.04536 -1.93772
H   1.46313  0.21967 -0.36558
C   2.13807  2.09509 -1.24484
H   1.17129  2.28323 -1.75375
H   2.17181  2.68913 -0.31186
H   2.92610  2.51654 -1.88849
C   2.32177  0.58529 -0.97207
Si  5.53853 -2.05017  1.69624
Si  7.21213 -0.03622 -0.01618
Al  4.00994 -0.07142 -0.17876
O   6.83920 -1.49758  0.75765
N   4.04713 -1.65122  0.78490
N   5.64499  0.77627 -0.32826
C   3.03630 -2.65340  0.61098
C   2.91136 -3.35140 -0.63667
C   1.99171 -4.41539 -0.74131
H   1.92644 -4.97148 -1.68345
C   1.19123 -4.80590  0.34163
H   0.51657 -5.66427  0.25272
C   1.26558 -4.07737  1.53805
H   0.62570 -4.35982  2.38276
C   2.15378 -2.99230  1.68852
C   3.76600 -3.01459 -1.86138
H   4.29419 -2.06091 -1.65383
C   2.92073 -2.80892 -3.13771
H   3.56055 -2.46906 -3.97002
H   2.12820 -2.05621 -2.98804
H   2.43708 -3.74686 -3.46190
C   4.86371 -4.07609 -2.10217
H   5.46870 -3.81664 -2.98834
H   4.41482 -5.06966 -2.27749
H   5.54256 -4.15769 -1.23855
C   2.11761 -2.19151  2.99079
H   2.87176 -1.39245  2.89496
C   0.73954 -1.51648  3.19023
H   0.76235 -0.85898  4.07745
H  -0.04766 -2.27127  3.37387
H   0.42884 -0.90965  2.32132
C   2.47376 -3.05167  4.22432
H   2.47973 -2.43198  5.13821
H   3.46363 -3.52890  4.12859
H   1.73273 -3.85614  4.37914
C   5.74234 -3.91458  1.90889
H   4.88844 -4.36154  2.44309
H   6.65728 -4.11774  2.49060
H   5.83604 -4.42261  0.93620
C   5.64247 -1.17111  3.37411
H   4.93657 -1.59541  4.10634
H   5.42826 -0.09287  3.28369
H   6.66186 -1.28069  3.78252
C   8.17858 -0.56458 -1.55564
H   8.55978  0.29319 -2.13224
H   7.56872 -1.19244 -2.22546
H   9.04585 -1.16460 -1.22897
C   8.33439  1.03688  1.06720
H   8.44603  2.04696  0.63782
H   9.33719  0.58120  1.12903
H   7.94256  1.14027  2.09154
C   5.61150  2.18645 -0.66029
C   5.74410  2.62746 -2.01371
C   5.62755  4.00306 -2.29866
H   5.71731  4.33929 -3.33788
C   5.39465  4.94507 -1.29131
H   5.30491  6.00852 -1.53685
C   5.28736  4.51299  0.03570
H   5.11651  5.25009  0.82816
C   5.39949  3.15121  0.37607
C   7.23008  2.04479 -4.00089
H   7.44805  1.26971 -4.75620
H   8.12226  2.16443 -3.36339
H   7.08189  2.99665 -4.54077
H   6.15320  0.66319 -2.73857
C   4.74630  1.55397 -4.09988
H   4.94203  0.85211 -4.92988
H   4.49276  2.53323 -4.54314
H   3.85829  1.19460 -3.55383
C   5.98132  1.66012 -3.17537
C   5.26115  2.73629  1.84292
H   5.66164  1.71135  1.92247
C   6.06944  3.63090  2.80629
H   6.03025  3.22187  3.83102
H   5.66958  4.65904  2.85250
H   7.12728  3.69527  2.50304
C   3.77782  2.68140  2.27874
H   3.68494  2.36764  3.33363
H   3.19577  1.96775  1.66484
H   3.30201  3.67344  2.17408
K  -0.93804 -2.41007 -0.15135
```

TS(E-F)

```
SCF (BP86) Energy = -2906.32132557
Enthalpy 0K = -2904.774247
Enthalpy 298K = -2904.773303
Free Energy 298K = -2905.012022
Lowest Frequency = -11.7326 cm-1
Second Frequency = 7.8929 cm-1
SCF (BP86-D3BJ) Energy =  -2906.82345977
SCF (C6H6) Energy =  -2906.33258293
SCF (6-311++G**) Energy =  -5673.69774355
```

```
K  -0.56144  2.56463 -0.01337
Si -6.28853  1.67506  0.64296
Si -6.57878 -1.09105 -0.81471
Al -3.73816  0.30730 -0.71170
O  -6.95662  0.51035 -0.39381
O  -0.71742 -0.04566  0.43843
N  -4.59613  1.83614  0.15999
N  -4.85408 -1.28442 -0.48288
C  -3.82169  3.00786  0.37300
C  -3.60493  3.96970 -0.68496
C  -2.79247  5.10111 -0.45636
H  -2.65974  5.82584 -1.26833
C  -2.18225  5.34526  0.78202
H  -1.58583  6.25033  0.94492
C  -2.38519  4.42134  1.81616
H  -1.92740  4.60271  2.79664
C  -3.17200  3.26238  1.63746
C  -4.25996  3.84518 -2.06241
H  -4.70839  2.83881 -2.11680
C  -3.24253  3.99311 -3.21762
```

H	-3.72433	3.75969	-4.18285
H	-2.38299	3.31209	-3.10245
H	-2.85570	5.02637	-3.28971
C	-5.37717	4.90036	-2.24589
H	-5.88340	4.76114	-3.21764
H	-4.95754	5.92307	-2.23055
H	-6.13594	4.83908	-1.45227
C	-3.27791	2.30194	2.82202
H	-3.91311	1.46198	2.49626
C	-1.89223	1.72924	3.20643
H	-1.97831	1.06936	4.08744
H	-1.19157	2.54084	3.48338
H	-1.45395	1.12744	2.38658
C	-3.93703	2.97832	4.04583
H	-4.10671	2.24304	4.85196
H	-4.90679	3.43567	3.78800
H	-3.29524	3.77836	4.45805
C	-7.30317	3.26048	0.40416
H	-6.77216	4.14883	0.78658
H	-8.25959	3.17769	0.94859
H	-7.53250	3.41920	-0.66122
C	-6.62634	1.09492	2.42692
H	-6.39544	1.87965	3.16636
H	-6.05881	0.19154	2.70121
H	-7.70169	0.85935	2.51680
C	-7.12956	-1.15881	-2.63255
H	-7.12460	-2.17704	-3.05280
H	-6.48084	-0.52084	-3.25589
H	-8.15889	-0.76607	-2.70410
C	-7.62041	-2.31366	0.19773
H	-7.28108	-3.35209	0.03959
H	-8.68159	-2.25347	-0.09836
H	-7.55211	-2.09193	1.27513
C	-4.24204	-2.54514	-0.25701
C	-3.88680	-3.44002	-1.33136
C	-3.25963	-4.67369	-1.04895
H	-3.02271	-5.34668	-1.88234
C	-2.95190	-5.07021	0.26019
H	-2.49103	-6.04473	0.45789
C	-3.28247	-4.20373	1.31288
H	-3.06691	-4.50378	2.34593
C	-3.91642	-2.96544	1.08405
C	-5.15928	-4.11691	-3.42348
H	-5.42743	-3.81229	-4.45047
H	-6.08824	-4.19255	-2.83419
H	-4.72256	-5.13069	-3.48077
H	-4.61465	-2.10546	-2.82063
C	-2.86458	-3.06204	-3.63283
H	-3.08510	-2.78380	-4.67839
H	-2.35860	-4.04557	-3.65426
H	-2.16353	-2.30850	-3.23252
C	-4.16540	-3.11147	-2.79776
C	-4.24467	-2.09300	2.29034
H	-4.74553	-1.19658	1.88797
C	-5.20965	-2.79580	3.27076
H	-5.49513	-2.11145	4.08943
H	-4.74184	-3.68453	3.73251
H	-6.13090	-3.12865	2.76516
C	-2.96177	-1.63928	3.01865
H	-3.21079	-0.95767	3.85090
H	-2.27271	-1.10951	2.33698
H	-2.42665	-2.50342	3.45670
C	-2.91569	0.42022	-2.56361
H	-3.19506	-0.35738	-3.30060
H	-2.91787	1.37534	-3.11761
C	-1.59869	0.14487	-1.83579
H	-0.62202	0.01395	-2.36278
C	-1.72043	0.09366	-0.44010
H	2.12910	-0.65750	-1.93528
H	1.44213	-0.00832	-0.46775
C	2.02134	1.51134	-1.92339
H	0.99619	1.55720	-2.34169
H	2.17913	2.37770	-1.25384
H	2.71187	1.66434	-2.76793
C	2.26107	0.16661	-1.20476
Si	5.81836	-2.00548	1.42362
Si	7.06176	0.60242	0.15656
Al	4.00818	-0.15516	-0.34187
O	6.97182	-1.00270	0.69324
N	4.32574	-1.79150	0.46032
N	5.38230	1.08396	-0.24188
C	3.42947	-2.88458	0.18614
C	3.50606	-3.58220	-1.06488
C	2.58541	-4.61414	-1.33589
H	2.64544	-5.14187	-2.29457
C	1.62210	-5.00588	-0.39765
H	0.94271	-5.83809	-0.61357
C	1.57008	-4.34512	0.83826
H	0.84553	-4.67251	1.59482
C	2.44557	-3.28251	1.14762
C	4.57935	-3.27711	-2.11396
H	5.24191	-2.50230	-1.68825
C	3.98033	-2.72804	-3.42950
H	4.78112	-2.50597	-4.15644
H	3.40340	-1.80213	-3.26709
H	3.30161	-3.46333	-3.89701
C	5.45103	-4.52008	-2.41097
H	6.27897	-4.25494	-3.09119
H	4.86355	-5.31741	-2.89957
H	5.88399	-4.94327	-1.49004
C	2.32490	-2.61467	2.51770
H	3.04377	-1.77863	2.53210
C	0.91841	-2.02766	2.77166
H	0.89340	-1.50657	3.74485
H	0.15630	-2.82765	2.82951
H	0.60260	-1.30303	1.99981
C	2.70314	-3.59982	3.64897
H	2.69045	-3.09116	4.62901
H	3.70531	-4.03635	3.49940
H	1.98493	-4.43759	3.70179
C	6.44335	-3.78394	1.33472
H	5.64365	-4.50572	1.57021
H	7.26305	-3.93228	2.05756
H	6.82967	-4.01166	0.32864
C	5.67807	-1.45302	3.23359
H	5.07272	-2.14727	3.83788
H	5.23630	-0.44672	3.32611
H	6.69049	-1.41784	3.67218
C	8.27423	0.48223	-1.29349
H	8.59144	1.46236	-1.68118
H	7.84964	-0.10378	-2.12545
H	9.17326	-0.04907	-0.93530
C	7.76825	1.73805	1.49439
H	7.69813	2.79601	1.18907
H	8.83147	1.50359	1.67100
H	7.22785	1.62088	2.44749
C	4.96477	2.46897	-0.28665
C	5.20863	3.28233	-1.43927
C	4.69538	4.59333	-1.47724
H	4.87926	5.20797	-2.36584
C	3.95636	5.12824	-0.41638
H	3.56770	6.15085	-0.46925
C	3.74549	4.34528	0.72420
H	3.19802	4.76952	1.57451
C	4.24584	3.03086	0.81958
C	7.31805	3.63137	-2.79542
H	7.95608	3.21737	-3.59600
H	7.90728	3.65896	-1.86326
H	7.08365	4.67647	-3.06474
H	6.31171	1.74981	-2.43335
C	5.22851	2.80702	-3.95953
H	5.86031	2.44871	-4.79115

```
H   4.88420  3.82357 -4.21874
H   4.34016  2.15661 -3.90759
C   6.02639  2.79441 -2.63632
C   4.00697  2.26412  2.12322
H   4.55483  1.30752  2.04399
C   4.57249  3.01878  3.34823
H   4.47195  2.40500  4.26053
H   4.03116  3.96458  3.52656
H   5.63814  3.26514  3.21307
C   2.51243  1.93409  2.34443
H   2.36665  1.37026  3.28188
H   2.09091  1.32429  1.52490
H   1.91635  2.86218  2.42457
K  -0.88782 -2.56912 -0.26316
```

F

```
SCF (BP86) Energy = -2906.32233318
Enthalpy 0K = -2904.774137
Enthalpy 298K = -2904.773193
Free Energy 298K = -2905.013582
Lowest Frequency = 7.8959 cm-1
Second Frequency = 11.9969 cm-1
SCF (BP86-D3BJ) Energy =  -2906.83070830
SCF (C6H6) Energy =  -2906.33290044
SCF (6-311++G**) Energy =  -5673.69745717
```

```
K  -0.41861  2.51312 -0.21735
Si -6.19875  1.56082  0.66094
Si -6.45434 -1.29704 -0.61216
Al -3.64738  0.17837 -0.67843
O  -6.87400  0.31206 -0.26748
O  -0.59188 -0.07702  0.42213
N  -4.53564  1.73938  0.09104
N  -4.71511 -1.42194 -0.32134
C  -3.78118  2.94057  0.17152
C  -3.60852  3.79850 -0.97916
C  -2.80261  4.95302 -0.88356
H  -2.70118  5.59816 -1.76414
C  -2.16216  5.32092  0.30808
H  -1.57269  6.24313  0.36590
C  -2.32975  4.50251  1.43342
H  -1.85415  4.78629  2.38074
C  -3.10799  3.32531  1.38854
C  -4.31736  3.54399 -2.31146
H  -4.72189  2.51862 -2.27190
C  -3.36807  3.65109 -3.52710
H  -3.88376  3.31460 -4.44311
H  -2.46580  3.02927 -3.40499
H  -3.04491  4.69378 -3.70269
C  -5.49062  4.53411 -2.50892
H  -6.03475  4.30715 -3.44300
H  -5.11808  5.57252 -2.58100
H  -6.20751  4.49357 -1.67611
C  -3.19349  2.49150  2.66633
H  -3.81583  1.61252  2.43289
C  -1.80092  1.98018  3.10118
H  -1.87497  1.40368  4.03992
H  -1.11288  2.82501  3.29842
H  -1.35462  1.31340  2.33948
C  -3.86023  3.28187  3.81526
H  -4.00924  2.63533  4.69799
H  -4.84130  3.68749  3.51652
H  -3.23404  4.13589  4.13214
C  -7.26836  3.10152  0.37158
H  -6.73879  4.02722  0.65469
H  -8.18796  3.03864  0.97848
H  -7.56537  3.17652 -0.68628
C  -6.45043  1.07699  2.48762
H  -6.22341  1.90916  3.17428
H  -5.84349  0.20880  2.78983
H  -7.51324  0.81068  2.62750
C  -7.06117 -1.47540 -2.40538
H  -7.05467 -2.51437 -2.77090
H  -6.44511 -0.86215 -3.08431
H  -8.09837 -1.10126 -2.46144
C  -7.42435 -2.49437  0.49715
H  -7.05690 -3.52908  0.38399
H  -8.49571 -2.48259  0.23368
H  -7.32943 -2.21261  1.55846
C  -4.05567 -2.64577 -0.03683
C  -3.74282 -3.61610 -1.05828
C  -3.07447 -4.81349 -0.71954
H  -2.87400 -5.54653 -1.51086
C  -2.68011 -5.09982  0.59517
H  -2.18981 -6.04893  0.84026
C  -2.96206 -4.15578  1.59415
H  -2.67714 -4.36826  2.63204
C  -3.63373 -2.94928  1.30935
C  -5.12251 -4.47085 -3.01280
H  -5.45254 -4.25385 -4.04406
H  -6.01561 -4.50824 -2.36710
H  -4.67417 -5.48116 -3.01288
H  -4.57518 -2.41168 -2.60320
C  -2.86167 -3.40972 -3.43993
H  -3.14841 -3.22014 -4.48927
H  -2.33963 -4.38459 -3.41315
H  -2.15177 -2.61694 -3.14522
C  -4.11032 -3.40786 -2.52731
C  -3.90533 -1.99177  2.46475
H  -4.41721 -1.11987  2.02419
C  -4.83809 -2.62132  3.52375
H  -5.08439 -1.88536  4.30977
H  -4.35992 -3.48624  4.01899
H  -5.78177 -2.97360  3.07579
C  -2.59336 -1.50133  3.11395
H  -2.81111 -0.77048  3.91248
H  -1.93081 -1.01561  2.37459
H  -2.04351 -2.33881  3.58414
C  -2.86763  0.16289 -2.54966
H  -3.16843 -0.66397 -3.22173
H  -2.87675  1.07702 -3.16836
C  -1.53716 -0.06767 -1.83361
H  -0.57888 -0.26052 -2.37418
C  -1.62025  0.00597 -0.43597
H   2.12994 -1.05318 -1.87454
H   1.38508 -0.03646 -0.65906
C   2.05958  1.04797 -2.44741
H   1.04507  1.02951 -2.89016
H   2.23388  2.05501 -2.02571
H   2.77487  0.93777 -3.28150
C   2.23171 -0.06243 -1.38758
Si  6.06283 -1.75259  1.20078
Si  6.77711  1.15774  0.14320
Al  3.94401 -0.11494 -0.41812
O   6.97256 -0.47501  0.55943
N   4.52078 -1.71742  0.29848
N   5.02756  1.36944 -0.16348
C   3.75301 -2.89258 -0.02708
C   3.91248 -3.52462 -1.30385
C   3.07201 -4.60199 -1.64796
H   3.18835 -5.07443 -2.62991
C   2.11033 -5.09767 -0.75908
H   1.48674 -5.95449 -1.03738
C   1.99025 -4.51077  0.50849
H   1.27735 -4.92910  1.23056
C   2.79026 -3.41426  0.89499
C   4.98153 -3.08599 -2.30897
H   5.59667 -2.31380 -1.81402
C   4.36970 -2.45960 -3.58372
H   5.16575 -2.14943 -4.28318
H   3.75355 -1.57385 -3.35431
H   3.72379 -3.18272 -4.11269
```

C	5.91681	-4.25662	-2.69190
H	6.73884	-3.89595	-3.33451
H	5.37800	-5.03893	-3.25502
H	6.35908	-4.73222	-1.80131
C	2.62652	-2.85262	2.30797
H	3.27749	-1.96542	2.37973
C	1.18252	-2.39765	2.61388
H	1.11753	-1.99097	3.63810
H	0.47488	-3.24735	2.57378
H	0.82928	-1.60492	1.93041
C	3.09493	-3.88376	3.36225
H	3.05998	-3.44877	4.37655
H	4.12494	-4.22854	3.16916
H	2.44458	-4.77677	3.36039
C	7.00063	-3.36250	0.89938
H	6.36047	-4.23955	1.09361
H	7.87490	-3.42313	1.56904
H	7.36018	-3.42029	-0.13999
C	5.89735	-1.44341	3.06454
H	5.42728	-2.29250	3.58657
H	5.30651	-0.53827	3.28279
H	6.90463	-1.30022	3.49289
C	7.91402	1.33167	-1.36254
H	8.07103	2.37623	-1.67235
H	7.52582	0.76559	-2.22572
H	8.89649	0.90399	-1.09721
C	7.35563	2.29443	1.53935
H	7.03816	3.33594	1.36037
H	8.45594	2.28052	1.61388
H	6.94254	1.97252	2.50871
C	4.30435	2.61041	-0.02535
C	4.37588	3.63482	-1.02617
C	3.58149	4.79022	-0.89193
H	3.64607	5.56877	-1.66033
C	2.71534	4.96975	0.19332
H	2.11663	5.88282	0.28234
C	2.65939	3.98113	1.18362
H	2.02271	4.13507	2.06343
C	3.44421	2.81071	1.10677
C	6.46772	4.55569	-2.10409
H	7.18442	4.44207	-2.93665
H	7.01903	4.43634	-1.15606
H	6.08500	5.59141	-2.13226
H	5.73445	2.52101	-2.23331
C	4.58252	3.73849	-3.58211
H	5.28959	3.59923	-4.41841
H	4.16370	4.75600	-3.67511
H	3.75674	3.02094	-3.71050
C	5.30885	3.53787	-2.23305
C	3.38067	1.83472	2.28493
H	4.00462	0.95806	2.02729
C	3.99445	2.46928	3.55635
H	4.01901	1.73784	4.38325
H	3.39382	3.33276	3.89338
H	5.02120	2.82720	3.37827
C	1.95131	1.33285	2.58151
H	1.96690	0.59269	3.39987
H	1.46535	0.85349	1.71305
H	1.29909	2.15892	2.91705
K	-0.70422	-2.63639	-0.24585

TS(F-2)

SCF (BP86) Energy = -2906.32073882
Enthalpy 0K = -2904.773391
Enthalpy 298K = -2904.772447
Free Energy 298K = -2905.006967
Lowest Frequency = -28.3986 cm-1
Second Frequency = 12.2307 cm-1
SCF (BP86-D3BJ) Energy = -2906.83498391
SCF (C6H6) Energy = -2906.32950026
SCF (6-311++G**) Energy = -5673.69478573

K	-0.36350	2.81868	0.27975
Si	-5.99545	1.12882	0.53308
Si	-5.93286	-1.49053	-1.19240
Al	-3.26909	0.10897	-0.74273
O	-6.45362	0.04464	-0.69091
O	-0.17991	0.16482	0.34799
N	-4.27297	1.43784	0.29135
N	-4.24086	-1.59766	-0.68890
C	-3.64712	2.61773	0.78165
C	-3.51609	3.80559	-0.03143
C	-2.88180	4.95400	0.49125
H	-2.81801	5.85074	-0.13696
C	-2.35574	4.99153	1.78965
H	-1.89419	5.90465	2.18247
C	-2.46130	3.84047	2.58208
H	-2.06372	3.85333	3.60439
C	-3.08751	2.66664	2.11141
C	-4.03423	3.89567	-1.46878
H	-4.45161	2.90688	-1.72446
C	-2.89025	4.21500	-2.46167
H	-3.26168	4.19111	-3.50121
H	-2.06841	3.48287	-2.38776
H	-2.47831	5.22597	-2.28398
C	-5.14041	4.96617	-1.62253
H	-5.54567	4.95352	-2.64988
H	-4.74163	5.98051	-1.43806
H	-5.97432	4.80379	-0.92380
C	-3.12978	1.46775	3.05745
H	-3.67979	0.66959	2.53169
C	-1.70373	0.95230	3.36027
H	-1.74145	0.09010	4.04981
H	-1.10068	1.73498	3.85913
H	-1.17946	0.63665	2.43761
C	-3.87152	1.79565	4.37300
H	-3.98774	0.88649	4.98909
H	-4.87483	2.21263	4.18606
H	-3.31404	2.53353	4.97829
C	-7.10048	2.65472	0.32043
H	-6.73606	3.51046	0.91369
H	-8.12362	2.41878	0.66055
H	-7.15458	2.95553	-0.73729
C	-6.48210	0.36276	2.20869
H	-6.39788	1.10054	3.02447
H	-5.86965	-0.51160	2.48025
H	-7.53725	0.04031	2.16079
C	-6.25840	-1.45853	-3.06280
H	-6.08466	-2.43419	-3.54518
H	-5.61560	-0.70797	-3.55190
H	-7.31016	-1.17411	-3.24116
C	-7.00473	-2.85053	-0.41288
H	-6.58540	-3.85221	-0.61135
H	-8.02182	-2.81951	-0.83977
H	-7.08817	-2.72234	0.67815
C	-3.56163	-2.83565	-0.54782
C	-2.90801	-3.48725	-1.65731
C	-2.18459	-4.68256	-1.45008
H	-1.70529	-5.16499	-2.31081
C	-2.07990	-5.27894	-0.18546
H	-1.52935	-6.21678	-0.05220
C	-2.72945	-4.66728	0.89704
H	-2.68158	-5.13613	1.88767
C	-3.46922	-3.47666	0.74191
C	-3.68305	-3.93475	-4.03175
H	-3.82220	-3.48457	-5.03018
H	-4.67357	-4.23123	-3.64810
H	-3.08994	-4.85813	-4.16300
H	-3.56870	-2.01033	-3.04015
C	-1.57875	-2.57617	-3.63212
H	-1.65961	-2.17738	-4.65855
H	-0.91790	-3.46249	-3.67458

```
H  -1.09681 -1.79688 -3.01493
C  -2.97748 -2.93921 -3.08223
C  -4.15456 -2.88807  1.97144
H  -4.74221 -2.02887  1.60749
C  -5.10913 -3.89118  2.65483
H  -5.65730 -3.39939  3.47782
H  -4.56036 -4.74527  3.09171
H  -5.84840 -4.29580  1.94503
C  -3.11980 -2.35557  2.98559
H  -3.62088 -1.88574  3.85066
H  -2.46515 -1.59469  2.52900
H  -2.48528 -3.17366  3.37624
C  -2.53442  0.69707 -2.53147
H  -2.53579 -0.02510 -3.37109
H  -2.86265  1.66138 -2.95587
C  -1.19831  0.76052 -1.79453
H  -0.26188  1.15835 -2.25936
C  -1.22267  0.23544 -0.49710
H   2.11951 -2.00032 -1.62238
H   1.13871 -0.52548 -1.36099
C   2.17443 -0.56985 -3.27877
H   1.37218 -1.07718 -3.84457
H   2.04888  0.50939 -3.45377
H   3.13539 -0.86266 -3.73838
C   2.09502 -0.90177 -1.77356
Si  5.89674 -0.89101  1.40910
Si  5.99658  1.59016 -0.54741
Al  3.48157 -0.16677 -0.57380
O   6.51214  0.48054  0.62825
N   4.39515 -1.34857  0.54023
N   4.20846  1.54674 -0.59326
C   3.83390 -2.68094  0.61765
C   4.15979 -3.67203 -0.36616
C   3.47416 -4.90371 -0.35664
H   3.71678 -5.65068 -1.12089
C   2.50162 -5.19992  0.60449
H   1.98736 -6.16689  0.59613
C   2.23026 -4.25743  1.60473
H   1.51632 -4.50693  2.39948
C   2.88564 -3.00805  1.64290
C   5.23861 -3.46490 -1.43280
H   5.68685 -2.47164 -1.25568
C   4.66209 -3.47927 -2.86661
H   5.46140 -3.29480 -3.60587
H   3.88379 -2.71347 -3.00497
H   4.21175 -4.45924 -3.10503
C   6.35675 -4.52852 -1.31820
H   7.17104 -4.30687 -2.03038
H   5.97468 -5.53610 -1.55959
H   6.78774 -4.56878 -0.30567
C   2.60379 -2.08672  2.83282
H   3.18967 -1.16633  2.67861
C   1.12028 -1.67695  2.96183
H   0.97101 -1.06891  3.87096
H   0.46753 -2.56419  3.07308
H   0.76472 -1.06404  2.11265
C   3.08014 -2.75157  4.14751
H   3.00007 -2.04469  4.99174
H   4.12659 -3.09327  4.07989
H   2.46264 -3.63401  4.39294
C   7.20230 -2.25637  1.34360
H   6.80129 -3.21334  1.71789
H   8.05240 -1.97208  1.98705
H   7.58366 -2.41208  0.32290
C   5.68504 -0.40136  3.22944
H   5.45497 -1.26946  3.86779
H   4.90085  0.35678  3.38243
H   6.64114  0.02766  3.57702
C   6.72895  0.98442 -2.18736
H   6.50437  1.67313 -3.01819
H   6.32650 -0.00869 -2.45402
H   7.82495  0.88740 -2.10503
C   6.64828  3.29561 -0.07089
H   6.24735  4.08892 -0.72205
H   7.74821  3.30421 -0.15252
H   6.38197  3.54033  0.96945
C   3.44595  2.76536 -0.50371
C   3.09313  3.50946 -1.67677
C   2.36782  4.71183 -1.54085
H   2.11112  5.28016 -2.44139
C   1.98674  5.20805 -0.28797
H   1.44926  6.15920 -0.20617
C   2.33077  4.48145  0.86079
H   2.05697  4.87233  1.84827
C   3.05014  3.27060  0.78010
C   4.68123  3.93309 -3.60688
H   4.99834  3.58998 -4.60754
H   5.55748  3.89309 -2.93906
H   4.37979  4.99213 -3.69480
H   3.86102  2.02731 -3.00218
C   2.33881  3.11526 -4.08984
H   2.64124  2.65449 -5.04594
H   2.03191  4.15224 -4.31381
H   1.45042  2.57801 -3.71685
C   3.50856  3.07060 -3.08074
C   3.37337  2.53241  2.07828
H   4.05202  1.70484  1.81309
C   4.09561  3.42390  3.11201
H   4.39436  2.82646  3.99086
H   3.44442  4.23813  3.47679
H   5.00332  3.88639  2.69064
C   2.09714  1.91737  2.69759
H   2.34321  1.31858  3.59217
H   1.56025  1.25917  1.99028
H   1.39933  2.71096  3.02624
K  -0.40946 -2.45626  0.07740
```

2

```
SCF (BP86) Energy = -2906.36069219
Enthalpy 0K = -2904.811447
Enthalpy 298K = -2904.810503
Free Energy 298K = -2905.040411
Lowest Frequency = 13.8170 cm-1
Second Frequency = 19.5823 cm-1
SCF (BP86-D3BJ) Energy =  -2906.88949822
SCF (C6H6) Energy =  -2906.36777789
SCF (6-311++G**) Energy =  -5673.72890991
```

```
K   0.57279 -3.34344 -0.06148
Si  5.15256 -0.23741  1.48705
Si  5.15752  1.61326 -0.96397
Al  2.49299 -0.02605 -0.40961
O   5.87007  0.79765  0.34502
O  -0.84441  0.14226  0.32996
N   3.74205 -1.01733  0.72438
N   3.39429  1.66813 -0.70224
C   3.50921 -2.41190  0.91959
C   3.82504 -3.38017 -0.10944
C   3.49184 -4.73775  0.08495
H   3.74053 -5.46055 -0.69985
C   2.88668 -5.19630  1.26584
H   2.65594 -6.25932  1.39687
C   2.63568 -4.27613  2.29187
H   2.21298 -4.62948  3.24001
C   2.94134 -2.90430  2.14964
C   4.59715 -2.99654 -1.37529
H   4.40753 -1.92486 -1.54833
C   4.17784 -3.77863 -2.63955
H   4.66028 -3.33577 -3.52745
H   3.08791 -3.76003 -2.81089
H   4.49784 -4.83550 -2.59664
C   6.11882 -3.18273 -1.16296
```

H	6.67037	-2.88819	-2.07309
H	6.35625	-4.24041	-0.94789
H	6.49188	-2.57359	-0.32862
C	2.72638	-1.99521	3.36080
H	2.93361	-0.96637	3.02635
C	1.28302	-2.01819	3.90005
H	1.19379	-1.34884	4.77353
H	0.97883	-3.02760	4.23178
H	0.56418	-1.67044	3.14224
C	3.71793	-2.35570	4.49248
H	3.61404	-1.65258	5.33778
H	4.76414	-2.32707	4.14777
H	3.52427	-3.37230	4.87989
C	6.49510	-1.48261	2.01439
H	6.13295	-2.52267	2.04883
H	6.87711	-1.22099	3.01611
H	7.34507	-1.43534	1.31425
C	4.76893	0.83794	3.01227
H	4.74392	0.23022	3.93205
H	3.80908	1.36844	2.93064
H	5.57039	1.58814	3.12878
C	5.66750	0.66627	-2.53098
H	5.48776	1.26680	-3.43835
H	5.11184	-0.27938	-2.63768
H	6.74404	0.42794	-2.48738
C	5.92328	3.34521	-1.03559
H	5.42713	3.99092	-1.77899
H	6.98583	3.25330	-1.31896
H	5.87887	3.84760	-0.05679
C	2.71526	2.89105	-0.99389
C	2.16508	3.15823	-2.29772
C	1.41252	4.33233	-2.51457
H	0.99031	4.51312	-3.50989
C	1.22682	5.28976	-1.50786
H	0.65297	6.20202	-1.70387
C	1.83253	5.07940	-0.26027
H	1.73654	5.84485	0.51856
C	2.57542	3.91066	0.01510
C	3.43112	2.98840	-4.46749
H	3.72259	2.32062	-5.29753
H	4.35007	3.32249	-3.95724
H	2.95381	3.88226	-4.90806
H	2.97467	1.36468	-3.11178
C	1.20640	1.80003	-4.26253
H	1.49164	1.14145	-5.10173
H	0.64897	2.65180	-4.69195
H	0.52088	1.23764	-3.60865
C	2.46481	2.26176	-3.49970
C	3.20186	3.75943	1.40102
H	3.92361	2.92951	1.33208
C	3.95708	5.02364	1.86473
H	4.51199	4.81217	2.79525
H	3.27117	5.86273	2.07986
H	4.67821	5.36895	1.10599
C	2.13964	3.36206	2.45033
H	2.58518	3.28208	3.45754
H	1.68569	2.38210	2.21902
H	1.33383	4.11815	2.50765
C	1.81243	-1.07771	-2.01152
H	1.99233	-0.66191	-3.02085
H	2.07029	-2.15350	-2.08905
C	0.41089	-0.82479	-1.49414
H	-0.50887	-1.14783	-2.02134
C	0.35311	-0.14623	-0.29608
H	-2.27532	2.34066	-1.71001
H	-1.10603	1.15248	-2.30009
C	-2.94980	1.22706	-3.41647
H	-2.56741	1.94596	-4.16791
H	-2.87864	0.22637	-3.87579
H	-4.02477	1.44116	-3.29277
C	-2.18210	1.29836	-2.08183

Si	-5.19456	0.35061	1.49959
Si	-5.13444	-1.63979	-0.89637
Al	-2.55911	0.12436	-0.47798
O	-5.77785	-0.85868	0.46366
N	-3.74579	1.07406	0.75951
N	-3.35158	-1.61613	-0.78919
C	-3.32620	2.39878	1.12485
C	-3.63754	3.55502	0.31235
C	-3.05558	4.80379	0.62198
H	-3.29572	5.66651	-0.00947
C	-2.21558	4.98433	1.72835
H	-1.78734	5.96784	1.95079
C	-1.98242	3.89160	2.57327
H	-1.37560	4.02855	3.47614
C	-2.52566	2.61671	2.30569
C	-4.66324	3.53805	-0.82816
H	-4.89843	2.47990	-1.03887
C	-4.18320	4.21771	-2.13320
H	-4.94585	4.09323	-2.92129
H	-3.23853	3.80289	-2.51493
H	-4.04573	5.30462	-1.99129
C	-5.95834	4.26621	-0.38289
H	-6.74316	4.16284	-1.15328
H	-5.76319	5.34569	-0.24917
H	-6.35431	3.88099	0.56608
C	-2.27097	1.51748	3.33692
H	-2.75378	0.60528	2.95640
C	-0.77437	1.19761	3.52750
H	-0.64929	0.40690	4.28751
H	-0.20836	2.07912	3.87920
H	-0.33567	0.83216	2.58371
C	-2.91263	1.89112	4.69433
H	-2.83923	1.04791	5.40346
H	-3.97704	2.15889	4.58634
H	-2.40089	2.75614	5.15378
C	-6.61831	1.57534	1.75816
H	-6.30279	2.47547	2.31226
H	-7.39899	1.07203	2.35477
H	-7.07060	1.88385	0.80370
C	-5.01542	-0.52265	3.18695
H	-4.97989	0.19627	4.02138
H	-4.13579	-1.17893	3.26546
H	-5.91656	-1.14681	3.32182
C	-5.83683	-0.70890	-2.39245
H	-5.44832	-1.08747	-3.35154
H	-5.60397	0.36629	-2.33081
H	-6.93587	-0.81282	-2.39830
C	-5.81785	-3.40704	-0.87447
H	-5.44497	-4.01734	-1.71268
H	-6.91773	-3.36030	-0.94604
H	-5.56061	-3.92617	0.06218
C	-2.71196	-2.89097	-0.87655
C	-2.23619	-3.41185	-2.13297
C	-1.64036	-4.69134	-2.18578
H	-1.30313	-5.07654	-3.15478
C	-1.51980	-5.50155	-1.04768
H	-1.09049	-6.50719	-1.12058
C	-2.00316	-5.01499	0.17682
H	-1.94544	-5.65107	1.06770
C	-2.59035	-3.73618	0.28419
C	-3.62572	-3.27321	-4.23999
H	-3.81380	-2.69664	-5.16300
H	-4.55736	-3.28736	-3.65222
H	-3.40019	-4.31438	-4.53334
H	-2.72335	-1.62676	-3.17224
C	-1.19594	-2.61210	-4.35354
H	-1.39254	-1.96919	-5.22895
H	-0.93642	-3.61311	-4.74320
H	-0.31112	-2.20974	-3.83371
C	-2.44605	-2.65873	-3.44678
C	-3.07678	-3.26530	1.65270

```
H  -3.80505 -2.45960  1.46775
C  -3.77571 -4.36036  2.48382
H  -4.23496 -3.91236  3.38163
H  -3.06980 -5.13502  2.83511
H  -4.57162 -4.86597  1.91183
C  -1.90241 -2.65480  2.44759
H  -2.23104 -2.28933  3.43691
H  -1.45229 -1.79973  1.91126
H  -1.12426 -3.41917  2.63572
K  -0.31822  2.81514  0.00046
```

G

```
SCF (BP86) Energy = -2906.28199843
Enthalpy 0K = -2904.736400
Enthalpy 298K = -2904.735456
Free Energy 298K = -2904.980016
Lowest Frequency = 4.7030 cm-1
Second Frequency = 10.2748 cm-1
SCF (BP86-D3BJ) Energy =  -2906.78400770
SCF (C6H6) Energy = -2906.296277
SCF (6-311++G**) Energy =  -5673.65841524

K   0.13995 -2.11081  1.49899
Si  6.01868 -2.30715 -0.68913
Si  6.50441  0.69463 -1.45592
Al  3.83147 -0.08933  0.03923
O   6.77121 -0.98400 -1.42930
N   4.30851 -1.87137 -0.42827
N   5.29704  1.07458 -0.21577
C   3.24075 -2.81882 -0.49785
C   2.40898 -2.89725 -1.67150
C   1.28456 -3.74784 -1.67562
H   0.65568 -3.79290 -2.57227
C   0.97611 -4.57338 -0.58513
H   0.11481 -5.24932 -0.62439
C   1.82483 -4.55701  0.53190
H   1.63093 -5.24435  1.36566
C   2.94272 -3.69660  0.60396
C   2.76483 -2.16591 -2.96911
H   3.62949 -1.51789 -2.74414
C   3.19221 -3.18430 -4.05493
H   3.99698 -3.84613 -3.69955
H   3.55054 -2.65935 -4.95801
H   2.34177 -3.82324 -4.35365
C   1.62586 -1.28307 -3.52502
H   0.73237 -1.88179 -3.77744
H   1.95441 -0.78009 -4.45179
H   1.32439 -0.50646 -2.80498
C   3.83481 -3.78079  1.84399
H   4.59761 -2.99282  1.74147
C   4.54930 -5.15136  1.90648
H   3.82438 -5.97318  2.04847
H   5.25723 -5.18219  2.75341
H   5.11053 -5.36376  0.98094
C   3.07374 -3.50787  3.16113
H   2.66613 -2.48177  3.17368
H   3.75766 -3.60245  4.02267
H   2.25795 -4.23921  3.32167
C   7.04265 -2.66750  0.87277
H   8.11045 -2.63897  0.59350
H   6.83209 -3.66449  1.29284
H   6.88358 -1.91841  1.66535
C   6.18134 -3.79379 -1.85299
H   5.99721 -3.49383 -2.89662
H   5.47464 -4.59972 -1.59169
H   7.20316 -4.20554 -1.79390
C   5.94096  1.02020 -3.24420
H   6.65018  0.54033 -3.94064
H   5.89269  2.09263 -3.49284
H   4.94517  0.58008 -3.42603
C   8.14471  1.57554 -1.11739
H   8.63428  1.15090 -0.22619
H   7.99787  2.65456 -0.94360
H   8.82810  1.45299 -1.97454
C   5.35661  2.27287  0.57773
C   4.88976  3.53102  0.07066
C   4.92991  4.67436  0.89412
H   4.58091  5.63299  0.49137
C   5.40602  4.61706  2.20780
H   5.42432  5.51577  2.83360
C   5.86782  3.39360  2.70434
H   6.25400  3.34041  3.72917
C   5.86298  2.22503  1.91908
C   4.37097  3.69131 -1.35957
H   4.30255  2.67820 -1.79135
C   5.34850  4.52438 -2.22107
H   5.44065  5.55279 -1.82839
H   4.99775  4.59408 -3.26683
H   6.35918  4.08412 -2.22808
C   2.96234  4.32667 -1.41594
H   2.24702  3.76148 -0.79241
H   2.58843  4.34683 -2.45656
H   2.96717  5.36806 -1.04869
C   6.43063  0.94300  2.52690
H   6.35114  0.16472  1.74878
C   7.92597  1.10036  2.88741
H   8.52011  1.43329  2.02029
H   8.34305  0.14112  3.24222
H   8.06925  1.84291  3.69283
C   5.62884  0.47263  3.76115
H   6.04823 -0.46930  4.15848
H   4.56968  0.29826  3.51297
H   5.66898  1.21999  4.57397
K  -0.72021  2.22167 -0.99206
Si -6.24956  2.17196  0.51510
Si -6.74485 -0.59537 -0.84874
Al -3.84982  0.15014  0.35130
O  -7.12382  0.97561 -0.30464
N  -4.51329  1.91840  0.18882
N  -5.21385 -1.07991 -0.10302
C  -3.68586  3.01145 -0.18777
C  -2.95962  3.76732  0.79763
C  -2.12110  4.82776  0.38849
H  -1.58157  5.40266  1.15036
C  -1.98575  5.18217 -0.96215
H  -1.35051  6.02497 -1.25566
C  -2.70582  4.46019 -1.92807
H  -2.62564  4.74846 -2.98279
C  -3.55039  3.38674 -1.57256
C  -3.10763  3.48240  2.29259
H  -3.70526  2.55921  2.38182
C  -3.87689  4.62806  2.99215
H  -4.85914  4.80401  2.52281
H  -4.04335  4.39033  4.05732
H  -3.31114  5.57587  2.94391
C  -1.75377  3.24545  2.99666
H  -1.11194  4.14459  2.97452
H  -1.91313  2.98395  4.05686
H  -1.18908  2.41586  2.53575
C  -4.27601  2.61639 -2.67545
H  -5.07531  2.03999 -2.18090
C  -4.92474  3.52840 -3.73696
H  -4.17308  4.05084 -4.35599
H  -5.54633  2.92662 -4.42165
H  -5.56949  4.29471 -3.27537
C  -3.32517  1.59476 -3.34637
H  -2.94278  0.86130 -2.61170
H  -3.84749  1.02709 -4.13636
H  -2.47081  2.10957 -3.82898
C  -6.81963  3.85750 -0.13032
H  -7.88744  3.99595  0.10987
H  -6.25662  4.68538  0.33239
```

```
H  -6.70639  3.93398 -1.22349
C  -6.66785  2.01678  2.35858
H  -6.25428  1.08976  2.78894
H  -6.26585  2.86628  2.93598
H  -7.76234  1.99656  2.49798
C  -8.16348 -1.74845 -0.35719
H  -9.05202 -1.53567 -0.97552
H  -7.89063 -2.80734 -0.50230
H  -8.44071 -1.60654  0.69911
C  -6.71968 -0.45660 -2.74565
H  -5.83711  0.09603 -3.10663
H  -6.71851 -1.44771 -3.22807
H  -7.62128  0.08391 -3.08241
C  -4.94237 -2.43762  0.29350
C  -5.19223 -2.87093  1.63840
C  -4.78982 -4.16112  2.03627
H  -4.97485 -4.47870  3.06945
C  -4.18114 -5.05182  1.14392
H  -3.88692 -6.05497  1.47234
C  -3.99304 -4.65411 -0.18498
H  -3.55917 -5.36173 -0.90182
C  -4.36388 -3.36949 -0.63064
C  -5.92447 -1.99636  2.65853
H  -6.22933 -1.07832  2.12701
C  -7.20144 -2.69187  3.18619
H  -6.95489 -3.58183  3.79248
H  -7.77583 -2.00371  3.83151
H  -7.85764 -3.02218  2.36450
C  -5.02444 -1.57909  3.84360
H  -4.15287 -1.00490  3.49118
H  -5.59005 -0.95381  4.55797
H  -4.65996 -2.46390  4.39697
C  -4.19892 -3.03800 -2.11504
H  -4.40733 -1.96032 -2.22699
C  -5.23066 -3.82593 -2.95795
H  -6.26133 -3.65312 -2.60578
H  -5.17665 -3.53648 -4.02294
H  -5.04108 -4.91259 -2.89488
C  -2.77763 -3.30520 -2.65724
H  -2.70805 -3.00012 -3.71663
H  -2.01145 -2.74702 -2.09314
H  -2.51661 -4.37773 -2.60989
C  -2.42265 -0.48662  1.55018
C  -1.93752 -0.44589  0.04228
H  -2.42756 -1.45560  2.08527
H  -1.96026  0.29768  2.17218
H  -2.09021 -1.42414 -0.45301
H  -0.88187 -0.15813 -0.15616
C   1.79800  1.36313  0.87682
C   2.01220  0.77846 -0.54571
H   0.76278  1.50909  1.26016
H   2.36236  2.30744  1.00280
H   1.25110  0.01820 -0.80271
H   2.12324  1.50944 -1.36552
C   2.53587  0.23454  1.65705
O   1.92444 -0.43636  2.51400
```

TS(G-D)

SCF (BP86) Energy = -2906.24456900
Enthalpy 0K = -2904.703715
Enthalpy 298K = -2904.702771
Free Energy 298K = -2904.942447
Lowest Frequency = -1028.3590 cm-1
Second Frequency = 7.9423 cm-1
SCF (BP86-D3BJ) Energy = -2906.75132346
SCF (C6H6) Energy = -2906.260906
SCF (6-311++G**) Energy = -5673.61794259

```
K   0.32193 -2.22211  0.66871
Si  5.83019 -1.65787 -1.53256
Si  6.29306  1.41148 -1.14542
Al  3.52808  0.17013 -0.25451
O   6.36544 -0.10146 -1.92485
N   4.12322 -1.48897 -1.07470
N   5.05018  1.28943  0.10369
C   3.17674 -2.53796 -1.21866
C   2.24810 -2.55727 -2.32519
C   1.29571 -3.59256 -2.43105
H   0.61671 -3.60041 -3.29194
C   1.21909 -4.63087 -1.49246
H   0.49567 -5.44464 -1.61547
C   2.11188 -4.62158 -0.41197
H   2.07422 -5.43460  0.32389
C   3.07259 -3.60006 -0.24674
C   2.28780 -1.51287 -3.44302
H   2.96887 -0.71187 -3.10843
C   2.84464 -2.11704 -4.75401
H   3.84109 -2.55853 -4.60853
H   2.92336 -1.34135 -5.53664
H   2.17697 -2.91173 -5.13436
C   0.89926 -0.89624 -3.72973
H   0.21823 -1.62820 -4.20119
H   0.99615 -0.04792 -4.43154
H   0.41088 -0.53077 -2.81034
C   3.96992 -3.67081  0.98967
H   4.65827 -2.81246  0.93628
C   4.80355 -4.97239  1.01347
H   4.16151 -5.86124  1.15326
H   5.52592 -4.95335  1.84804
H   5.36509 -5.11673  0.07525
C   3.15280 -3.53098  2.29651
H   2.66666 -2.54004  2.37476
H   3.81292 -3.63312  3.17525
H   2.38911 -4.32984  2.37594
C   7.02283 -2.33746 -0.21436
H   8.05303 -2.20066 -0.58858
H   6.87646 -3.41439 -0.02996
H   6.94168 -1.80798  0.74765
C   6.07134 -2.73210 -3.07985
H   5.83320 -2.16026 -3.99069
H   5.44090 -3.63726 -3.05655
H   7.12420 -3.05513 -3.15011
C   5.93522  2.55608 -2.62717
H   6.63584  2.30208 -3.44171
H   6.05352  3.62643 -2.39697
H   4.91074  2.39160 -3.00339
C   7.98161  1.85595 -0.40555
H   8.36120  1.03499  0.22428
H   7.91446  2.76090  0.22165
H   8.71665  2.04375 -1.20660
C   5.10015  2.11071  1.28270
C   4.76330  3.50648  1.25565
C   4.86430  4.27995  2.42946
H   4.61392  5.34723  2.38402
C   5.26764  3.72384  3.64616
H   5.34122  4.34184  4.54769
C   5.56714  2.35889  3.68810
H   5.87596  1.90539  4.63758
C   5.49164  1.54491  2.54235
C   4.26930  4.21244 -0.00745
H   4.23707  3.45640 -0.80923
C   5.21487  5.35391 -0.44470
H   5.24373  6.15986  0.31047
H   4.88206  5.80347 -1.39821
H   6.24866  4.99449 -0.57813
C   2.83469  4.76329  0.17940
H   2.14881  3.96164  0.50401
H   2.45975  5.20305 -0.76577
H   2.80485  5.55668  0.94746
C   5.84496  0.06778  2.69707
H   5.68961 -0.39917  1.70894
C   7.32790 -0.12448  3.08962
```

```
H   8.00643  0.35838  2.36661
H   7.58533 -1.19814  3.13908
H   7.53693  0.31214  4.08297
C   4.91942 -0.63736  3.71318
H   5.18034 -1.70861  3.78884
H   3.85997 -0.55528  3.41828
H   5.02992 -0.20314  4.72356
K  -0.56897  2.74798 -1.26415
Si -6.50111  1.61028  0.49622
Si -6.60254 -1.53692  0.61440
Al -4.03808 -0.05456 -0.36254
O  -7.07600  0.04034  0.19158
N  -4.87091  1.61455 -0.22640
N  -4.82296 -1.46699  0.57064
C  -4.24692  2.71731 -0.88613
C  -3.59443  3.75420 -0.13304
C  -2.93566  4.80097 -0.81492
H  -2.47605  5.60829 -0.23178
C  -2.87349  4.84244 -2.21666
H  -2.37083  5.67124 -2.72831
C  -3.51508  3.83512 -2.95235
H  -3.50643  3.88417 -4.04751
C  -4.22288  2.78940 -2.32255
C  -3.64121  3.79722  1.39516
H  -4.06997  2.83746  1.72863
C  -4.56683  4.94146  1.87459
H  -5.57544  4.86406  1.43508
H  -4.66720  4.92819  2.97398
H  -4.15803  5.92683  1.58807
C  -2.24815  3.94141  2.04898
H  -1.75532  4.89025  1.76995
H  -2.34063  3.93868  3.14832
H  -1.57406  3.10785  1.78373
C  -5.01358  1.82116 -3.20759
H  -5.49280  1.08401 -2.53816
C  -6.14051  2.57015 -3.95994
H  -5.72600  3.28989 -4.68810
H  -6.76982  1.85583 -4.51864
H  -6.78794  3.13213 -3.26779
C  -4.13044  1.04905 -4.21276
H  -3.36961  0.43768 -3.70083
H  -4.75164  0.37134 -4.82366
H  -3.61287  1.73370 -4.90866
C  -7.64844  2.83215 -0.37376
H  -8.58178  2.95654  0.20052
H  -7.17750  3.82431 -0.47782
H  -7.91183  2.46666 -1.37898
C  -6.55010  1.88335  2.37265
H  -5.80610  1.26030  2.89621
H  -6.36527  2.93528  2.64405
H  -7.55075  1.61025  2.75020
C  -7.28259 -2.04119  2.30539
H  -8.38346 -2.09808  2.25691
H  -6.90864 -3.03815  2.59545
H  -7.00787 -1.32771  3.09746
C  -7.42495 -2.61272 -0.70949
H  -7.08072 -2.34247 -1.72144
H  -7.24259 -3.68840 -0.55624
H  -8.51560 -2.44616 -0.66979
C  -3.90719 -2.45797  1.07769
C  -3.36280 -2.32268  2.40027
C  -2.36513 -3.22060  2.83184
H  -1.95284 -3.11038  3.84131
C  -1.90533 -4.26077  2.01111
H  -1.14733 -4.96333  2.37652
C  -2.47011 -4.42058  0.73816
H  -2.14576 -5.25647  0.10752
C  -3.46960 -3.54892  0.25589
C  -3.85816 -1.26709  3.39223
H  -4.64273 -0.68863  2.87502
C  -4.48878 -1.93478  4.63801
H  -3.72872 -2.47704  5.22760
H  -4.93436 -1.17098  5.29947
H  -5.27665 -2.65504  4.36552
C  -2.75247 -0.28156  3.83386
H  -2.33111  0.27381  2.98121
H  -3.16062  0.45258  4.55072
H  -1.91928 -0.80314  4.33684
C  -4.09365 -3.85280 -1.10767
H  -4.82208 -3.05207 -1.31658
C  -4.85466 -5.20012 -1.06505
H  -5.58062 -5.23354 -0.23562
H  -5.40078 -5.36932 -2.00947
H  -4.15851 -6.04632 -0.92587
C  -3.06816 -3.86723 -2.26252
H  -3.57135 -4.09404 -3.21876
H  -2.56346 -2.89350 -2.37201
H  -2.29123 -4.63703 -2.10882
C  -1.52221  0.30279  0.19658
C  -2.23510 -0.14811 -1.11034
H  -1.67431 -0.42936  1.01430
H  -1.93664  1.25339  0.60185
H  -1.91171 -1.15323 -1.42658
H  -2.04273  0.51485 -1.97397
C   1.24785  1.10891  0.39738
C   1.97723  1.33885 -0.95344
H  -0.02363  0.59965  0.22819
H   0.93874  1.98512  1.01487
H   1.42557  0.96335 -1.83754
H   2.32629  2.36910 -1.16000
C   2.03041  0.10233  1.17331
O   1.62256 -0.44435  2.24323
```

[1]$_2$

```
SCF (BP86) Energy = -2792.92584571
Enthalpy 0K = -2791.392493
Enthalpy 298K = -2791.391548
Free Energy 298K = -2791.632781
Lowest Frequency = 5.4788 cm-1
Second Frequency = 9.5386 cm-1
SCF (BP86-D3BJ) Energy =  -2793.41822052
SCF (C6H6) Energy =  -2792.93887976
SCF (6-311++G**) Energy =  -5560.27200567
```

```
K   0.40897  2.45195 -0.44509
Si  6.19903  1.70559 -0.94643
Si  6.50144 -0.49225  1.28324
Al  3.64340  0.09607 -0.01166
O   6.96543  0.76401  0.23251
N   4.44909  1.70203 -0.60501
N   4.91962 -1.07286  0.74827
C   3.64496  2.86758 -0.74980
C   3.33466  3.68965  0.39235
C   2.49296  4.81163  0.23822
H   2.27277  5.43326  1.11428
C   1.96096  5.17190 -1.01039
H   1.33316  6.06402 -1.11277
C   2.27697  4.38816 -2.13048
H   1.88736  4.67593 -3.11446
C   3.10129  3.24538 -2.02821
C   3.91019  3.39304  1.77718
H   4.62137  2.55945  1.64702
C   4.68482  4.60002  2.35251
H   5.46028  4.95653  1.65453
H   5.17836  4.31939  3.29896
H   4.01499  5.45110  2.57087
C   2.81502  2.92917  2.76571
H   2.05031  3.71512  2.91330
H   3.25345  2.70906  3.75501
H   2.32450  2.01007  2.40044
C   3.41423  2.45752 -3.30105
H   4.00404  1.57856 -2.99257
```

C	4.26445	3.30058	-4.27994
H	3.70434	4.18212	-4.64033
H	4.54892	2.70210	-5.16312
H	5.18820	3.66832	-3.80310
C	2.14228	1.94272	-4.01113
H	1.55455	1.27258	-3.35934
H	2.41210	1.36279	-4.91052
H	1.48749	2.77077	-4.33854
C	6.66589	0.92787	-2.61474
H	7.75688	0.76403	-2.64657
H	6.39476	1.57110	-3.46806
H	6.17424	-0.04964	-2.75316
C	6.89089	3.46408	-0.84110
H	6.82762	3.85038	0.18883
H	6.34833	4.15956	-1.50344
H	7.95260	3.46614	-1.14040
C	6.53717	0.31079	3.00625
H	7.49818	0.83707	3.14020
H	6.43804	-0.42722	3.81858
H	5.72877	1.05297	3.11868
C	7.80509	-1.86238	1.19848
H	8.01534	-2.13817	0.15271
H	7.47012	-2.76890	1.73090
H	8.74786	-1.52346	1.66009
C	4.47057	-2.43318	0.83891
C	3.86770	-2.94057	2.03935
C	3.31352	-4.23737	2.04584
H	2.86966	-4.62201	2.97226
C	3.34219	-5.05483	0.90828
H	2.91452	-6.06350	0.93633
C	3.97139	-4.57994	-0.24931
H	4.03169	-5.22734	-1.13241
C	4.54886	-3.29642	-0.30500
C	3.87033	-2.14168	3.34455
H	4.16436	-1.10978	3.08757
C	4.92124	-2.72217	4.32193
H	4.65274	-3.75190	4.61918
H	4.98741	-2.11242	5.24091
H	5.92347	-2.76407	3.86398
C	2.49510	-2.07979	4.04589
H	1.72181	-1.63905	3.39284
H	2.55642	-1.45395	4.95327
H	2.14743	-3.07932	4.36350
C	5.27706	-2.88181	-1.58434
H	5.69494	-1.87763	-1.39644
C	6.45138	-3.83767	-1.89803
H	7.14263	-3.92719	-1.04378
H	7.02472	-3.47238	-2.76846
H	6.09147	-4.85326	-2.14232
C	4.32874	-2.77967	-2.80007
H	4.88542	-2.46169	-3.70011
H	3.52666	-2.04844	-2.60906
H	3.86405	-3.75641	-3.02838
K	-0.30602	-2.37049	0.39065
Si	-5.91300	-1.73830	-1.43315
Si	-6.59023	0.00979	1.06466
Al	-3.66797	0.11600	-0.31518
O	-6.85266	-1.16747	-0.14114
N	-4.19943	-1.57283	-0.98904
N	-5.08755	0.86878	0.68884
C	-3.29285	-2.66133	-1.08854
C	-2.44340	-2.80806	-2.23866
C	-1.54329	-3.89277	-2.31292
H	-0.91210	-4.00022	-3.20288
C	-1.46039	-4.85285	-1.29330
H	-0.77737	-5.70450	-1.38417
C	-2.28898	-4.71802	-0.16723
H	-2.24387	-5.47449	0.62537
C	-3.19464	-3.64296	-0.03912
C	-2.52876	-1.83533	-3.41445
H	-3.17698	-1.00305	-3.09044
C	-3.19258	-2.51114	-4.63710
H	-4.19244	-2.90414	-4.38774
H	-3.30509	-1.79160	-5.46711
H	-2.58436	-3.35740	-5.00446
C	-1.15521	-1.24847	-3.80493
H	-0.47049	-2.02152	-4.19790
H	-1.27521	-0.48689	-4.59515
H	-0.65782	-0.76913	-2.94333
C	-4.01888	-3.51587	1.24211
H	-4.84184	-2.81576	1.02247
C	-4.63358	-4.84687	1.72042
H	-3.86659	-5.55938	2.07455
H	-5.32031	-4.66398	2.56442
H	-5.20470	-5.34215	0.91759
C	-3.16737	-2.87754	2.36705
H	-2.81246	-1.87117	2.07561
H	-3.75327	-2.76267	3.29576
H	-2.29719	-3.51839	2.61354
C	-6.37867	-3.54703	-1.73786
H	-7.43954	-3.60751	-2.03423
H	-5.77285	-3.99038	-2.54603
H	-6.24424	-4.16069	-0.83301
C	-6.33449	-0.69195	-2.95756
H	-5.98557	0.34707	-2.83471
H	-5.85939	-1.10164	-3.86522
H	-7.42538	-0.66755	-3.12163
C	-8.08814	1.16907	1.09684
H	-8.95558	0.64320	1.53096
H	-7.89001	2.06470	1.70959
H	-8.36143	1.49816	0.08232
C	-6.59166	-0.96157	2.70028
H	-5.69691	-1.59485	2.81002
H	-6.63886	-0.28778	3.57176
H	-7.47923	-1.61734	2.73068
C	-4.87997	2.24956	1.04357
C	-5.20975	3.30293	0.12626
C	-4.86450	4.63089	0.44552
H	-5.10822	5.42733	-0.26779
C	-4.23148	4.96026	1.65001
H	-3.97759	6.00126	1.87912
C	-3.95955	3.94212	2.57070
H	-3.49979	4.19584	3.53374
C	-4.27542	2.59613	2.29669
C	-5.95081	3.04747	-1.18844
H	-6.21077	1.97488	-1.20273
C	-7.26125	3.86504	-1.27163
H	-7.05654	4.94846	-1.34089
H	-7.83430	3.58212	-2.17264
H	-7.90342	3.70409	-0.39073
C	-5.07522	3.33448	-2.42964
H	-4.17886	2.69422	-2.43143
H	-5.64516	3.14331	-3.35700
H	-4.75245	4.39157	-2.45183
C	-4.01128	1.55352	3.38486
H	-4.25096	0.56877	2.94922
C	-4.94257	1.78849	4.59814
H	-6.00248	1.82077	4.29503
H	-4.82269	0.98748	5.34962
H	-4.71412	2.74947	5.09319
C	-2.54044	1.51799	3.85486
H	-2.39957	0.74072	4.62748
H	-1.85575	1.29279	3.01991
H	-2.22895	2.48047	4.29900
C	-2.37799	1.39598	-1.09524
C	-1.73334	0.49025	0.05515
H	-2.48839	2.47097	-0.86928
H	-1.94146	1.23384	-2.09476
H	-1.49930	1.03414	0.98903
H	-0.82521	-0.02772	-0.29823
C	1.98877	-0.67830	-0.78809
C	1.79633	-0.17281	0.69960

```
H   1.51546 -0.02092 -1.54114
H   1.78715 -1.73290 -1.06266
H   0.84056  0.28893  1.01615
H   2.02214 -0.97757  1.42521
```

TS(1-J)

```
SCF (BP86) Energy = -2906.22902600
Enthalpy 0K = -2904.686913
Enthalpy 298K = -2904.685969
Free Energy 298K = -2904.933255
Lowest Frequency = -22.4527 cm-1
Second Frequency = 7.2360 cm-1
SCF (BP86-D3BJ) Energy =  -2906.73455049
SCF (C6H6) Energy =  -2906.24116680
SCF (6-311++G**) Energy =  -5673.61231606
```

```
K  -0.37764  2.46804  0.46214
Si -6.23895  1.71522  0.91296
Si -6.45578 -0.75732 -1.04821
Al -3.64815  0.07900  0.10111
O  -6.85555  0.29631  0.22363
N  -4.48973  1.71404  0.56430
N  -4.76913 -1.19376 -0.76127
C  -3.69574  2.90094  0.54254
C  -3.37018  3.54512 -0.70603
C  -2.53231  4.67974 -0.71526
H  -2.30145  5.15809 -1.67446
C  -2.01861  5.23233  0.46780
H  -1.39463  6.13250  0.44018
C  -2.35091  4.62820  1.68833
H  -1.97808  5.06580  2.62224
C  -3.16862  3.47706  1.75245
C  -3.92706  3.05918 -2.04339
H  -4.58698  2.20448 -1.81651
C  -4.77146  4.15218 -2.73878
H  -5.56623  4.53657 -2.07938
H  -5.24563  3.74734 -3.65005
H  -4.14758  5.01030 -3.04680
C  -2.81169  2.55897 -2.98801
H  -2.10432  3.37043 -3.24120
H  -3.24367  2.19392 -3.93616
H  -2.25522  1.72806 -2.52314
C  -3.46946  2.89836  3.13553
H  -4.08544  1.99676  2.98047
C  -4.26889  3.90186  3.99920
H  -3.66262  4.79283  4.24266
H  -4.57544  3.43749  4.95258
H  -5.17583  4.25317  3.48026
C  -2.18420  2.47308  3.88222
H  -1.63098  1.69059  3.33296
H  -2.42887  2.05633  4.87391
H  -1.50497  3.32933  4.04646
C  -6.74566  1.55694  2.73199
H  -7.82511  1.32842  2.76939
H  -6.57621  2.47958  3.30955
H  -6.20527  0.73019  3.22077
C  -7.06811  3.24647  0.16431
H  -7.05968  3.20716 -0.93663
H  -6.55893  4.17344  0.47998
H  -8.11839  3.30712  0.49651
C  -6.81468  0.17858 -2.66571
H  -7.84666  0.56972 -2.63011
H  -6.73548 -0.47705 -3.54846
H  -6.13481  1.03346 -2.81651
C  -7.63019 -2.23747 -0.95832
H  -7.72882 -2.59932  0.07692
H  -7.27381 -3.07158 -1.58689
H  -8.63225 -1.94756 -1.31740
C  -4.10938 -2.40868 -1.12616
C  -3.51071 -2.56765 -2.42454
C  -2.71826 -3.70113 -2.69877
H  -2.27613 -3.81258 -3.69605
C  -2.50291 -4.69851 -1.73651
H  -1.90393 -5.58518 -1.97572
C  -3.13033 -4.57603 -0.48829
H  -3.01428 -5.37830  0.25019
C  -3.93810 -3.46544 -0.16621
C  -3.74140 -1.55598 -3.54698
H  -4.21737 -0.67380 -3.08691
C  -4.71075 -2.14267 -4.60131
H  -4.26075 -3.01622 -5.10657
H  -4.95129 -1.39306 -5.37603
H  -5.65551 -2.47986 -4.14285
C  -2.43922 -1.08759 -4.23234
H  -1.73724 -0.64202 -3.50779
H  -2.66266 -0.32174 -4.99524
H  -1.92258 -1.91644 -4.74915
C  -4.65506 -3.46705  1.18469
H  -5.13698 -2.48030  1.29494
C  -5.75178 -4.55955  1.21411
H  -6.45660 -4.45723  0.37443
H  -6.32708 -4.50558  2.15539
H  -5.30505 -5.56852  1.15338
C  -3.69748 -3.67120  2.37774
H  -4.25517 -3.61922  3.32910
H  -2.91300 -2.89865  2.39671
H  -3.20965 -4.66265  2.34454
K   0.03509 -2.56633 -0.41729
Si  5.91400 -1.82446  1.29664
Si  6.62980  0.07168 -1.07875
Al  3.69596  0.12836  0.24724
O   6.87685 -1.16976  0.06366
N   4.20615 -1.59432  0.85492
N   5.15596  0.94617 -0.63853
C   3.28681 -2.67443  0.93175
C   2.48487 -2.88026  2.10596
C   1.60699 -3.98416  2.17302
H   1.02046 -4.14133  3.08616
C   1.49967 -4.90534  1.12034
H   0.84692 -5.78094  1.20977
C   2.26659 -4.70044 -0.03950
H   2.20037 -5.42355 -0.86131
C   3.14412 -3.60204 -0.16119
C   2.59278 -1.95019  3.31379
H   3.26239 -1.12468  3.01765
C   3.22744 -2.67766  4.52166
H   4.21369 -3.10054  4.26703
H   3.36170 -1.98175  5.36835
H   2.58901 -3.50977  4.86978
C   1.23001 -1.34099  3.70901
H   0.52132 -2.11137  4.06303
H   1.35808 -0.61290  4.52913
H   0.75800 -0.81641  2.85989
C   3.90230 -3.39332 -1.47130
H   4.74076 -2.71453 -1.24313
C   4.48159 -4.68845 -2.07434
H   3.69222 -5.37066 -2.43948
H   5.12472 -4.44752 -2.93792
H   5.09048 -5.24255 -1.34051
C   2.99872 -2.66953 -2.50003
H   2.64542 -1.69919 -2.10334
H   3.54508 -2.46823 -3.43842
H   2.12567 -3.29968 -2.76491
C   6.34199 -3.66210  1.45266
H   7.40112 -3.76758  1.74282
H   5.72641 -4.15896  2.22128
H   6.19695 -4.19597  0.49991
C   6.35897 -0.92403  2.90602
H   6.02641  0.12697  2.88047
H   5.88463 -1.40654  3.77741
H   7.45134 -0.93210  3.06204
C   8.15205  1.19852 -1.06663
```

```
H   9.01074  0.67291 -1.51796
H   7.97348  2.12157 -1.64356
H   8.42738  1.48246 -0.03889
C   6.57285 -0.81542 -2.76098
H   5.65194 -1.40856 -2.88300
H   6.62760 -0.10054 -3.59868
H   7.43368 -1.50158 -2.84305
C   5.00685  2.35869 -0.87340
C   5.35362  3.31174  0.14162
C   5.08773  4.67840 -0.07137
H   5.34508  5.39698  0.71600
C   4.51652  5.14208 -1.26243
H   4.32526  6.21097 -1.40925
C   4.22054  4.22056 -2.27283
H   3.80315  4.57815 -3.22208
C   4.45579  2.84091 -2.10573
C   6.01980  2.90181  1.45631
H   6.23964  1.82330  1.37613
C   7.35350  3.64987  1.68497
H   7.18899  4.72971  1.85077
H   7.86657  3.25594  2.58034
H   8.03460  3.54653  0.82448
C   5.08566  3.10189  2.67186
H   4.16487  2.50752  2.55626
H   5.59028  2.79048  3.60464
H   4.80596  4.16542  2.78537
C   4.15318  1.90694 -3.27864
H   4.32571  0.87786 -2.92079
C   5.11513  2.18091 -4.45896
H   6.17054  2.11860 -4.14477
H   4.95520  1.45472 -5.27621
H   4.95615  3.19258 -4.87389
C   2.68940  1.99868 -3.76287
H   2.50828  1.28667 -4.58800
H   1.98354  1.76038 -2.94952
H   2.44588  3.00809 -4.14065
C   2.36368  1.37085  1.03407
C   1.77689  0.47395 -0.15335
H   2.53392  2.43391  0.78773
H   1.87285  1.25250  2.01533
H   1.43079  1.00729 -1.05842
H   0.96906 -0.18001  0.21641
C  -1.97405 -0.56429  0.96643
C  -1.76678 -0.12400 -0.53274
H  -1.59411  0.18229  1.68861
H  -1.65824 -1.56421  1.32742
H  -0.81679  0.33821 -0.85886
H  -1.96504 -0.95715 -1.23390
C  -3.93889 -0.67465  3.94638
O  -3.01169 -0.85927  4.60347
```

J

```
SCF (BP86) Energy = -2906.23201208
Enthalpy 0K = -2904.689150
Enthalpy 298K = -2904.688206
Free Energy 298K = -2904.932724
Lowest Frequency = 6.4035 cm-1
Second Frequency = 10.1660 cm-1
SCF (BP86-D3BJ) Energy =  -2906.74206709
SCF (C6H6) Energy =  -2906.24413082
SCF (6-311++G**) Energy =  -5673.61224545
```

```
K   0.36289  2.48514 -0.58255
Si  6.24765  1.79986 -0.75825
Si  6.39451 -0.86482  0.95590
Al  3.70477  0.03347 -0.31609
O   6.77743  0.27453 -0.24845
N   4.47339  1.76407 -0.56670
N   4.70916 -1.28691  0.63747
C   3.67056  2.94259 -0.54242
C   3.28929  3.55658  0.70429
C   2.43346  4.67894  0.70154
H   2.15506  5.13361  1.65959
C   1.95729  5.24670 -0.48977
H   1.31519  6.13428 -0.46858
C   2.35096  4.67616 -1.70890
H   2.01059  5.12818 -2.64836
C   3.19349  3.54320 -1.76232
C   3.79630  3.04816  2.05331
H   4.50123  2.22773  1.83492
C   4.54828  4.14853  2.83704
H   5.36013  4.59587  2.24164
H   4.98948  3.72690  3.75722
H   3.86891  4.96345  3.14484
C   2.65137  2.46818  2.91210
H   1.89110  3.23811  3.14104
H   3.04035  2.09541  3.87563
H   2.16780  1.62729  2.38900
C   3.57368  3.00403 -3.14204
H   4.27022  2.16729 -2.97794
C   4.29305  4.07509 -3.99374
H   3.61356  4.90330 -4.26357
H   4.66565  3.63341 -4.93398
H   5.15018  4.51351 -3.45650
C   2.35052  2.45331 -3.91065
H   1.86666  1.62361 -3.36608
H   2.65513  2.05628 -4.89401
H   1.59642  3.24188 -4.09090
C   6.92939  1.95427 -2.52160
H   8.02296  1.80679 -2.48185
H   6.74170  2.94778 -2.96048
H   6.51172  1.18928 -3.19502
C   7.02750  3.18322  0.27837
H   6.90563  3.00631  1.35836
H   6.57813  4.16183  0.03579
H   8.10748  3.24624  0.06100
C   6.76186 -0.02227  2.62236
H   7.79189  0.37500  2.59773
H   6.69362 -0.72110  3.47165
H   6.07862  0.82052  2.82055
C   7.58686 -2.32047  0.76271
H   7.63711 -2.65438 -0.28544
H   7.27654 -3.17635  1.38651
H   8.60132 -2.02304  1.07843
C   4.01876 -2.47689  1.02389
C   3.40393 -2.60299  2.32030
C   2.58673 -3.71573  2.60887
H   2.13402 -3.79750  3.60427
C   2.36061 -4.72991  1.66749
H   1.74144 -5.59910  1.91867
C   3.00487 -4.64499  0.42517
H   2.88322 -5.46023 -0.29795
C   3.83680 -3.55682  0.08863
C   3.63830 -1.58225  3.43353
H   4.14341 -0.71713  2.97285
C   4.56778 -2.18179  4.51653
H   4.07569 -3.02627  5.03196
H   4.82382 -1.42547  5.27955
H   5.50603 -2.56570  4.08261
C   2.33397 -1.07777  4.08936
H   1.65219 -0.62843  3.34822
H   2.55907 -0.30632  4.84591
H   1.79309 -1.88943  4.60865
C   4.56862 -3.61543 -1.25313
H   5.08444 -2.64704 -1.37834
C   5.63852 -4.73452 -1.23904
H   6.34176 -4.61832 -0.39972
H   6.21905 -4.72672 -2.17851
H   5.16737 -5.72971 -1.14580
C   3.62314 -3.82665 -2.45619
H   4.19129 -3.79049 -3.40155
H   2.84202 -3.05116 -2.50496
```

```
H   3.12723 -4.81326 -2.41146
K  -0.04512 -2.56274  0.36972
Si -5.92502 -1.81463 -1.28990
Si -6.60192  0.09634  1.08535
Al -3.68455  0.13145 -0.27887
O  -6.86851 -1.14965 -0.04745
N  -4.21043 -1.59145 -0.87094
N  -5.12864  0.96050  0.62308
C  -3.29823 -2.67739 -0.95279
C  -2.51124 -2.89415 -2.13512
C  -1.63901 -4.00229 -2.20515
H  -1.06360 -4.16794 -3.12382
C  -1.52381 -4.91735 -1.14808
H  -0.87496 -5.79558 -1.23903
C  -2.27737 -4.70276  0.01864
H  -2.20500 -5.42129  0.84388
C  -3.14866 -3.59984  0.14353
C  -2.63020 -1.97219 -3.34826
H  -3.29089 -1.14059 -3.04897
C  -3.28577 -2.70493 -4.54173
H  -4.27101 -3.11975 -4.27054
H  -3.42731 -2.01475 -5.39192
H  -2.65718 -3.54364 -4.89190
C  -1.26965 -1.37373 -3.76733
H  -0.57020 -2.15084 -4.12555
H  -1.40521 -0.65092 -4.59100
H  -0.78297 -0.84598 -2.92856
C  -3.89229 -3.38049  1.46029
H  -4.73117 -2.70060  1.23697
C  -4.46888 -4.67001  2.07771
H  -3.67768 -5.35212  2.43902
H  -5.10232 -4.42166  2.94635
H  -5.08691 -5.22704  1.35381
C  -2.97574 -2.65308  2.47490
H  -2.62361 -1.68620  2.06866
H  -3.51146 -2.44405  3.41771
H  -2.10193 -3.28423  2.73459
C  -6.36449 -3.65061 -1.43220
H  -7.42796 -3.75172 -1.70767
H  -5.76190 -4.15396 -2.20682
H  -6.20941 -4.18119 -0.47919
C  -6.38596 -0.91864 -2.89727
H  -6.04807  0.13084 -2.88047
H  -5.92527 -1.40705 -3.77269
H  -7.48028 -0.92203 -3.03923
C  -8.11842  1.23099  1.08520
H  -8.97465  0.71137  1.54801
H  -7.92910  2.15530  1.65665
H  -8.40372  1.51236  0.05947
C  -6.53067 -0.78243  2.77140
H  -5.61199 -1.38050  2.88583
H  -6.57167 -0.06319  3.60613
H  -7.39455 -1.46305  2.86663
C  -4.96731  2.37301  0.84950
C  -5.31976  3.32383 -0.16573
C  -5.03880  4.68919  0.03636
H  -5.30051  5.40593 -0.75125
C  -4.44791  5.15403  1.21736
H  -4.24529  6.22194  1.35589
C  -4.14819  4.23541  2.22935
H  -3.71664  4.59446  3.17170
C  -4.39787  2.85707  2.07277
C  -6.01007  2.91358 -1.46790
H  -6.23714  1.83721 -1.37953
C  -7.34138  3.67154 -1.67746
H  -7.17084  4.74926 -1.85077
H  -7.87263  3.27800 -2.56234
H  -8.00871  3.57750 -0.80522
C  -5.09513  3.10112 -2.69987
H  -4.17789  2.49894 -2.59775
H  -5.61829  2.79086 -3.62274
H  -4.80806  4.16172 -2.82222
C  -4.09236  1.92700  3.24801
H  -4.27705  0.89753  2.89750
C  -5.04181  2.21595  4.43492
H  -6.10036  2.16092  4.13007
H  -4.88107  1.49307  5.25494
H  -4.87062  3.22858  4.84258
C  -2.62383  2.00841  3.71918
H  -2.44232  1.30004  4.54735
H  -1.92699  1.75845  2.90159
H  -2.36785  3.01784  4.08857
C  -2.36309  1.36170 -1.09956
C  -1.75932  0.48385  0.09434
H  -2.52442  2.43077 -0.87388
H  -1.89002  1.21859 -2.08616
H  -1.41726  1.03455  0.99052
H  -0.94318 -0.16389 -0.26767
C   1.91764 -0.57002 -1.09642
C   1.80832 -0.13245  0.38556
H   1.52865  0.16227 -1.82528
H   1.65006 -1.58624 -1.45203
H   0.89967  0.38800  0.74082
H   1.99435 -0.96456  1.08714
C   4.33737 -0.49516 -2.40352
O   4.10694 -0.69659 -3.53002
```

TS(J-G)

```
SCF (BP86) Energy = -2906.22935179
Enthalpy 0K = -2904.687271
Enthalpy 298K = -2904.686327
Free Energy 298K = -2904.931268
Lowest Frequency = -216.1503 cm-1
Second Frequency = 5.3287 cm-1
SCF (BP86-D3BJ) Energy =  -2906.73884388
SCF (C6H6) Energy =  -2906.24196281
SCF (6-311++G**) Energy =
```

```
K   0.36727  2.50295 -0.86437
Si  6.29575  1.71792 -0.70947
Si  6.35575 -0.95675  1.04233
Al  3.76077  0.00081 -0.30773
O   6.75093  0.18289 -0.15596
N   4.52151  1.74212 -0.53920
N   4.67021 -1.35363  0.69520
C   3.72319  2.91919 -0.57678
C   3.27209  3.54332  0.64127
C   2.41795  4.66526  0.58415
H   2.08863  5.12764  1.52225
C   2.01134  5.22650 -0.63561
H   1.37382  6.11740 -0.65657
C   2.47030  4.64457 -1.82683
H   2.18469  5.09086 -2.78728
C   3.30783  3.50684 -1.82630
C   3.71734  3.05136  2.01751
H   4.40279  2.20438  1.84171
C   4.48301  4.14799  2.79357
H   5.33522  4.53842  2.21458
H   4.87057  3.74404  3.74534
H   3.82664  5.00124  3.04180
C   2.52852  2.53426  2.85506
H   1.78958  3.33434  3.04534
H   2.87539  2.16872  3.83701
H   2.02144  1.70019  2.34350
C   3.75295  2.95186 -3.18026
H   4.41125  2.09303 -2.97640
C   4.55292  4.00174 -3.98578
H   3.91376  4.84924 -4.29224
H   4.96795  3.55053 -4.90344
H   5.38843  4.41567 -3.39748
C   2.56325  2.43803 -4.02359
H   2.02218  1.62226 -3.51278
```

```
H   2.91732  2.02972 -4.98540
H   1.84616  3.24854 -4.25162
C   7.00832  1.77444 -2.46471
H   8.09078  1.56545 -2.40402
H   6.88258  2.75603 -2.94909
H   6.55193  1.00316 -3.10586
C   7.10361  3.09376  0.31488
H   6.93545  2.94970  1.39385
H   6.70218  4.08271  0.03273
H   8.19217  3.10870  0.13527
C   6.69175 -0.12143  2.71799
H   7.73160  0.24994  2.72915
H   6.57573 -0.81610  3.56571
H   6.02278  0.73859  2.88955
C   7.55139 -2.41050  0.86240
H   7.59735 -2.75981 -0.18079
H   7.24926 -3.25804  1.50137
H   8.56594 -2.10290  1.16830
C   3.93433 -2.51442  1.08745
C   3.25296 -2.57909  2.35421
C   2.40108 -3.66540  2.64326
H   1.89799 -3.70031  3.61704
C   2.20399 -4.71196  1.73196
H   1.55785 -5.56074  1.98481
C   2.90803 -4.68437  0.52013
H   2.80348 -5.52250 -0.17894
C   3.77369 -3.62344  0.18268
C   3.44484 -1.51861  3.43760
H   4.00091 -0.68547  2.97623
C   4.28668 -2.09006  4.60400
H   3.74088 -2.89874  5.12277
H   4.51333 -1.30624  5.34830
H   5.24029 -2.51495  4.24917
C   2.11196 -0.96011  3.98274
H   1.49014 -0.53256  3.17840
H   2.30123 -0.16059  4.71955
H   1.52057 -1.73883  4.49730
C   4.54884 -3.73651 -1.13109
H   5.07634 -2.77818 -1.27888
C   5.60545 -4.86487 -1.04379
H   6.28559 -4.72568 -0.18916
H   6.21290 -4.89821 -1.96551
H   5.11984 -5.85074 -0.92790
C   3.63689 -3.98584 -2.35348
H   4.23004 -3.96618 -3.28356
H   2.85193 -3.21856 -2.44381
H   3.14851 -4.97568 -2.29697
K  -0.20219 -2.50718  0.24101
Si -5.99342 -1.71379 -1.23457
Si -6.50311  0.18867  1.18792
Al -3.65531  0.15890 -0.33311
O  -6.85543 -1.04352  0.06356
N  -4.25525 -1.54865 -0.89429
N  -5.02234  1.00777  0.66868
C  -3.37846 -2.66006 -1.01113
C  -2.62400 -2.88184 -2.21382
C  -1.77142 -4.00301 -2.31063
H  -1.21657 -4.16979 -3.24151
C  -1.64752 -4.92724 -1.26254
H  -1.01046 -5.81178 -1.37201
C  -2.37648 -4.71349 -0.08054
H  -2.29741 -5.43987  0.73698
C  -3.22720 -3.59783  0.07174
C  -2.76072 -1.95177 -3.41896
H  -3.38095 -1.09981 -3.09142
C  -3.49225 -2.66045 -4.58280
H  -4.48034 -3.03793 -4.27041
H  -3.64284 -1.96615 -5.42809
H  -2.90973 -3.52227 -4.95564
C  -1.40103 -1.39789 -3.89745
H  -0.74614 -2.19513 -4.29347
H  -1.55017 -0.66522 -4.71007
H  -0.85574 -0.89377 -3.08014
C  -3.93888 -3.37859  1.40665
H  -4.77122 -2.68245  1.21069
C  -4.52259 -4.66500  2.02405
H  -3.73496 -5.36246  2.36287
H  -5.13473 -4.41528  2.90740
H  -5.16324 -5.20564  1.30747
C  -2.98871 -2.67794  2.40913
H  -2.63689 -1.70689  2.01224
H  -3.49716 -2.47851  3.36883
H  -2.11528 -3.32159  2.63637
C  -6.49683 -3.53182 -1.38957
H  -7.57385 -3.59453 -1.62009
H  -5.94461 -4.03767 -2.19932
H  -6.31764 -4.08612 -0.45460
C  -6.49348 -0.77138 -2.80283
H  -6.12546  0.26784 -2.77770
H  -6.08062 -1.25322 -3.70546
H  -7.59211 -0.74157 -2.90171
C  -7.98289  1.36923  1.25531
H  -8.83070  0.87596  1.76057
H  -7.73919  2.28821  1.81466
H  -8.30917  1.65712  0.24380
C  -6.39818 -0.69858  2.86764
H  -5.50068 -1.33317  2.94531
H  -6.37894  0.01848  3.70505
H  -7.28439 -1.34454  2.99434
C  -4.79590  2.40871  0.91194
C  -5.16405  3.39382 -0.06460
C  -4.80782  4.74087  0.14311
H  -5.08205  5.48400 -0.61528
C  -4.12720  5.15568  1.29418
H  -3.86673  6.21037  1.43746
C  -3.81576  4.20599  2.27360
H  -3.31676  4.52740  3.19608
C  -4.13972  2.84405  2.10997
C  -5.95591  3.04376 -1.32650
H  -6.21851  1.97458 -1.24928
C  -7.26716  3.85816 -1.42233
H  -7.06457  4.93253 -1.58122
H  -7.87463  3.50982 -2.27661
H  -7.87510  3.76696 -0.50761
C  -5.12507  3.23095 -2.61661
H  -4.22976  2.58908 -2.59973
H  -5.72704  2.97014 -3.50602
H  -4.80285  4.28223 -2.73185
C  -3.82206  1.88068  3.25489
H  -4.07998  0.86813  2.90154
C  -4.69249  2.20065  4.49341
H  -5.76664  2.20761  4.24315
H  -4.52958  1.45658  5.29378
H  -4.44589  3.19564  4.90578
C  -2.32926  1.87642  3.64986
H  -2.14586  1.15255  4.46413
H  -1.68975  1.59487  2.79655
H  -1.99866  2.86684  4.01121
C  -2.38160  1.37628 -1.23519
C  -1.69984  0.49764 -0.08317
H  -2.50523  2.45140 -1.01521
H  -1.98320  1.20754 -2.24986
H  -1.33791  1.06009  0.79779
H  -0.87274 -0.11005 -0.48929
C   1.85513 -0.59928 -1.30434
C   1.84165 -0.16876  0.17342
H   1.51932  0.15886 -2.03163
H   1.51236 -1.59665 -1.64284
H   1.02281  0.47250  0.54995
H   1.90908 -1.02520  0.86786
C   4.02026 -0.60074 -2.25960
O   3.94544 -0.87457 -3.40582
```

III
SCF (BP86) Energy = -2906.28199843
Enthalpy 0K = -2904.736400
Enthalpy 298K = -2904.735456
Free Energy 298K = -2904.980016
Lowest Frequency = 4.7030 cm-1
Second Frequency = 10.2748 cm-1
SCF (BP86-D3BJ) Energy = -2906.78400770
SCF (C6H6) Energy = -2906.29627742
SCF (6-311++G**) Energy = -5673.65841524

3
SCF (BP86) Energy = -2319.47413323
Enthalpy 0K = -2318.328714
Enthalpy 298K = -2318.327770
Free Energy 298K = -2318.508491
Lowest Frequency = 5.2810 cm-1
Second Frequency = 12.1316 cm-1
SCF (BP86-D3BJ) Energy = -2319.79746549
SCF (C6H6) Energy = -2319.48542493
SCF (6-311++G**) Energy = -3703.40750790

K	-3.38366	-0.17847	0.06686
Si	4.00116	1.63725	-0.75909
Si	4.15468	-1.16968	0.60806
Al	1.30289	0.09943	-0.01035
O	4.74470	0.25128	-0.11472
O	-3.89967	-0.59935	-2.70882
O	-3.66129	-2.86537	-0.98631
O	-3.23869	-2.40864	1.78293
O	-3.96537	0.09894	2.91496
O	-4.04722	2.35249	1.15169
O	-4.68141	1.88936	-1.57803
N	2.32408	1.68864	-0.20178
N	2.47131	-1.39455	0.11920
C	1.67848	2.92289	0.13537
C	1.02831	3.71463	-0.86714
C	0.35659	4.89775	-0.49939
H	-0.12727	5.49899	-1.27973
C	0.30127	5.32610	0.83149
H	-0.22288	6.25079	1.09908
C	0.94442	4.56232	1.81315
H	0.91810	4.89702	2.85747
C	1.63905	3.37981	1.49511
C	1.05379	3.32119	-2.34409
H	1.57476	2.35132	-2.40606
C	-0.36535	3.12180	-2.92034
H	-0.95132	4.05924	-2.89014
H	-0.90930	2.35160	-2.34737
H	-0.31583	2.79355	-3.97452
C	1.83305	4.35325	-3.19090
H	2.85656	4.50004	-2.80687
H	1.33378	5.33930	-3.18019
H	1.90539	4.02641	-4.24406
C	2.33257	2.61450	2.62182
H	2.92344	1.81681	2.13967
C	1.30884	1.93582	3.56083
H	0.66946	1.24202	2.98872
H	0.66665	2.68962	4.05382
H	1.82483	1.36553	4.35518
C	3.30150	3.50564	3.43021
H	2.76337	4.28845	3.99505
H	4.03108	4.01158	2.77589
H	3.86129	2.89847	4.16373
C	4.21513	1.47795	-2.64359
H	3.58955	0.66614	-3.05104
H	5.26825	1.24293	-2.87671
H	3.94960	2.40971	-3.17026
C	4.96666	3.15665	-0.16462
H	5.97954	3.15627	-0.60212
H	5.06818	3.15390	0.93249
H	4.46467	4.09358	-0.45954
C	5.23917	-2.60923	0.02053
H	6.26008	-2.50398	0.42540
H	5.30799	-2.62276	-1.07916
H	4.83668	-3.58208	0.34950
C	4.40499	-0.91992	2.47770
H	3.71159	-0.15890	2.87323
H	5.43482	-0.57330	2.67203
H	4.24588	-1.85194	3.04497
C	1.94118	-2.67616	-0.23099
C	1.87559	-3.09216	-1.60326
C	1.29775	-4.33425	-1.92906
H	1.25415	-4.63794	-2.98234
C	0.79965	-5.19696	-0.94463
H	0.37212	-6.16835	-1.21879
C	0.87926	-4.80586	0.39733
H	0.51292	-5.48339	1.17916
C	1.43264	-3.56486	0.77341
C	2.42844	-2.22537	-2.73407
H	2.93049	-1.36845	-2.25307
C	1.30005	-1.66563	-3.63019
H	0.59760	-1.06414	-3.02882
H	0.74140	-2.48426	-4.12175
H	1.71754	-1.02333	-4.42723
C	3.47616	-2.97593	-3.58620
H	3.02327	-3.81528	-4.14475
H	4.28559	-3.39133	-2.96282
H	3.92972	-2.29412	-4.32740
C	1.49929	-3.21408	2.25949
H	1.84754	-2.16907	2.32084
C	0.11998	-3.29147	2.95017
H	-0.25761	-4.33090	2.98442
H	-0.62774	-2.67453	2.42422
H	0.19271	-2.93726	3.99471
C	2.51187	-4.11583	3.00273
H	3.51479	-4.06170	2.54745
H	2.19457	-5.17435	2.97480
H	2.60104	-3.82036	4.06398
C	-0.50047	0.22461	0.80242
H	-0.85952	1.25831	0.95511
H	-0.96579	-0.46158	1.53367
C	-0.51401	-0.23320	-0.72738
H	-1.10335	0.36792	-1.44387
H	-0.74006	-1.30770	-0.85796
C	-4.10158	-1.94274	-3.15414
H	-3.84194	-2.03814	-4.23044
H	-5.16621	-2.23726	-3.02957
C	-3.20993	-2.86860	-2.34536
H	-3.27376	-3.89206	-2.77420
H	-2.15431	-2.53283	-2.40577
C	-2.89723	-3.76104	-0.16627
H	-1.82415	-3.48092	-0.16460
H	-2.97634	-4.80059	-0.55067
C	-3.45358	-3.71648	1.24556
H	-4.53827	-3.95883	1.23917
H	-2.93019	-4.47803	1.86134
C	-3.69208	-2.28069	3.13113
H	-3.23087	-3.06132	3.77288
H	-4.79622	-2.39784	3.18538
C	-3.27704	-0.91661	3.65421
H	-3.52977	-0.84867	4.73481
H	-2.17953	-0.79652	3.54498
C	-3.53654	1.41106	3.29942
H	-2.45821	1.54528	3.07334
H	-3.68720	1.56621	4.38971
C	-4.35938	2.43883	2.54340
H	-5.44283	2.25491	2.71071
H	-4.11782	3.45078	2.93361
C	-4.78256	3.29229	0.36883
H	-4.59669	4.32872	0.72407

```
H  -5.87385  3.09395  0.44536
C  -4.33303  3.18576 -1.07754
H  -4.83823  3.98009 -1.66868
H  -3.23761  3.34902 -1.14718
C  -4.33204  1.74178 -2.95865
H  -3.24423  1.90667 -3.10370
H  -4.87918  2.48361 -3.58016
C  -4.70406  0.34232 -3.41658
H  -5.78418  0.15216 -3.23343
H  -4.52557  0.26763 -4.51123
```

K

```
SCF (BP86) Energy = -1368.13206084
Enthalpy 0K = -1367.370840
Enthalpy 298K = -1367.369896
Free Energy 298K = -1367.495239
Lowest Frequency = 13.6829 cm-1
Second Frequency = 22.2866 cm-1
SCF (BP86-D3BJ) Energy =  -1368.35194230
SCF (C6H6) Energy =  -1368.16479501
SCF (6-311++G**) Energy =  -2180.16954365

Si -1.38045  0.70077  1.91365
Si  1.38059 -0.70421  1.91233
Al -0.00003 -0.00157 -0.90734
O   0.00062 -0.00121  2.62119
N  -1.53002  0.15585  0.24900
N   1.53047 -0.15720  0.24848
C  -2.81400 -0.16908 -0.29334
C  -3.64512  0.83902 -0.88303
C  -4.89598  0.48636 -1.42755
H  -5.51982  1.27071 -1.87530
C  -5.35416 -0.83506 -1.41820
H  -6.32757 -1.09187 -1.85267
C  -4.54559 -1.82492 -0.84648
H  -4.89390 -2.86538 -0.83754
C  -3.29386 -1.52160 -0.27917
C  -3.21181  2.30401 -0.94414
H  -2.20426  2.35509 -0.49842
C  -3.10104  2.81343 -2.39759
H  -4.07670  2.77678 -2.91697
H  -2.38261  2.20462 -2.96869
H  -2.75105  3.86197 -2.41598
C  -4.15770  3.21083 -0.12423
H  -4.23703  2.87326  0.92363
H  -5.17927  3.21217 -0.54779
H  -3.79718  4.25607 -0.12175
C  -2.46557 -2.65491  0.32400
H  -1.63834 -2.17359  0.87340
C  -1.84032 -3.53755 -0.78065
H  -1.20536 -2.92283 -1.44219
H  -2.62672 -4.01944 -1.39159
H  -1.21940 -4.33896 -0.33729
C  -3.26646 -3.51496  1.32567
H  -4.07153 -4.08543  0.82660
H  -3.73527 -2.89736  2.11128
H  -2.60285 -4.24941  1.81715
C  -1.15197  2.58109  2.13095
H  -0.36429  2.96444  1.46134
H  -0.85765  2.80934  3.17053
H  -2.08193  3.13129  1.90838
C  -2.87934  0.16832  2.95494
H  -2.79318  0.57315  3.97826
H  -2.93919 -0.92997  3.02373
H  -3.82279  0.53379  2.51555
C   2.88011 -0.17489  2.95423
H   2.79122 -0.57733  3.97826
H   2.94378  0.92332  3.02084
H   3.82283 -0.54454  2.51679
C   1.14952 -2.58459  2.12606
H   0.36296 -2.96573  1.45387
H   0.85263 -2.81461  3.16452
H   2.07936 -3.13538  1.90441
C   2.81425  0.17036 -0.29258
C   3.29176  1.52367 -0.27702
C   4.54386  1.82945 -0.84218
H   4.89040  2.87049 -0.83207
C   5.35499  0.84128 -1.41319
H   6.32871  1.09994 -1.84586
C   4.89888 -0.48085 -1.42434
H   5.52464 -1.26386 -1.87180
C   3.64772 -0.83590 -0.88210
C   2.46030  2.65515  0.32516
H   1.63340  2.17195  0.87341
C   1.83462  3.53632 -0.78043
H   1.20208  2.92011 -1.44292
H   2.62080  4.02000 -1.39023
H   1.21114  4.33627 -0.33804
C   3.25784  3.51690  1.32800
H   4.06252  4.08903  0.83020
H   3.72668  2.90030  2.11438
H   2.59195  4.24997  1.81844
C   3.21634 -2.30134 -0.94577
H   2.20773 -2.35407 -0.50265
C   3.10990 -2.80922 -2.40010
H   4.08710 -2.77194 -2.91654
H   2.39322 -2.19985 -2.97281
H   2.76006 -3.85776 -2.42067
C   4.16122 -3.20802 -0.12456
H   4.23717 -2.87185  0.92400
H   5.18395 -3.20729 -0.54531
H   3.80217 -4.25376 -0.12454
C  -0.24525 -0.76151 -2.70982
H  -1.30834 -0.84318 -2.99920
H   0.34118 -1.46927 -3.32088
C   0.24655  0.75381 -2.71157
H  -0.33910  1.45965 -3.32558
H   1.31007  0.83497 -2.99953
```

TS(K-L)

```
SCF (BP86) Energy = -1481.43601885
Enthalpy 0K = -1480.665586
Enthalpy 298K = -1480.664642
Free Energy 298K = -1480.793550
Lowest Frequency = -19.8598 cm-1
Second Frequency = 19.7212 cm-1
SCF (BP86-D3BJ) Energy =  -1481.66784272
SCF (C6H6) Energy =  -1481.46842510
SCF (6-311++G**) Energy =  -2293.51018332

Si  1.19673 -1.40346  1.69017
Si -1.30716  0.32607  2.06273
Al -0.06893 -0.13489 -0.86058
O  -0.06291 -0.70910  2.60790
N   1.47853 -0.40392  0.26387
N  -1.55620  0.08402  0.33616
C   2.80072 -0.04982 -0.15735
C   3.47924 -0.80571 -1.17351
C   4.75462 -0.40412 -1.61594
H   5.25426 -0.99318 -2.39492
C   5.40036  0.71618 -1.08335
H   6.38846  1.01963 -1.44869
C   4.76469  1.43264 -0.06466
H   5.26822  2.30270  0.37532
C   3.49259  1.07003  0.41965
C   2.89156 -2.09582 -1.74986
H   1.83632 -2.15162 -1.43105
C   2.91082 -2.13613 -3.29265
H   3.94132 -2.13261 -3.69420
H   2.37383 -1.27378 -3.71977
H   2.42061 -3.05695 -3.65694
C   3.63555 -3.32565 -1.17685
```

```
H   3.61351 -3.33381 -0.07376
H   4.69758 -3.32433 -1.48586
H   3.17927 -4.26650 -1.53612
C   2.91177  1.89405  1.57054
H   1.97508  1.40023  1.87878
C   2.56841  3.33711  1.13965
H   1.83118  3.34816  0.32031
H   3.47265  3.87011  0.79109
H   2.14979  3.90826  1.98905
C   3.86307  1.93819  2.78992
H   4.77665  2.51949  2.56643
H   4.18100  0.93059  3.10283
H   3.36638  2.42636  3.64831
C   0.65467 -3.17245  1.24211
H  -0.05581 -3.16418  0.40019
H   0.17421 -3.66951  2.10290
H   1.52612 -3.77772  0.93714
C   2.70827 -1.54225  2.83508
H   2.60319 -2.43474  3.47623
H   2.79190 -0.66132  3.48979
H   3.64419 -1.63945  2.25958
C  -2.85818 -0.10128  3.07563
H  -2.71265  0.20813  4.12540
H  -3.05642 -1.18459  3.06320
H  -3.74967  0.41632  2.68344
C  -0.79270  2.09966  2.52265
H  -0.02688  2.48145  1.82963
H  -0.38545  2.13616  3.54837
H  -1.65907  2.78179  2.47336
C  -2.88289  0.01131 -0.20106
C  -3.59033 -1.23621 -0.26965
C  -4.87666 -1.27858 -0.84089
H  -5.39860 -2.24186 -0.89901
C  -5.50424 -0.12840 -1.33234
H  -6.50732 -0.18410 -1.77156
C  -4.82840  1.09307 -1.25033
H  -5.31239  2.00390 -1.62518
C  -3.53502  1.18708 -0.69945
C  -2.98521 -2.54019  0.25056
H  -2.08651 -2.26260  0.82666
C  -2.53144 -3.45216 -0.91220
H  -1.77196 -2.93767 -1.52409
H  -3.38701 -3.71680 -1.56142
H  -2.09765 -4.39322 -0.52516
C  -3.93992 -3.30748  1.19238
H  -4.82095 -3.70056  0.65219
H  -4.31166 -2.66810  2.01116
H  -3.42033 -4.17364  1.64078
C  -2.87387  2.56388 -0.63573
H  -1.84939  2.41272 -0.25563
C  -2.76464  3.22210 -2.02754
H  -3.76064  3.41993 -2.46570
H  -2.20717  2.57463 -2.72282
H  -2.23241  4.18778 -1.95882
C  -3.62287  3.49827  0.34237
H  -3.69923  3.05369  1.34952
H  -4.65242  3.70044 -0.00760
H  -3.10299  4.46948  0.43465
C   0.10784  0.47446 -2.75071
H   1.15953  0.52682 -3.08182
H  -0.49516  1.17337 -3.35113
C  -0.43933 -0.99467 -2.60521
H   0.10564 -1.77471 -3.16183
H  -1.51294 -1.04892 -2.85381
C   0.63453  3.08271 -1.70997
O   0.96413  3.41804 -2.77172
```

L

```
SCF (BP86) Energy = -1481.49376410
Enthalpy 0K = -1480.720414
Enthalpy 298K = -1480.719470
Free Energy 298K = -1480.848209
Lowest Frequency = 16.9294 cm-1
Second Frequency = 25.2193 cm-1
SCF (BP86-D3BJ) Energy =  -1481.72336207
SCF (C6H6) Energy =  -1481.52664381
SCF (6-311++G**) Energy =  -2293.56384773

Si  1.28981 -1.18202  1.86040
Si -1.39192  0.32855  2.10647
Al -0.00956  0.07597 -0.79603
O  -0.05415 -0.54738  2.69398
N   1.50160 -0.31255  0.34639
N  -1.54996  0.05382  0.37363
C   2.79823  0.08565 -0.11448
C   3.60329 -0.79483 -0.91188
C   4.85745 -0.36295 -1.38674
H   5.46102 -1.05043 -1.99301
C   5.34671  0.91740 -1.10789
H   6.32080  1.23945 -1.49440
C   4.56957  1.77913 -0.32596
H   4.94346  2.78531 -0.09859
C   3.31518  1.39235  0.18249
C   3.15352 -2.21856 -1.24368
H   2.11639 -2.31682 -0.88072
C   3.15080 -2.50417 -2.76108
H   4.16814 -2.44535 -3.19006
H   2.51365 -1.78516 -3.30104
H   2.76595 -3.52061 -2.96288
C   4.02792 -3.26144 -0.50909
H   4.02799 -3.09409  0.58150
H   5.07833 -3.20835 -0.85065
H   3.66460 -4.28843 -0.69921
C   2.55322  2.38348  1.06194
H   1.64513  1.85898  1.40353
C   2.11731  3.64931  0.28893
H   1.48722  3.41280 -0.58519
H   3.00015  4.21115 -0.07096
H   1.55023  4.32565  0.95637
C   3.37210  2.78476  2.31123
H   4.26896  3.36900  2.03326
H   3.71455  1.90385  2.87984
H   2.76395  3.41500  2.98542
C   0.93259 -3.04370  1.65375
H   0.15850 -3.21959  0.88910
H   0.57524 -3.47089  2.60730
H   1.83822 -3.59547  1.34932
C   2.79260 -1.00593  3.01160
H   2.71495 -1.72216  3.84806
H   2.84292  0.00954  3.43571
H   3.73641 -1.20070  2.47500
C  -2.92017 -0.27472  3.06412
H  -2.84502  0.03081  4.12228
H  -3.00070 -1.37295  3.03048
H  -3.84868  0.15034  2.64695
C  -1.08519  2.14275  2.60006
H  -0.29627  2.60034  1.98141
H  -0.76903  2.19968  3.65658
H  -1.99899  2.74954  2.48243
C  -2.84175 -0.11096 -0.22249
C  -3.41935 -1.41780 -0.36662
C  -4.66278 -1.56836 -1.00868
H  -5.08303 -2.57561 -1.12238
C  -5.37301 -0.46809 -1.50271
H  -6.33983 -0.60551 -2.00110
C  -4.82742  0.80998 -1.34550
H  -5.37891  1.68112 -1.72100
C  -3.58217  1.01374 -0.71812
C  -2.70992 -2.66798  0.15267
H  -1.86569 -2.31320  0.76772
C  -2.12556 -3.50850 -1.00517
H  -1.41600 -2.91408 -1.60211
```

```
H  -2.92799 -3.86163 -1.67928
H  -1.59612 -4.39873 -0.61686
C  -3.61876 -3.54358  1.04322
H  -4.44411 -3.99833  0.46488
H  -4.07077 -2.96001  1.86305
H  -3.03775 -4.37103  1.48944
C  -3.07981  2.44963 -0.55799
H  -2.06371  2.39088 -0.13293
C  -2.98112  3.20366 -1.90166
H  -3.97018  3.30032 -2.38710
H  -2.29830  2.69484 -2.59832
H  -2.58574  4.22213 -1.74046
C  -3.97500  3.23604  0.42880
H  -4.05609  2.72341  1.40278
H  -5.00045  3.34959  0.03046
H  -3.56992  4.24926  0.60548
C   0.41146  0.44395 -3.33487
H   1.49297  0.26646 -3.52085
H  -0.03088  0.77485 -4.29799
C  -0.23955 -0.78236 -2.63702
H   0.22939 -1.74373 -2.90655
H  -1.31055 -0.85449 -2.90466
C   0.36949  1.56741 -2.23577
O   0.43403  2.77153 -2.50226
```

TS(3-H)

```
SCF (BP86) Energy = -2432.77887346
Enthalpy 0K = -2431.624620
Enthalpy 298K = -2431.623676
Free Energy 298K = -2431.812753
Lowest Frequency = -19.2613 cm-1
Second Frequency = 6.9563 cm-1
SCF (BP86-D3BJ) Energy =  -2433.11061199
SCF (C6H6) Energy =  -2432.79029566
SCF (6-311++G**) Energy =  -3816.74997779
```

```
K   3.33742 -0.27045  0.37633
Si -4.04660 -1.05503 -1.29760
Si -3.97956  1.88759 -0.19909
Al -1.33316  0.07799 -0.14701
O  -4.59895  0.52633 -0.99945
O   4.43347  1.35977 -1.71488
O   4.20448  2.48194  0.89119
O   3.14464  0.87138  2.96900
O   3.33393 -1.96733  2.75161
O   3.58229 -3.09323  0.14157
O   4.73493 -1.47485 -1.89889
N  -2.59526 -1.32604 -0.32835
N  -2.21359  1.76809 -0.18757
C  -2.35854 -2.56735  0.34668
C  -1.71207 -3.66046 -0.31640
C  -1.48814 -4.86784  0.37583
H  -1.00646 -5.70110 -0.15113
C  -1.86801 -5.02939  1.71236
H  -1.68920 -5.97691  2.23333
C  -2.48962 -3.96069  2.36946
H  -2.79243 -4.07601  3.41729
C  -2.74866 -2.74124  1.71639
C  -1.25262 -3.56208 -1.77061
H  -1.41706 -2.51741 -2.08799
C   0.25380 -3.87377 -1.91772
H   0.47650 -4.92470 -1.65645
H   0.86174 -3.22841 -1.26098
H   0.58189 -3.72021 -2.96180
C  -2.07827 -4.48955 -2.69115
H  -3.15768 -4.27709 -2.61503
H  -1.93167 -5.55130 -2.42099
H  -1.77970 -4.36724 -3.74821
C  -3.42220 -1.61794  2.50034
H  -3.69029 -0.84422  1.76139
C  -2.43789 -0.97993  3.50768
H  -1.54368 -0.60074  2.98298
H  -2.11459 -1.71960  4.26380
H  -2.91328 -0.13775  4.04349
C  -4.71651 -2.06910  3.21027
H  -4.51222 -2.79571  4.01753
H  -5.42138 -2.54364  2.50676
H  -5.22411 -1.20302  3.67106
C  -3.74973 -1.11084 -3.17329
H  -2.86977 -0.50640 -3.45024
H  -4.62650 -0.70506 -3.70709
H  -3.57546 -2.13871 -3.53288
C  -5.42497 -2.26826 -0.82852
H  -6.30382 -2.11101 -1.47667
H  -5.73979 -2.12206  0.21741
H  -5.09609 -3.31501 -0.94161
C  -4.58022  3.42415 -1.13588
H  -5.65399  3.58609 -0.93970
H  -4.44742  3.30089 -2.22212
H  -4.03520  4.32965 -0.81928
C  -4.81656  1.91633  1.51137
H  -4.45442  1.11074  2.17008
H  -5.90447  1.78195  1.37853
H  -4.65636  2.87699  2.02857
C  -1.36437  2.92042 -0.20689
C  -0.87590  3.45433 -1.44758
C   0.03269  4.53153 -1.43389
H   0.39816  4.92748 -2.38950
C   0.45962  5.12237 -0.23821
H   1.15125  5.97280 -0.25196
C  -0.04349  4.63120  0.97285
H   0.25360  5.11058  1.91437
C  -0.94458  3.54828  1.01438
C  -1.32458  2.90650 -2.80343
H  -2.08442  2.13276 -2.59798
C  -0.16311  2.23187 -3.56572
H   0.25434  1.40420 -2.97127
H   0.64493  2.95500 -3.78446
H  -0.51873  1.82829 -4.53126
C  -1.96603  4.00319 -3.68472
H  -1.22503  4.76721 -3.98284
H  -2.78625  4.52156 -3.16274
H  -2.37297  3.56187 -4.61213
C  -1.48845  3.11022  2.37451
H  -2.12644  2.22966  2.19168
C  -0.37665  2.68622  3.35832
H   0.29615  3.53197  3.59622
H   0.22870  1.86421  2.94314
H  -0.81660  2.34007  4.31093
C  -2.35439  4.22499  3.00674
H  -3.15251  4.55728  2.32214
H  -1.74345  5.11258  3.25354
H  -2.82704  3.87406  3.94163
C   0.34164 -0.32411  0.82109
H   0.45097 -1.39069  1.08843
H   0.88965  0.31283  1.53574
C   0.57042 -0.03460 -0.73050
H   1.04867 -0.83023 -1.33137
H   1.04676  0.94235 -0.92964
C   4.92464  2.66138 -1.38719
H   4.89859  3.32367 -2.27928
H   5.97794  2.59907 -1.03699
C   4.05427  3.26406 -0.29945
H   4.38582  4.31019 -0.12082
H   2.99216  3.29651 -0.61924
C   3.46042  3.03361  1.98505
H   2.38002  3.08290  1.73893
H   3.80721  4.06660  2.20647
C   3.68432  2.17068  3.21410
H   4.76988  2.10342  3.44450
H   3.17740  2.64648  4.08071
C   3.23117  0.01228  4.10744
```

```
H   2.70691  0.46459  4.97641
H   4.29325 -0.14970  4.39305
C   2.57018 -1.31362  3.77329
H   2.53612 -1.93926  4.69163
H   1.53110 -1.14114  3.42690
C   2.76648 -3.23332  2.39398
H   1.73518 -3.10322  2.00611
H   2.72131 -3.90406  3.27915
C   3.64313 -3.87787  1.33574
H   4.69106 -3.94634  1.69982
H   3.27696 -4.90865  1.14220
C   4.38501 -3.63520 -0.90700
H   4.07320 -4.67585 -1.14089
H   5.45526 -3.65745 -0.60693
C   4.20919 -2.78294 -2.15099
H   4.75019 -3.26317 -2.99520
H   3.13429 -2.72500 -2.41953
C   4.61355 -0.62776 -3.04772
H   3.54864 -0.51409 -3.33535
H   5.15846 -1.06609 -3.91200
C   5.21622  0.72808 -2.72766
H   6.26774  0.60603 -2.38752
H   5.22653  1.34182 -3.65444
C  -0.00046 -1.11565 -4.47182
O   1.14723 -1.22032 -4.45683
```

H

```
SCF (BP86) Energy = -2432.78228709
Enthalpy 0K = -2431.627042
Enthalpy 298K = -2431.626098
Free Energy 298K = -2431.810745
Lowest Frequency = 6.2936 cm-1
Second Frequency = 15.2173 cm-1
SCF (BP86-D3BJ) Energy =  -2433.12279490
SCF (C6H6) Energy =  -2432.79314710
SCF (6-311++G**) Energy =  -3816.74986982
```

```
K   3.33655 -0.61134  0.18609
Si -4.24363 -0.75513 -1.03940
Si -3.66802  2.23326 -0.22004
Al -1.31600  0.20180 -0.43548
O  -4.30576  0.94011 -1.11780
O   4.41844  1.25786 -1.65784
O   4.50845  1.90120  1.10990
O   3.27067  0.09118  2.91918
O   3.09136 -2.67319  2.19888
O   3.12228 -3.32337 -0.57385
O   4.32229 -1.50174 -2.40759
N  -2.65762 -1.15612 -0.35367
N  -1.91622  1.99883 -0.19687
C  -2.40038 -2.41639  0.27766
C  -1.96046 -3.54959 -0.48485
C  -1.66886 -4.76591  0.16495
H  -1.33869 -5.62187 -0.43696
C  -1.79753 -4.90887  1.55073
H  -1.57296 -5.86453  2.03818
C  -2.23799 -3.81157  2.29962
H  -2.35311 -3.91355  3.38570
C  -2.54976 -2.57783  1.69635
C  -1.77852 -3.48652 -2.00043
H  -2.06575 -2.47065 -2.31601
C  -0.30387 -3.70838 -2.40624
H   0.01935 -4.74155 -2.17884
H   0.36579 -3.02092 -1.86329
H  -0.17006 -3.54514 -3.49077
C  -2.68539 -4.49919 -2.73529
H  -3.74415 -4.37485 -2.45370
H  -2.40193 -5.54053 -2.49660
H  -2.60473 -4.37461 -3.82983
C  -3.02121 -1.43706  2.59537
H  -3.32437 -0.61521  1.92447
C  -1.86776 -0.91451  3.47931
H  -1.03457 -0.56329  2.84818
H  -1.49586 -1.70884  4.15344
H  -2.20698 -0.07291  4.11008
C  -4.23435 -1.82851  3.46785
H  -3.96959 -2.60188  4.21149
H  -5.06489 -2.22153  2.85916
H  -4.60455 -0.95115  4.02816
C  -4.59126 -1.30368 -2.82443
H  -3.79846 -0.96740 -3.51085
H  -5.54354 -0.85267 -3.15505
H  -4.68892 -2.39833 -2.91254
C  -5.65609 -1.42188  0.04231
H  -6.62270 -1.28208 -0.47149
H  -5.70446 -0.89978  1.01101
H  -5.52807 -2.50016  0.23959
C  -4.20544  3.83007 -1.08754
H  -5.27980  4.00935 -0.91032
H  -4.04446  3.75994 -2.17456
H  -3.64792  4.70250 -0.70607
C  -4.54417  2.20064  1.47217
H  -4.24221  1.33579  2.08532
H  -5.63366  2.13427  1.30393
H  -4.35177  3.11435  2.05791
C  -0.95789  3.04680 -0.01914
C  -0.36572  3.70806 -1.15204
C   0.62480  4.69127 -0.95365
H   1.05699  5.18814 -1.83097
C   1.05250  5.06479  0.32513
H   1.81064  5.84557  0.45655
C   0.46409  4.44506  1.43344
H   0.76422  4.75275  2.44304
C  -0.52643  3.45248  1.29141
C  -0.78592  3.41729 -2.59429
H  -1.55221  2.62297 -2.55287
C   0.39157  2.91199 -3.45883
H   0.85854  2.01506 -3.02309
H   1.17059  3.68973 -3.56279
H   0.04206  2.65298 -4.47361
C  -1.40985  4.66331 -3.26685
H  -0.66253  5.46949 -3.38318
H  -2.24911  5.06957 -2.68153
H  -1.78409  4.41122 -4.27528
C  -1.14842  2.88569  2.56725
H  -1.82324  2.06945  2.25959
C  -0.09928  2.30011  3.53749
H   0.59274  3.08219  3.90215
H   0.49743  1.50686  3.05792
H  -0.59335  1.86447  4.42400
C  -1.97651  3.96991  3.29796
H  -2.71263  4.44177  2.62673
H  -1.32224  4.77387  3.68204
H  -2.52013  3.54016  4.15838
C   0.36297 -0.32484  0.53510
H   0.38353 -1.38853  0.82771
H   0.90070  0.30608  1.26259
C   0.64575 -0.07487 -0.96863
H   1.00642 -0.90039 -1.60754
H   1.14564  0.87738 -1.21266
C   5.12636  2.39332 -1.15705
H   5.14559  3.20638 -1.91429
H   6.17802  2.12065 -0.92058
C   4.42487  2.90361  0.08874
H   4.92762  3.83787  0.42148
H   3.36633  3.14913 -0.13582
C   3.87952  2.33263  2.32321
H   2.81130  2.57246  2.14420
H   4.37557  3.24883  2.71169
C   4.00526  1.23287  3.36292
H   5.07462  0.97123  3.51862
H   3.60002  1.60702  4.32744
```

C   3.25851 -0.96636 3.88103
H   2.81008 -0.61713 4.83569
H   4.29397 -1.31070 4.09338
C   2.42655 -2.11611 3.33972
H   2.31518 -2.88319 4.13640
H   1.41636 -1.75413 3.05992
C   2.37173 -3.78847 1.65565
H   1.34720 -3.48799 1.35540
H   2.28466 -4.59872 2.41122
C   3.13129 -4.31849 0.45261
H   4.17546 -4.57070 0.73909
H   2.63684 -5.24747 0.09742
C   3.77895 -3.75420 -1.76598
H   3.32497 -4.69506 -2.14425
H   4.85655 -3.94743 -1.57149
C   3.62050 -2.67843 -2.82608
H   4.03174 -3.05404 -3.78821
H   2.54357 -2.45548 -2.97359
C   4.15712 -0.42916 -3.34313
H   3.08618 -0.14802 -3.42203
H   4.51206 -0.73411 -4.35133
C   4.97421 0.76239 -2.87640
H   6.03405 0.46120 -2.72798
H   4.94801 1.54620 -3.66384
C -1.35196 0.10381 -2.67070
O -0.85132 -0.20114 -3.68995

TS(H-I)
SCF (BP86) Energy = -2432.78139557
Enthalpy 0K = -2431.626921
Enthalpy 298K = -2431.625977
Free Energy 298K = -2431.809264
Lowest Frequency = -184.7321 cm-1
Second Frequency = 9.5796 cm-1
SCF (BP86-D3BJ) Energy = -2433.12138621
SCF (C6H6) Energy = -2432.79257746
SCF (6-311++G**) Energy = -3816.74859197

K   3.34858 -0.64415 0.22265
Si -4.23918 -0.70939 -1.14373
Si -3.64060 2.27600 -0.29071
Al -1.34245 0.20815 -0.48145
O -4.26220 0.98872 -1.20649
O   4.53504 1.19710 -1.57866
O   4.50672 1.85426 1.18606
O   3.15392 0.07298 2.94294
O   2.97540 -2.69167 2.23066
O   3.12173 -3.35903 -0.53121
O   4.42148 -1.56340 -2.32255
N -2.69731 -1.13872 -0.38233
N -1.89068 2.02218 -0.22539
C -2.47621 -2.40479 0.24647
C -2.01307 -3.53659 -0.50470
C -1.76155 -4.75950 0.14915
H -1.41355 -5.61484 -0.44361
C -1.95161 -4.91039 1.52740
H -1.75764 -5.87159 2.01704
C -2.41082 -3.81326 2.26516
H -2.57089 -3.92042 3.34507
C -2.68308 -2.57286 1.65667
C -1.76357 -3.46313 -2.01019
H -2.02409 -2.44115 -2.33030
C -0.27525 -3.70039 -2.35385
H   0.02534 -4.73924 -2.12120
H   0.38108 -3.02522 -1.78012
H -0.09439 -3.53146 -3.43070
C -2.65017 -4.45816 -2.79241
H -3.71868 -4.32106 -2.55726
H -2.39163 -5.50489 -2.54891
H -2.51910 -4.32696 -3.88125
C -3.16871 -1.42513 2.53816
H -3.44679 -0.60463 1.85488
C -2.03025 -0.90876 3.44476
H -1.17562 -0.57209 2.83420
H -1.68167 -1.70257 4.13167
H -2.37248 -0.05820 4.06179
C -4.40705 -1.79858 3.38160
H -4.17243 -2.57425 4.13293
H -5.22665 -2.18136 2.75160
H -4.78009 -0.91536 3.93064
C -4.48428 -1.22475 -2.95409
H -3.63803 -0.89258 -3.57624
H -5.40237 -0.74935 -3.34198
H -4.59540 -2.31568 -3.06791
C -5.71580 -1.35877 -0.14058
H -6.65569 -1.18720 -0.69279
H -5.79375 -0.85291 0.83505
H -5.62105 -2.44300 0.04258
C -4.15529 3.87463 -1.16745
H -5.23661 4.04375 -1.02560
H -3.95692 3.81307 -2.24867
H -3.61901 4.74898 -0.76091
C -4.54899 2.25058 1.38404
H -4.26791 1.38251 2.00243
H -5.63585 2.19671 1.19560
H -4.35734 3.16233 1.97330
C -0.92479 3.05569 -0.00810
C -0.30891 3.74027 -1.11487
C   0.68576 4.71003 -0.87539
H   1.13627 5.22453 -1.73304
C   1.09586 5.04851 0.41879
H   1.85777 5.81960 0.58157
C   0.48549 4.40544 1.50150
H   0.77147 4.68498 2.52338
C -0.51007 3.42459 1.31884
C -0.70592 3.49034 -2.57169
H -1.47222 2.69542 -2.56713
C   0.48485 3.01243 -3.43415
H   0.95095 2.10552 -3.01978
H   1.26118 3.79627 -3.50834
H   0.14761 2.77879 -4.45897
C -1.31630 4.75729 -3.21796
H -0.56333 5.56222 -3.30158
H -2.16172 5.15227 -2.63421
H -1.67686 4.53447 -4.23816
C -1.15312 2.82948 2.57128
H -1.83474 2.03085 2.23368
C -0.12011 2.20448 3.53465
H   0.57420 2.96977 3.92958
H   0.47765 1.42154 3.03906
H -0.62763 1.74737 4.40251
C -1.97677 3.90069 3.32562
H -2.70036 4.40066 2.66130
H -1.31793 4.68521 3.74076
H -2.53451 3.45148 4.16684
C   0.34986 -0.33374 0.40623
H   0.36036 -1.40144 0.68656
H   0.85988 0.27882 1.16973
C   0.72610 -0.07408 -1.07066
H   1.14436 -0.89015 -1.68609
H   1.21648 0.89097 -1.28034
C   5.23870 2.32325 -1.05203
H   5.30649 3.13156 -1.81160
H   6.27370 2.03473 -0.76597
C   4.48915 2.85243 0.15756
H   4.99202 3.77959 0.50935
H   3.44656 3.11468 -0.11707
C   3.82599 2.30250 2.36514
H   2.77215 2.55968 2.13291
H   4.31774 3.21248 2.77325
C   3.88283 1.20735 3.41547
H   4.93879 0.93019 3.62583

```
H   3.43551  1.59358  4.35622
C   3.08232 -0.97750  3.91029
H   2.59176 -0.61609  4.83925
H   4.10223 -1.33146  4.17524
C   2.26502 -2.12229  3.33738
H   2.11054 -2.88369  4.13223
H   1.27134 -1.75350  3.01099
C   2.27418 -3.80793  1.66608
H   1.26413 -3.50569  1.32176
H   2.15216 -4.61226  2.42306
C   3.08017 -4.34940  0.49890
H   4.10937 -4.60810  0.83025
H   2.59412 -5.27654  0.12762
C   3.81585 -3.80388 -1.69693
H   3.36121 -4.73857 -2.08941
H   4.88206 -4.01364 -1.46142
C   3.71507 -2.72870 -2.76459
H   4.15521 -3.11405 -3.70991
H   2.64826 -2.48754 -2.95218
C   4.31541 -0.49183 -3.26812
H   3.25400 -0.19210 -3.39335
H   4.70779 -0.80746 -4.25901
C   5.13332  0.68689 -2.77085
H   6.18039  0.36775 -2.57641
H   5.15453  1.46790 -3.56122
C  -1.10389  0.11202 -2.55426
O  -0.68862 -0.09431 -3.64501
```

I

```
SCF (BP86) Energy = -2432.82834304
Enthalpy 0K = -2431.670164
Enthalpy 298K = -2431.669219
Free Energy 298K = -2431.850441
Lowest Frequency = 8.7178 cm-1
Second Frequency = 16.9709 cm-1
SCF (BP86-D3BJ) Energy =  -2433.16407451
SCF (C6H6) Energy =  -2432.84133400
SCF (6-311++G**) Energy =  -3816.79209069
```

```
K   3.74736  0.01230 -0.11621
Si -4.12070 -1.61015  0.03718
Si -3.96572  1.09176  1.55671
Al -1.44009  0.08760 -0.13500
O  -4.68834 -0.35911  1.03725
O   4.54479  2.68309 -0.80034
O   5.13373  1.68535  1.80452
O   4.18516 -0.89810  2.52199
O   3.73269 -2.76727  0.41078
O   3.08964 -1.75576 -2.17876
O   4.04464  0.80965 -2.87806
N  -2.34513 -1.58029  0.05116
N  -2.61298  1.46439  0.48196
C  -1.61272 -2.80716  0.12624
C  -1.23850 -3.52560 -1.05854
C  -0.53392 -4.74220 -0.94559
H  -0.27862 -5.29209 -1.86019
C  -0.17190 -5.27148  0.29866
H   0.36187 -6.22662  0.36547
C  -0.52096 -4.56729  1.45820
H  -0.25249 -4.97897  2.43883
C  -1.23932 -3.35723  1.40023
C  -1.61397 -3.03311 -2.45649
H  -2.09058 -2.04599 -2.33495
C  -0.37901 -2.84632 -3.36576
H   0.11219 -3.81548 -3.57718
H   0.35839 -2.16895 -2.90616
H  -0.67774 -2.40576 -4.33244
C  -2.62891 -3.98524 -3.13082
H  -3.52583 -4.13358 -2.50633
H  -2.18378 -4.98134 -3.30879
H  -2.95274 -3.58397 -4.10764
C  -1.61241 -2.66057  2.70842
H  -2.34468 -1.87862  2.44642
C  -0.38871 -1.95746  3.33929
H   0.05175 -1.22799  2.63968
H   0.38890 -2.69642  3.61148
H  -0.67380 -1.42079  4.26274
C  -2.26803 -3.60972  3.73467
H  -1.55451 -4.36332  4.11514
H  -3.12278 -4.15295  3.29885
H  -2.63360 -3.03759  4.60554
C  -4.87881 -1.31740 -1.68066
H  -4.41467 -0.45644 -2.18836
H  -5.95963 -1.11503 -1.58425
H  -4.75407 -2.19898 -2.33135
C  -4.79708 -3.24299  0.72292
H  -5.89415 -3.26449  0.60599
H  -4.56771 -3.35359  1.79451
H  -4.37811 -4.11259  0.18946
C  -5.29646  2.44179  1.52701
H  -5.99690  2.30024  2.36778
H  -5.87519  2.39706  0.59100
H  -4.85574  3.44936  1.61123
C  -3.46797  0.78176  3.36925
H  -2.60097  0.10456  3.44118
H  -4.30960  0.31533  3.91034
H  -3.20888  1.71995  3.88779
C  -2.32398  2.80261  0.04867
C  -2.83332  3.29625 -1.19977
C  -2.45618  4.57739 -1.64709
H  -2.84364  4.93549 -2.60853
C  -1.61411  5.40425 -0.89604
H  -1.33659  6.39878 -1.26295
C  -1.14917  4.94615  0.34136
H  -0.51244  5.59874  0.95164
C  -1.48482  3.66731  0.82889
C  -3.81866  2.49619 -2.05274
H  -4.01928  1.56021 -1.50482
C  -3.25307  2.12955 -3.44379
H  -2.33007  1.53279 -3.37711
H  -3.02744  3.03948 -4.03022
H  -3.99995  1.54983 -4.01686
C  -5.15910  3.25127 -2.21773
H  -5.03065  4.16908 -2.81987
H  -5.58539  3.55017 -1.24613
H  -5.89791  2.61595 -2.73817
C  -0.97894  3.26419  2.21516
H  -1.22079  2.19555  2.34493
C   0.54934  3.42779  2.37610
H   0.85636  4.48709  2.30287
H   1.09552  2.86262  1.60148
H   0.87193  3.06038  3.36792
C  -1.70714  4.06807  3.31800
H  -2.80153  3.95623  3.24323
H  -1.48071  5.14649  3.23305
H  -1.39579  3.73597  4.32540
C   0.56412  0.21512  0.38642
H   1.02467 -0.78512  0.25827
H   0.86446  0.63439  1.36123
C   0.81676  1.13397 -0.83846
H   1.80450  1.13948 -1.34738
H   0.58714  2.18613 -0.56975
C   5.39400  3.37258  0.11546
H   5.34685  4.46999 -0.05479
H   6.45143  3.05342 -0.01299
C   4.93373  3.07770  1.53238
H   5.52319  3.69996  2.23964
H   3.86278  3.34667  1.64758
C   4.76539  1.34206  3.14477
H   3.68994  1.55559  3.31994
H   5.35657  1.93509  3.87543
C   5.04492 -0.13424  3.36709
```

H 6.11124 -0.35548 3.14348
H 4.86285 -0.37773 4.43606
C 4.37329 -2.30873 2.67940
H 4.18526 -2.60969 3.73206
H 5.41823 -2.58824 2.42332
C 3.39927 -3.04265 1.77513
H 3.47372 -4.13209 1.98189
H 2.35817 -2.72836 1.99440
C 2.84139 -3.44579 -0.49252
H 1.79377 -3.12528 -0.32667
H 2.88440 -4.54173 -0.32256
C 3.26169 -3.15130 -1.91953
H 4.32196 -3.44661 -2.08028
H 2.62635 -3.75269 -2.60259
C 3.41345 -1.41198 -3.53084
H 2.77797 -1.98610 -4.23708
H 4.47748 -1.65868 -3.74118
C 3.15921 0.06875 -3.74353
H 3.37704 0.31342 -4.80557
H 2.09707 0.32213 -3.53285
C 3.91747 2.21981 -3.07591
H 2.88189 2.55265 -2.85792
H 4.15063 2.48952 -4.12906
C 4.90176 2.93212 -2.16473
H 5.93490 2.57249 -2.36250
H 4.86982 4.02151 -2.38039
C -0.31870 0.67771 -1.81112
O -0.13274 0.56042 -3.02953

4
SCF (BP86) Energy = -2432.83576798
Enthalpy 0K = -2431.677977
Enthalpy 298K = -2431.677033
Free Energy 298K = -2431.861226
Lowest Frequency = 7.4919 cm-1
Second Frequency = 14.1154 cm-1
SCF (BP86-D3BJ) Energy = -2433.16393903
SCF (C6H6) Energy = -2432.84806218
SCF (6-311++G**) Energy = -3816.80016202

K -3.90171 0.04109 0.10242
Si 3.96664 1.23677 -1.59839
Si 4.35263 -1.52809 -0.24073
Al 1.67817 0.10272 0.33279
O 4.77843 -0.21446 -1.23378
O -3.49689 -2.52128 1.14878
O -4.44195 -0.54870 2.93876
O -4.64821 2.13135 1.96388
O -5.34200 2.45220 -0.78758
O -4.54679 0.44395 -2.60796
O -4.03764 -2.21973 -1.64392
N 2.73943 1.52920 -0.35668
N 2.59475 -1.53564 -0.03907
C 2.50263 2.85153 0.15057
C 1.52443 3.70971 -0.45181
C 1.27132 4.98046 0.10216
H 0.52376 5.62813 -0.37286
C 1.95251 5.43635 1.23512
H 1.73846 6.42554 1.65519
C 2.92287 4.61144 1.81476
H 3.47104 4.96498 2.69635
C 3.22215 3.33857 1.29270
C 0.76228 3.30645 -1.71480
H 1.02313 2.25615 -1.92885
C -0.77075 3.38210 -1.53408
H -1.10297 4.41789 -1.33500
H -1.10436 2.74287 -0.70123
H -1.27981 3.03514 -2.45198
C 1.19465 4.17151 -2.92163
H 2.28498 4.12624 -3.08091
H 0.92991 5.23315 -2.76461
H 0.69791 3.83397 -3.84952
C 4.31434 2.51308 1.97341
H 4.50827 1.64573 1.31986
C 3.84889 1.97406 3.34535
H 2.94996 1.34615 3.24154
H 3.60645 2.80466 4.03331
H 4.64363 1.36856 3.81843
C 5.63594 3.29751 2.13323
H 5.53294 4.13029 2.85223
H 5.97522 3.72663 1.17583
H 6.43216 2.63364 2.51440
C 3.25451 1.01491 -3.34885
H 2.39954 0.31947 -3.35204
H 4.02910 0.60935 -4.02269
H 2.90946 1.97592 -3.76619
C 5.26048 2.62108 -1.65642
H 5.91770 2.48037 -2.53162
H 5.89027 2.61387 -0.75303
H 4.78704 3.61391 -1.73771
C 4.98030 -3.10747 -1.08241
H 6.07926 -3.16305 -1.00025
H 4.71964 -3.11354 -2.15245
H 4.55499 -4.01321 -0.61833
C 5.30377 -1.29450 1.38950
H 4.89143 -0.46192 1.98254
H 6.36422 -1.07118 1.17970
H 5.26398 -2.20450 2.01179
C 1.82343 -2.73888 -0.09646
C 1.28395 -3.20539 -1.34379
C 0.46874 -4.35358 -1.36645
H 0.06973 -4.70038 -2.32798
C 0.17884 -5.07547 -0.20109
H -0.43859 -5.98057 -0.24397
C 0.72923 -4.64532 1.01218
H 0.53715 -5.22308 1.92482
C 1.54659 -3.49988 1.08913
C 1.59300 -2.50393 -2.66645
H 2.33969 -1.72477 -2.43922
C 0.34898 -1.80566 -3.26115
H -0.09192 -1.07885 -2.55855
H -0.42713 -2.55219 -3.51767
H 0.61256 -1.27492 -4.19460
C 2.20340 -3.46817 -3.70880
H 1.47056 -4.22667 -4.04003
H 3.07731 -4.00623 -3.30647
H 2.52582 -2.91022 -4.60584
C 2.17541 -3.14213 2.43681
H 2.65441 -2.15608 2.31311
C 1.14412 -3.02873 3.57979
H 0.64445 -3.99440 3.77924
H 0.36504 -2.28385 3.34608
H 1.64021 -2.71789 4.51648
C 3.26932 -4.16900 2.81495
H 4.03412 -4.25574 2.02511
H 2.83396 -5.17403 2.96374
H 3.77601 -3.87898 3.75317
C 0.72092 0.44577 2.10507
H 0.66550 1.53138 2.30523
H 1.02529 -0.05076 3.04040
C -0.62659 -0.07339 1.54284
H -1.54863 0.46507 1.86578
H -0.77595 -1.14775 1.78889
C -3.97441 -2.85839 2.44868
H -3.44894 -3.75444 2.84290
H -5.06270 -3.08648 2.41990
C -3.70098 -1.69283 3.38352
H -4.00504 -1.97412 4.41501
H -2.61506 -1.46630 3.38976
C -4.18249 0.59522 3.75753
H -3.10845 0.87352 3.70738
H -4.43150 0.38074 4.81960

C	-5.04584	1.75023	3.28358
H	-6.11629	1.45097	3.29174
H	-4.92305	2.60091	3.98813
C	-5.41559	3.23431	1.47779
H	-5.25507	4.13201	2.11319
H	-6.49975	2.99027	1.49795
C	-4.99162	3.55379	0.05542
H	-5.51482	4.47861	-0.27085
H	-3.89884	3.74721	0.01257
C	-5.07722	2.72739	-2.16787
H	-4.00642	2.98050	-2.31462
H	-5.68798	3.58786	-2.51725
C	-5.42742	1.49705	-2.98778
H	-6.48784	1.20912	-2.81681
H	-5.31024	1.74178	-4.06579
C	-4.68397	-0.72975	-3.41349
H	-4.49447	-0.48954	-4.48165
H	-5.71235	-1.14349	-3.33025
C	-3.66504	-1.75473	-2.94715
H	-3.64676	-2.59953	-3.66973
H	-2.65654	-1.29577	-2.91885
C	-3.07296	-3.15078	-1.12653
H	-2.07465	-2.67727	-1.05033
H	-2.98481	-4.03074	-1.79877
C	-3.53283	-3.62653	0.23860
H	-4.56303	-4.03910	0.17433
H	-2.84909	-4.43035	0.58202
C	-0.41638	-0.05933	-0.00498
O	-1.37536	-0.01274	-0.80166

TS(L-M)

SCF (BP86) Energy = -2849.52442399
Enthalpy 0K = -2847.992532
Enthalpy 298K = -2847.991588
Free Energy 298K = -2848.225907
Lowest Frequency = -994.6452 cm-1
Second Frequency = 6.6663 cm-1
SCF (BP86-D3BJ) Energy = -2849.99008426
SCF (C6H6) Energy = -2849.62833379
SCF (6-311++G**) Energy = -4473.62803442

Si	-6.54780	-1.26106	0.32193
Si	-5.95780	0.90909	-1.82029
Si	6.53736	0.16461	1.12834
Si	5.94353	-1.59406	-1.31085
Al	-3.73215	0.25383	0.34557
Al	3.45636	0.01470	0.17335
O	-6.90515	-0.24647	-0.99350
O	6.97303	-0.85358	-0.17349
N	-4.80081	-1.30551	0.56985
N	-4.67730	1.47534	-0.75241
N	5.04867	1.01057	0.77398
N	4.28747	-1.54540	-0.73796
C	-4.15592	-2.51482	1.00932
C	-3.62679	-3.44406	0.05352
C	-2.94093	-4.58745	0.50675
H	-2.51567	-5.27741	-0.23149
C	-2.77906	-4.85671	1.87021
H	-2.23022	-5.74614	2.19985
C	-3.32140	-3.96903	2.80382
H	-3.20273	-4.17361	3.87522
C	-4.00624	-2.80369	2.40376
C	-3.79284	-3.24702	-1.45454
H	-4.48096	-2.39425	-1.58766
C	-2.45793	-2.88878	-2.14595
H	-1.69752	-3.67395	-1.98779
H	-2.04767	-1.94638	-1.75077
H	-2.60428	-2.77167	-3.23589
C	-4.42518	-4.48393	-2.13421
H	-5.36596	-4.78751	-1.64400
H	-3.74196	-5.35235	-2.11074
H	-4.64226	-4.26831	-3.19630
C	-4.57894	-1.89275	3.49052
H	-5.04341	-1.03377	2.97727
C	-3.48685	-1.34244	4.43353
H	-2.72211	-0.78551	3.87031
H	-2.97763	-2.15539	4.98245
H	-3.92833	-0.65862	5.18164
C	-5.67318	-2.61815	4.30846
H	-5.25008	-3.47129	4.87023
H	-6.47091	-3.01688	3.65803
H	-6.13660	-1.93275	5.04213
C	-7.24975	-2.98399	-0.06854
H	-6.94674	-3.31102	-1.07569
H	-8.35290	-2.95826	-0.03208
H	-6.89584	-3.73478	0.65773
C	-7.55446	-0.55722	1.78164
H	-8.59647	-0.37929	1.46137
H	-7.13981	0.40138	2.13449
H	-7.57226	-1.25404	2.63651
C	-5.34271	0.02809	-3.38925
H	-6.18027	-0.48348	-3.89531
H	-4.58113	-0.72848	-3.13704
H	-4.88755	0.73728	-4.10006
C	-7.13012	2.32360	-2.30466
H	-7.66727	2.70237	-1.41973
H	-7.87684	1.96616	-3.03484
H	-6.57853	3.16613	-2.75359
C	-4.36374	2.87534	-0.64218
C	-3.45489	3.50433	-1.55363
C	-3.16622	4.87572	-1.40446
H	-2.46112	5.34284	-2.10164
C	-3.73481	5.64287	-0.38315
H	-3.48406	6.70508	-0.27954
C	-4.61620	5.02867	0.51408
H	-5.06010	5.61892	1.32578
C	-4.94479	3.66461	0.40358
C	-2.79936	2.74685	-2.70949
H	-3.01920	1.67558	-2.55629
C	-3.42595	3.17683	-4.05946
H	-3.21625	4.24345	-4.26302
H	-4.52276	3.04835	-4.07211
H	-2.99980	2.58875	-4.89299
C	-1.26584	2.92269	-2.77009
H	-0.73269	2.63528	-1.84726
H	-0.98974	3.97011	-2.99339
H	-0.85120	2.30366	-3.58534
C	-5.90297	3.05793	1.42739
H	-6.14576	2.04417	1.06501
C	-7.22744	3.84300	1.54653
H	-7.72060	3.95601	0.56563
H	-7.06700	4.85696	1.95692
H	-7.92727	3.32051	2.22428
C	-5.22593	2.91609	2.80994
H	-4.93890	3.90510	3.21239
H	-4.31283	2.30144	2.73396
H	-5.90838	2.43861	3.53820
C	-1.37804	0.05999	0.15716
H	-1.48963	0.47781	-0.85881
H	-1.41443	-1.04160	0.18216
C	-2.14491	0.79340	1.30236
H	-1.88865	1.86611	1.31490
H	-1.87430	0.36214	2.27816
C	4.98660	2.42321	0.96676
C	4.71948	2.98623	2.25983
C	4.71995	4.38369	2.43448
H	4.52070	4.79329	3.43388
C	4.94876	5.25768	1.36566
H	4.93969	6.34397	1.51900
C	5.16459	4.71741	0.09250
H	5.31914	5.39046	-0.76107
C	5.18579	3.32815	-0.13058

```
C   4.39331  2.10372  3.46526
H   4.40526  1.06094  3.10488
C   2.97429  2.39237  4.00351
H   2.22529  2.25877  3.20641
H   2.88951  3.42682  4.38680
H   2.72295  1.70485  4.83260
C   5.43981  2.24428  4.59338
H   5.46634  3.27692  4.99010
H   6.45648  2.00088  4.23840
H   5.20234  1.57024  5.43804
C   5.42316  2.82110 -1.55117
H   5.42281  1.72046 -1.48275
C   4.28888  3.24056 -2.51333
H   4.25179  4.34235 -2.62183
H   3.30027  2.89067 -2.15443
H   4.47227  2.81949 -3.52117
C   6.79994  3.26157 -2.09935
H   7.62652  2.93807 -1.44272
H   6.86060  4.36229 -2.19633
H   6.97458  2.82983 -3.10246
C   6.43648 -0.97418  2.65720
H   7.29984 -1.66208  2.69662
H   5.51415 -1.57720  2.62450
H   6.41877 -0.38686  3.59172
C   8.01061  1.34878  1.37468
H   8.20167  1.93333  0.45996
H   8.92273  0.77419  1.61478
H   7.82018  2.06205  2.19410
C   6.58702 -3.37747 -1.54512
H   6.74658 -3.87037 -0.57275
H   7.55129 -3.36313 -2.08411
H   5.87492 -3.98700 -2.12713
C   6.28737 -0.70832 -2.96864
H   7.37757 -0.63125 -3.13159
H   5.86612  0.30940 -2.98450
H   5.85307 -1.26767 -3.81522
C   3.40333 -2.62267 -1.05011
C   2.58288 -2.59453 -2.22898
C   1.66926 -3.63937 -2.47249
H   1.04438 -3.59517 -3.37381
C   1.53755 -4.72316 -1.59805
H   0.81505 -5.52252 -1.80284
C   2.34762 -4.76844 -0.45662
H   2.25350 -5.61446  0.23647
C   3.27739 -3.75222 -0.16890
C   2.69663 -1.47795 -3.26894
H   3.46383 -0.77666 -2.90091
C   3.16678 -2.04257 -4.63109
H   2.40667 -2.71551 -5.07101
H   4.10163 -2.62245 -4.53320
H   3.34310 -1.22389 -5.35319
C   1.38621 -0.67970 -3.44181
H   1.13594 -0.12562 -2.52409
H   0.54064 -1.34253 -3.70420
H   1.49658  0.06235 -4.25408
C   4.13014 -3.87925  1.09434
H   4.90409 -3.09571  1.02535
C   4.83508 -5.25001  1.20109
H   5.41469 -5.48429  0.29200
H   4.11019 -6.07118  1.35549
H   5.52717 -5.26085  2.06376
C   3.30386 -3.60376  2.37019
H   2.47922 -4.33411  2.47104
H   2.86485 -2.59454  2.33561
H   3.93867 -3.68304  3.27350
C   1.20600  0.84785  0.83813
H   0.72181  1.64406  1.44406
H  -0.03614  0.34619  0.37102
C   1.98337 -0.28083  1.54834
H   2.21889 -0.06349  2.60974
H   1.42279 -1.23712  1.53757
C   1.92168  1.29338 -0.37282
O   1.52836  2.18507 -1.17589
```

M

SCF (BP86) Energy = -2849.55659388
Enthalpy 0K = -2848.019225
Enthalpy 298K = -2848.018281
Free Energy 298K = -2848.251616
Lowest Frequency = 8.9385 cm-1
Second Frequency = 11.4965 cm-1
SCF (BP86-D3BJ) Energy = -2850.02761172
SCF (C6H6) Energy = -2849.66606610
SCF (BS2) Energy = -4473.65794440

```
Si -6.42614  1.31769  0.88992
Si -5.77663 -1.70856  1.24891
Si  6.10411 -1.54709  0.76286
Si  5.68255  1.40674  1.42409
Al -3.81756  0.15013 -0.48243
Al  3.51563 -0.05930 -0.56188
O  -6.63578 -0.25878  1.48752
O   6.50597 -0.08416  1.54871
N  -4.74622  1.54036  0.37551
N  -4.66498 -1.48098 -0.10926
N   4.40389 -1.58092  0.34653
N   4.72636  1.43887 -0.03911
C  -4.03563  2.75268  0.69459
C  -3.05861  2.76051  1.74209
C  -2.40191  3.96583  2.05859
H  -1.65649  3.96367  2.86149
C  -2.67365  5.15653  1.37511
H  -2.14794  6.08072  1.63987
C  -3.60092  5.14016  0.32882
H  -3.79485  6.06106 -0.23560
C  -4.27700  3.96027 -0.03822
C  -2.71776  1.51733  2.56734
H  -3.22069  0.65346  2.09141
C  -1.20524  1.21174  2.62720
H  -0.65015  2.03864  3.10712
H  -0.74541  1.00977  1.64075
H  -1.03617  0.31308  3.24649
C  -3.30131  1.64003  3.99644
H  -4.39232  1.80574  3.98487
H  -2.83795  2.48822  4.53297
H  -3.09730  0.72376  4.57923
C  -5.22281  4.01133 -1.23964
H  -5.61778  2.99141 -1.38151
C  -4.47647  4.40020 -2.53636
H  -3.64048  3.71054 -2.73291
H  -4.06074  5.42209 -2.47112
H  -5.16234  4.37238 -3.40357
C  -6.41504  4.96623 -1.00306
H  -6.06889  6.00691 -0.86462
H  -6.99073  4.68849 -0.10336
H  -7.10491  4.95851 -1.86761
C  -6.89450  2.52353  2.27859
H  -6.39357  2.24651  3.22007
H  -7.98495  2.50180  2.44882
H  -6.59857  3.55571  2.02895
C  -7.70596  1.47041 -0.51507
H  -8.67680  1.07275 -0.17055
H  -7.39452  0.88678 -1.39773
H  -7.85538  2.51532 -0.83353
C  -4.96252 -2.06833  2.92699
H  -5.72633 -2.01888  3.72329
H  -4.18512 -1.32065  3.15571
H  -4.49446 -3.06554  2.95631
C  -7.03480 -3.08133  0.87340
H  -7.67617 -2.80751  0.01988
H  -7.68159 -3.25477  1.75088
H  -6.52304 -4.02684  0.62762
```

C	-4.34396	-2.58498	-0.98413
C	-3.28892	-3.49608	-0.66950
C	-2.97740	-4.52244	-1.58503
H	-2.15258	-5.20375	-1.34714
C	-3.67504	-4.67683	-2.78680
H	-3.40506	-5.47633	-3.48659
C	-4.71376	-3.78925	-3.09008
H	-5.25967	-3.89893	-4.03525
C	-5.06521	-2.74656	-2.21166
C	-2.48617	-3.41968	0.62973
H	-2.87339	-2.56099	1.20339
C	-2.71745	-4.69612	1.47650
H	-2.29106	-5.58573	0.97819
H	-3.79151	-4.89636	1.64735
H	-2.22005	-4.60043	2.45809
C	-0.97496	-3.19176	0.39922
H	-0.73297	-2.18359	0.01567
H	-0.55285	-3.94169	-0.29255
H	-0.42924	-3.29685	1.35256
C	-6.18824	-1.78960	-2.61365
H	-6.44843	-1.21025	-1.71055
C	-7.46344	-2.51864	-3.08915
H	-7.81448	-3.24795	-2.33895
H	-7.29651	-3.06695	-4.03416
H	-8.27717	-1.79316	-3.27182
C	-5.70537	-0.78608	-3.68634
H	-5.39559	-1.31263	-4.60740
H	-4.83265	-0.21083	-3.32683
H	-6.50513	-0.06961	-3.95262
C	-1.81804	1.50585	-2.30978
H	-1.85206	2.41633	-1.68622
H	-2.50458	1.65955	-3.16435
C	-2.15126	0.23923	-1.48992
H	-1.27843	0.05381	-0.77820
H	-2.14551	-0.65220	-2.14852
C	3.68787	-2.78496	0.62470
C	3.58477	-3.83125	-0.35127
C	2.93921	-5.04023	-0.02467
H	2.87936	-5.83064	-0.78485
C	2.36605	-5.25144	1.23409
H	1.86928	-6.20032	1.47174
C	2.41512	-4.21521	2.17509
H	1.94063	-4.35424	3.15520
C	3.05257	-2.99189	1.89708
C	4.12453	-3.65700	-1.77021
H	4.60052	-2.66224	-1.80921
C	2.96967	-3.65866	-2.79678
H	2.24501	-2.86181	-2.55525
H	2.43757	-4.62928	-2.79956
H	3.35425	-3.48057	-3.81845
C	5.18529	-4.72060	-2.13046
H	4.75418	-5.73995	-2.13126
H	6.02423	-4.71805	-1.41216
H	5.59847	-4.53587	-3.13982
C	3.01070	-1.88387	2.94537
H	3.72342	-1.11529	2.60361
C	1.60624	-1.23842	3.00489
H	0.86794	-1.96887	3.39147
H	1.25364	-0.89080	2.01319
H	1.60989	-0.37627	3.70006
C	3.44905	-2.35091	4.35063
H	4.44418	-2.82972	4.33810
H	2.73494	-3.07827	4.78156
H	3.49335	-1.48989	5.04272
C	7.27616	-1.69708	-0.73803
H	8.31364	-1.43593	-0.46221
H	6.95531	-1.03287	-1.55576
H	7.27280	-2.73062	-1.12724
C	6.60005	-2.93936	1.96898
H	6.07959	-2.83501	2.93438
H	7.68796	-2.89975	2.15752
H	6.35295	-3.93200	1.55627
C	7.02880	2.76043	1.45928
H	7.80296	2.57212	0.69803
H	7.51869	2.77690	2.44942
H	6.59683	3.75757	1.26942
C	4.67896	1.61048	3.03522
H	5.29016	1.34881	3.91759
H	3.78333	0.96884	3.03099
H	4.33934	2.65514	3.14836
C	4.69726	2.62881	-0.82351
C	3.67944	3.62333	-0.63091
C	3.66743	4.78289	-1.43131
H	2.87614	5.52635	-1.26855
C	4.62530	5.00424	-2.42626
H	4.59472	5.91187	-3.04220
C	5.61904	4.03785	-2.62620
H	6.37147	4.19345	-3.41062
C	5.67677	2.86546	-1.85019
C	2.58964	3.46697	0.42938
H	2.76122	2.49608	0.92424
C	2.67921	4.58078	1.49815
H	2.48689	5.57821	1.05876
H	3.67758	4.61539	1.97012
H	1.92851	4.41849	2.29339
C	1.18178	3.42021	-0.20131
H	1.10477	2.56478	-0.89170
H	0.95641	4.35229	-0.75410
H	0.40945	3.29490	0.57736
C	6.77521	1.84569	-2.14654
H	6.78667	1.14769	-1.29249
C	8.17848	2.47741	-2.27055
H	8.42692	3.09705	-1.39140
H	8.26322	3.12188	-3.16603
H	8.94786	1.68860	-2.36403
C	6.43850	1.02485	-3.41205
H	6.36376	1.68473	-4.29726
H	5.47380	0.50702	-3.28622
H	7.22372	0.27152	-3.61623
C	1.61671	-0.17788	-2.00415
H	0.73946	-0.30991	-2.67534
H	-0.78995	1.43043	-2.70981
C	3.04754	-0.05926	-2.52953
H	3.36067	-0.90023	-3.18662
H	3.23961	0.87275	-3.10523
C	1.47251	-0.10922	-0.61177
O	0.37553	-0.14854	0.10144
